# Targeting oxeiptosis-mediated tumor suppression: a novel approach to treat colorectal cancers by sanguinarine

**DOI:** 10.1038/s41420-023-01376-3

**Published:** 2023-03-13

**Authors:** Siraj Pallichankandy, Faisal Thayyullathil, Anees Rahman Cheratta, Karthikeyan Subburayan, Ameer Alakkal, Mehar Sultana, Nizar Drou, Muhammad Arshad, Saeed Tariq, Sehamuddin Galadari

**Affiliations:** 1 Cell Death Signaling Laboratory, Division of Science (Biology), Experimental Research Building, New York University Abu Dhabi, P.O. Box 129188, Abu Dhabi, UAE; 2 Center for Genomics and Systems Biology (CGSB), New York University Abu Dhabi, P.O. Box 129188, Abu Dhabi, UAE; 3 Bioinformatics Core, New York University Abu Dhabi, P.O. Box 129188, Abu Dhabi, UAE; 4 Department of Anatomy, College of Medicine and Health Sciences, UAE University, P.O. Box 17666, Al Ain, UAE

**Keywords:** Cell death, Natural products, Tumour-suppressor proteins

## Abstract

Oxeiptosis is a recently identified reactive oxygen species (ROS)-sensitive, caspase independent, non-inflammatory regulated cell death pathway. The activation of Kelch-like ECH-associated protein 1-Phosphoglycerate mutase 5-Apoptosis inducing factor mitochondria associated 1 (KEAP1-PGAM5-AIFM1) pathway is the key signaling event in the execution of oxeiptosis. In the present study, we demonstrate that sanguinarine (SNG), a quaternary benzophenanthridine alkaloid, induces oxeiptosis in human colorectal cancer (CRC) cells via ROS, specifically hydrogen peroxide (H_2_O_2_)-dependent activation of KEAP1-PGAM5-AIFM1 signaling axis. Whilst, knockdown of KEAP1, PGAM5, and AIFM1 largely abolishes SNG-induced oxeiptosis, hence reinforcing the importance of the role of this pathway in the SNG-mediated cytotoxicity. Moreover, extracellular addition of H_2_O_2_ sensitizes SNG-induced oxeiptosis in CRC cells, while removal of intracellular ROS by ROS scavengers, not only alleviated the overproduction of ROS caused by SNG, but also reversed the biochemical events associated with oxeiptosis. Finally, in vivo study demonstrates that SNG effectively reduces the tumor growth in HT-29 xenograft mouse model through features associated with oxeiptosis. This study highlights oxeiptosis as a novel tumor suppressive mechanism and further investigation of the role of oxeiptosis in cancer treatment is warranted.

## Introduction

Programmed or regulated death is a basic cellular process that orchestrates different pathological conditions and physiological process [1, 2]. Decades of studies have explored the functions and consequences of multiple modes of programmed cell death pathways such as apoptosis, autophagy, necroptosis, ferroptosis, entosis, pyroptosis, and oxeiptosis [3, 4].

Oxeiptosis is a recently identified cell death mechanism, which involves reactive oxygen species (ROS)-dependent activation of Kelch-like ECH-associated protein 1 (KEAP1)-Phosphoglycerate mutase family member 5 (PGAM5)-Apoptosis inducing factor mitochondria associated 1 (AIFM1), referred to as the (KEAP1-PGAM5-AIFM1) signaling axis [5]. KEAP1 is an endogenous inhibitor of nuclear factor erythroid-derived 2-like 2 (NRF2), a transcription factor that drives the expression of numerous cytoprotective antioxidant genes [6]. Under low oxidative stress, C-terminal cysteine residues on KEAP1 get oxidized, initiating a conformational change, and its dissociation from NRF2 [7, 8]. Consequently, NRF2 translocates to the nucleus and trans activate several cytoprotective antioxidative genes [6]. However, under high levels of oxidative stress, KEAP1 loses its interaction with another binding partner PGAM5, a mitochondrial serine-threonine phosphatase, and translocates PGAM5 into the mitochondria [5]. Once in the mitochondria, PGAM5 dephosphorylates AIFM1 at Ser^116^, finally leading to oxeiptotic cell death [5, 9, 10].

ROS is a signaling molecule central to various physiological and pathological processes. Depending on the type of ROS, its threshold, specific location, and cell type, ROS can activate distinct cell death pathways [11]. Hydrogen peroxide (H_2_O_2_) is the most prominent ROS molecule involved in the facilitation of cell death mechanisms such as oxeiptosis, apoptosis, autophagy, necrosis, and ferroptosis [5, 12–14]. However, it is not known how H_2_O_2_ regulates diverse cell death mechanisms. H_2_O_2_ is directly linked to oxeiptosis. In addition to H_2_O_2_, different stimuli such as ozone exposure, viral infection, and anticancer drug 5-Fluorouracil attached to magnetic nanoparticles have been established to induce oxeiptosis [5, 9, 10, 15, 16]. Recently, it has been shown that macrophages induce oxeiptosis in mesothelioma cells via ROS-dependent PGAM5 activation [17]. Thus, oxeiptosis seems to be functionally central to various biological activities ranging from viral infections, inflammation, and tumor suppression.

Colorectal cancer (CRC) has become one of the deadliest cancers affecting especially younger population [18]. CRC cells are notorious for their ability to evade apoptotic cell death [19]. This resistance limits the effectiveness of radiotherapy and chemotherapy routinely used in CRC treatment. Therefore, novel compounds and treatment methods are essential for the management of this disease. Over the last few years, the use of natural compounds as a genuine option for the management of different cancers has largely been accepted [20, 21]. Therefore, natural products and their structural analogs are massively being explored for drug discovery [22]. Sanguinarine (SNG) is a benzophenanthridine alkaloid isolated from *Sanguinaria Canadensis*. The antibacterial, anti-inflammatory, and antifungal activities of SNG have been well studied [23–25]. The anticancer activities of SNG have been a recent interest pertaining to its potent effect on diverse cellular events associated with initiation, promotion, and progression of different malignancies [14, 23–25]. To date, anticancer study on SNG was predominantly concentrated on its ability to stimulate apoptosis. Yet, whether SNG mediates its anticancer effect *via* activating oxeiptotic cell death signaling pathway has never been examined.

In this study, we have shown that SNG inhibits the growth of CRC cells *via* efficiently inducing oxeiptosis both in vitro and in vivo. Further, we assessed the molecular signaling mechanism behind SNG-mediated oxeiptosis. Indeed, this is a novel report demonstrating SNG induces oxeiptosis via H_2_O_2_-dependent activation of the KEAP1-PGAM5-AIFM1 signaling axis. This study offers the foundation for future investigations on SNG as a pro-oxeiptotic drug for the management of CRCs.

## Results

### SNG induces CRC cell death and modulates differential expression of distinct genes involved in multiple signaling pathways

**Fig. 1:**
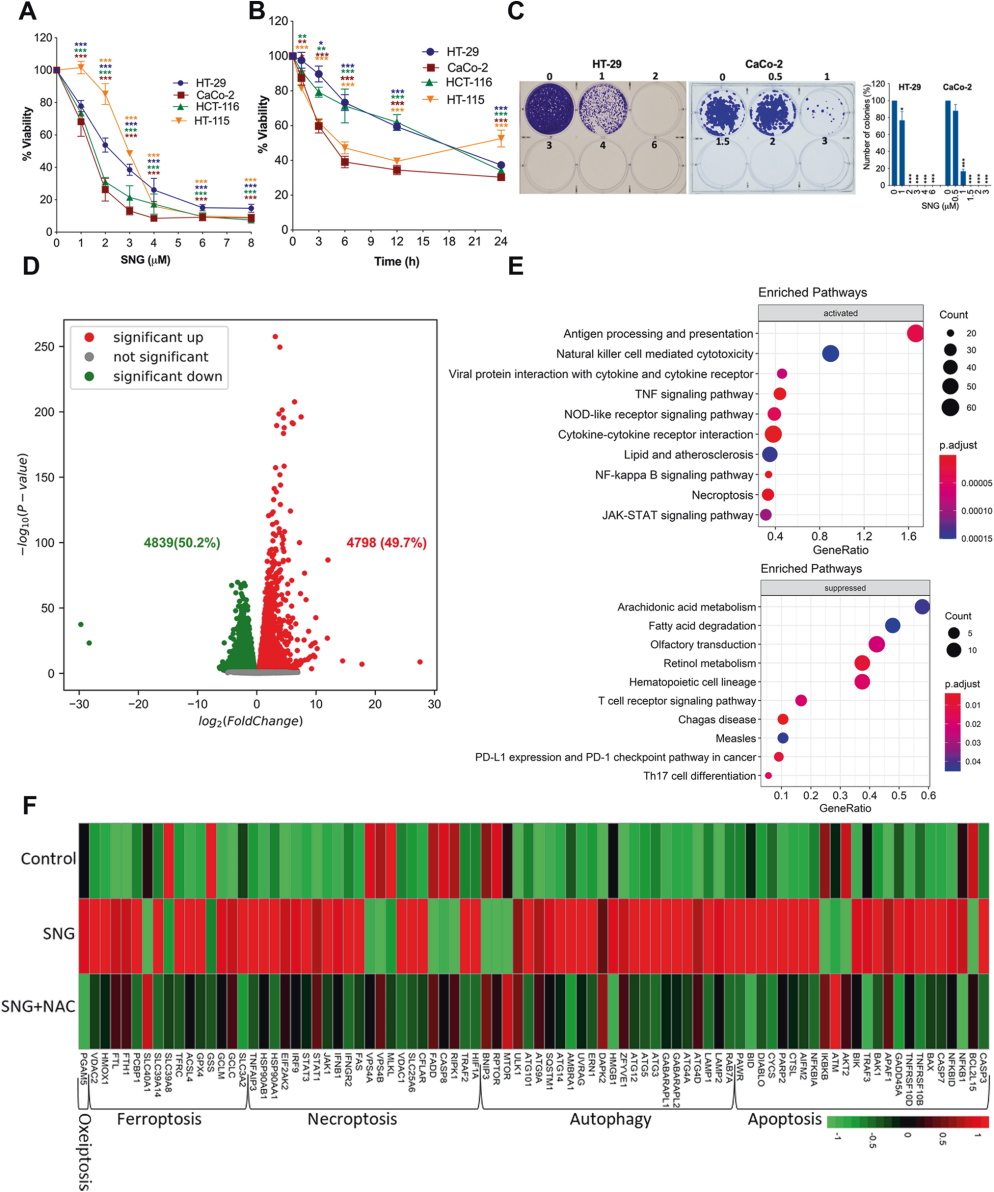
SNG inhibits proliferation and colony formation in multiple CRC cells and modulates differential expression of distinct genes involved in different cell death pathways. **A** CRC cells were treated with the indicated concentration of SNG for 24 h and **B** Cells were treated with SNG for the indicated time period and cell viability was assessed by MTT assay. Data shown are means ± SD (*n* = 3) (*p < 0.05, **p < 0.01, and ***p < 0.001 versus respective control). **C** Representative images of colony formation for cells treated with indicated concentrations of SNG for 16 h followed by 14 days culture and the colonies were counted. **D** HT-29 cells were treated with SNG (4 μM) for 16 h. Total RNA was isolated and subjected to RNA-seq analysis. The volcano plot shows the DEGs in SNG treated group compared to the control group. Red and green dots indicate upregulated 4798 (49.7%) and downregulated 4839 (50.2%) DEGs, respectively (corrected *P* value < 0.05). **E** The cellular signaling pathways triggered by SNG were obtained by KEGG pathway enrichment analysis. **F** The heat map illustrating the dynamic regulation of cell death associated genes by SNG in the presence or absence of NAC. The green-to-red gradient bar represents log2 values of fold-changes in the gene expression.

We and others have reported on the profound anticancer activities of SNG [14, 25–28]. Here, we examined the sensitivity of SNG on a panel of CRC cell lines (HT-29, HCT-116, CaCo-2, and HT-115). SNG effectively induced both dose- and time-dependent death in all the cell lines tested (Fig. 1A, B). In addition, long-term survival study using clonogenic assay showed a dose-dependent survival inhibition by SNG (Fig. 1C). Following SNG-treatment cells became rounded, swollen, and partially detached from the culture dish with appearance of apoptotic bodies (Supplementary Fig. 1).

To examine the molecular mechanisms of cell death induced by SNG, we treated HT-29 cells with SNG and carried out high-depth next-generation sequencing. The global gene expression profile revealed that a total of 9367 genes exhibited differential expression between control and SNG-treated groups (*p* value < 0.05) (Supplementary Table S1). Among them, 4798 (49.7%) genes were upregulated and 4839 (50.2%) genes were downregulated (Fig. 1D). In addition, the KEGG pathway enrichment analysis was carried out to identify the pathways affected by the SNG treatment. As a result, the top-ten significantly enriched pathways in both up-and downregulated differentially expressed genes (DEGs) are shown (Fig. 1E). The five most significant pathways among the upregulated DEGs are those involved in (i) antigen processing and presentation, (ii) natural killer cell mediated cytotoxicity, (iii) necroptosis pathway, (iv) tumor necrosis factor (TNF) signaling pathway, and (v) cytokine-cytokine receptor interaction (Fig. 1E), while those among the downregulated DEGs are (i) arachidonic acid metabolism, (ii) fatty acid degradation, (iii) olfactory transduction, (iv) retinol metabolism, and (v) hematopoietic cell lineage. Importantly, several genes involved in various cell death pathways such as apoptosis, autophagy, necroptosis, ferroptosis, and oxeiptosis were differentially modulated by the SNG treatment in HT-29 cells (Fig. 1F and Supplementary Table S2), suggesting concurrent activation of the multiple cell death pathways.

### SNG-induced human CRC cell death is not associated with classical cell death pathways

**Fig. 2:**
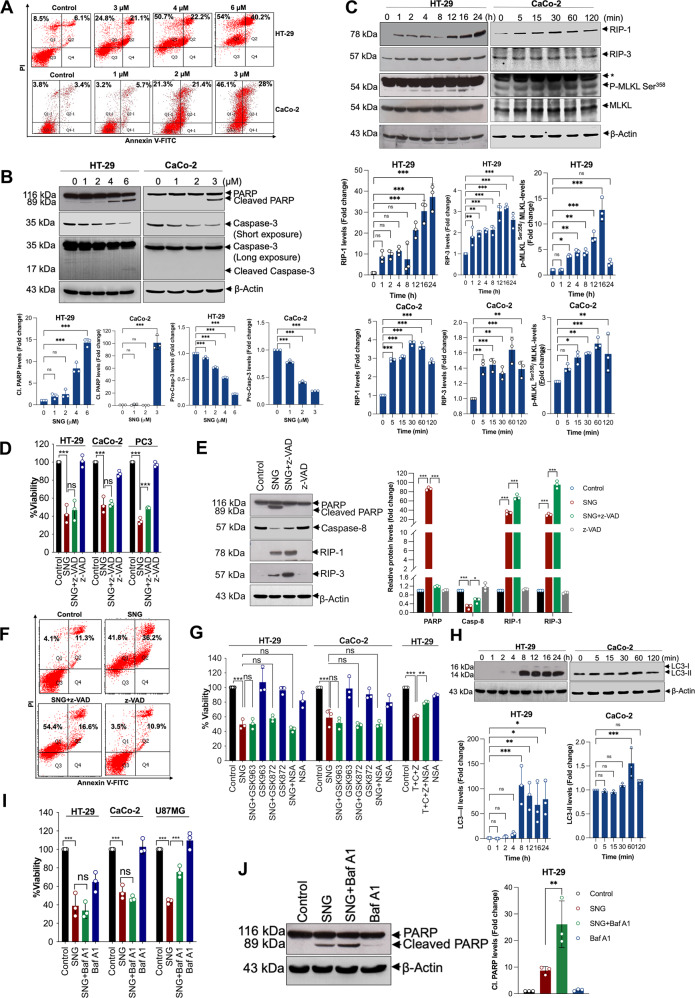
SNG-induced loss of viability in CRC cells is not associated with activation of classical cell death pathways. **A** Cells were treated with the indicated concentrations of SNG for 16 h. Apoptosis was determined by Annexin V-FITC apoptosis detection kit. Cells were treated with **B** indicated concentrations of SNG (HT-29 for 16 h and CaCo-2 cells for 6 h) **C** HT-29 and CaCo-2 cells were treated with SNG for the indicated time period. Western blot analysis was performed. The signal intensities of western blot bands were normalized to actin of each group, and fold changes were plotted in a histogram from three independent experiments. Significant difference, *p < 0.05, **p < 0.01, ***p < 0.001 and ns = no significance. The Blots shown here are representative of three independent experiments. **D** Cells were pretreated with z-VAD for 1 h followed by SNG treatment (HT-29 for 16 h and CaCo-2 for 6 h) and cell viability was measured by using MTT assay. Similar experiment in PC3 cells treated with SNG (3 μM) in the presence or absence of z-VAD were used as a positive control for apoptosis. Data shown are means ± SD (*n* = 3) (***p < 0.001 and ns = no significance). HT-29 cells were pretreated with z-VAD for 1 h followed by SNG treatment for a further 16 h, **E** Western blot analysis was performed. The signal intensities of western blot bands were normalized to actin of each group, and fold changes were plotted in a histogram from three independent experiments. Significant difference, *p < 0.05, ***p < 0.001. The blots shown here are representative of three independent experiments and **F** apoptosis was determined by Annexin V-FITC apoptosis detection kit. **G** Cells were pretreated with GSK963, GSK872, and NSA for 1 h, followed by incubation with SNG (HT-29 for 16 h and CaCo-2 for 6 h). Cell viability was measured by using MTT assay. Similar experiment in HT-29 cells treated with TCZ in the presence or absence of NSA was used as a positive control for necroptosis. Data shown are means ± SD (*n* = 3) (**p < 0.01, ***p < 0.001 and ns = no significance). **H** Cells were treated with SNG for the indicated time period, and Western blot analysis was performed. The signal intensities of western blot bands were normalized to actin of each group, and fold changes were plotted in a histogram from three independent experiments. Significant difference, *p < 0.05, **p < 0.01, ***p < 0.001 and ns = no significance. **I** HT-29 and CaCo-2 cells were pretreated with Baf A1 followed by incubation with SNG (HT-29 for 16 h and CaCo-2 for 6 h). Cell viability was measured by using MTT assay. Similar experiment in U87MG cells treated with SNG (3 μM) in the presence or absence of Baf A1 was used as a positive control for autophagy. Data shown are means ± SD (*n* = 3) (***p < 0.001 and ns = no significance). **J** HT-29 cells were pretreated with Baf A1 followed by incubation with SNG, and Western blot analysis was performed. The signal intensities of western blot bands were normalized to actin of each group, and fold changes were plotted in a histogram from three independent experiments. Significant difference, **p < 0.01.

Diverse classification of cell death pathways is emerging. Each cell death pathway provides an attractive target for developing new anticancer drugs [4]. Therefore, we sought to investigate modes of cell death induced by SNG. First, we assessed whether SNG treatment causes Annexin V/PI staining in CaCo-2 and HT-29 cells. Flow cytometry analysis revealed features of both apoptosis (Annexin V + PI-) and necroptosis (Annexin V-PI+) in SNG-treated cells (Fig. 2A). Furthermore, we have checked markers of apoptosis including cleavage of PARP and activation of caspase-3 on CRC cells. SNG clearly induced PARP cleavage and caspase-3 activation in both cell lines, while slight cleavage of caspase-3 was observed specifically in HT-29 cells (Fig. 2B). Additionally, the expression of necroptosis markers such as receptor-interacting protein-1 (RIP-1), RIP-3, and phosphorylated Mixed lineage kinase domain-like protein (MLKL) at Ser^358^ were also increased (Fig. 2C), suggesting that SNG can trigger both features of apoptosis and necroptosis in human CRC cells. Pre-treatment with z-VAD (a cell-permeable caspase inhibitor that hinders apoptosis) did not influence the SNG-induced cytotoxicity (Fig. 2D) and morphological changes (Supplementary Fig. 1), indicating that SNG-mediated tumor suppression is not connected with apoptosis. Whilst, pre-treatment with z-VAD completely inhibited SNG-induced PARP cleavage and reduction of pro-caspase-8 (Fig. 2E), portions of the Annexin V-PI + population (representing necroptosis) were significantly elevated in HT-29 cells (Fig. 2F), raising the possibility that inhibition of apoptosis potentiates necroptosis induction. This notion was further supported by elevated expression of necroptosis markers such as RIP-1 and RIP-3 in z-VAD pre-treated cells (Fig. 2E).

Since SNG triggered caspase-independent cell death with necroptotic features, we next investigated whether necroptosis is responsible for SNG-induced tumor suppression. Both GSK963 (inhibitor of RIP1 kinase) and GSK872 (inhibitor of RIP3 kinase) failed to suppress SNG-induced cell death (Fig. 2G). Furthermore, necrosufonamide (NSA), an inhibitor of MLKL, which is a key downstream effector of RIP-1 and RIP-3 kinase-dependent necroptosis, did not negate SNG-induced cytotoxicity (Fig. 2G). However, NSA effectively inhibited the TNF-α (100 ng/mL) + cycloheximide (5 μg/mL) + z-VAD (25 μM) (T + C + Z) induced classical necroptotic cell death in HT-29 cells, which was used as a positive control (Fig. 2G). Together, these data indicate that the canonical necroptosis pathway is not responsible for SNG-induced tumor suppression.

Autophagy is another important form of cell death mechanism [29]. Treatment of HT-29 and CaCo-2 cells with SNG demonstrated an increased expression of autophagic cell death marker microtubule-associated protein light chain (LC3-II) (Fig. 2H). However, pre-treatment with bafilomycin A1 (Baf A1), an agent that blocks autophagic proteolysis [30], did not protect from SNG-induced cytotoxicity in both CaCo-2 and HT-29 cells (Fig. 2I). On the contrary, Baf A1 slightly increased the caspase activation as evidenced by PARP cleavage (Fig. 2J). In addition, SNG-induced cytotoxicity was not modulated by ferroptosis inhibitors ferrostatin-1 (Fer-1) and deferoxamine mesylate (DFO) (Supplementary Fig. 2A). Furthermore, combining the inhibitors of apoptosis, necroptosis and autophagy did not prevent SNG-induced cytotoxicity (Supplementary Fig. 2B). Collectively, our results suggest that SNG-induced cell death could be mediated by a pathway independent of apoptosis, necroptosis, autophagy, and ferroptosis.

### SNG-induces ROS-dependent cell death

**Fig. 3:**
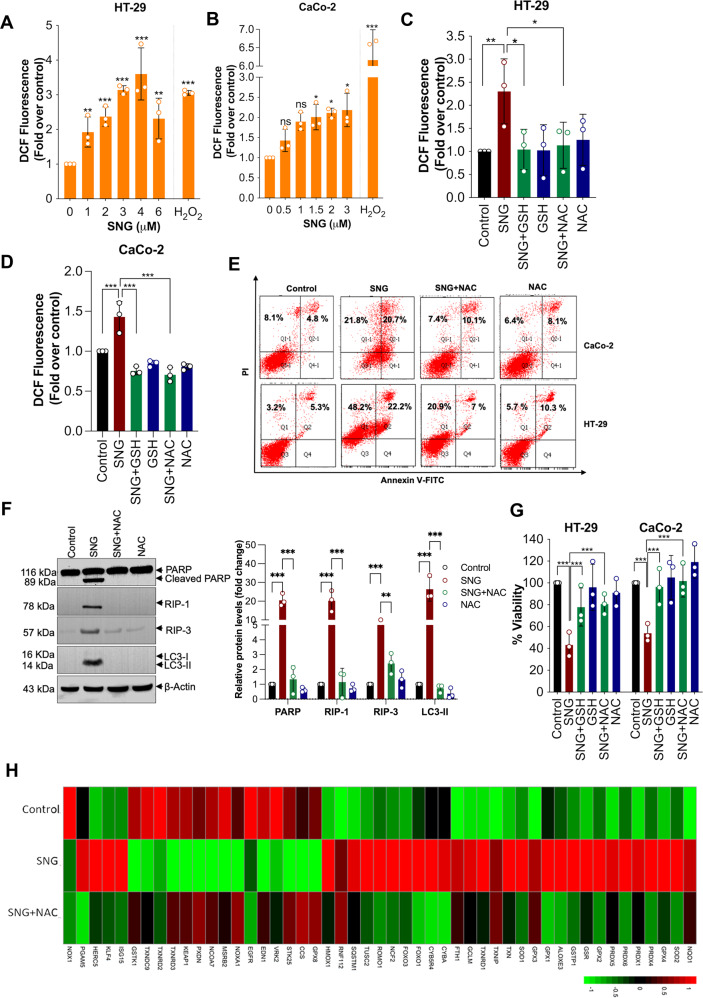
SNG-induced ROS-dependent cell death in CRC cells. **A** HT-29 and **B** CaCo-2 cells were treated with the indicated concentration of SNG. DCFH-DA-derived fluorescence was quantitated. H_2_O_2_ (500 μM) was used as a positive control. Data shown are means ± SD (*n* = 3) (*p < 0.05, **p < 0.01, ***p < 0.001 vs respective control, ns = no significance). **C** HT-29 and **D** CaCo-2 cells were treated with SNG in the presence or absence of GSH and NAC. ROS assay by using DCFH-DA was performed. Data shown are means ± SD (*n* = 3) (*p < 0.05, **p < 0.01, ***p < 0.001). **E** Cells were treated with SNG in the presence or absence of NAC. Apoptosis was determined by FACS analysis using Annexin V-FITC apoptosis detection kit. **F** HT-29 cells were pretreated with NAC followed by incubation with SNG for 16 h, and Western blot analysis was performed. The signal intensities of western blot bands were normalized to actin of each group, and fold changes were plotted in a histogram from three independent experiments. Significant difference, **p < 0.01 and ***p < 0.001. **G** Cells were treated with SNG in the *p*resence or absence of GSH and NAC. Following the treatment cell viability was measured by using MTT assay. Data shown are means ± SD (*n* = 3) (***p < 0.001). **H** Heat map illustrating dynamic regulation of ROS associated genes by SNG in the presence or absence of NAC. The green-to-red gradient bar represents log2 values of fold-changes in the gene expression.

Previously, we have demonstrated that oxidative damage plays a crucial role in the anticancer effect of SNG [14, 25–27]. While looking for a possible trigger for the SNG-induced cell death, we observed elevated levels of intracellular ROS, measured by 2′,7′-dichlorodihydrofluorescein diacetate (DCFH-DA) (a cell permeable probe that produces fluorescence in response to a broad spectrum of ROS molecules) (Fig. 3A, B). Interestingly, ROS inhibitors, such as N-acetyl-l-cysteine (NAC) and glutathione (GSH), significantly neutralized SNG-induced ROS generation (Fig. 3C, D), features of apoptosis, necroptosis, and autophagy (Fig. 3E, F), and cytotoxicity in CRC cells (Fig. 3G).

Since SNG treatment increases ROS generation, we sought to confirm this increase by analyzing the expression of several genes involved in ROS production and scavenging. First, we examined the levels of superoxide dismutases (SODs), an enzyme responsible for O_2_^•−^ dismutation into H_2_O_2_. Interestingly, SNG treatment significantly upregulated the expression of SOD1 and SOD2 (Fig. 3H, Supplemental Table S3). Subsequently, we analyzed other important ROS-related genes such as (i) interferon-stimulated gene 15 (ISG15), an interferon-induced ubiquitin-like protein which is involved in oxidative stress [31] (ii) HECT and RLD domain containing E3 ubiquitin protein ligase 5 (HERC5), an E3 ligase enzyme required for conjugation of ISG15 to a broad spectrum of target proteins in human cells [32], (iii) neutrophil cytosolic factor 2 (NCF2), a part of the NADPH oxidase complex that produces superoxide, and (iv) thioredoxin interaction protein (TXNIP), a thioredoxin-binding protein that can facilitate oxidative stress. ISG15, HERC5, NCF2, and TXNIP were significantly upregulated in SNG-treated HT-29 cells (Fig. 3H, Supplemental Table S3). At the same time, ROS scavenging systems were noticeably influenced, showing different expression patterns under SNG treatment (Fig. 3H, Supplemental Table S3). For example, PXDN, TXNRD2, TXNRD3, GSTK1, GPX8, GSTCD, GSTK1, and GSS expression were significantly downregulated; however, genes such as GPX1–4, PRDX1, PRDX4, PRDX5, PRDX6, GSR, and TXN were significantly upregulated. Next, we analyzed whether the difference in gene expression observed here on treatment with SNG is directly linked to ROS production. Notably, Pre-treatment with NAC significantly reversed the expression of various cell death pathway associated genes and oxidative stress-related genes (Figs. 1F and 3H), indicating that SNG-mediated cell death, at least in part, is mediated by increasing ROS generation.

### SNG-induced cell death is associated with features of oxeiptosis

**Fig. 4:**
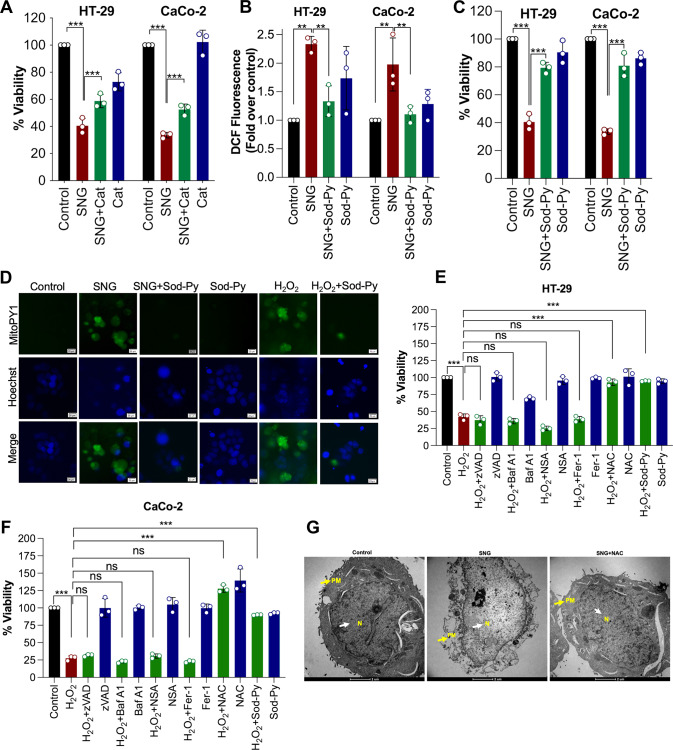
SNG-induced cell death is associated with the features of oxeiptosis. **A** **CRC** cells were treated with SNG in the presence or absence of Cat. Cell viability was assessed by MTT assay. Data shown are means ± SD (*n* = 3) (***p < 0.001). CRC cells were treated with SNG in the presence or absence of Sod-Py. **B** ROS assay was performed by using DCFH-DA and **C** Cell viability using MTT assay was carried out. Data shown are means ± SD (*n* = 3) (**p < 0.01 and ***p < 0.001) **D** HT-29 cells were treated with 10 µM MitoPY1 for 1 h at 37 °C. The medium was replaced with fresh growth media containing Hoechst 33342 dye (1.5 µl; 10 mg/ml stock solution). Following the treatment with SNG in the presence or absence of Sod-Py for 30 min, the fluorescence of Hoechst (blue), and MitoPY1 (green) was captured by fluorescent microscopy. H_2_O_2_ (1 mM) was used as a positive control. Scale bar: 20 µm. **E** HT-29 and **F** CaCo-2 cells were treated with H_2_O_2_ (500 µM) in the presence or absence of indicated inhibitors. Following the treatment, cell viability was performed by MTT assay. Data shown are means ± SD (*n* = 3) (***p < 0.001, ns = no significance). **G** Representative TEM images of HT-29 cells treated with SNG in the presence or absence of NAC for 16 h. The yellow arrowheads indicate plasma membrane and the white arrowhead indicates nucleus (PM, plasma membrane; N, nucleus). Scale bar: 2 µm.

Our results above demonstrate that SNG-induces ROS-dependent non-canonical cell death in human CRC cells. In order to identify the mechanism of cell death, we sought to determine whether SNG induces oxeiptosis, a recently identified ROS-sensitive caspase-independent cell death pathway [5]. H_2_O_2_ generation has been reported to play a leading role in the induction of oxeiptosis [5]. Therefore, we tested whether H_2_O_2_ is generated in response to SNG treatment in human CRC cells. HT-29 and CaCo-2 cells were pre-treated with scavengers specific for two major ROS molecules and tested for SNG-induced cell death. Pre-treatment with catalase (Cat) (H_2_O_2_ scavenger), but not SOD (O_2_^•−^ scavenger), significantly reduced SNG-induced cell death (Fig. 4A and Supplementary Fig. 3). The role of H_2_O_2_ in SNG-induced cytotoxicity was further established by using another well-known H_2_O_2_ scavenger sodium pyruvate (Sod-Py) [33]. Pre-treatment of cells with Sod-Py significantly attenuated SNG-induced ROS generation (Fig. 4B) and cytotoxicity (Fig. 4C). Importance of H_2_O_2_ in SNG-induced cytotoxicity was further confirmed by mitochondria peroxy yellow 1 (MitoPY1), a fluorescent probe for imaging H_2_O_2_ in living cells [34]. MitoPY1-derived fluorescence was significantly increased when the cells were treated with SNG and positive control H_2_O_2_. However, pre-treatment with Sod-Py completely blocked the MitoPY1-derived fluorescence (Fig. 4D), confirming that SNG induces H_2_O_2_ generation in CRC cells.

The role of H_2_O_2_ generation in CRC cell death was further confirmed by treating the cells with extracellular H_2_O_2_ and cell viability was measured. Comparable to the effect of SNG, cell death induced by H_2_O_2_ was not inhibited by any of the classical cell death pathway inhibitors, yet completely inhibited by ROS scavengers (Fig. 4E, F), indicating that SNG-induces H_2_O_2_-sensitive caspase-independent oxeiptotic cell death. Furthermore, transmission electron microscopy revealed features of oxeiptosis such as membrane blebbing and nuclear enlargement in SNG-treated HT-29 cells (Fig. 4G) [5]. These effects were reversed by pre-treatment with NAC (Fig. 4G). Taken together, our data strongly suggest that SNG induces oxeiptosis in CRC cells.

### SNG induces oxeiptosis via activation of KEAP1-PGAM5-AIFM1 signaling axis

**Fig. 5:**
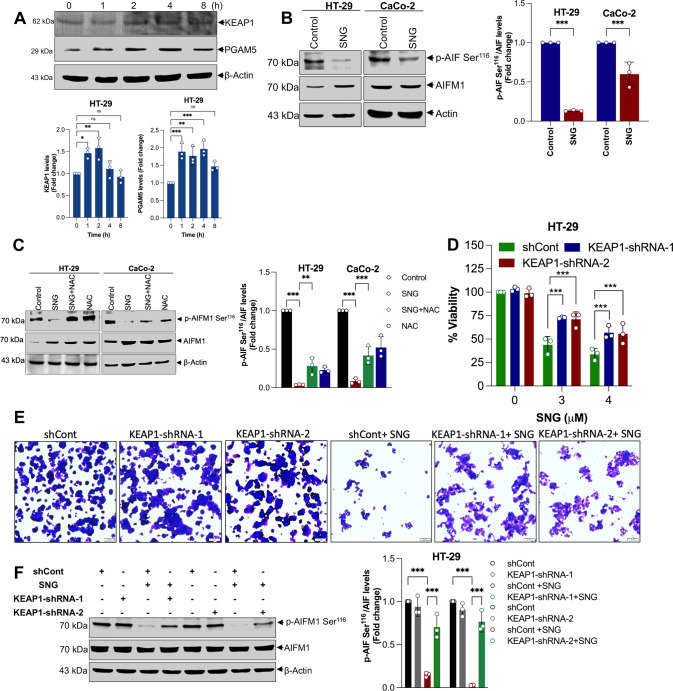
Activation of KEAP1-PGAM5-AIFM1 signaling axis in SNG-induced oxeiptosis. **A** HT-29 cells were treated with SNG for indicated time and Western blot analysis was carried out. The signal intensities of western blot bands were normalized to actin of each group, and fold changes were plotted in a histogram from three independent experiments. Significant difference, *p < 0.05, **p < 0.01, ***p < 0.001 and ns = no significance. **B** HT-29 and CaCo-2 cells were treated with SNG for 16 h and 6 h, respectively. Following the treatment, Western blot analysis of indicated proteins was performed. The signal intensities of western blot bands were normalized to AIFM1 of each group, and fold changes were plotted in a histogram from three independent experiments. Significant difference, ***p < 0.001. **C** Cells were treated with SNG in the presence or absence of NAC, and Western blot analysis of indicated proteins was performed. The signal intensities of western blot bands were normalized to AIFM1 of each group, and fold changes were plotted in a histogram from three independent experiments. Significant difference, **p < 0.01 and ***p < 0.001. KEAP1-shRNAs-transfected HT-29 cells were treated with the indicated concentrations of SNG for 16 h. Following the treatment, **D** cell viability was performed by MTT assay. Data shown are mean ± SD (*n* = 3) (***p < 0.001), **E** crystal violet staining, Scale bar: 10 µm, and **F** Western blot analysis of indicated proteins was performed. The signal intensities of western blot bands were normalized to AIFM1 of each group, and fold changes were plotted in a histogram from three independent experiments. Significant difference, ***p < 0.001.

**Fig. 6:**
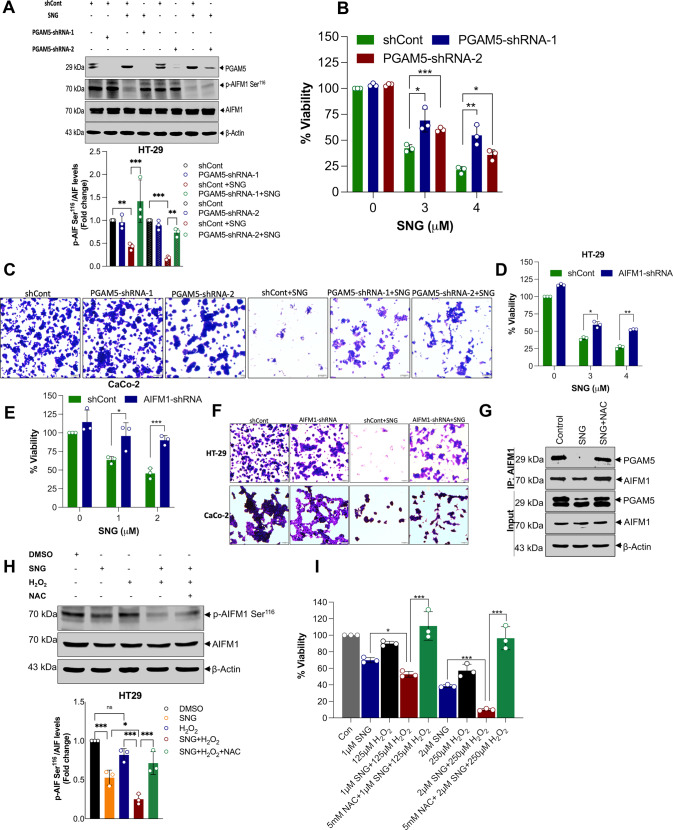
Pivotal role of KEAP1-PGAM5-AIFM1 signaling axis in SNG-induced oxeiptosis. CRC cells were stably transfected with PGAM5-shRNAs. After transfection, cells were treated with SNG (4 μM) for 16 h. Following the treatment, **A** Western blot analysis of indicated proteins was performed. The signal intensities of western blot bands were normalized to AIFM1 or actin of each group, and fold changes were plotted in a histogram from three independent experiments. Significant difference, **p < 0.01 and ***p < 0.001. **B** CRC cells were stably transfected with PGAM5-shRNAs. After transfection, cells were treated with the indicated concentration of SNG for 16 h and then cell viability was measured by MTT assay. Data shown are mean ± SD (*n* = 3) (*p < 0.05, **p < 0.01 and ***p < 0.001) and **C** crystal violet staining was performed. Scale bar: 10 µm. Indicated cells were stably transfected with AIFM1-shRNA, after transfection, cells were treated with the indicated concentrations of SNG (HT-29 for 16 h and CaCo-2 for 6 h), respectively. Following the treatment, **D** and **E** cell viability was measured by MTT assay. Data shown are mean ± SD (*n* = 3) (*p < 0.05, **p < 0.01 and ***p < 0.001), and **F** crystal violet staining was performed. Scale bar: 10 µm. **G** HT-29 cells were treated with SNG in the presence or absence of NAC for 16 h. Whole cell lysates of treated as well as untreated cells were subjected to immunoprecipitation with the respective antibodies as indicated, followed by detection precipitates (top) and input lysates (bottom) with the appropriate antibodies by Western blotting. HT-29 cells co-treated with H_2_O_2_ (250 μM) were exposed to SNG (2 μM) in the presence or absence of NAC. **H** Western blot analysis of indicated proteins was performed. The signal intensities of western blot bands were normalized to AIFM1 of each group, and fold changes were plotted in a histogram from three independent experiments. Significant difference, *p < 0.05, ***p < 0.001 and ns = no significance, **I** Cell viability was measured by using MTT assay. Data shown are mean ± SD (*n* = 3) (*p < 0.05, ***p < 0.001).

KEAP1 is a major sensor of oxidative stress. In response to minor ROS generation, KEAP1 activates transcription factor NRF2 (which is responsible for the expression of various antioxidant enzymes) as a cellular protectant, whereas when the ROS levels are high, KEAP1 might release PGAM5 (a mitochondrial serine-threonine phosphatase). Once released, PGAM5 dephosphorylates AIFM1 at Ser^116^, thus initiating the oxeiptosis death signaling [5]. Interestingly, SNG significantly induced the expression of PGAM5, and KEAP1 (Figs. 1F and 5A). PGAM5 mediated-dephosphorylation of AIFM1 at Ser^116^ is considered as a distinguishing feature for cells undergoing oxeiptosis [5]. SNG induces AIFM1 dephosphorylation at Ser^116^ (Fig. 5B), which was inhibited by ROS scavenger NAC (Fig. 5C). Similar to SNG, H_2_O_2_ also induced the AIFM1 dephosphorylation at Ser^116^ in HeLa and Jurkat cells which was used as a positive control for the oxeiptosis (Supplementary Fig. 4). Together, these data indicate that SNG-induces the activation of KEAP1-PGAM5-AIFM1 signaling axis in CRC cells. To further study the potential role of this signaling axis in SNG-induced oxeiptosis, we depleted HT-29 cells of KEAP1 with two independent shRNAs (Supplementary Fig. 5) and evaluated their survival after the SNG treatment. As expected, KEAP1 depletion significantly decreased SNG-induced cytotoxicity (Fig. 5D, E) and AIFM1 dephosphorylation at Ser^116^ (Fig. 5F), suggesting that KEAP1 plays a critical role in SNG-induced ROS-dependent cell death pathway. Next, we checked the phosphorylation status of AIFM1 Ser^116^ and cell survival following the depletion of two non-overlapping shRNAs against PGAM5 in HT-29 cells. Depletion of PGAM5 significantly reversed the SNG-induced AIFM1 dephosphorylation and cell death (Fig. 6A–C), further reinforcing the involvement of KEAP1-PGAM5-AIFM1 signaling axis in SNG-induced CRC cell death.

To further confirm the role of oxeiptosis in SNG-induced cell death, we downregulated the expression of AIFM1 using shRNA (Supplementary Fig. 6) and checked the cell viability. Downregulation of AIFM1 significantly reverted the SNG-induced loss of viability in both CRC cells (Fig. 6D–F). Next, we examined the functional interaction between AIFM1 and PGAM5 in SNG-treated cells by Immunoprecipitation (IP) analysis. The PGAM5 binding to AIFM1 was remarkably reduced when the cells were subjected to SNG treatment, and this binding was restored when pre-treated with NAC (Fig. 6G), suggesting that SNG-induced H_2_O_2_ can modulate the association between PGAM5 and AIFM1. It should be noted that during the SNG treatment interaction between AIFM1 and PGAM5 is reduced. This may be because various studies have revealed that processed substrates often lose interaction with their modifying enzymes [35].

Our aforementioned results prompted us to examine whether dephosphorylation of AIFM1 with H_2_O_2_ would sensitize CRC cells to SNG, thus promoting oxeiptosis. CRC cells were co-treated with a low concentration of H_2_O_2_ and SNG in the presence or absence of NAC. As expected, our results showed that SNG induced AIFM1 dephosphorylation at Ser^116^ in HT-29 cells. Interestingly, the addition of H_2_O_2_ potentiated the SNG-induced AIFM1 dephosphorylation at Ser^116^ and cell death, this effect was significantly inhibited by pretreatment with NAC (Fig. 6H, I), further strengthening the evidence that SNG is a potent oxeiptosis inducer.

### Oxeiptosis is involved in SNG-induced tumor suppression in vivo

**Fig. 7:**
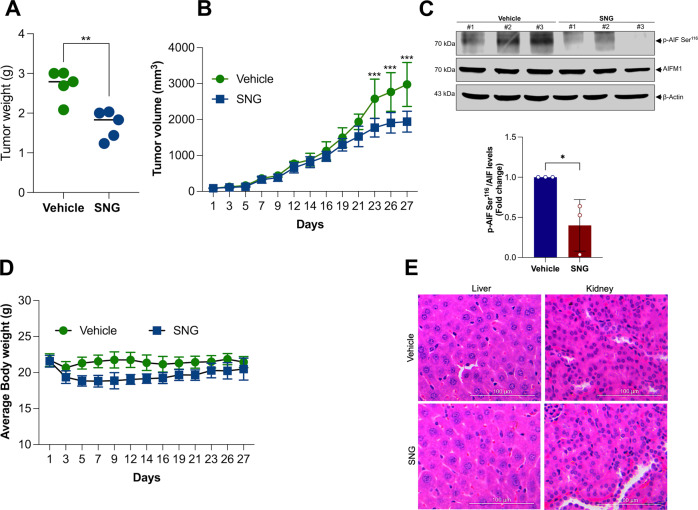
Oxeiptosis is involved in SNG-induced tumor suppression in vivo. Mice were subcutaneously inoculated with HT-29 cells into the right flanks and randomly divided into two groups (*n* = 5). Mice were injected intraperitoneally (i.p.) with 6 mg/kg/ SNG or an equal volume of vehicle. **A** Individual value plot showing the weights of HT-29 tumor xenografts in the vehicle and SNG treatment groups. Data shown are mean ± SD (*n* = 5) (**p < 0.01). **B** Tumor volumes of HT-29 xenograft tumors with the different time points (days) after exposure to SNG. Data shown are mean ± SD (*n* = 5) (***p < 0.001). **C** Western blot analysis of indicated proteins was performed. The signal intensities of western blot bands were normalized to AIFM1 of each group, and fold changes were plotted in a histogram from three independent experiments. Significant difference, *p < 0.05 **D** The relative body weight was evaluated during the treatment. Data shown are mean ± SD (*n* = 5). **E** H&E-stained liver and kidney sections obtained from the mice treated with vehicle and SNG are shown. Scale bar: 100 µm.

Next, we sought to determine the importance of oxeiptosis in SNG-induced tumor suppression in vivo. Therefore, NCr nude mice were inoculated with HT-29 cells subcutaneously and injected with either vehicle or SNG (6 mg/kg, intraperitoneally) every three days for a period of 24 days. As expected, SNG potently suppressed HT-29 xenograft tumor growth after 3 weeks of treatment (Fig. 7A, B). Notably, excised tumor tissue from HT-29 xenografts demonstrated dephosphorylation of AIFM1 at Ser^116^ in SNG-treated mice, which is indicative of oxeiptosis (Fig. 7C). SNG-treated mice did not exhibit any major change in the body weight compared with vehicle-treated mice throughout the study period (Fig. 7D). To further evaluate the toxicity of SNG, mice liver and kidney were subjected to histopathological examination following H&E staining. No significant morphological difference was observed in SNG-treated samples compared to control samples (Fig. 7E). These findings suggest that SNG efficiently suppresses tumor growth in the HT-29 xenograft mouse model without producing any added toxic effect on normal tissues like liver and kidney. Thus, mirroring our in vitro data, our in vivo data further suggest that oxeiptosis is at least partly responsible for SNG-induced tumor suppression.

## Discussion

Oxeiptosis is a newly discovered ROS-sensitive, non-canonical cell death mechanism that plays an important role in protection from inflammation induced by ROS, viral pathogens, ozone exposure, and many other biological processes [5, 10, 16, 17]. In this study, we have disclosed that a chemotherapeutic agent, SNG induces oxeiptosis-mediated tumor suppression in human CRC cells. Mechanistically, we have demonstrated that SNG induces H_2_O_2_ production (a hallmark of oxeiptosis), which in turn leads to KEAP1-PGAM5 signaling-axis activation and AIFM1 dephosphorylation at Ser^116^, finally leading to robust oxeiptosis induction as a consequence. Several lines of evidence in this study have demonstrated the SNG-mediated oxeiptosis in CRC cells. For instance, (1) SNG causes significant ROS accumulation which is detectable with a dose as low as 1 µM concentration, (2) pre-treatment of cells with ROS inhibitors almost entirely abrogates SNG-induced ROS generation, features of oxeiptosis, and loss of viability, and (3) SNG induces ROS-dependent activation of the KEAP1 and PGAM5-dependent dephosphorylation of AIFM1at Ser^116^, which is considered as a readout for the oxeiptosis [5, 9]. ROS are highly reactive short-lived molecules. ROS levels in the cell are maintained in equilibrium with a variety of ROS-scavenging systems [36]. At low to modest levels, ROS are considered to be important for the regulation of various physiological functions such as proliferation, cell cycle progress, and differentiation, to name a few. On the contrary, if the antioxidant detoxification system is unable to sustain tolerated levels of ROS, then the excess cellular ROS can have a devastating effect [36].

Unlike other forms of apoptotic and non-apoptotic cell death pathways [37, 38] the requirement for ROS accumulation appears to be decisive and universal in oxeiptosis. In the present study, SNG treatment of CRC cells also elicited features of apoptotic, necroptotic, and autophagic cell death. However, inhibiting any of these classical cell death pathways did not block the SNG-mediated cytotoxicity. A similar response was also observed in H_2_O_2_ exposure to cultured fibroblasts or ozone exposure in mice [5] and glucose deprivation-induced cell death in cancer cells [39]. On the contrary, SNG-induced CRC cell death was completely inhibited by both general (GSH and NAC) and H_2_O_2_-specific (Cat and Sod-Py) ROS scavengers. Moreover, extracellular H_2_O_2_-induced cell death in CRC cells was not prevented by inhibitors of any of the classical cell death pathways, yet significantly inhibited by the ROS scavengers (NAC and Sod-Py), reinforcing that SNG activates an alternative ROS-dependent cell death pathway. Thus, it is reasonable to assume that CRC cells under oxidative stress could undergo cell death through oxeiptosis.

**Fig. 8:**
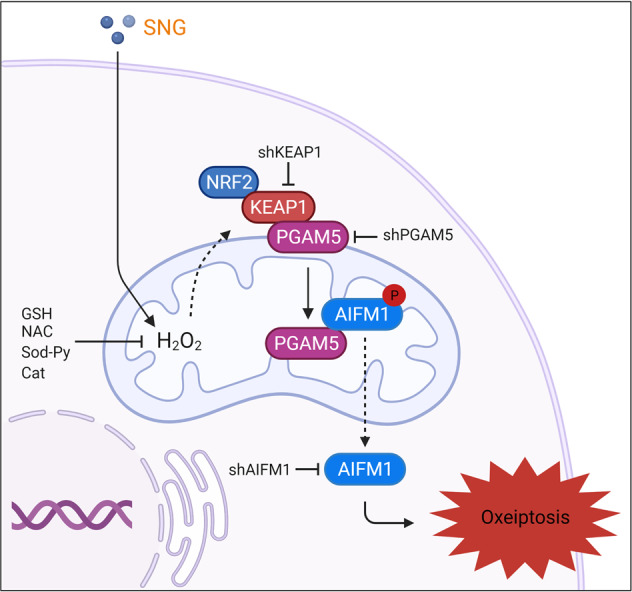
Summary diagram. Schematic diagram of the pathway in which SNG induces oxeiptosis in human colorectal cancer cells.

Oxeiptosis is characterized by the overwhelming accumulation of ROS (specifically H_2_O_2_) followed by KEAP1-PGAM5-AIFM1 signaling activation. The KEAP1-NRF2 signaling pathway has been recognized to facilitate cryoprotection against oxidative stress [5]. On the contrary, KEAP1 when hyperactivated can mediate H_2_O_2_-induced oxeiptosis independent of NRF2, through a pathway that involves PGAM5 (KEAP1 interaction partner), which causes AIFM1 at Ser^116^ dephosphorylation [5]. Dephosphorylated AIFM1-dependent oxeiptosis does not need the translocation of AIFM1 from mitochondria to the nucleus [5]. In this study, we have found that SNG-induced ROS can activate KEAP1-PGAM5-mediated dephosphorylation of AIFM1 at Ser^116^. Moreover, ROS scavengers or shRNA-mediated inhibition of KEAP1 and PGAM5 significantly inhibited the SNG-induced AIFM1 dephosphorylation at Ser^116^ and cell death. Together, our data suggest that SNG mediates H_2_O_2_-induced activation of KEAP1-PGAM5-AIFM1 pathway ultimately leading to oxeiptosis (Fig. 8).

Defective apoptosis in CRC contributes to pathogenesis and therapeutic resistance [40]. Therefore, new therapeutic agents or strategies are required to treat this devastating disease. In recent years, a large number of naturally occurring compounds from plants known as phytochemicals, are successfully employed in the development of novel anticancer drugs [20]. The anticancer properties of SNG have attracted interest among cancer researchers [27, 28, 41, 42]. Besides activating various death signaling pathways, SNG suppresses multiple signaling pathways and inhibits cell proliferation, angiogenesis, invasion, and metastasis of various tumors [25]. Furthermore, SNG could also synergistically augments the tumoricidal function of several anticancer drugs [25, 43]. Therefore, SNG has the potential to become a promising anti-cancer drug. From our in vivo data, SNG efficiently reduced tumor growth, without causing any significant hepatotoxicity and nephrotoxicity, indicating that SNG is a promising therapeutic lead in the treatment of CRC. Identifying and elucidating the mechanism by which SNG mediates its anticancer functions is essential to building a solid groundwork for its use as an anticancer drug. Previously, we have shown that SNG induces H_2_O_2_-dependent apoptosis in human leukemic and prostate cancer cells [26, 27] and H_2_O_2_-dependent autophagic cell death in human malignant glioma cells [14]. In the present study, and for the first time, we report that SNG induces H_2_O_2_-dependent oxeiptosis in human CRC cells. It is possible that additional mechanisms are also involved in SNG-induced oxeiptosis; clearly, studying such mechanisms will remain an important area of our future investigations.

Altogether, our results reveal that SNG is a potent oxeiptosis inducer in CRC cell lines and xenograft tumors. Our study provides a broader framework for further understanding and targeting oxeiptosis in cancer therapy. Finally, we envision that a better understanding of the molecular and genetic basis of oxeiptotic cell death signaling pathways may indeed improve the efficacy of cancer therapy and bypass resistance.

## Materials and methods

### Reagents

3-[4,5-dimethylthiazol-2-yl]-2,5-diphenyl tetrazolium bromide (MTT), glutathione (GSH), N-acetyl-l-cysteine (NAC), SNG, SOD, Cat, Baf A1, DFO, Fer-1, DCFH-DA, MitoPY1, crystal violet, Hoechst 33342, and dimethyl sulfoxide (DMSO) were obtained from Sigma Chemical Co. (St. Louis, MO, USA). GSK963 was purchased from Glixx Laboratories (Hopkinton, MA, USA), GSK872 was from Merck Millipore (Massachusetts, USA), NSA was from Calbiochem (San Diego, CA, USA), and z-VAD was from Enzo Life Sciences (Switzerland). Dulbecco’s modified essential medium (DMEM), Opti-MEM, McCoy’s 5A medium, penicillin-streptomycin (10,000 U/mL), phosphate-buffered saline (PBS), Sod-Py, trypsin-EDTA, and fetal bovine serum (FBS) were purchased from Gibco BRL (Grand Island, NY, USA). Anti- LC3-II (D11) XP (#3868), anti-KEAP1 (#8047S), anti-PARP (9542L), anti-caspase-3 (#9662S), anti-RIP-1 (#3493S), anti-RIP-3 (#13526S), anti-MLKL (#14993S), and anti-phospho-MLKL-Ser^358^ (#91689S) antibodies were purchased from Cell Signaling Technology (Beverly, MA, USA). Anti-PGAM5 (#HPA036978), anti-mouse IgG (#A0412), and anti-rabbit IgG (#A6154) were from Sigma Chemical Co. (St. Louis, MO, USA). Anti-actin (#sc-1616; #sc-47778) and anti-AIFM1 (#sc-9416) antibodies were from Santa Cruz Biotechnology Inc. (Santa Cruz, CA, USA). Anti-phospho-AIFM1 Ser^116^ (#AP5501) was purchased from ECM Biosciences (Versaolles, KY, USA). Peroxidase affinity pure goat anti-mouse HRP (#115-035-003) was purchased from Jackson Immuno Research Europe Ltd. (Cambridge House, UK).

### Cell culture conditions and drug treatment

Human CRC cell lines (CaCo-2, HCT-116, and HT-115), were procured from The European Collection of Authenticated Cell Cultures (ECACC). Human CRC cell line HT-29, human T-cell leukemia cells (Jurkat), human prostate cancer cells (PC3), human cervical cancer cells (HeLa), and human malignant glioma cell line (U87MG) were procured from ATCC (Rockville, MD, USA). HT-29 and HCT-116 cells were grown in McCoy’s 5 A media. CaCo-2, HT-115, HeLa, and U87MG cells were grown in DMEM. PC3 and Jurkat cells were cultured in RPMI 1640 GlutaMAX media. All cells were grown in an incubator containing 95% humidified atmosphere and 5% CO_2_ at 37 °C. Cells were supplemented with heat-inactivated FBS (10%), penicillin (50 IU/mL), and streptomycin (50 μg/mL). Cells were quarterly tested for mycoplasma contamination using the first generation MycoAlert^TM^ mycoplasma detection kit (Lonza, #LT07-118). SNG (10 mM) stock in DMSO was prepared and stored in an amber-colored bottle. Desired dilutions were made from this stock and were used to treat the cells (at about 70–80% confluence).

### Pre-treatment with inhibitors/scavengers

To examine the different signaling pathways regulated by SNG, cells were pre-treated (1 h prior to the SNG treatment) with different inhibitors/scavengers such as z-VAD (50 μM), GSK963 (10 μM), GSK872 (10 μM), NSA (10 μM), Baf A1 (250 nM), Fer-1 (10 μM), DFO (50 μM), GSH (5 mM), NAC (5 mM), Sod-Py (5 mM for HT-29 and 2 mM for CaCo-2), Cat (1000 U), and SOD (500 U). The SNG concentration of 3 μM (HT-29, HCT-116, and HT-115) and 1.5 μM (CaCo-2) were used, unless stated otherwise.

### Cell viability assay

Viability assay was performed as reported [44]. Briefly, cells (8000 cells/well) were seeded in 96-well plates and exposed to SNG. When the treatment is over, MTT (25 μL of 5 mg/mL in PBS) was added to each well and incubated at 37 °C for 2 h. The formazan crystals formed were solubilized in DMSO and the absorbance (570 nm wavelength) was quantified using EnSpire™ multimode plate reader (PerkinElmer, Waltham, USA).

### Colony formation assay

Cells (500 cells/well) seeded in 6-well culture plates were treated with SNG at different concentrations for 16 h. The cells were rinsed with PBS, and incubated for 2 weeks in a complete growth medium (without SNG), replacing the medium on every two days. The colonies obtained were washed with PBS and stained with crystal violet staining solution (50% methanol and 0.25% crystal violet) for 30 min at room temperature. After the incubation, plates were washed twice with PBS, images were taken under a microscope, and visible colonies were manually counted.

### Quantification of apoptosis and necroptosis

Quantification of SNG-induced apoptosis or necroptosis was performed according to the manufacturer’s protocol as described in the Annexin V-FITC/ propidium iodide (PI) apoptosis detection kit (BD Pharmingen, USA). Briefly, SNG-treated cells were harvested by centrifugation, washed twice with PBS, and stained with Annexin V-FITC/ PI. Following incubation, apoptosis and necroptosis were quantified using BD FACSAria III and FACS Diva software (BD Biosciences, NJ, USA).

### Western blot analysis

Following the treatment, cells were PBS washed and lysed using RIPA lysis buffer (50 mM Tris HCl pH 7.4, 40 mM NaF, 10 mM Na3VO4, 1% NP-40, 1 mM phenylmethylsulfonyl fluoride, 10 mM NaCl, 10 mM dithiothreitol, and EDTA-free protease inhibitor tablet). Total protein concentration of the lysates was estimated and the volume was equalized using loading buffer. The sample was boiled for 3 min at 100 °C and each sample/lane was resolved by SDS-PAGE. The proteins resolved were wet-transferred onto nitrocellulose membrane and the membranes were blocked with 5% non-fat milk in Tris-buffered saline containing 0.1% Tween-20. Blots were then placed in the primary antibody solution followed by a conjugated secondary antibody solution. Protein bands were visualized using Super Signal West Pico Chemiluminescence substrate (Thermo Scientific, CA, USA). The relative western blot band intensity was quantified using Image Studio Lite 5.2.5 software (LI-COR Biosciences).

### Intracellular ROS measurement

ROS levels were assessed by a ROS-sensitive fluorescent probe (DCFH-DA). Briefly, SNG-treated cells were PBS-washed and stained with DCFH-DA for 30 min. After washing 3–4 times with PBS, cells were harvested (in phenol red-free medium) and fluorescence intensity was measured using an EnSpire™ multimode plate reader (PerkinElmer, Waltham, USA). An excitation wavelength of 485 nm and an emission wavelength of 535 nm was used.

### Mitochondrial H_2_O_2_ measurement

Mitochondrial-derived H_2_O_2_ was detected using MitoPY1 (a specific fluorescent probe that selectively tracks mitochondrial-derived H_2_O_2_) as described previously [34]. Briefly, cells exposed to MitoPY1 (10 μM for 1 h) were PBS washed and treated with SNG (in the presence or absence of inhibitors/scavengers). Following PBS washing, phenol red-free medium was added and mitochondrial-derived H_2_O_2_ fluorescence was photographed using IX73 inverted fluorescent microscope (Olympus, Tokyo, Japan).

### Immunoprecipitation

IP assay was carried out using Pierce Direct IP kit (Thermo Scientific, Rockford, IL, USA) as instructed by the manufacturer’s protocol. Briefly, primary antibody (5 µg) against human AIFM1 was immobilized chemically onto the coupling resin. Cells lysed in IP lysis buffer were protein assayed and 400 µg total protein was added to the antibody-coupled resin in the spin column. The column was incubated overnight at 4˚ C in a rotating platform. Eluted proteins were subjected to western blot analysis to verify the protein.

### shRNA-mediated knockdown

shRNAs for PGAM5 (TL318057), KEAP1 (TL303778), AIFM1 (TL302572) and shCont (TR30021) constructs in lentiviral GFP vector were procured from OriGene Technologies (Rockville, MD, USA):

PGAM5-shRNA-1: ATCACAGCAATGAACACCATCCGAAGCGG

PGAM5-shRNA-2: CCAAGCAAGAGGAGTTCTTCAACCTGTCC

KEAP1-shRNA-1: GAACCACTGTCTCTGATCAACGTGCGGAA

KEAP1-shRNA-2: CCAACGTCATCCGCTACATCGTGTGCAGC

AIFM1-shRNA: TACTGGCATCAGTCAATGTTCTGGAGTGA

shCont: GCACTACCAGAGCTAACTCAGATAGTACT

Using Lenti-Pac expression packing kit (Genecopoeia, Rockville, MD, USA) lentiviruses carrying shRNAs were made in Lenti HEK-293Ta cells according to the manufacturer’s instructions. HT-29 and CaCo-2 cells were transduced by these viruses and puromycin selection (0.3 μg/mL for HT-29 and 0.15 μg/mL for CaCo-2 cells) was carried out in order to make the stable cell lines. Scrambled shRNA sequence was used as a negative control for shRNA-targeted experiments.

### RNA sequencing and data analysis

Whole-Transcriptome gene expression analysis was performed in HT-29 cells (*n* = 4 per group). The total RNA was obtained using RNeasy extraction kit (Qiagen). For QC of extracted RNA, absorbance ratios were evaluated with Nanodrop 2000. The integrity of RNA was assessed based on RIN acquired *via* electrophoresis Bioanalyzer instrument (Agilent Technologies, Inc., Santa Clara, CA, USA) and quantified with Qubit 4 Fluorometer.

Libraries were prepared using 250 ng of RNA with the NEBNext Ultra II RNA Library Prep Kit#E7770L (New England Biolabs, Ipswich, MA, USA), following the manufacturer’s protocol, using oligo-dT beads to capture RNA containing an enriched polyA tail. The RNA is chemically fragmented and randomly primed for reverse transcription, followed by second-strand cDNA synthesis, with end-repair, A-base tailing, and adapter ligation consecutively. Fragments were amplified by 12 cycles of PCR, resulting in a 350–400 bp fragment including adapters. The fragment size and purity of the libraries were assessed on a 2100 electrophoresis Bioanalyzer instrument. (Agilent Technologies, Inc., Santa Clara, CA, USA). Quantification of the pooled libraries was determined by real-time PCR using a Kapa kit (Kapa Biosystems, Inc. Wilmington, MA, USA), and libraries were sequenced using the Illumina NextSeq 500/550 system with a Paired-end MO V2 kit, 150 cycles (Illumina, Inc, San Diego, CA, USA).

First, the quality of raw FASTQ sequences was assessed with FastQC [45] v0.11.5 followed by the quality trimming and adapter sequences removal with Trimmomatic [46] v0.36 using these parameters (*ILLUMINACLIP: trimmomatic_adapter.fa:2:30:10 TRAILING:3 LEADING:3 SLIDINGWINDOW:4:15 MINLEN:36*). Trimmed read pairs were then analyzed with Fastp [47] to remove poly-G tails and Novaseq/Nextseq specific artefacts. Finally, the quality of the surviving reads was checked again with FastQC.

After performing QC and QT, the surviving high-quality reads were mapped to the human reference genome GRCh38.p4 with HISAT2 [48] using all the default settings except that the *–dta* flag was provided, and the SAM files were generated. Next, SAMtools [49] v1.3.1 was used for converting the SAM alignments into BAM format and coordinate sorting. The HTSeq-count [45] v0.6.1p1 pipeline was run on the sorted BAM files with the following options (*-s no -t exon -I gene_id*) for reads count generation. Subsequently, the transcriptome quantification step was performed with Stringtie [50] v1.3.0 using the sorted alignments. Briefly, the process looked as follows: stringtie -> stringtie merge (a merged transcriptome GTF file was generated with all the samples) -> stringtie (with the GTF file generated in the previous step). Finally, Qualimap [51] v2.2.2 tool was employed to produce RNAseq specific QC metrics for each sample.

Raw reads counts were used to identify DEGs between control and SNG and SNG + NAC with DESeq2 using NASQAR; an online platform for RNA-seq data analysis [52] Surrogate variable analysis was performed for hidden batch-effect correction before feeding the data into DESeq2. All the DEGs were used for Gene Set Enrichment Analysis with padj value < 0.05 as the threshold to determine significant KEGG pathways. Heatmaps were generated with the selected significant DEGs associated with oxidative stress and cell death pathways. Bioinfo-Kit [53] and pheatmap [54] packages were used to generate volcano plot and heatmaps, respectively.

### Transmission electron microscopy

Transmission electron microscopy was carried out as explained previously [55]. Briefly, cells rinsed with 0.1 M phosphate buffer (pH 7.2) were harvested and the pellet formed was instantaneously fixed using Karnovsky’s solution (2% paraformaldehyde and 2.5% glutaraldehyde in 0.1 M phosphate buffer at pH 7.2) at room temperature for 3 h. The cells were then washed with phosphate buffer, post-fixed with 1% osmium tetroxide (for 1 h), and rinsed with distilled water. The water content was then removed in a series of ethanol concentrations from 30% to 95% to 100% and in propylene oxide at the end. After infiltration, embedding, and polymerizing (at 65 °C for 24 h), ultrathin sections with golden color (95 nm) were prepared with a microtome (Leica, Ultracuts EM UC7, Germany). The sections were then collected on 200 mesh Cu grids and double stained with uranyl acetate and lead citrate. The prepared sections were then recorded under the FEI Tecnai Spirit Bio-twin G2 transmission electron microscope.

### Tumor xenograft establishment

All the animal experiments were performed at Vivo Bio Tech Ltd, (Telangana, India) in accordance with approved protocols by the Institute of Animal Ethical Committee of Vivo Bio Tech Ltd (Approval No. 1117/PO/RcBiBt/S/07/CPCSEA). Five-week-old NCr nude mice with 18–22 g body weights were obtained from Vivo Bio Non-Clinical Research Services (Vivo Bio Tech Ltd). The NCr nude mice were accommodated in the specified-pathogen-free animal laboratory. Mice had access to sterilized food and water properly. Mice were inoculated with HT-29 cells subcutaneously (resuspended in DMEM/Matrigel 1:1 v/v) into the right flanks. The mice were examined every day. Animals were randomized one day before the first dosing based on tumor volume. When the volume of the tumors was ~80 mm^3^, SNG (6 mg/kg) every 3 days or an equal volume of vehicle was administered intraperitoneally for 24 days as a treatment course. Tumor volume and body weight were measured every day after grouping. 27 days after SNG treatment, the mice were euthanized, the tumor was collected, and western blot analysis was carried out using representative tumor tissues from each group. Tumor, kidney, and liver tissues were fixed for 7 days in paraformaldehyde, and paraffin-embedded tissues were sectioned and further analyzed by hematoxylin and eosin (H&E) staining.

### Statistical analysis

Statistical analysis was performed by Graph Pad Prism 9.0 software. All data were expressed as mean ± standard deviation (*n* = 3). The significance of the difference between the two groups was determined by Student- *t* test. ANOVA using Bonferroni post-hoc test analysis was used to compare the differences for multiple comparisons. Asterisk (*) represents *p* value < 0.05, double-asterisk (**) represents *p* value < 0.01, and triple-asterisk (***) represents *p* value < 0.001. A *p* value below 0.05 was considered to be statistically significant.

## Supplementary information

Supplementary Figures 1-6

Supplemental Table S1

Supplemental Table S2

Supplemental Table S3

Original Western Blots

## Data Availability

The authors declare that the data supporting the outcomes of this study are available in the article and its supplementary files. The source data are available from the corresponding author upon reasonable request.

## References

[1] Green DR (2019). The coming decade of cell death research: five riddles. Cell.

[2] Ashkenazi A, Salvesen G (2014). Regulated cell death: signaling and mechanisms. Annu Rev Cell Dev Biol.

[3] Galluzzi L, Vitale I, Aaronson SA, Abrams JM, Adam D, Agostinis P (2018). Molecular mechanisms of cell death: recommendations of the Nomenclature Committee on Cell Death 2018. Cell Death Differ.

[4] Tang D, Kang R, Berghe TV, Vandenabeele P, Kroemer G (2019). The molecular machinery of regulated cell death. Cell Res.

[5] Holze C, Michaudel C, Mackowiak C, Haas DA, Benda C, Hubel P (2018). Oxeiptosis, a ROS-induced caspase-independent apoptosis-like cell-death pathway. Nat Immunol.

[6] Deshmukh P, Unni S, Krishnappa G, Padmanabhan B (2017). The Keap1-Nrf2 pathway: promising therapeutic target to counteract ROS-mediated damage in cancers and neurodegenerative diseases. Biophys Rev.

[7] Fourquet S, Guerois R, Biard D, Toledano MB (2010). Activation of NRF2 by nitrosative agents and H2O2 involves KEAP1 disulfide formation. J Biol Chem.

[8] Yamamoto T, Suzuki T, Kobayashi A, Wakabayashi J, Maher J, Motohashi H (2008). Physiological significance of reactive cysteine residues of Keap1 in determining Nrf2 activity. Mol Cell Biol.

[9] Scaturro P, Pichlmair A (2018). Oxeiptosis-a cell death pathway to mitigate damage caused by radicals. Cell Death Differ.

[10] Scaturro P, Pichlmair A (2019). Oxeiptosis: a discreet way to respond to radicals. Curr Opin Immunol.

[11] Galadari S, Rahman A, Pallichankandy S, Thayyullathil F (2017). Reactive oxygen species and cancer paradox: To promote or to suppress?. Free Radic Biol Med.

[12] Saito Y, Nishio K, Ogawa Y, Kimata J, Kinumi T, Yoshida Y (2006). Turning point in apoptosis/necrosis induced by hydrogen peroxide. Free Radic Res.

[13] Ingold I, Berndt C, Schmitt S, Doll S, Poschmann G, Buday K (2018). Selenium utilization by GPX4 is required to prevent hydroperoxide-induced ferroptosis. Cell.

[14] Pallichankandy S, Rahman A, Thayyullathil F, Galadari S (2015). ROS-dependent activation of autophagy is a critical mechanism for the induction of anti-glioma effect of sanguinarine. Free Radic Biol Med.

[15] Sokolowska M, Quesniaux VFJ, Akdis CA, Chung KF, Ryffel B, Togbe D (2019). Acute respiratory barrier disruption by ozone exposure in mice. Front Immunol.

[16] Dabaghi M, Quaas R, Hilger I. The treatment of heterotopic human colon xenograft tumors in mice with 5-fluorouracil attached to magnetic nanoparticles in combination with magnetic hyperthermia is more efficient than either therapy alone. Cancers. 2020;12. 10.3390/cancers12092562. 10.3390/cancers12092562 PMC7566013 32916798

[17] Hamaidia M, Gazon H, Hoyos C, Hoffmann GB, Louis R, Duysinx B, et al. Inhibition of EZH2 methyltransferase decreases immunoediting of mesothelioma cells by autologous macrophages through a PD-1-dependent mechanism. JCI Insight. 2019;4. 10.1172/jci.insight.128474. 10.1172/jci.insight.128474 PMC6795292 31534051

[18] Mauri G, Sartore-Bianchi A, Russo AG, Marsoni S, Bardelli A, Siena S (2019). Early-onset colorectal cancer in young individuals. Mol Oncol.

[19] Fichtner M, Bozkurt E, Salvucci M, McCann C, McAllister KA, Halang L (2020). Molecular subtype-specific responses of colon cancer cells to the SMAC mimetic Birinapant. Cell Death Dis.

[20] Choudhari AS, Mandave PC, Deshpande M, Ranjekar P, Prakash O (2019). Phytochemicals in cancer treatment: from preclinical studies to clinical practice. Front Pharmacol.

[21] Hosseinzadeh E, Hassanzadeh A, Marofi F, Alivand MR, Solali S (2020). Flavonoid-based cancer therapy: an updated review. Anticancer Agents Med Chem.

[22] Manogaran P, Umapathy D, Karthikeyan M, Venkatachalam K, Singaravelu A (2021). Dietary phytochemicals as a potential source for targeting cancer stem cells. Cancer Invest.

[23] Zhang Q, Lyu Y, Huang J, Zhang X, Yu N, Wen Z (2020). Antibacterial activity and mechanism of sanguinarine against Providencia rettgeri in vitro. PeerJ.

[24] Yang XJ, Miao F, Yao Y, Cao FJ, Yang R, Ma YN (2012). In vitro antifungal activity of sanguinarine and chelerythrine derivatives against phytopathogenic fungi. Molecules.

[25] Galadari S, Rahman A, Pallichankandy S, Thayyullathil F (2017). Molecular targets and anticancer potential of sanguinarine-a benzophenanthridine alkaloid. Phytomedicine.

[26] Rahman A, Thayyullathil F, Pallichankandy S, Galadari S (2016). Hydrogen peroxide/ceramide/Akt signaling axis play a critical role in the antileukemic potential of sanguinarine. Free Radic Biol Med.

[27] Rahman A, Pallichankandy S, Thayyullathil F, Galadari S (2019). Critical role of H_2_O_2_ in mediating sanguinarine-induced apoptosis in prostate cancer cells via facilitating ceramide generation, ERK1/2 phosphorylation, and Par-4 cleavage. Free Radic Biol Med.

[28] Su Q, Fan M, Wang J, Ullah A, Ghauri MA, Dai B (2019). Sanguinarine inhibits epithelial-mesenchymal transition via targeting HIF-1alpha/TGF-beta feed-forward loop in hepatocellular carcinoma. Cell Death Dis.

[29] Denton D, Kumar S (2019). Autophagy-dependent cell death. Cell Death Differ.

[30] Mizushima N, Yoshimori T, Levine B (2010). Methods in mammalian autophagy research. Cell.

[31] Gonzalez-Amor M, Garcia-Redondo AB, Jorge I, Zalba G, Becares M, Ruiz-Rodriguez MJ, et al. Interferon stimulated gene 15 pathway is a novel mediator of endothelial dysfunction and aneurysms development in angiotensin II infused mice through increased oxidative stress. Cardiovasc Res. 2021. 10.1093/cvr/cvab321. 10.1093/cvr/cvab321 PMC9799052 34672341

[32] Dastur A, Beaudenon S, Kelley M, Krug RM, Huibregtse JM (2006). Herc5, an interferon-induced HECT E3 enzyme, is required for conjugation of ISG15 in human cells. J Biol Chem.

[33] Desagher S, Glowinski J, Premont J (1997). Pyruvate protects neurons against hydrogen peroxide-induced toxicity. J Neurosci.

[34] Dickinson BC, Lin VS, Chang CJ (2013). Preparation and use of MitoPY1 for imaging hydrogen peroxide in mitochondria of live cells. Nat Protoc.

[35] Lo SC, Hannink M (2006). PGAM5, a Bcl-XL-interacting protein, is a novel substrate for the redox-regulated Keap1-dependent ubiquitin ligase complex. J Biol Chem.

[36] Redza-Dutordoir M, Averill-Bates DA (2016). Activation of apoptosis signalling pathways by reactive oxygen species. Biochim Biophys Acta.

[37] Christofferson DE, Yuan J (2010). Necroptosis as an alternative form of programmed cell death. Curr Opin Cell Biol.

[38] Jacobson MD, Raff MC (1995). Programmed cell death and Bcl-2 protection in very low oxygen. Nature.

[39] Lee HY, Itahana Y, Schuechner S, Fukuda M, Je HS, Ogris E, et al. Ca(2+)-dependent demethylation of phosphatase PP2Ac promotes glucose deprivation-induced cell death independently of inhibiting glycolysis. Sci Signal. 2018;11. 10.1126/scisignal.aam7893. 10.1126/scisignal.aam7893 29317521

[40] Watson AJ (2004). Apoptosis and colorectal cancer. Gut.

[41] Zhang B, Wang X, Deng J, Zheng H, Liu W, Chen S (2019). p53-dependent upregulation of miR-16-2 by sanguinarine induces cell cycle arrest and apoptosis in hepatocellular carcinoma. Cancer Lett.

[42] Fu C, Guan G, Wang H (2018). The anticancer effect of sanguinarine: a review. Curr Pharm Des.

[43] Choi WY, Jin CY, Han MH, Kim GY, Kim ND, Lee WH (2009). Sanguinarine sensitizes human gastric adenocarcinoma AGS cells to TRAIL-mediated apoptosis via down-regulation of AKT and activation of caspase-3. Anticancer Res.

[44] Thayyullathil F, Chathoth S, Hago A, Patel M, Galadari S (2008). Rapid reactive oxygen species (ROS) generation induced by curcumin leads to caspase-dependent and -independent apoptosis in L929 cells. Free Radic Biol Med.

[45] Anders S, Pyl PT, Huber W (2015). HTSeq-a Python framework to work with high-throughput sequencing data. Bioinformatics.

[46] Bolger AM, Lohse M, Usadel B (2014). Trimmomatic: a flexible trimmer for Illumina sequence data. Bioinformatics.

[47] Chen S, Zhou Y, Chen Y, Gu J (2018). fastp: an ultra-fast all-in-one FASTQ preprocessor. Bioinformatics.

[48] Kim D, Langmead B, Salzberg SL (2015). HISAT: a fast spliced aligner with low memory requirements. Nat Methods.

[49] Li H, Handsaker B, Wysoker A, Fennell T, Ruan J, Homer N (2009). The sequence alignment/map format and SAMtools. Bioinformatics.

[50] Pertea M, Kim D, Pertea GM, Leek JT, Salzberg SL (2016). Transcript-level expression analysis of RNA-seq experiments with HISAT, StringTie and Ballgown. Nat Protoc.

[51] Garcia-Alcalde F, Okonechnikov K, Carbonell J, Cruz LM, Gotz S, Tarazona S (2012). Qualimap: evaluating next-generation sequencing alignment data. Bioinformatics.

[52] Yousif A, Drou N, Rowe J, Khalfan M, Gunsalus KC (2020). NASQAR: a web-based platform for high-throughput sequencing data analysis and visualization. BMC Bioinform.

[53] Huang Y, Wang JY, Wei XM, Hu B (2014). Bioinfo-Kit: a sharing software tool for bioinformatics. AppL Mech Mater.

[54] Kolde R, Maintainer Raivo Kolde. “Package ‘pheatmap’.” R package. 2015;1:790.

[55] Thayyullathil F, Cheratta AR, Pallichankandy S, Subburayan K, Tariq S, Rangnekar VM (2020). Par-4 regulates autophagic cell death in human cancer cells via upregulating p53 and BNIP3. Biochim Biophys Acta Mol Cell Res.

