# Supplementary material for: Targeting oxeiptosis-mediated tumor suppression: a novel approach to treat colorectal cancers by sanguinarine
Source: Cell Death Discov. 2023 Mar 13;9:94. doi: 10.1038/s41420-023-01376-3 (PMC10011521; doi:10.1038/s41420-023-01376-3)
Supplement: Supplementary file 1 — Supplementary Figures 1-6 [file 41420_2023_1376_MOESM1_ESM.docx]

**Supplemental Figures**

**
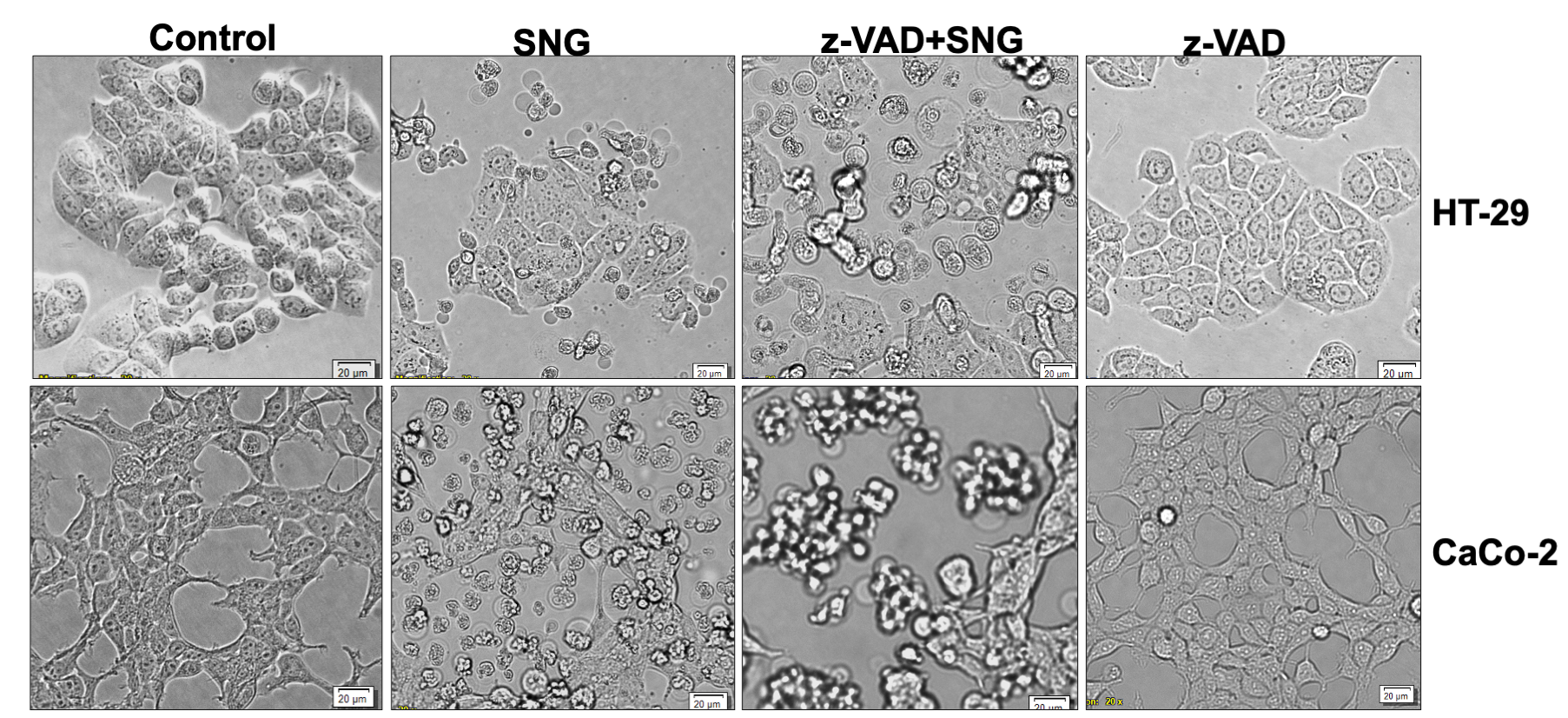
**

**Supplementary figure 1.**  CRC cells were pretreated with z-VAD for 1 h followed by SNG treatment for further 16 h. Morphological changes were examined by using phase contrast inverted microscope.

**A B**


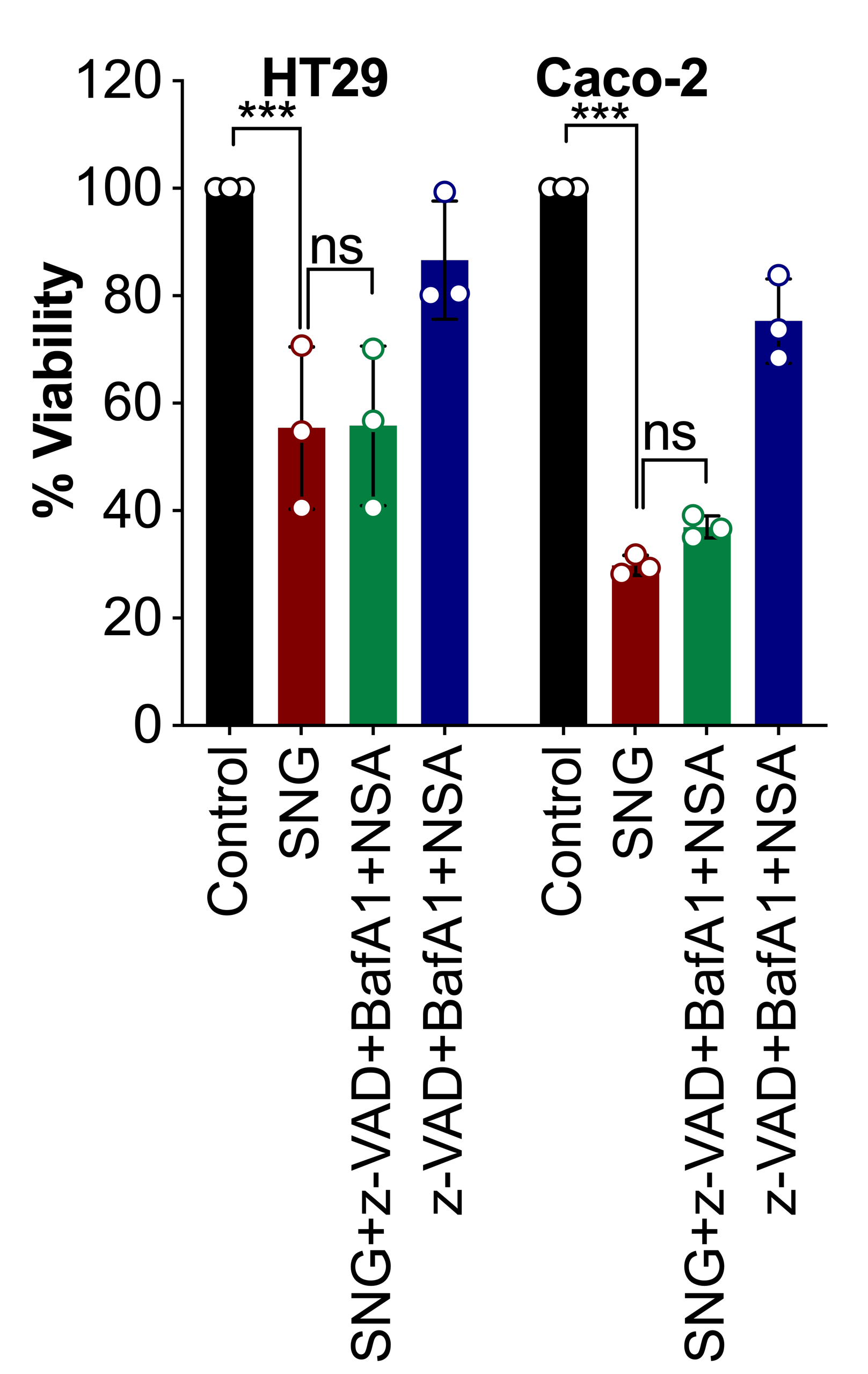


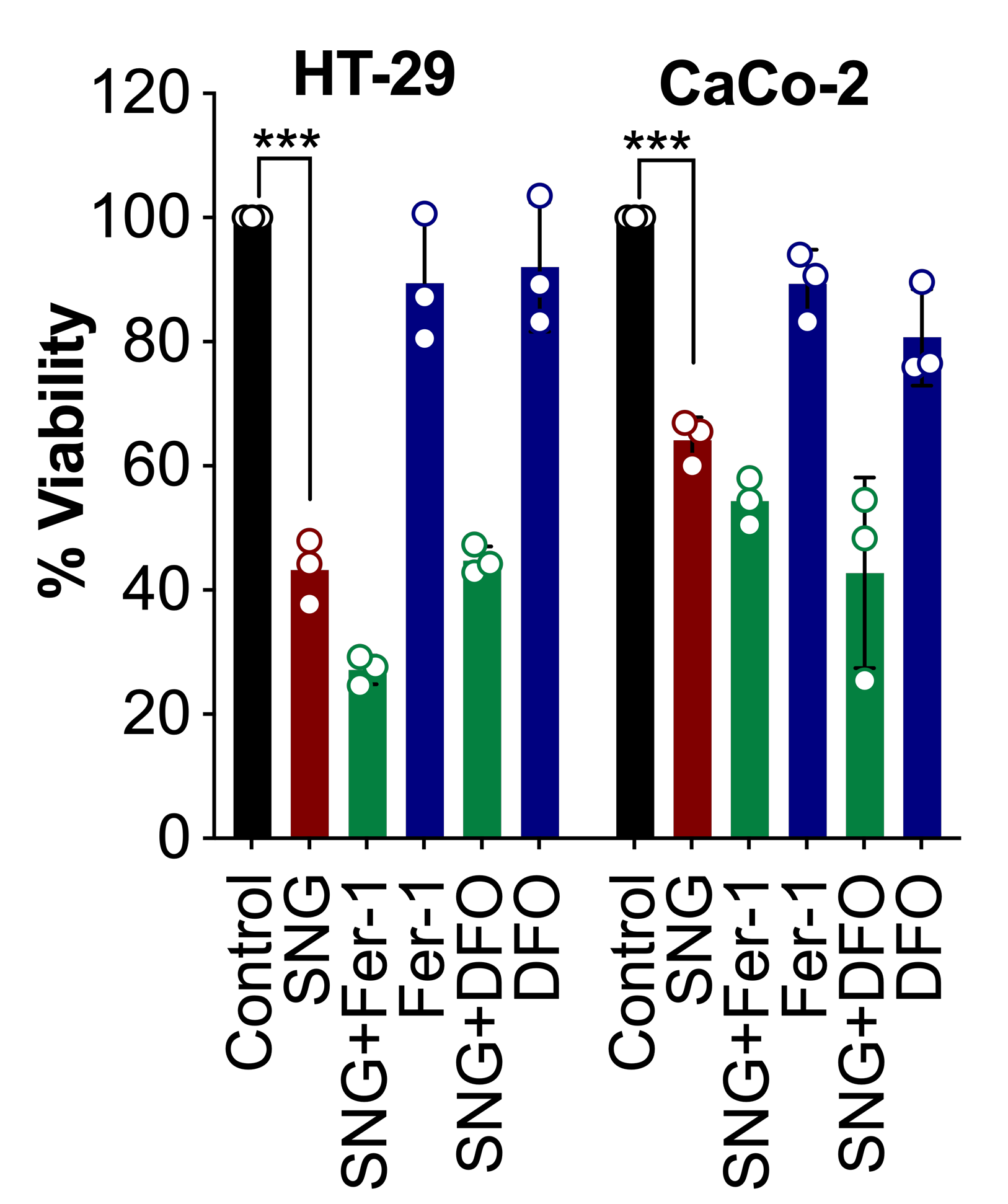


**Supplementary figure 2. (A),** CRC cells were pre-treated with Fer-1 and DFO for 1h followed by treatment with SNG. After the treatment cell viability was measured by using MTT assay. (**B),** CRC cells were pre-treated with combination of z-VAD, Baf A1 and NSA for 1 h followed by treatment with SNG for 16 h. After the treatment cell viability was measured by using MTT assay. Data shown are means ± SD (*n*=3) (****p*<0.001 and ns= no significance).


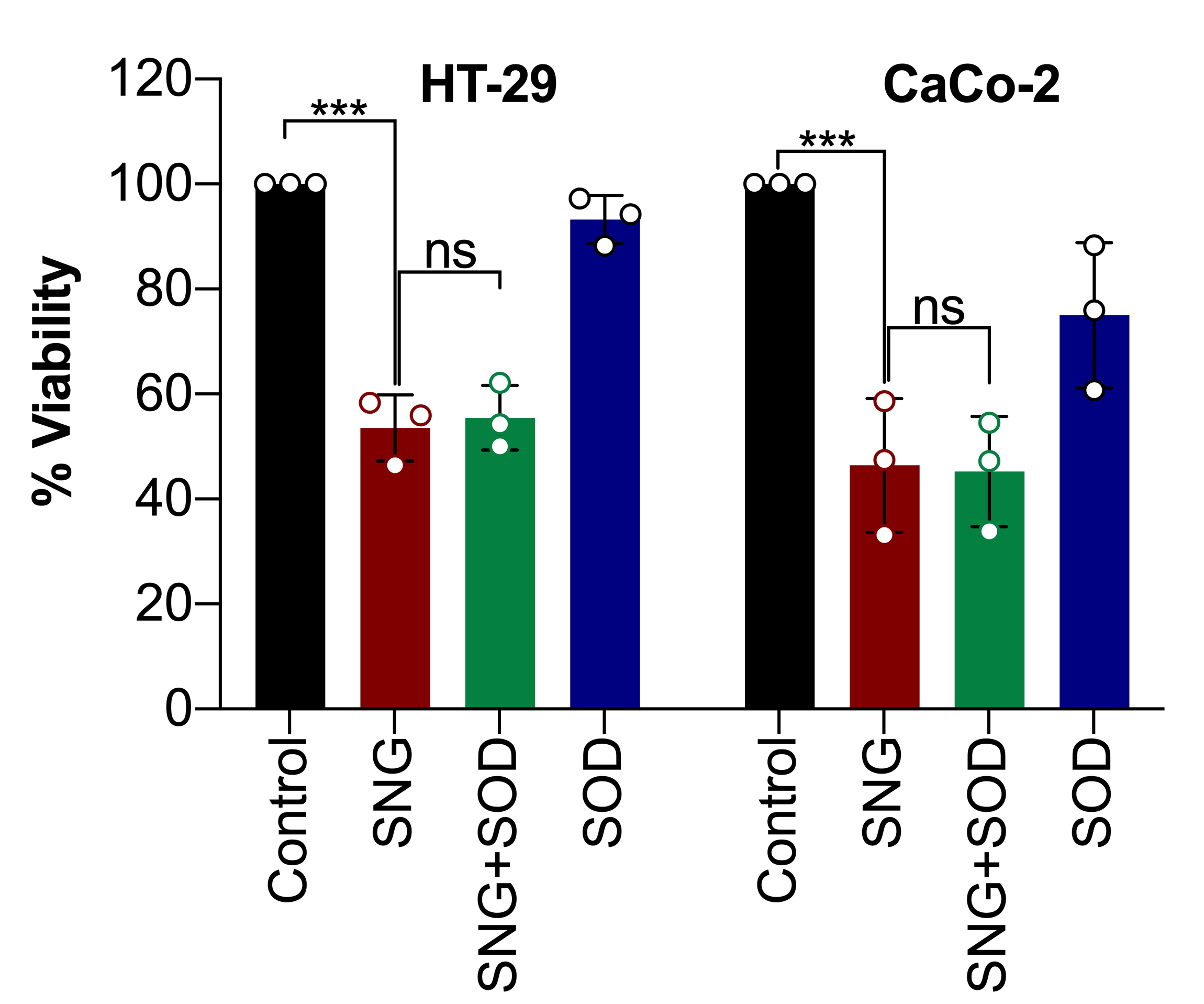


**Supplementary figure 3.** CRC cells were pre-treated with combination of SOD for 1h followed by treatment with SNG. After the treatment cell viability was measured by using MTT assay. Data shown are means ± SD (*n*=3) (****p*<0.001 and ns= no significance).


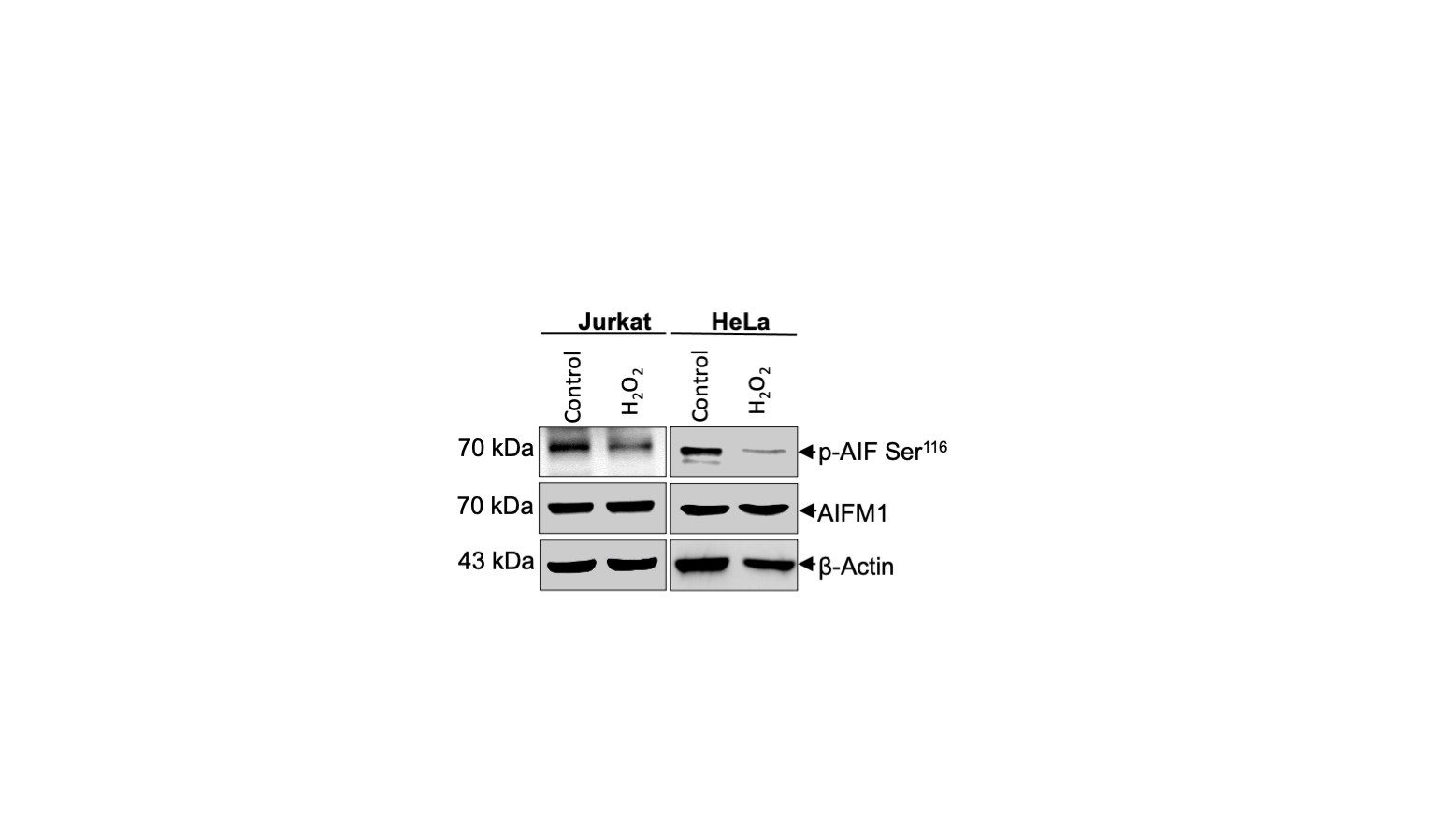


**Supplementary figure 4.** Cells were treated with H_2_O_2_ (0.5mM for Jurkat and 2mM for HeLa) for 4 h and 24 h respectively. Following the treatment, cells were lysed and western blot assay for indicated proteins were performed.


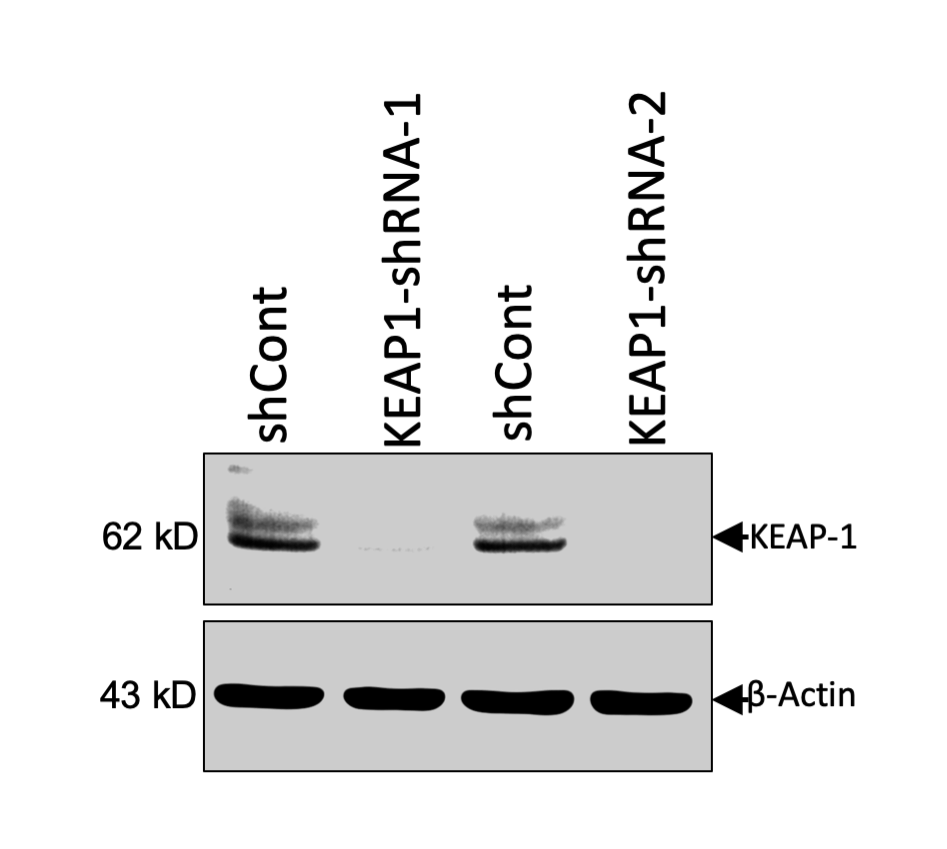


**Supplementary figure 5.** KEAP1 protein was depleted in HT-29 cells using lentiviral vector expressing shRNAs.


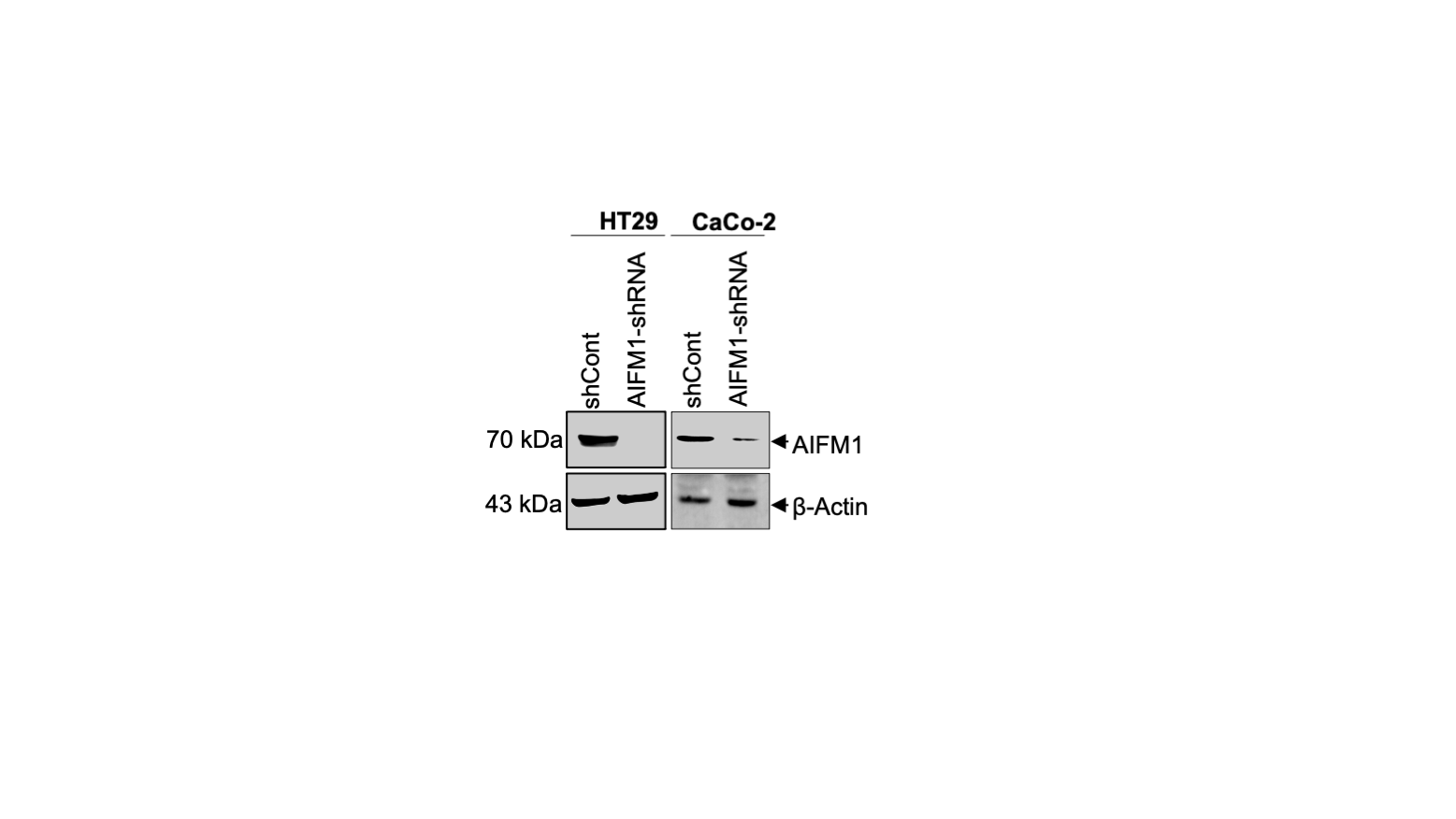


**Supplementary figure 6** AIFM1 protein was depleted using lentiviral vectors expressing shRNAs.
