# Supplementary material for: Targeting oxeiptosis-mediated tumor suppression: a novel approach to treat colorectal cancers by sanguinarine
Source: Cell Death Discov. 2023 Mar 13;9:94. doi: 10.1038/s41420-023-01376-3 (PMC10011521; doi:10.1038/s41420-023-01376-3)
Supplement: Supplementary file 2 — Supplemental Table S1 [file 41420_2023_1376_MOESM2_ESM.docx]

| **Supplemental Table S1.** | | | |  |  |  |
| --- | --- | --- | --- | --- | --- | --- |
|  |  |  |  |  |  |  |
| **List of differential expression between control and SNG treated groups (p value＜0.05)** | | | | | | |
|  |  |  |  |  |  |  |

**List of upregulated genes (p value＜0.05)**

| Gene ID | Gene symbol | log2FoldChange | padj |
| --- | --- | --- | --- |
| ENSG00000169813 | HNRNPF | 0.190505259 | 0.038816385 |
| ENSG00000147140 | NONO | 0.192827339 | 0.036348131 |
| ENSG00000025796 | SEC63 | 0.204769413 | 0.042539888 |
| ENSG00000003147 | ICA1 | 0.206716984 | 0.036213434 |
| ENSG00000198087 | CD2AP | 0.207095651 | 0.03883486 |
| ENSG00000102996 | MMP15 | 0.207543563 | 0.018975981 |
| ENSG00000138758 | SEPT11. | 0.208631503 | 0.036727831 |
| ENSG00000180370 | PAK2 | 0.209314331 | 0.045894997 |
| ENSG00000197324 | LRP10 | 0.211408221 | 0.022175918 |
| ENSG00000072803 | FBXW11 | 0.214340022 | 0.027954432 |
| ENSG00000151239 | TWF1 | 0.21440761 | 0.048276761 |
| ENSG00000133703 | KRAS | 0.219481106 | 0.015377866 |
| ENSG00000156642 | NPTN | 0.219859228 | 0.040114442 |
| ENSG00000141198 | TOM1L1 | 0.21989336 | 0.037142401 |
| ENSG00000078061 | ARAF | 0.219910088 | 0.03718663 |
| ENSG00000110321 | EIF4G2 | 0.221462406 | 0.024089463 |
| ENSG00000136754 | ABI1 | 0.22602437 | 0.029732152 |
| ENSG00000089009 | RPL6 | 0.229121354 | 0.036067572 |
| ENSG00000087152 | ATXN7L3 | 0.230373724 | 0.01134221 |
| ENSG00000130985 | UBA1 | 0.230645663 | 0.017992071 |
| ENSG00000167193 | CRK | 0.232160824 | 0.01371152 |
| ENSG00000129925 | TMEM8A | 0.232233172 | 0.029457946 |
| ENSG00000111843 | TMEM14C | 0.233255008 | 0.044968976 |
| ENSG00000264364 | DYNLL2 | 0.234747216 | 0.02922256 |
| ENSG00000176853 | FAM91A1 | 0.237221799 | 0.004656331 |
| ENSG00000131653 | TRAF7 | 0.239075189 | 0.038252171 |
| ENSG00000048828 | FAM120A | 0.2392567 | 0.014291614 |
| ENSG00000132676 | DAP3 | 0.240732694 | 0.019964363 |
| ENSG00000213024 | NUP62 | 0.241032989 | 0.039553939 |
| ENSG00000165527 | ARF6 | 0.241230253 | 0.028976261 |
| ENSG00000126822 | PLEKHG3 | 0.242350848 | 0.022922078 |
| ENSG00000109184 | DCUN1D4 | 0.242365422 | 0.031830873 |
| ENSG00000002834 | LASP1 | 0.242590936 | 0.011273541 |
| ENSG00000091039 | OSBPL8 | 0.243361878 | 0.037331893 |
| ENSG00000105701 | FKBP8 | 0.244190731 | 0.008894725 |
| ENSG00000101558 | VAPA | 0.244344541 | 0.009741838 |
| ENSG00000119446 | RBM18 | 0.244731067 | 0.031264814 |
| ENSG00000149658 | YTHDF1 | 0.245008706 | 0.036368772 |
| ENSG00000112062 | MAPK14 | 0.245177078 | 0.047475172 |
| ENSG00000064666 | CNN2 | 0.245352679 | 0.047938829 |
| ENSG00000075415 | SLC25A3 | 0.245755986 | 0.01529429 |
| ENSG00000100220 | RTCB | 0.245920229 | 0.046061562 |
| ENSG00000071127 | WDR1 | 0.246377054 | 0.016244761 |
| ENSG00000169057 | MECP2 | 0.246405827 | 0.036460045 |
| ENSG00000126247 | CAPNS1 | 0.246485721 | 0.011674006 |
| ENSG00000170759 | KIF5B | 0.247596004 | 0.014158672 |
| ENSG00000064726 | BTBD1 | 0.250246961 | 0.021137239 |
| ENSG00000171862 | PTEN | 0.250816445 | 0.006147383 |
| ENSG00000132698 | RAB25 | 0.251872218 | 0.018162653 |
| ENSG00000090054 | SPTLC1 | 0.251986743 | 0.029432662 |
| ENSG00000158985 | CDC42SE2 | 0.252990895 | 0.005542859 |
| ENSG00000205937 | RNPS1 | 0.254303367 | 0.018606409 |
| ENSG00000126457 | PRMT1 | 0.254601525 | 0.035504785 |
| ENSG00000167770 | OTUB1 | 0.255403256 | 0.010471965 |
| ENSG00000139132 | FGD4 | 0.255470679 | 0.035375466 |
| ENSG00000121022 | COPS5 | 0.256186101 | 0.04432572 |
| ENSG00000196422 | PPP1R26 | 0.25622624 | 0.027515114 |
| ENSG00000100129 | EIF3L | 0.256708685 | 0.008606889 |
| ENSG00000127328 | RAB3IP | 0.257507295 | 0.01352906 |
| ENSG00000135624 | CCT7 | 0.257890994 | 0.025869564 |
| ENSG00000002549 | LAP3 | 0.258917495 | 0.005924614 |
| ENSG00000112378 | PERP | 0.259238058 | 0.012954019 |
| ENSG00000105568 | PPP2R1A | 0.260573058 | 0.015998627 |
| ENSG00000065882 | TBC1D1 | 0.26178564 | 0.015201806 |
| ENSG00000185728 | YTHDF3 | 0.262142324 | 0.02428795 |
| ENSG00000132155 | RAF1 | 0.262824994 | 0.042472261 |
| ENSG00000143612 | C1orf43 | 0.263203328 | 0.021025543 |
| ENSG00000113384 | GOLPH3 | 0.263306689 | 0.016203801 |
| ENSG00000128309 | MPST | 0.263587473 | 0.046342427 |
| ENSG00000135506 | OS9 | 0.264096339 | 0.011469395 |
| ENSG00000160218 | TRAPPC10 | 0.264139576 | 0.031272952 |
| ENSG00000113924 | HGD | 0.2645035 | 0.039905045 |
| ENSG00000088832 | FKBP1A | 0.264615312 | 0.01828058 |
| ENSG00000185122 | HSF1 | 0.265419354 | 0.034401498 |
| ENSG00000010244 | ZNF207 | 0.266368982 | 0.035169255 |
| ENSG00000125991 | ERGIC3 | 0.266681593 | 0.007191123 |
| ENSG00000072274 | TFRC | 0.266995164 | 0.013504764 |
| ENSG00000159592 | GPBP1L1 | 0.26719359 | 0.007358734 |
| ENSG00000116209 | TMEM59 | 0.267474702 | 0.013062965 |
| ENSG00000164023 | SGMS2 | 0.267862915 | 0.03116888 |
| ENSG00000182220 | ATP6AP2 | 0.269300565 | 0.030905612 |
| ENSG00000165119 | HNRNPK | 0.269315962 | 0.007970818 |
| ENSG00000090266 | NDUFB2 | 0.269448883 | 0.047367503 |
| ENSG00000166226 | CCT2 | 0.269536255 | 0.038645875 |
| ENSG00000087191 | PSMC5 | 0.269892126 | 0.018408211 |
| ENSG00000163655 | GMPS | 0.270498867 | 0.023581115 |
| ENSG00000155463 | OXA1L | 0.271071382 | 0.024746266 |
| ENSG00000104331 | IMPAD1 | 0.271075003 | 0.019482277 |
| ENSG00000111321 | LTBR | 0.271505175 | 0.008290205 |
| ENSG00000164597 | COG5 | 0.272064396 | 0.007008253 |
| ENSG00000149657 | LSM14B | 0.272387699 | 0.039008657 |
| ENSG00000146833 | TRIM4 | 0.272623594 | 0.026370872 |
| ENSG00000185238 | PRMT3 | 0.272733402 | 0.049561822 |
| ENSG00000188706 | ZDHHC9 | 0.272858742 | 0.014257289 |
| ENSG00000149480 | MTA2 | 0.272949185 | 0.022098924 |
| ENSG00000059122 | FLYWCH1 | 0.272958579 | 0.042739533 |
| ENSG00000110713 | NUP98 | 0.27341936 | 0.009872361 |
| ENSG00000175387 | SMAD2 | 0.274609623 | 0.011439497 |
| ENSG00000171490 | RSL1D1 | 0.274756984 | 0.032827062 |
| ENSG00000154001 | PPP2R5E | 0.275030261 | 0.017833799 |
| ENSG00000078403 | MLLT10 | 0.275215703 | 0.037499821 |
| ENSG00000173511 | VEGFB | 0.275885046 | 0.035697033 |
| ENSG00000072415 | MPP5 | 0.276073272 | 0.031235475 |
| ENSG00000051523 | CYBA | 0.276208196 | 0.012915551 |
| ENSG00000077549 | CAPZB | 0.276273415 | 0.004745946 |
| ENSG00000197170 | PSMD12 | 0.277043427 | 0.010549325 |
| ENSG00000164300 | SERINC5 | 0.277933439 | 0.013371551 |
| ENSG00000076984 | MAP2K7 | 0.27802932 | 0.036371409 |
| ENSG00000154144 | TBRG1 | 0.278500894 | 0.031191431 |
| ENSG00000164164 | OTUD4 | 0.278509523 | 0.031116619 |
| ENSG00000101294 | HM13 | 0.279103747 | 0.026824463 |
| ENSG00000152620 | NADK2 | 0.279363211 | 0.034473091 |
| ENSG00000165688 | PMPCA | 0.279374747 | 0.00882023 |
| ENSG00000165156 | ZHX1 | 0.279633753 | 0.006359682 |
| ENSG00000110422 | HIPK3 | 0.279832438 | 0.016707568 |
| ENSG00000067955 | CBFB | 0.280258434 | 0.031272407 |
| ENSG00000179195 | ZNF664 | 0.280444745 | 0.006960696 |
| ENSG00000255112 | CHMP1B | 0.281135367 | 0.024843643 |
| ENSG00000114544 | SLC41A3 | 0.281489388 | 0.018481153 |
| ENSG00000156515 | HK1 | 0.282628978 | 0.00500758 |
| ENSG00000079313 | REXO1 | 0.282780023 | 0.016635165 |
| ENSG00000122203 | KIAA1191 | 0.282962286 | 0.014622383 |
| ENSG00000114650 | SCAP | 0.283243404 | 0.005056675 |
| ENSG00000186660 | ZFP91 | 0.283253959 | 0.002977682 |
| ENSG00000166024 | R3HCC1L | 0.283980065 | 0.037361389 |
| ENSG00000176978 | DPP7 | 0.284019553 | 0.011858038 |
| ENSG00000153922 | CHD1 | 0.285055355 | 0.035147398 |
| ENSG00000106628 | POLD2 | 0.285773035 | 0.038028063 |
| ENSG00000102898 | NUTF2 | 0.28820918 | 0.046597362 |
| ENSG00000091009 | RBM27 | 0.290147864 | 0.036882364 |
| ENSG00000128534 | LSM8 | 0.290205338 | 0.048504935 |
| ENSG00000138386 | NAB1 | 0.29037299 | 0.01520023 |
| ENSG00000109458 | GAB1 | 0.290834549 | 0.017940695 |
| ENSG00000182107 | TMEM30B | 0.290888224 | 0.014950052 |
| ENSG00000133961 | NUMB | 0.291119716 | 0.002208915 |
| ENSG00000091164 | TXNL1 | 0.291661701 | 0.004807361 |
| ENSG00000080031 | PTPRH | 0.292607555 | 0.030905612 |
| ENSG00000177105 | RHOG | 0.292643265 | 0.043358979 |
| ENSG00000080845 | DLGAP4 | 0.292883459 | 0.013145662 |
| ENSG00000065150 | IPO5 | 0.293152595 | 0.025638026 |
| ENSG00000115241 | PPM1G | 0.293325313 | 0.008378981 |
| ENSG00000137692 | DCUN1D5 | 0.293836696 | 0.025729729 |
| ENSG00000105379 | ETFB | 0.29440599 | 0.012107965 |
| ENSG00000135655 | USP15 | 0.294413752 | 0.004057347 |
| ENSG00000198026 | ZNF335 | 0.295011767 | 0.030990666 |
| ENSG00000171314 | PGAM1 | 0.295085323 | 0.024488602 |
| ENSG00000105127 | AKAP8 | 0.295676573 | 0.032625995 |
| ENSG00000148426 | PROSER2 | 0.295882536 | 0.045651999 |
| ENSG00000126254 | RBM42 | 0.295962882 | 0.014447026 |
| ENSG00000090889 | KIF4A | 0.296064862 | 0.03835653 |
| ENSG00000159210 | SNF8 | 0.296192079 | 0.025953148 |
| ENSG00000170638 | TRABD | 0.296314911 | 0.018942735 |
| ENSG00000136144 | RCBTB1 | 0.296626236 | 0.032752333 |
| ENSG00000186416 | NKRF | 0.29691744 | 0.032817564 |
| ENSG00000105402 | NAPA | 0.297647423 | 0.025812941 |
| ENSG00000134882 | UBAC2 | 0.29794331 | 0.029098353 |
| ENSG00000149115 | TNKS1BP1 | 0.298001328 | 0.047394869 |
| ENSG00000104881 | PPP1R13L | 0.29831929 | 0.016062449 |
| ENSG00000067334 | DNTTIP2 | 0.298363504 | 0.013452679 |
| ENSG00000022277 | RTF2 | 0.298401398 | 0.008810934 |
| ENSG00000153113 | CAST | 0.298526682 | 0.002749332 |
| ENSG00000092010 | PSME1 | 0.298793578 | 0.010569072 |
| ENSG00000125977 | EIF2S2 | 0.299258215 | 0.003190407 |
| ENSG00000177051 | FBXO46 | 0.299331631 | 0.008106134 |
| ENSG00000175756 | AURKAIP1 | 0.300181309 | 0.043137037 |
| ENSG00000089154 | GCN1 | 0.300540769 | 0.02191767 |
| ENSG00000170234 | PWWP2A | 0.300637407 | 0.011767999 |
| ENSG00000101577 | LPIN2 | 0.300743591 | 0.012972912 |
| ENSG00000136527 | TRA2B | 0.301156663 | 0.001525786 |
| ENSG00000145912 | NHP2 | 0.301453818 | 0.034365321 |
| ENSG00000131408 | NR1H2 | 0.301470302 | 0.012187861 |
| ENSG00000101457 | DNTTIP1 | 0.302159542 | 0.043081818 |
| ENSG00000198887 | SMC5 | 0.302645396 | 0.040134292 |
| ENSG00000136717 | BIN1 | 0.303681747 | 0.008651854 |
| ENSG00000121864 | ZNF639 | 0.303763262 | 0.03276124 |
| ENSG00000156671 | SAMD8 | 0.303772695 | 0.013041633 |
| ENSG00000135916 | ITM2C | 0.303881834 | 0.010752786 |
| ENSG00000084463 | WBP11 | 0.30415622 | 0.04432572 |
| ENSG00000099968 | BCL2L13 | 0.304241569 | 0.007018043 |
| ENSG00000119760 | SUPT7L | 0.304446777 | 0.02296761 |
| ENSG00000167182 | SP2 | 0.304524309 | 0.010482913 |
| ENSG00000170027 | YWHAG | 0.304602564 | 0.007505361 |
| ENSG00000095139 | ARCN1 | 0.30498353 | 0.012352726 |
| ENSG00000214655 | ZSWIM8 | 0.30520971 | 0.0484051 |
| ENSG00000198815 | FOXJ3 | 0.305209825 | 0.008511975 |
| ENSG00000185596 | WASH3P | 0.306069867 | 0.017393033 |
| ENSG00000166140 | ZFYVE19 | 0.306925605 | 0.021608686 |
| ENSG00000126934 | MAP2K2 | 0.308779909 | 0.001868397 |
| ENSG00000247077 | PGAM5 | 0.308966918 | 0.022134575 |
| ENSG00000164609 | SLU7 | 0.30902118 | 0.009657024 |
| ENSG00000196262 | PPIA | 0.309722491 | 0.025960262 |
| ENSG00000063978 | RNF4 | 0.311292764 | 0.012107965 |
| ENSG00000109606 | DHX15 | 0.311815623 | 0.004039991 |
| ENSG00000140307 | GTF2A2 | 0.312110067 | 0.019168924 |
| ENSG00000169756 | LIMS1 | 0.312669596 | 0.02367789 |
| ENSG00000181789 | COPG1 | 0.312765923 | 0.015678218 |
| ENSG00000100603 | SNW1 | 0.312929054 | 0.004070699 |
| ENSG00000140391 | TSPAN3 | 0.313137453 | 0.009450042 |
| ENSG00000145390 | USP53 | 0.31328389 | 0.009041149 |
| ENSG00000069345 | DNAJA2 | 0.313416729 | 0.001219048 |
| ENSG00000182004 | SNRPE | 0.313610595 | 0.047943943 |
| ENSG00000116001 | TIA1 | 0.313723182 | 0.008546741 |
| ENSG00000170144 | HNRNPA3 | 0.313932834 | 0.004505212 |
| ENSG00000035403 | VCL | 0.314398946 | 0.027355109 |
| ENSG00000100109 | TFIP11 | 0.314758174 | 0.025974754 |
| ENSG00000126267 | COX6B1 | 0.314846741 | 0.040040126 |
| ENSG00000100258 | LMF2 | 0.314872941 | 0.016119974 |
| ENSG00000111666 | CHPT1 | 0.314967994 | 0.025638026 |
| ENSG00000047849 | MAP4 | 0.3152436 | 0.006012259 |
| ENSG00000176153 | GPX2 | 0.315492908 | 0.008106134 |
| ENSG00000130429 | ARPC1B | 0.315565527 | 0.00702462 |
| ENSG00000233493 | TMEM238 | 0.316143994 | 0.025225748 |
| ENSG00000096401 | CDC5L | 0.316210063 | 0.001314721 |
| ENSG00000101187 | SLCO4A1 | 0.316416262 | 0.018020911 |
| ENSG00000137699 | TRIM29 | 0.316539892 | 0.000860571 |
| ENSG00000150459 | SAP18 | 0.317443929 | 0.012380102 |
| ENSG00000147687 | TATDN1 | 0.31794209 | 0.016333384 |
| ENSG00000176903 | PNMA1 | 0.318264325 | 0.002782155 |
| ENSG00000106546 | AHR | 0.318420947 | 0.003031392 |
| ENSG00000169764 | UGP2 | 0.319358219 | 0.00471286 |
| ENSG00000104774 | MAN2B1 | 0.319858279 | 0.009177842 |
| ENSG00000131269 | ABCB7 | 0.319867234 | 0.004127312 |
| ENSG00000196365 | LONP1 | 0.319895201 | 0.027767786 |
| ENSG00000244038 | DDOST | 0.319911822 | 0.006045528 |
| ENSG00000177697 | CD151 | 0.319933349 | 0.006658846 |
| ENSG00000124831 | LRRFIP1 | 0.320192204 | 0.031264814 |
| ENSG00000253293 | HOXA10 | 0.320510025 | 0.037620011 |
| ENSG00000136940 | PDCL | 0.32062928 | 0.047233887 |
| ENSG00000101000 | PROCR | 0.320877704 | 0.004469253 |
| ENSG00000127837 | AAMP | 0.320998099 | 0.011378943 |
| ENSG00000175467 | SART1 | 0.321249141 | 0.004157288 |
| ENSG00000102910 | LONP2 | 0.321576586 | 0.005796863 |
| ENSG00000109794 | FAM149A | 0.32161655 | 0.033926368 |
| ENSG00000145191 | EIF2B5 | 0.32180252 | 0.011380718 |
| ENSG00000122958 | VPS26A | 0.322274246 | 0.001050134 |
| ENSG00000100731 | PCNX1 | 0.322274631 | 0.039538325 |
| ENSG00000013563 | DNASE1L1 | 0.322426871 | 0.008071767 |
| ENSG00000090060 | PAPOLA | 0.322563484 | 0.003308135 |
| ENSG00000115091 | ACTR3 | 0.322657837 | 0.001316667 |
| ENSG00000176658 | MYO1D | 0.322768356 | 0.009282982 |
| ENSG00000117000 | RLF | 0.323421949 | 0.009121168 |
| ENSG00000060339 | CCAR1 | 0.323909361 | 0.012945381 |
| ENSG00000139629 | GALNT6 | 0.324499384 | 0.010676173 |
| ENSG00000136193 | SCRN1 | 0.324540485 | 0.001075799 |
| ENSG00000070367 | EXOC5 | 0.325095343 | 0.007299615 |
| ENSG00000170348 | TMED10 | 0.32583574 | 0.000648757 |
| ENSG00000141759 | TXNL4A | 0.32618643 | 0.015557769 |
| ENSG00000176624 | MEX3C | 0.326247566 | 0.009058645 |
| ENSG00000107643 | MAPK8 | 0.326366837 | 0.002774461 |
| ENSG00000131323 | TRAF3 | 0.326722728 | 0.017865882 |
| ENSG00000137166 | FOXP4 | 0.326744617 | 0.047070478 |
| ENSG00000213523 | SRA1 | 0.32689345 | 0.034577846 |
| ENSG00000096433 | ITPR3 | 0.327219702 | 0.029969779 |
| ENSG00000105404 | RABAC1 | 0.327446746 | 0.007698677 |
| ENSG00000157450 | RNF111 | 0.327719008 | 0.008804383 |
| ENSG00000153339 | TRAPPC8 | 0.327847784 | 0.004215171 |
| ENSG00000198858 | R3HDM4 | 0.328062052 | 0.01327225 |
| ENSG00000076201 | PTPN23 | 0.328072639 | 0.037091417 |
| ENSG00000135823 | STX6 | 0.328251637 | 0.005315264 |
| ENSG00000213341 | CHUK | 0.328392241 | 0.004895666 |
| ENSG00000079277 | MKNK1 | 0.328630253 | 0.003904744 |
| ENSG00000111907 | TPD52L1 | 0.328788594 | 0.011967927 |
| ENSG00000011454 | RABGAP1 | 0.329668426 | 0.001047637 |
| ENSG00000164828 | SUN1 | 0.330684998 | 0.004691494 |
| ENSG00000175166 | PSMD2 | 0.330751603 | 0.003771042 |
| ENSG00000095380 | NANS | 0.330949887 | 0.028687272 |
| ENSG00000105229 | PIAS4 | 0.331153211 | 0.029238607 |
| ENSG00000172586 | CHCHD1 | 0.331510301 | 0.030931193 |
| ENSG00000106610 | STAG3L4 | 0.332108505 | 0.020211231 |
| ENSG00000162636 | FAM102B | 0.332376182 | 0.029836881 |
| ENSG00000198113 | TOR4A | 0.332836181 | 0.000224574 |
| ENSG00000104885 | DOT1L | 0.333286079 | 0.008710881 |
| ENSG00000111144 | LTA4H | 0.333807016 | 0.000630886 |
| ENSG00000143889 | HNRNPLL | 0.333889775 | 0.010388704 |
| ENSG00000181220 | ZNF746 | 0.33443289 | 0.00689069 |
| ENSG00000116260 | QSOX1 | 0.334571593 | 0.000165415 |
| ENSG00000155508 | CNOT8 | 0.334656747 | 0.011167107 |
| ENSG00000075856 | SART3 | 0.334794442 | 0.003557778 |
| ENSG00000114107 | CEP70 | 0.334859513 | 0.00939007 |
| ENSG00000089597 | GANAB | 0.335011115 | 0.003494368 |
| ENSG00000136485 | DCAF7 | 0.335609348 | 0.010194578 |
| ENSG00000143727 | ACP1 | 0.335684978 | 0.012837664 |
| ENSG00000106799 | TGFBR1 | 0.335751591 | 0.039008657 |
| ENSG00000174903 | RAB1B | 0.335836398 | 0.017722228 |
| ENSG00000125970 | RALY | 0.335910797 | 0.002214567 |
| ENSG00000177731 | FLII | 0.335938911 | 0.000123469 |
| ENSG00000164080 | RAD54L2 | 0.336093714 | 0.024189931 |
| ENSG00000166908 | PIP4K2C | 0.336550177 | 0.002123856 |
| ENSG00000065615 | CYB5R4 | 0.337245328 | 0.013208525 |
| ENSG00000176531 | PHLDB3 | 0.33760331 | 0.034717826 |
| ENSG00000141753 | IGFBP4 | 0.337667517 | 0.029163215 |
| ENSG00000081154 | PCNP | 0.337739922 | 0.001436441 |
| ENSG00000178078 | STAP2 | 0.337965149 | 0.001876883 |
| ENSG00000089248 | ERP29 | 0.338535034 | 0.002752019 |
| ENSG00000162517 | PEF1 | 0.338608989 | 0.020520728 |
| ENSG00000184903 | IMMP2L | 0.339110808 | 0.03624411 |
| ENSG00000070961 | ATP2B1 | 0.339137874 | 0.037432967 |
| ENSG00000144029 | MRPS5 | 0.339470328 | 0.000478014 |
| ENSG00000125821 | DTD1 | 0.340277254 | 0.016259359 |
| ENSG00000137942 | FNBP1L | 0.34057687 | 0.001730731 |
| ENSG00000119950 | MXI1 | 0.340822786 | 0.000394533 |
| ENSG00000143570 | SLC39A1 | 0.340855842 | 0.002496619 |
| ENSG00000084093 | REST | 0.341007947 | 0.003886594 |
| ENSG00000134698 | AGO4 | 0.341260707 | 0.037678406 |
| ENSG00000175215 | CTDSP2 | 0.341300997 | 0.00020732 |
| ENSG00000233101 | HOXB-AS3 | 0.341468765 | 0.042586784 |
| ENSG00000167553 | TUBA1C | 0.341722297 | 0.026639501 |
| ENSG00000160014 | CALM3 | 0.341998603 | 0.028994786 |
| ENSG00000196235 | SUPT5H | 0.342010349 | 0.003384075 |
| ENSG00000092108 | SCFD1 | 0.342133242 | 0.005347509 |
| ENSG00000184110 | EIF3C | 0.342298134 | 0.011493191 |
| ENSG00000075624 | ACTB | 0.342504828 | 0.014880005 |
| ENSG00000163527 | STT3B | 0.342516298 | 0.004135877 |
| ENSG00000122490 | PQLC1 | 0.342671271 | 0.006385832 |
| ENSG00000101310 | SEC23B | 0.342882713 | 0.00474751 |
| ENSG00000265491 | RNF115 | 0.343007328 | 0.0029399 |
| ENSG00000104936 | DMPK | 0.343109363 | 0.011293821 |
| ENSG00000101966 | XIAP | 0.343319773 | 0.005376073 |
| ENSG00000180233 | ZNRF2 | 0.343664853 | 0.022798941 |
| ENSG00000151689 | INPP1 | 0.343713224 | 0.006341614 |
| ENSG00000108433 | GOSR2 | 0.344037579 | 0.030375734 |
| ENSG00000182400 | TRAPPC6B | 0.34529812 | 0.021491298 |
| ENSG00000089289 | IGBP1 | 0.345438159 | 0.001193327 |
| ENSG00000118960 | HS1BP3 | 0.345641816 | 0.003626374 |
| ENSG00000134996 | OSTF1 | 0.345965133 | 0.006315779 |
| ENSG00000107897 | ACBD5 | 0.346105549 | 0.007076113 |
| ENSG00000214223 | HNRNPA1P10 | 0.346279068 | 0.006628482 |
| ENSG00000136715 | SAP130 | 0.346831821 | 0.013118644 |
| ENSG00000124789 | NUP153 | 0.346913306 | 0.011590292 |
| ENSG00000139722 | VPS37B | 0.347373546 | 0.004309911 |
| ENSG00000146386 | ABRACL | 0.347898756 | 0.005032683 |
| ENSG00000177479 | ARIH2 | 0.347958274 | 0.007303994 |
| ENSG00000118363 | SPCS2 | 0.348530704 | 0.005294433 |
| ENSG00000073921 | PICALM | 0.348713236 | 0.0000517 |
| ENSG00000269556 | TMEM185A | 0.348856785 | 0.006586297 |
| ENSG00000127511 | SIN3B | 0.348930581 | 0.002269864 |
| ENSG00000112739 | PRPF4B | 0.348965177 | 0.005207957 |
| ENSG00000120725 | SIL1 | 0.349073824 | 0.020495032 |
| ENSG00000011260 | UTP18 | 0.349438928 | 0.013332488 |
| ENSG00000107262 | BAG1 | 0.349571358 | 0.002357412 |
| ENSG00000134049 | IER3IP1 | 0.349753638 | 0.029380117 |
| ENSG00000139350 | NEDD1 | 0.349770796 | 0.006711747 |
| ENSG00000131187 | F12 | 0.349839099 | 0.037370324 |
| ENSG00000176946 | THAP4 | 0.349896311 | 0.022732196 |
| ENSG00000122705 | CLTA | 0.350400639 | 0.000441455 |
| ENSG00000189308 | LIN54 | 0.350574552 | 0.005768253 |
| ENSG00000109861 | CTSC | 0.350779844 | 0.002449191 |
| ENSG00000136770 | DNAJC1 | 0.350821468 | 0.020080856 |
| ENSG00000103994 | ZNF106 | 0.350879703 | 0.025488277 |
| ENSG00000106049 | HIBADH | 0.351064258 | 0.012526023 |
| ENSG00000063245 | EPN1 | 0.351300618 | 0.003067703 |
| ENSG00000110046 | ATG2A | 0.351555701 | 0.00383357 |
| ENSG00000135124 | P2RX4 | 0.351631277 | 0.047479933 |
| ENSG00000123836 | PFKFB2 | 0.351646408 | 0.008283466 |
| ENSG00000072506 | HSD17B10 | 0.352449689 | 0.00727906 |
| ENSG00000187051 | RPS19BP1 | 0.352600808 | 0.021977101 |
| ENSG00000196305 | IARS | 0.352781649 | 0.007486857 |
| ENSG00000151461 | UPF2 | 0.352883103 | 0.001291151 |
| ENSG00000172007 | RAB33B | 0.352964043 | 0.027378821 |
| ENSG00000116288 | PARK7 | 0.353116959 | 0.003187699 |
| ENSG00000183735 | TBK1 | 0.353467685 | 0.004410983 |
| ENSG00000179010 | MRFAP1 | 0.353592355 | 0.000761718 |
| ENSG00000115350 | POLE4 | 0.353733124 | 0.018262139 |
| ENSG00000106524 | ANKMY2 | 0.354474709 | 0.006387658 |
| ENSG00000101146 | RAE1 | 0.354510769 | 0.015291542 |
| ENSG00000125520 | SLC2A4RG | 0.355186936 | 0.006249619 |
| ENSG00000064961 | HMG20B | 0.355304717 | 0.022722955 |
| ENSG00000064419 | TNPO3 | 0.355396428 | 0.00568333 |
| ENSG00000165752 | STK32C | 0.355431099 | 0.047090468 |
| ENSG00000136379 | ABHD17C | 0.355752742 | 0.00479527 |
| ENSG00000151718 | WWC2 | 0.355893126 | 0.044861772 |
| ENSG00000171729 | TMEM51 | 0.355959267 | 0.006359682 |
| ENSG00000088808 | PPP1R13B | 0.356291977 | 0.010090408 |
| ENSG00000065427 | KARS | 0.356363052 | 0.003310662 |
| ENSG00000055070 | SZRD1 | 0.356377754 | 0.004104491 |
| ENSG00000116473 | RAP1A | 0.356424031 | 0.000634445 |
| ENSG00000167967 | E4F1 | 0.35689798 | 0.0353935 |
| ENSG00000071051 | NCK2 | 0.357038738 | 0.0019734 |
| ENSG00000225921 | NOL7 | 0.357127334 | 0.003121295 |
| ENSG00000174136 | RGMB | 0.357357443 | 0.039254587 |
| ENSG00000152102 | FAM168B | 0.357380993 | 0.000709655 |
| ENSG00000196968 | FUT11 | 0.357537861 | 0.036676768 |
| ENSG00000189043 | NDUFA4 | 0.357570108 | 0.025910606 |
| ENSG00000100225 | FBXO7 | 0.357827823 | 0.000929024 |
| ENSG00000054277 | OPN3 | 0.357909383 | 0.049727666 |
| ENSG00000130175 | PRKCSH | 0.358557623 | 0.000295978 |
| ENSG00000137814 | HAUS2 | 0.359114474 | 0.02926761 |
| ENSG00000110717 | NDUFS8 | 0.359701122 | 0.020014495 |
| ENSG00000111786 | SRSF9 | 0.359905842 | 0.009509817 |
| ENSG00000051620 | HEBP2 | 0.360159088 | 0.001778248 |
| ENSG00000126005 | MMP24OS | 0.360264327 | 0.003018308 |
| ENSG00000151779 | NBAS | 0.360599691 | 0.017479898 |
| ENSG00000143442 | POGZ | 0.360706148 | 0.012229706 |
| ENSG00000126524 | SBDS | 0.361216502 | 0.002826599 |
| ENSG00000176101 | SSNA1 | 0.361290525 | 0.00882023 |
| ENSG00000003436 | TFPI | 0.361693481 | 0.001427804 |
| ENSG00000169908 | TM4SF1 | 0.362162711 | 0.003930905 |
| ENSG00000105464 | GRIN2D | 0.362715717 | 0.010191227 |
| ENSG00000163162 | RNF149 | 0.363363235 | 0.002946745 |
| ENSG00000116857 | TMEM9 | 0.364186677 | 0.003713207 |
| ENSG00000171202 | TMEM126A | 0.364714023 | 0.013057566 |
| ENSG00000109111 | SUPT6H | 0.364970406 | 0.032875362 |
| ENSG00000257923 | CUX1 | 0.365231248 | 0.003426475 |
| ENSG00000091947 | TMEM101 | 0.365320241 | 0.007666445 |
| ENSG00000115415 | STAT1 | 0.365362051 | 0.005178985 |
| ENSG00000198408 | MGEA5 | 0.365465708 | 0.002113327 |
| ENSG00000033800 | PIAS1 | 0.365753532 | 0.000557902 |
| ENSG00000065518 | NDUFB4 | 0.365871806 | 0.012526104 |
| ENSG00000153989 | NUS1 | 0.366107701 | 0.002203794 |
| ENSG00000135677 | GNS | 0.366186589 | 0.002452542 |
| ENSG00000184281 | TSSC4 | 0.366301381 | 0.02854211 |
| ENSG00000109670 | FBXW7 | 0.366442448 | 0.016349872 |
| ENSG00000135441 | BLOC1S1 | 0.366645361 | 0.043885355 |
| ENSG00000005187 | ACSM3 | 0.36679813 | 0.016880998 |
| ENSG00000075643 | MOCOS | 0.366900391 | 0.02807333 |
| ENSG00000164535 | DAGLB | 0.367599328 | 0.02191767 |
| ENSG00000159216 | RUNX1 | 0.368007537 | 0.024898914 |
| ENSG00000106299 | WASL | 0.368837347 | 0.0000707 |
| ENSG00000092203 | TOX4 | 0.368860828 | 0.000668828 |
| ENSG00000139977 | NAA30 | 0.369018326 | 0.016993641 |
| ENSG00000177200 | CHD9 | 0.369088288 | 0.027606824 |
| ENSG00000132256 | TRIM5 | 0.369120614 | 0.003538395 |
| ENSG00000010292 | NCAPD2 | 0.369199634 | 0.026779818 |
| ENSG00000103335 | PIEZO1 | 0.369245766 | 0.004384729 |
| ENSG00000164024 | METAP1 | 0.36969013 | 0.004002988 |
| ENSG00000137831 | UACA | 0.369744653 | 0.001037426 |
| ENSG00000119185 | ITGB1BP1 | 0.369962841 | 0.005416901 |
| ENSG00000133731 | IMPA1 | 0.370111003 | 0.010366309 |
| ENSG00000138663 | COPS4 | 0.370358328 | 0.001741681 |
| ENSG00000067560 | RHOA | 0.37048788 | 0.001906111 |
| ENSG00000174748 | RPL15 | 0.370638768 | 0.000133437 |
| ENSG00000163605 | PPP4R2 | 0.370750724 | 0.000878315 |
| ENSG00000168610 | STAT3 | 0.37083647 | 0.000108385 |
| ENSG00000206527 | HACD2 | 0.370863301 | 0.003953362 |
| ENSG00000100412 | ACO2 | 0.370878324 | 0.012319505 |
| ENSG00000086598 | TMED2 | 0.371229346 | 0.030304995 |
| ENSG00000171204 | TMEM126B | 0.371798382 | 0.006408769 |
| ENSG00000149177 | PTPRJ | 0.37204488 | 0.002326476 |
| ENSG00000116574 | RHOU | 0.372261924 | 0.024893919 |
| ENSG00000232573 | RPL3P4 | 0.372765971 | 0.029444427 |
| ENSG00000131013 | PPIL4 | 0.372913829 | 0.005510756 |
| ENSG00000137710 | RDX | 0.373079124 | 0.000488549 |
| ENSG00000126453 | BCL2L12 | 0.373454196 | 0.037534003 |
| ENSG00000179632 | MAF1 | 0.373999472 | 0.009282982 |
| ENSG00000198612 | COPS8 | 0.374799062 | 0.007020588 |
| ENSG00000163694 | RBM47 | 0.375939468 | 0.004902839 |
| ENSG00000167130 | DOLPP1 | 0.376378075 | 0.0172601 |
| ENSG00000107949 | BCCIP | 0.376451171 | 0.002414601 |
| ENSG00000135476 | ESPL1 | 0.37663278 | 0.043032652 |
| ENSG00000113638 | TTC33 | 0.376733425 | 0.005221226 |
| ENSG00000154305 | MIA3 | 0.376805262 | 0.01538768 |
| ENSG00000025293 | PHF20 | 0.376959258 | 0.000285594 |
| ENSG00000149100 | EIF3M | 0.377060238 | 0.000055 |
| ENSG00000167491 | GATAD2A | 0.377148209 | 0.00259107 |
| ENSG00000101843 | PSMD10 | 0.377696235 | 0.014360222 |
| ENSG00000162385 | MAGOH | 0.377756223 | 0.006341614 |
| ENSG00000078808 | SDF4 | 0.378025641 | 0.003430894 |
| ENSG00000144233 | AMMECR1L | 0.378246569 | 0.003324695 |
| ENSG00000124171 | PARD6B | 0.378363067 | 0.004140402 |
| ENSG00000103495 | MAZ | 0.37901207 | 0.007231261 |
| ENSG00000224470 | ATXN1L | 0.379071351 | 0.001957465 |
| ENSG00000152219 | ARL14EP | 0.379419809 | 0.001677679 |
| ENSG00000179562 | GCC1 | 0.380163468 | 0.002945225 |
| ENSG00000124198 | ARFGEF2 | 0.380344008 | 0.005330845 |
| ENSG00000076053 | RBM7 | 0.380445613 | 0.003989879 |
| ENSG00000182149 | IST1 | 0.381117142 | 0.00012966 |
| ENSG00000152558 | TMEM123 | 0.381697555 | 0.00000573 |
| ENSG00000127526 | SLC35E1 | 0.381789992 | 0.005363214 |
| ENSG00000144228 | SPOPL | 0.382407512 | 0.001899838 |
| ENSG00000165264 | NDUFB6 | 0.382907421 | 0.016277945 |
| ENSG00000156675 | RAB11FIP1 | 0.383313479 | 0.001525786 |
| ENSG00000111716 | LDHB | 0.383965498 | 0.000635537 |
| ENSG00000077721 | UBE2A | 0.384142481 | 0.009534165 |
| ENSG00000197903 | HIST1H2BK | 0.384192025 | 0.030059864 |
| ENSG00000138433 | CIR1 | 0.384369185 | 0.002911832 |
| ENSG00000184924 | PTRHD1 | 0.384465732 | 0.034054544 |
| ENSG00000076043 | REXO2 | 0.384504905 | 0.006223218 |
| ENSG00000155975 | VPS37A | 0.384510761 | 0.028174457 |
| ENSG00000176915 | ANKLE2 | 0.38481621 | 0.00000847 |
| ENSG00000182544 | MFSD5 | 0.385030636 | 0.020693886 |
| ENSG00000204673 | AKT1S1 | 0.385179746 | 0.008654759 |
| ENSG00000162695 | SLC30A7 | 0.385546081 | 0.000985358 |
| ENSG00000102103 | PQBP1 | 0.385546985 | 0.01020094 |
| ENSG00000068912 | ERLEC1 | 0.385941627 | 0.024472103 |
| ENSG00000124155 | PIGT | 0.386143829 | 0.000251605 |
| ENSG00000140612 | SEC11A | 0.386177684 | 0.00103673 |
| ENSG00000118454 | ANKRD13C | 0.386639544 | 0.000770349 |
| ENSG00000175634 | RPS6KB2 | 0.38699223 | 0.011924178 |
| ENSG00000074527 | NTN4 | 0.387054819 | 0.017211374 |
| ENSG00000127952 | STYXL1 | 0.387106097 | 0.00677291 |
| ENSG00000198931 | APRT | 0.387497157 | 0.007152474 |
| ENSG00000132740 | IGHMBP2 | 0.38809673 | 0.020425926 |
| ENSG00000141627 | DYM | 0.388129732 | 0.000537567 |
| ENSG00000113282 | CLINT1 | 0.388166208 | 0.00005 |
| ENSG00000101901 | ALG13 | 0.388230259 | 0.001456727 |
| ENSG00000101150 | TPD52L2 | 0.388492568 | 0.006302092 |
| ENSG00000130311 | DDA1 | 0.388769024 | 0.000692149 |
| ENSG00000109079 | TNFAIP1 | 0.388822132 | 0.000735995 |
| ENSG00000105887 | MTPN | 0.389091352 | 0.000649774 |
| ENSG00000109519 | GRPEL1 | 0.389130234 | 0.00250439 |
| ENSG00000143543 | JTB | 0.389268165 | 0.016391851 |
| ENSG00000281649 | EBLN3P | 0.389554714 | 0.000271195 |
| ENSG00000214022 | REPIN1 | 0.389720987 | 0.000401074 |
| ENSG00000156304 | SCAF4 | 0.389781984 | 0.002362937 |
| ENSG00000116221 | MRPL37 | 0.389878185 | 0.003621212 |
| ENSG00000213614 | HEXA | 0.390189313 | 0.000839955 |
| ENSG00000118640 | VAMP8 | 0.39019439 | 0.004521804 |
| ENSG00000004059 | ARF5 | 0.390201982 | 0.001562659 |
| ENSG00000163558 | PRKCI | 0.390257473 | 0.0000231 |
| ENSG00000175216 | CKAP5 | 0.390893215 | 0.025924749 |
| ENSG00000065154 | OAT | 0.391128334 | 0.001971745 |
| ENSG00000178105 | DDX10 | 0.391131278 | 0.000446117 |
| ENSG00000178035 | IMPDH2 | 0.391153759 | 0.0000835 |
| ENSG00000138032 | PPM1B | 0.391160179 | 0.0000449 |
| ENSG00000112200 | ZNF451 | 0.391770172 | 0.004708448 |
| ENSG00000129197 | RPAIN | 0.39191771 | 0.004323404 |
| ENSG00000105722 | ERF | 0.392313433 | 0.003087227 |
| ENSG00000105339 | DENND3 | 0.392387007 | 0.002956301 |
| ENSG00000134851 | TMEM165 | 0.392747322 | 0.000986668 |
| ENSG00000186298 | PPP1CC | 0.392830571 | 0.00656469 |
| ENSG00000008018 | PSMB1 | 0.392918617 | 0.003714754 |
| ENSG00000082898 | XPO1 | 0.393076904 | 0.00026996 |
| ENSG00000010256 | UQCRC1 | 0.39312604 | 0.00367805 |
| ENSG00000105976 | MET | 0.393314542 | 0.002779975 |
| ENSG00000143420 | ENSA | 0.393370229 | 0.000559042 |
| ENSG00000120458 | MSANTD2 | 0.393425527 | 0.012053631 |
| ENSG00000180228 | PRKRA | 0.393752705 | 0.03580715 |
| ENSG00000188986 | NELFB | 0.394593565 | 0.007911977 |
| ENSG00000125652 | ALKBH7 | 0.394654256 | 0.005563771 |
| ENSG00000141644 | MBD1 | 0.39471868 | 0.003420835 |
| ENSG00000007545 | CRAMP1 | 0.394887434 | 0.015405905 |
| ENSG00000169564 | PCBP1 | 0.394995887 | 0.00021119 |
| ENSG00000129566 | TEP1 | 0.395967022 | 0.016539383 |
| ENSG00000186187 | ZNRF1 | 0.396225987 | 0.00621911 |
| ENSG00000080986 | NDC80 | 0.396314981 | 0.031992675 |
| ENSG00000173327 | MAP3K11 | 0.396417793 | 0.002913975 |
| ENSG00000171466 | ZNF562 | 0.396418132 | 0.001403954 |
| ENSG00000137074 | APTX | 0.396514389 | 0.00516874 |
| ENSG00000134153 | EMC7 | 0.396912296 | 0.004019593 |
| ENSG00000248333 | CDK11B | 0.396989626 | 0.000293425 |
| ENSG00000103061 | SLC7A6OS | 0.397038777 | 0.047671155 |
| ENSG00000100387 | RBX1 | 0.397288593 | 0.023248635 |
| ENSG00000085365 | SCAMP1 | 0.397335321 | 0.006162145 |
| ENSG00000101574 | METTL4 | 0.397639875 | 0.014785116 |
| ENSG00000134815 | DHX34 | 0.397667927 | 0.000543815 |
| ENSG00000129250 | KIF1C | 0.397704637 | 0.0000477 |
| ENSG00000137807 | KIF23 | 0.397779746 | 0.036067572 |
| ENSG00000067606 | PRKCZ | 0.397997821 | 0.005613532 |
| ENSG00000123131 | PRDX4 | 0.39802129 | 0.005239094 |
| ENSG00000130726 | TRIM28 | 0.398048616 | 0.000211859 |
| ENSG00000100242 | SUN2 | 0.398146225 | 0.001605758 |
| ENSG00000133142 | TCEAL4 | 0.398193157 | 0.012418087 |
| ENSG00000152601 | MBNL1 | 0.3982819 | 0.000326709 |
| ENSG00000060237 | WNK1 | 0.398385328 | 0.018609505 |
| ENSG00000113595 | TRIM23 | 0.398767774 | 0.008908807 |
| ENSG00000105963 | ADAP1 | 0.399111158 | 0.00178367 |
| ENSG00000076554 | TPD52 | 0.399137449 | 0.000315981 |
| ENSG00000171105 | INSR | 0.399391261 | 0.040499578 |
| ENSG00000184349 | EFNA5 | 0.399669141 | 0.007842427 |
| ENSG00000104343 | UBE2W | 0.399769833 | 0.000190882 |
| ENSG00000095787 | WAC | 0.399871888 | 0.00000244 |
| ENSG00000105373 | NOP53 | 0.400183051 | 0.0000583 |
| ENSG00000118680 | MYL12B | 0.40030554 | 0.001742524 |
| ENSG00000179588 | ZFPM1 | 0.400589912 | 0.012038661 |
| ENSG00000009307 | CSDE1 | 0.400591093 | 0.0000415 |
| ENSG00000111206 | FOXM1 | 0.400655376 | 0.048407176 |
| ENSG00000086062 | B4GALT1 | 0.400908688 | 0.000283122 |
| ENSG00000129932 | DOHH | 0.400927137 | 0.017247764 |
| ENSG00000109736 | MFSD10 | 0.400938013 | 0.000143216 |
| ENSG00000141562 | NARF | 0.401409592 | 0.04103636 |
| ENSG00000004455 | AK2 | 0.401508176 | 0.00018052 |
| ENSG00000107554 | DNMBP | 0.401922125 | 0.002218431 |
| ENSG00000109270 | LAMTOR3 | 0.40210477 | 0.000478962 |
| ENSG00000108799 | EZH1 | 0.402211462 | 0.009136897 |
| ENSG00000136522 | MRPL47 | 0.402228686 | 0.0062012 |
| ENSG00000146066 | HIGD2A | 0.403082281 | 0.006902493 |
| ENSG00000071462 | BUD23 | 0.403562816 | 0.001473978 |
| ENSG00000156858 | PRR14 | 0.403854674 | 0.018273655 |
| ENSG00000144840 | RABL3 | 0.404637272 | 0.009835449 |
| ENSG00000174943 | KCTD13 | 0.404666896 | 0.033427044 |
| ENSG00000214485 | RPL7P1 | 0.404731035 | 0.025753971 |
| ENSG00000136758 | YME1L1 | 0.404736964 | 0.0000981 |
| ENSG00000158109 | TPRG1L | 0.405012124 | 0.00075122 |
| ENSG00000177885 | GRB2 | 0.405190431 | 0.00019721 |
| ENSG00000101608 | MYL12A | 0.405360721 | 0.000125584 |
| ENSG00000121039 | RDH10 | 0.405360861 | 0.001287961 |
| ENSG00000084754 | HADHA | 0.405640612 | 0.0000405 |
| ENSG00000111880 | RNGTT | 0.406705152 | 0.004009983 |
| ENSG00000075188 | NUP37 | 0.406710612 | 0.032283891 |
| ENSG00000080298 | RFX3 | 0.406787117 | 0.029359316 |
| ENSG00000132819 | RBM38 | 0.407061119 | 0.001971087 |
| ENSG00000272886 | DCP1A | 0.407150329 | 0.000400144 |
| ENSG00000174851 | YIF1A | 0.407309249 | 0.019756205 |
| ENSG00000057252 | SOAT1 | 0.407420247 | 0.035930992 |
| ENSG00000163507 | CIP2A | 0.407667629 | 0.047663943 |
| ENSG00000135164 | DMTF1 | 0.408073949 | 0.0000542 |
| ENSG00000136950 | ARPC5L | 0.408139147 | 0.005683312 |
| ENSG00000103363 | ELOB | 0.408561779 | 0.001097297 |
| ENSG00000140830 | TXNL4B | 0.408567338 | 0.002040408 |
| ENSG00000166689 | PLEKHA7 | 0.408862709 | 0.000200107 |
| ENSG00000124920 | MYRF | 0.409111065 | 0.00363636 |
| ENSG00000131504 | DIAPH1 | 0.409154376 | 0.0000932 |
| ENSG00000183291 | SELENOF | 0.40931757 | 0.000750152 |
| ENSG00000162923 | WDR26 | 0.409517577 | 0.000857719 |
| ENSG00000100196 | KDELR3 | 0.409530416 | 0.049077954 |
| ENSG00000141448 | GATA6 | 0.409948327 | 0.008866712 |
| ENSG00000123106 | CCDC91 | 0.409959814 | 0.00882733 |
| ENSG00000119396 | RAB14 | 0.410007252 | 0.0000456 |
| ENSG00000173486 | FKBP2 | 0.4104766 | 0.013021428 |
| ENSG00000066455 | GOLGA5 | 0.410514246 | 0.013454199 |
| ENSG00000094975 | SUCO | 0.41076356 | 0.000192464 |
| ENSG00000175470 | PPP2R2D | 0.410877137 | 0.000913156 |
| ENSG00000055483 | USP36 | 0.410945099 | 0.002224174 |
| ENSG00000175390 | EIF3F | 0.411077336 | 0.000126826 |
| ENSG00000172175 | MALT1 | 0.411148811 | 0.002326038 |
| ENSG00000182240 | BACE2 | 0.411344841 | 0.002319954 |
| ENSG00000120868 | APAF1 | 0.411372234 | 0.002702533 |
| ENSG00000164754 | RAD21 | 0.412015626 | 0.003193774 |
| ENSG00000163512 | AZI2 | 0.412046249 | 0.013021428 |
| ENSG00000156384 | SFR1 | 0.412091 | 0.026623063 |
| ENSG00000157014 | TATDN2 | 0.412152969 | 0.004401666 |
| ENSG00000107438 | PDLIM1 | 0.412779874 | 0.0000291 |
| ENSG00000079432 | CIC | 0.413007689 | 0.003137064 |
| ENSG00000138279 | ANXA7 | 0.413021294 | 0.0000466 |
| ENSG00000086065 | CHMP5 | 0.413115924 | 0.000460955 |
| ENSG00000220842 | RPL21P16 | 0.413325616 | 0.024285858 |
| ENSG00000103111 | MON1B | 0.41371463 | 0.000692149 |
| ENSG00000213096 | ZNF254 | 0.413756576 | 0.022960932 |
| ENSG00000136738 | STAM | 0.413779887 | 0.000638813 |
| ENSG00000100075 | SLC25A1 | 0.414340662 | 0.032447227 |
| ENSG00000035115 | SH3YL1 | 0.414420907 | 0.001805734 |
| ENSG00000170881 | RNF139 | 0.414436752 | 0.000366899 |
| ENSG00000111639 | MRPL51 | 0.414758018 | 0.004347359 |
| ENSG00000118579 | MED28 | 0.414864358 | 0.001084405 |
| ENSG00000182979 | MTA1 | 0.414973043 | 0.000352569 |
| ENSG00000143819 | EPHX1 | 0.415292404 | 0.005299947 |
| ENSG00000175203 | DCTN2 | 0.415660723 | 0.000196034 |
| ENSG00000102225 | CDK16 | 0.415744744 | 0.002147858 |
| ENSG00000048544 | MRPS10 | 0.415853398 | 0.000102069 |
| ENSG00000158636 | EMSY | 0.415975748 | 0.017182577 |
| ENSG00000240682 | ISY1 | 0.415977843 | 0.000645634 |
| ENSG00000059758 | CDK17 | 0.416117945 | 0.001335682 |
| ENSG00000140153 | WDR20 | 0.416302678 | 0.01421056 |
| ENSG00000136938 | ANP32B | 0.416515645 | 0.00000146 |
| ENSG00000008283 | CYB561 | 0.416545541 | 0.002670466 |
| ENSG00000114439 | BBX | 0.416857708 | 0.001418405 |
| ENSG00000157869 | RAB28 | 0.416909567 | 0.008707677 |
| ENSG00000112977 | DAP | 0.417128536 | 0.000229191 |
| ENSG00000122068 | FYTTD1 | 0.417679638 | 0.000027 |
| ENSG00000105968 | H2AFV | 0.417684219 | 0.003480566 |
| ENSG00000150753 | CCT5 | 0.417701618 | 0.00000497 |
| ENSG00000215421 | ZNF407 | 0.417899466 | 0.029037487 |
| ENSG00000143545 | RAB13 | 0.417957808 | 0.000526864 |
| ENSG00000013306 | SLC25A39 | 0.418274843 | 0.001055167 |
| ENSG00000111229 | ARPC3 | 0.418541922 | 0.001215746 |
| ENSG00000176092 | CRYBG2 | 0.419159001 | 0.011519655 |
| ENSG00000178982 | EIF3K | 0.41916547 | 0.00027689 |
| ENSG00000155621 | C9orf85 | 0.419198306 | 0.002005786 |
| ENSG00000113712 | CSNK1A1 | 0.419343269 | 0.00000279 |
| ENSG00000105518 | TMEM205 | 0.419652121 | 0.006613734 |
| ENSG00000144635 | DYNC1LI1 | 0.419796876 | 0.001149005 |
| ENSG00000071205 | ARHGAP10 | 0.419823973 | 0.026982422 |
| ENSG00000136709 | WDR33 | 0.419961409 | 0.0000482 |
| ENSG00000144867 | SRPRB | 0.420027369 | 0.013945625 |
| ENSG00000138081 | FBXO11 | 0.420285769 | 0.000232577 |
| ENSG00000100796 | PPP4R3A | 0.420494016 | 0.000169568 |
| ENSG00000116337 | AMPD2 | 0.420511769 | 0.006879981 |
| ENSG00000104765 | BNIP3L | 0.420637606 | 0.002660281 |
| ENSG00000134884 | ARGLU1 | 0.420732628 | 0.001258758 |
| ENSG00000239672 | NME1 | 0.420847027 | 0.007226578 |
| ENSG00000205476 | CCDC85C | 0.420917176 | 0.000438985 |
| ENSG00000129559 | NEDD8 | 0.421094781 | 0.002676229 |
| ENSG00000156261 | CCT8 | 0.421166573 | 0.001173425 |
| ENSG00000111110 | PPM1H | 0.421302028 | 0.000365275 |
| ENSG00000182446 | NPLOC4 | 0.421490307 | 0.0000219 |
| ENSG00000168303 | MPLKIP | 0.421545046 | 0.003803076 |
| ENSG00000165891 | E2F7 | 0.421693459 | 0.046049207 |
| ENSG00000118564 | FBXL5 | 0.421794269 | 0.019877723 |
| ENSG00000179967 | PPP1R14BP3 | 0.421934341 | 0.0077143 |
| ENSG00000147533 | GOLGA7 | 0.421946023 | 0.006960696 |
| ENSG00000166822 | TMEM170A | 0.422399142 | 0.000163478 |
| ENSG00000068724 | TTC7A | 0.422500374 | 0.007513502 |
| ENSG00000086589 | RBM22 | 0.42274385 | 0.00000282 |
| ENSG00000153317 | ASAP1 | 0.422794363 | 0.008521671 |
| ENSG00000028203 | VEZT | 0.422801734 | 0.005250538 |
| ENSG00000101882 | NKAP | 0.422852251 | 0.000868222 |
| ENSG00000134480 | CCNH | 0.423270842 | 0.000756811 |
| ENSG00000172366 | MCRIP2 | 0.423436754 | 0.017500575 |
| ENSG00000133275 | CSNK1G2 | 0.423534565 | 0.001637265 |
| ENSG00000224578 | HNRNPA1P48 | 0.423645622 | 0.005210018 |
| ENSG00000254999 | BRK1 | 0.423691578 | 0.000693579 |
| ENSG00000178719 | GRINA | 0.423735305 | 0.000224397 |
| ENSG00000143375 | CGN | 0.423771887 | 0.005294228 |
| ENSG00000135250 | SRPK2 | 0.424008676 | 0.0000173 |
| ENSG00000097033 | SH3GLB1 | 0.424506217 | 0.003088745 |
| ENSG00000105556 | MIER2 | 0.424556207 | 0.0141759 |
| ENSG00000142655 | PEX14 | 0.425439605 | 0.004134477 |
| ENSG00000162482 | AKR7A3 | 0.425586043 | 0.004564344 |
| ENSG00000163235 | TGFA | 0.425752513 | 0.00023473 |
| ENSG00000182208 | MOB2 | 0.425860604 | 0.000661601 |
| ENSG00000148334 | PTGES2 | 0.427328894 | 0.0000294 |
| ENSG00000060971 | ACAA1 | 0.427587881 | 0.00033643 |
| ENSG00000119335 | SET | 0.428038744 | 0.000000562 |
| ENSG00000145216 | FIP1L1 | 0.428193771 | 0.00545065 |
| ENSG00000130803 | ZNF317 | 0.428501745 | 0.0000137 |
| ENSG00000165355 | FBXO33 | 0.428649068 | 0.001931663 |
| ENSG00000181588 | MEX3D | 0.428700966 | 0.001668293 |
| ENSG00000103126 | AXIN1 | 0.429434745 | 0.000029 |
| ENSG00000117262 | GPR89A | 0.430461737 | 0.031693096 |
| ENSG00000158552 | ZFAND2B | 0.430677486 | 0.002632018 |
| ENSG00000126215 | XRCC3 | 0.431395775 | 0.043414682 |
| ENSG00000117691 | NENF | 0.431739067 | 0.000765724 |
| ENSG00000095574 | IKZF5 | 0.432011776 | 0.000864356 |
| ENSG00000156273 | BACH1 | 0.432284188 | 0.0105625 |
| ENSG00000143774 | GUK1 | 0.432347037 | 0.004165515 |
| ENSG00000120798 | NR2C1 | 0.432445857 | 0.000411762 |
| ENSG00000147471 | PLPBP | 0.432582037 | 0.007571121 |
| ENSG00000147162 | OGT | 0.432959365 | 0.017330423 |
| ENSG00000077458 | FAM76B | 0.433384154 | 0.000725821 |
| ENSG00000010278 | CD9 | 0.433617254 | 0.000356839 |
| ENSG00000154978 | VOPP1 | 0.433680936 | 0.000586073 |
| ENSG00000213753 | CENPBD1P1 | 0.433893207 | 0.001448933 |
| ENSG00000142444 | TIMM29 | 0.433998347 | 0.008310823 |
| ENSG00000144034 | TPRKB | 0.434087059 | 0.000595417 |
| ENSG00000112237 | CCNC | 0.434149481 | 0.001483965 |
| ENSG00000124783 | SSR1 | 0.434294774 | 0.0000161 |
| ENSG00000183726 | TMEM50A | 0.434690356 | 0.000438981 |
| ENSG00000124688 | MAD2L1BP | 0.434755649 | 0.000219674 |
| ENSG00000173272 | MZT2A | 0.435603616 | 0.004552782 |
| ENSG00000099617 | EFNA2 | 0.435919553 | 0.003300754 |
| ENSG00000168566 | SNRNP48 | 0.436180832 | 0.004091571 |
| ENSG00000102144 | PGK1 | 0.436296061 | 0.000409498 |
| ENSG00000105576 | TNPO2 | 0.436301511 | 0.000587967 |
| ENSG00000129473 | BCL2L2 | 0.436723818 | 0.000128258 |
| ENSG00000123143 | PKN1 | 0.436736332 | 0.0000222 |
| ENSG00000185104 | FAF1 | 0.437234912 | 0.000313117 |
| ENSG00000109436 | TBC1D9 | 0.437384081 | 0.000193268 |
| ENSG00000148773 | MKI67 | 0.437435475 | 0.048216613 |
| ENSG00000160789 | LMNA | 0.437490893 | 0.000144397 |
| ENSG00000125753 | VASP | 0.437662077 | 0.00028761 |
| ENSG00000012983 | MAP4K5 | 0.437813661 | 0.002224174 |
| ENSG00000179085 | DPM3 | 0.437848353 | 0.031302922 |
| ENSG00000138050 | THUMPD2 | 0.438156076 | 0.006966256 |
| ENSG00000074657 | ZNF532 | 0.438221032 | 0.036163462 |
| ENSG00000103534 | TMC5 | 0.438351269 | 0.00026603 |
| ENSG00000076604 | TRAF4 | 0.438362988 | 0.000443587 |
| ENSG00000109133 | TMEM33 | 0.438816688 | 0.0000485 |
| ENSG00000124782 | RREB1 | 0.439102568 | 0.009490684 |
| ENSG00000020426 | MNAT1 | 0.439266278 | 0.000652797 |
| ENSG00000143198 | MGST3 | 0.439840494 | 0.00069779 |
| ENSG00000250317 | SMIM20 | 0.440019851 | 0.0000616 |
| ENSG00000171067 | C11orf24 | 0.440369104 | 0.031400214 |
| ENSG00000256060 | TRAPPC2B | 0.440928066 | 0.047450025 |
| ENSG00000128245 | YWHAH | 0.44106087 | 0.006279291 |
| ENSG00000138107 | ACTR1A | 0.441300192 | 0.000479261 |
| ENSG00000165219 | GAPVD1 | 0.441302445 | 0.000915879 |
| ENSG00000172534 | HCFC1 | 0.441523667 | 0.005374262 |
| ENSG00000173281 | PPP1R3B | 0.441986509 | 0.020981035 |
| ENSG00000129235 | TXNDC17 | 0.442578246 | 0.007177545 |
| ENSG00000155368 | DBI | 0.442882678 | 0.005036902 |
| ENSG00000112893 | MAN2A1 | 0.443234355 | 0.0000166 |
| ENSG00000176058 | TPRN | 0.443409479 | 0.000620044 |
| ENSG00000101654 | RNMT | 0.443572238 | 0.00000935 |
| ENSG00000103326 | CAPN15 | 0.443636321 | 0.000667196 |
| ENSG00000167644 | C19orf33 | 0.443847736 | 0.037004745 |
| ENSG00000174695 | TMEM167A | 0.444079562 | 0.0000441 |
| ENSG00000100697 | DICER1 | 0.444243036 | 0.010569588 |
| ENSG00000158158 | CNNM4 | 0.44454089 | 0.004001473 |
| ENSG00000233426 | EIF3FP3 | 0.444810545 | 0.000303998 |
| ENSG00000170293 | CMTM8 | 0.444957137 | 0.00142565 |
| ENSG00000117385 | P3H1 | 0.444969462 | 0.006356637 |
| ENSG00000187231 | SESTD1 | 0.445540004 | 0.025426908 |
| ENSG00000068394 | GPKOW | 0.445691192 | 0.000785831 |
| ENSG00000070882 | OSBPL3 | 0.445750271 | 0.003160133 |
| ENSG00000171988 | JMJD1C | 0.446213701 | 0.005124628 |
| ENSG00000163535 | SGO2 | 0.446607853 | 0.003498806 |
| ENSG00000110711 | AIP | 0.446883035 | 0.002110478 |
| ENSG00000015475 | BID | 0.446961746 | 0.000715566 |
| ENSG00000153066 | TXNDC11 | 0.447128214 | 0.002596242 |
| ENSG00000067225 | PKM | 0.44731628 | 0.0000162 |
| ENSG00000169136 | ATF5 | 0.447563528 | 0.040324534 |
| ENSG00000112312 | GMNN | 0.448540845 | 0.034748512 |
| ENSG00000177156 | TALDO1 | 0.448571097 | 0.000184003 |
| ENSG00000205581 | HMGN1 | 0.448708368 | 0.000165244 |
| ENSG00000178741 | COX5A | 0.448801567 | 0.000868286 |
| ENSG00000105700 | KXD1 | 0.449633288 | 0.000924733 |
| ENSG00000099337 | KCNK6 | 0.449931154 | 0.000332482 |
| ENSG00000144597 | EAF1 | 0.450216599 | 0.0000404 |
| ENSG00000140995 | DEF8 | 0.450250577 | 0.000210977 |
| ENSG00000057663 | ATG5 | 0.450472152 | 0.000692636 |
| ENSG00000116691 | MIIP | 0.451356046 | 0.010559828 |
| ENSG00000095951 | HIVEP1 | 0.451787618 | 0.009325599 |
| ENSG00000181274 | FRAT2 | 0.45187252 | 0.00351994 |
| ENSG00000181163 | NPM1 | 0.451956171 | 0.000000522 |
| ENSG00000196976 | LAGE3 | 0.452174606 | 0.005739215 |
| ENSG00000176986 | SEC24C | 0.452196862 | 0.002034611 |
| ENSG00000141401 | IMPA2 | 0.452610174 | 0.012152496 |
| ENSG00000130958 | SLC35D2 | 0.452830814 | 0.00000903 |
| ENSG00000146731 | CCT6A | 0.452971272 | 0.000207953 |
| ENSG00000144746 | ARL6IP5 | 0.453091136 | 0.001006048 |
| ENSG00000149541 | B3GAT3 | 0.453141843 | 0.005893841 |
| ENSG00000137817 | PARP6 | 0.453149374 | 0.002513908 |
| ENSG00000074416 | MGLL | 0.453632177 | 0.0000365 |
| ENSG00000213609 | RPL7AP50 | 0.453752321 | 0.012721134 |
| ENSG00000101745 | ANKRD12 | 0.453816463 | 0.000159974 |
| ENSG00000099797 | TECR | 0.453956944 | 0.006549947 |
| ENSG00000107679 | PLEKHA1 | 0.455075877 | 0.00000655 |
| ENSG00000102218 | RP2 | 0.455472133 | 0.000253061 |
| ENSG00000184232 | OAF | 0.455486101 | 0.000124425 |
| ENSG00000123473 | STIL | 0.455501855 | 0.01293134 |
| ENSG00000214530 | STARD10 | 0.455757496 | 0.001299178 |
| ENSG00000160058 | BSDC1 | 0.456405565 | 0.000615067 |
| ENSG00000068745 | IP6K2 | 0.456660382 | 0.0000145 |
| ENSG00000169689 | CENPX | 0.456747088 | 0.032045325 |
| ENSG00000198356 | ASNA1 | 0.457018634 | 0.001706086 |
| ENSG00000147684 | NDUFB9 | 0.457074723 | 0.000595118 |
| ENSG00000168495 | POLR3D | 0.457627205 | 0.003747914 |
| ENSG00000173540 | GMPPB | 0.457821198 | 0.004104491 |
| ENSG00000102572 | STK24 | 0.457954939 | 0.0000128 |
| ENSG00000065135 | GNAI3 | 0.458020084 | 0.000234772 |
| ENSG00000138430 | OLA1 | 0.458064511 | 0.0000154 |
| ENSG00000184216 | IRAK1 | 0.45833061 | 0.0000763 |
| ENSG00000183298 | RPSAP19 | 0.458340948 | 0.017211374 |
| ENSG00000169045 | HNRNPH1 | 0.458824667 | 0.0000135 |
| ENSG00000103496 | STX4 | 0.459412639 | 0.000109604 |
| ENSG00000136986 | DERL1 | 0.459763532 | 0.000910874 |
| ENSG00000137075 | RNF38 | 0.460276635 | 0.000995273 |
| ENSG00000162910 | MRPL55 | 0.460372495 | 0.021224534 |
| ENSG00000157538 | VPS26C | 0.460526721 | 0.003630451 |
| ENSG00000130150 | MOSPD2 | 0.460693157 | 0.001498585 |
| ENSG00000274779 |  | 0.460761554 | 0.049763028 |
| ENSG00000166233 | ARIH1 | 0.460782099 | 0.00000267 |
| ENSG00000145919 | BOD1 | 0.46079458 | 0.006437664 |
| ENSG00000136682 | CBWD2 | 0.46079538 | 0.003123223 |
| ENSG00000003402 | CFLAR | 0.461204887 | 0.000166845 |
| ENSG00000076944 | STXBP2 | 0.461659624 | 0.0000439 |
| ENSG00000175029 | CTBP2 | 0.461667517 | 0.000000165 |
| ENSG00000163636 | PSMD6 | 0.461683668 | 0.000350891 |
| ENSG00000163001 | CFAP36 | 0.461699455 | 0.0000325 |
| ENSG00000133935 | ERG28 | 0.461999161 | 0.012556603 |
| ENSG00000143575 | HAX1 | 0.46218856 | 0.000562595 |
| ENSG00000205208 | C4orf46 | 0.46220674 | 0.03375557 |
| ENSG00000166037 | CEP57 | 0.462739918 | 0.000207986 |
| ENSG00000078369 | GNB1 | 0.462900719 | 0.000000341 |
| ENSG00000065548 | ZC3H15 | 0.463270445 | 0.00000579 |
| ENSG00000115183 | TANC1 | 0.464039017 | 0.00098692 |
| ENSG00000165244 | ZNF367 | 0.464373427 | 0.037534003 |
| ENSG00000175311 | ANKS4B | 0.46443026 | 0.03268114 |
| ENSG00000115808 | STRN | 0.464510582 | 0.00238961 |
| ENSG00000117118 | SDHB | 0.464609916 | 0.000505484 |
| ENSG00000127418 | FGFRL1 | 0.464866571 | 0.000138176 |
| ENSG00000233762 | AC007969.1 | 0.464893699 | 0.037450861 |
| ENSG00000143977 | SNRPG | 0.464997598 | 0.029732152 |
| ENSG00000197321 | SVIL | 0.465012331 | 0.001405947 |
| ENSG00000159840 | ZYX | 0.465289217 | 0.016821042 |
| ENSG00000058272 | PPP1R12A | 0.465309267 | 0.0000164 |
| ENSG00000204149 | AGAP6 | 0.465539047 | 0.006557962 |
| ENSG00000145016 | RUBCN | 0.465600437 | 0.000545818 |
| ENSG00000167625 | ZNF526 | 0.465855367 | 0.008046651 |
| ENSG00000102265 | TIMP1 | 0.466521309 | 0.000646732 |
| ENSG00000143970 | ASXL2 | 0.466549791 | 0.005125392 |
| ENSG00000130202 | NECTIN2 | 0.467041107 | 0.0000278 |
| ENSG00000144401 | METTL21A | 0.467073766 | 0.00778619 |
| ENSG00000189403 | HMGB1 | 0.467191474 | 0.001943429 |
| ENSG00000116698 | SMG7 | 0.467632592 | 0.00000538 |
| ENSG00000164975 | SNAPC3 | 0.467911154 | 0.004937523 |
| ENSG00000270629 | NBPF14 | 0.468052026 | 0.006359682 |
| ENSG00000133773 | CCDC59 | 0.468069363 | 0.0000654 |
| ENSG00000179262 | RAD23A | 0.4681885 | 0.000414958 |
| ENSG00000163191 | S100A11 | 0.468761056 | 0.000743339 |
| ENSG00000112339 | HBS1L | 0.4693774 | 0.000692149 |
| ENSG00000108061 | SHOC2 | 0.469392297 | 0.0000149 |
| ENSG00000125534 | PPDPF | 0.469624885 | 0.00003 |
| ENSG00000149260 | CAPN5 | 0.469933621 | 0.001953616 |
| ENSG00000174607 | UGT8 | 0.469970839 | 0.000466051 |
| ENSG00000164758 | MED30 | 0.47070488 | 0.001439687 |
| ENSG00000139644 | TMBIM6 | 0.470753268 | 0.0000136 |
| ENSG00000218891 | ZNF579 | 0.470936161 | 0.000172756 |
| ENSG00000163611 | SPICE1 | 0.471075031 | 0.020844601 |
| ENSG00000166987 | MBD6 | 0.47128128 | 0.000111761 |
| ENSG00000179604 | CDC42EP4 | 0.471323993 | 0.00000117 |
| ENSG00000133318 | RTN3 | 0.471671672 | 0.0000676 |
| ENSG00000166508 | MCM7 | 0.471815489 | 0.012381981 |
| ENSG00000036257 | CUL3 | 0.471981088 | 0.00000422 |
| ENSG00000147324 | MFHAS1 | 0.472039649 | 0.00000581 |
| ENSG00000130811 | EIF3G | 0.472167618 | 0.0000899 |
| ENSG00000137221 | TJAP1 | 0.472203405 | 0.0008121 |
| ENSG00000072062 | PRKACA | 0.472251952 | 0.000272833 |
| ENSG00000144645 | OSBPL10 | 0.472287872 | 0.0000124 |
| ENSG00000153443 | UBALD1 | 0.472436366 | 0.003601146 |
| ENSG00000137364 | TPMT | 0.472447703 | 0.000657588 |
| ENSG00000158526 | TSR2 | 0.472751709 | 0.0000861 |
| ENSG00000124201 | ZNFX1 | 0.473194456 | 0.003354909 |
| ENSG00000149187 | CELF1 | 0.473489422 | 0.00000132 |
| ENSG00000169379 | ARL13B | 0.473497536 | 0.000198791 |
| ENSG00000090776 | EFNB1 | 0.473835216 | 2.63E-08 |
| ENSG00000076685 | NT5C2 | 0.474128557 | 0.00000244 |
| ENSG00000151923 | TIAL1 | 0.474409773 | 0.0000122 |
| ENSG00000107372 | ZFAND5 | 0.474581337 | 0.000000116 |
| ENSG00000095203 | EPB41L4B | 0.474795989 | 0.0000175 |
| ENSG00000171421 | MRPL36 | 0.474812533 | 0.005375121 |
| ENSG00000140521 | POLG | 0.475040016 | 0.00882023 |
| ENSG00000114383 | TUSC2 | 0.475073071 | 0.001308041 |
| ENSG00000188313 | PLSCR1 | 0.475120736 | 0.0000487 |
| ENSG00000103966 | EHD4 | 0.47519877 | 0.000106342 |
| ENSG00000170860 | LSM3 | 0.475296526 | 0.008285455 |
| ENSG00000272333 | KMT2B | 0.475422583 | 0.0000337 |
| ENSG00000164134 | NAA15 | 0.475614759 | 0.00000666 |
| ENSG00000164830 | OXR1 | 0.475873135 | 0.0000593 |
| ENSG00000143398 | PIP5K1A | 0.475895901 | 0.0000437 |
| ENSG00000189266 | PNRC2 | 0.475997579 | 0.000000723 |
| ENSG00000116977 | LGALS8 | 0.476135216 | 0.00044765 |
| ENSG00000197183 | NOL4L | 0.476137567 | 0.000012 |
| ENSG00000148737 | TCF7L2 | 0.47635901 | 0.014622708 |
| ENSG00000174939 | ASPHD1 | 0.476711232 | 0.00780254 |
| ENSG00000135945 | REV1 | 0.477271151 | 0.000263506 |
| ENSG00000092208 | GEMIN2 | 0.477323229 | 0.033953158 |
| ENSG00000130669 | PAK4 | 0.477412685 | 0.0000307 |
| ENSG00000105732 | ZNF574 | 0.477851373 | 0.000684506 |
| ENSG00000106105 | GARS | 0.477908479 | 0.006951323 |
| ENSG00000136122 | BORA | 0.477974861 | 0.018756846 |
| ENSG00000186184 | POLR1D | 0.478211496 | 0.000131504 |
| ENSG00000182484 | WASH6P | 0.478308994 | 0.014183942 |
| ENSG00000121766 | ZCCHC17 | 0.478529922 | 0.00950855 |
| ENSG00000245848 | CEBPA | 0.478545734 | 0.000865749 |
| ENSG00000156052 | GNAQ | 0.478645318 | 0.0000199 |
| ENSG00000109046 | WSB1 | 0.478837948 | 0.000278899 |
| ENSG00000152078 | TMEM56 | 0.47889478 | 0.005869293 |
| ENSG00000107833 | NPM3 | 0.478919747 | 0.000539875 |
| ENSG00000125743 | SNRPD2 | 0.48026174 | 0.001047713 |
| ENSG00000116251 | RPL22 | 0.480598397 | 0.00000416 |
| ENSG00000166986 | MARS | 0.481049235 | 0.001381327 |
| ENSG00000157540 | DYRK1A | 0.481225007 | 0.0000511 |
| ENSG00000120656 | TAF12 | 0.481241435 | 0.001395215 |
| ENSG00000125835 | SNRPB | 0.481510767 | 0.00079811 |
| ENSG00000235776 | AC000089.1 | 0.481699446 | 0.005299372 |
| ENSG00000241741 | RPL7AP30 | 0.482061496 | 0.0000466 |
| ENSG00000158467 | AHCYL2 | 0.482131539 | 0.001458189 |
| ENSG00000171843 | MLLT3 | 0.48222538 | 0.0000141 |
| ENSG00000171159 | C9orf16 | 0.482302207 | 0.00041671 |
| ENSG00000120533 | ENY2 | 0.482611933 | 0.0000672 |
| ENSG00000219545 | UMAD1 | 0.48270967 | 0.003449919 |
| ENSG00000104231 | ZFAND1 | 0.484107641 | 0.0000448 |
| ENSG00000168615 | ADAM9 | 0.484166383 | 0.0000117 |
| ENSG00000123159 | GIPC1 | 0.484492983 | 0.0000067 |
| ENSG00000164172 | MOCS2 | 0.484578389 | 0.000297159 |
| ENSG00000169020 | ATP5ME | 0.484816639 | 0.006175825 |
| ENSG00000119431 | HDHD3 | 0.485027692 | 0.000103944 |
| ENSG00000085433 | WDR47 | 0.485588037 | 0.006804778 |
| ENSG00000132003 | ZSWIM4 | 0.485864278 | 0.001657419 |
| ENSG00000104897 | SF3A2 | 0.486097494 | 0.000218714 |
| ENSG00000167757 | KLK11 | 0.486105883 | 0.00000928 |
| ENSG00000092758 | COL9A3 | 0.486404972 | 0.008872306 |
| ENSG00000134046 | MBD2 | 0.486793862 | 0.000203666 |
| ENSG00000004777 | ARHGAP33 | 0.486873376 | 0.014601183 |
| ENSG00000070669 | ASNS | 0.486889668 | 0.01140659 |
| ENSG00000165169 | DYNLT3 | 0.486968151 | 0.0000191 |
| ENSG00000213639 | PPP1CB | 0.487067635 | 0.00000701 |
| ENSG00000167978 | SRRM2 | 0.487228149 | 0.015815604 |
| ENSG00000139116 | KIF21A | 0.487400977 | 0.0000109 |
| ENSG00000249353 | NPM1P27 | 0.487434445 | 0.00237904 |
| ENSG00000152404 | CWF19L2 | 0.487440013 | 0.001025903 |
| ENSG00000005075 | POLR2J | 0.487668542 | 0.0006553 |
| ENSG00000106086 | PLEKHA8 | 0.487675229 | 0.001617172 |
| ENSG00000162729 | IGSF8 | 0.487913931 | 0.000033 |
| ENSG00000211450 | SELENOH | 0.488419973 | 0.011553671 |
| ENSG00000116898 | MRPS15 | 0.489452683 | 0.003430894 |
| ENSG00000185215 | TNFAIP2 | 0.489626072 | 0.000979083 |
| ENSG00000145425 | RPS3A | 0.489973064 | 0.001055167 |
| ENSG00000022840 | RNF10 | 0.490227068 | 9.25E-08 |
| ENSG00000141552 | ANAPC11 | 0.49072591 | 0.003575335 |
| ENSG00000110801 | PSMD9 | 0.490823808 | 0.005568805 |
| ENSG00000127589 | TUBBP1 | 0.490956447 | 0.047479756 |
| ENSG00000116747 | TROVE2 | 0.491107309 | 0.0000805 |
| ENSG00000139880 | CDH24 | 0.491236851 | 0.011499253 |
| ENSG00000103066 | PLA2G15 | 0.491873658 | 0.009191468 |
| ENSG00000214517 | PPME1 | 0.491874677 | 0.000610405 |
| ENSG00000230989 | HSBP1 | 0.491970453 | 0.001241685 |
| ENSG00000169139 | UBE2V2 | 0.492524428 | 0.0000409 |
| ENSG00000126777 | KTN1 | 0.492585758 | 0.0000258 |
| ENSG00000185627 | PSMD13 | 0.492808786 | 0.000625547 |
| ENSG00000109084 | TMEM97 | 0.493028372 | 0.018826032 |
| ENSG00000116030 | SUMO1 | 0.493045136 | 0.000013 |
| ENSG00000110171 | TRIM3 | 0.493078454 | 0.008715118 |
| ENSG00000103353 | UBFD1 | 0.493308157 | 0.000171763 |
| ENSG00000171311 | EXOSC1 | 0.49345587 | 0.002064252 |
| ENSG00000125107 | CNOT1 | 0.493531116 | 0.000330146 |
| ENSG00000116670 | MAD2L2 | 0.493634943 | 0.011073522 |
| ENSG00000099817 | POLR2E | 0.493860787 | 0.000436167 |
| ENSG00000150593 | PDCD4 | 0.494036748 | 0.0000981 |
| ENSG00000186063 | AIDA | 0.494076873 | 0.000246362 |
| ENSG00000125995 | ROMO1 | 0.494758084 | 0.001091942 |
| ENSG00000155097 | ATP6V1C1 | 0.494836212 | 0.0000197 |
| ENSG00000088340 | FER1L4 | 0.494997197 | 0.0133517 |
| ENSG00000114209 | PDCD10 | 0.495060759 | 0.000056 |
| ENSG00000141298 | SSH2 | 0.495634326 | 0.002993107 |
| ENSG00000161057 | PSMC2 | 0.495659907 | 0.0000106 |
| ENSG00000106992 | AK1 | 0.496649335 | 0.045192155 |
| ENSG00000138398 | PPIG | 0.496732855 | 0.0000589 |
| ENSG00000131507 | NDFIP1 | 0.496869469 | 0.000160752 |
| ENSG00000101189 | MRGBP | 0.497048848 | 0.0000303 |
| ENSG00000160908 | ZNF394 | 0.497190388 | 0.0000883 |
| ENSG00000156381 | ANKRD9 | 0.497617422 | 0.001456947 |
| ENSG00000141934 | PLPP2 | 0.498065745 | 0.000035 |
| ENSG00000017797 | RALBP1 | 0.49902944 | 0.000000686 |
| ENSG00000206573 | THUMPD3-AS1 | 0.499176411 | 0.001760005 |
| ENSG00000126945 | HNRNPH2 | 0.499188508 | 0.000120512 |
| ENSG00000173457 | PPP1R14B | 0.499304204 | 0.0000312 |
| ENSG00000171425 | ZNF581 | 0.499365039 | 0.00000821 |
| ENSG00000147883 | CDKN2B | 0.499372017 | 0.048698506 |
| ENSG00000134690 | CDCA8 | 0.499399301 | 0.038262944 |
| ENSG00000183648 | NDUFB1 | 0.499454637 | 0.00257113 |
| ENSG00000119899 | SLC17A5 | 0.499461741 | 0.01840035 |
| ENSG00000066651 | TRMT11 | 0.499482491 | 0.002131067 |
| ENSG00000095059 | DHPS | 0.499502087 | 0.000435984 |
| ENSG00000185164 | NOMO2 | 0.499593025 | 0.032347757 |
| ENSG00000176973 | FAM89B | 0.499685159 | 0.031473505 |
| ENSG00000061938 | TNK2 | 0.500429915 | 0.000000657 |
| ENSG00000136856 | SLC2A8 | 0.500647889 | 0.012935772 |
| ENSG00000185088 | RPS27L | 0.500834778 | 0.000989216 |
| ENSG00000165650 | PDZD8 | 0.50085496 | 0.0000312 |
| ENSG00000088833 | NSFL1C | 0.500898807 | 0.00000589 |
| ENSG00000050426 | LETMD1 | 0.501123943 | 0.00000177 |
| ENSG00000090372 | STRN4 | 0.501693468 | 0.000000383 |
| ENSG00000180611 | MB21D2 | 0.501718255 | 0.001556375 |
| ENSG00000104408 | EIF3E | 0.501917822 | 0.000000162 |
| ENSG00000198399 | ITSN2 | 0.501941315 | 0.0000117 |
| ENSG00000245694 | CRNDE | 0.502082518 | 0.000916963 |
| ENSG00000187109 | NAP1L1 | 0.502356699 | 0.000000315 |
| ENSG00000109572 | CLCN3 | 0.502483602 | 3.87E-08 |
| ENSG00000246067 | RAB30-AS1 | 0.502630869 | 0.020994179 |
| ENSG00000041357 | PSMA4 | 0.502647156 | 0.00000546 |
| ENSG00000182903 | ZNF721 | 0.502937134 | 0.00210137 |
| ENSG00000130520 | LSM4 | 0.502987302 | 0.006730757 |
| ENSG00000085449 | WDFY1 | 0.503150305 | 0.000000217 |
| ENSG00000152082 | MZT2B | 0.50324087 | 0.000919713 |
| ENSG00000105053 | VRK3 | 0.503399738 | 0.00052152 |
| ENSG00000182504 | CEP97 | 0.50370413 | 0.033654433 |
| ENSG00000154328 | NEIL2 | 0.50375668 | 0.020028981 |
| ENSG00000130312 | MRPL34 | 0.503961683 | 0.003396578 |
| ENSG00000241839 | PLEKHO2 | 0.504513896 | 0.009371102 |
| ENSG00000165474 | GJB2 | 0.50503966 | 0.000567055 |
| ENSG00000132424 | PNISR | 0.50564858 | 0.0000144 |
| ENSG00000173226 | IQCB1 | 0.505851202 | 0.00000255 |
| ENSG00000145740 | SLC30A5 | 0.505904679 | 0.001702136 |
| ENSG00000213585 | VDAC1 | 0.506144175 | 0.000000028 |
| ENSG00000176095 | IP6K1 | 0.50664089 | 0.0000195 |
| ENSG00000089693 | MLF2 | 0.507597342 | 0.0000165 |
| ENSG00000116729 | WLS | 0.508533311 | 0.0000464 |
| ENSG00000100519 | PSMC6 | 0.508808653 | 0.0000695 |
| ENSG00000158292 | GPR153 | 0.508813593 | 0.001528292 |
| ENSG00000131115 | ZNF227 | 0.509123787 | 0.0000576 |
| ENSG00000105583 | WDR83OS | 0.509259329 | 0.001724943 |
| ENSG00000114857 | NKTR | 0.51001383 | 0.000707938 |
| ENSG00000124802 | EEF1E1 | 0.510374499 | 0.001750385 |
| ENSG00000060491 | OGFR | 0.510598822 | 0.00000278 |
| ENSG00000119411 | BSPRY | 0.51069721 | 0.000407962 |
| ENSG00000013374 | NUB1 | 0.510944626 | 0.000000412 |
| ENSG00000213047 | DENND1B | 0.51101259 | 0.000233258 |
| ENSG00000175087 | PDIK1L | 0.511029495 | 0.006450475 |
| ENSG00000278970 | HEIH | 0.511544135 | 0.001310011 |
| ENSG00000267809 | NDUFV2P1 | 0.512083115 | 0.003596761 |
| ENSG00000167074 | TEF | 0.513100043 | 0.015500216 |
| ENSG00000161091 | MFSD12 | 0.513166551 | 0.003991493 |
| ENSG00000164823 | OSGIN2 | 0.513210738 | 0.000046 |
| ENSG00000176994 | SMCR8 | 0.513524499 | 0.000868286 |
| ENSG00000090097 | PCBP4 | 0.513547693 | 0.003134219 |
| ENSG00000187514 | PTMA | 0.513697817 | 0.000118254 |
| ENSG00000278191 |  | 0.513909504 | 0.001569949 |
| ENSG00000135940 | COX5B | 0.514559284 | 0.0000333 |
| ENSG00000142197 | DOPEY2 | 0.51456902 | 0.0000401 |
| ENSG00000145741 | BTF3 | 0.514772214 | 0.000000714 |
| ENSG00000197024 | ZNF398 | 0.514911838 | 0.002123856 |
| ENSG00000123983 | ACSL3 | 0.514944542 | 3.73E-08 |
| ENSG00000155640 |  | 0.515363209 | 0.0160304 |
| ENSG00000145495 | 44261 | 0.515820676 | 0.000000179 |
| ENSG00000184454 | NCMAP | 0.517443032 | 0.005363547 |
| ENSG00000123643 | SLC36A1 | 0.517627672 | 0.003026199 |
| ENSG00000229638 | RPL4P4 | 0.517672616 | 0.012932334 |
| ENSG00000151348 | EXT2 | 0.517912868 | 4.37E-08 |
| ENSG00000055332 | EIF2AK2 | 0.517920104 | 0.000360071 |
| ENSG00000180992 | MRPL14 | 0.51830339 | 0.002336199 |
| ENSG00000107959 | PITRM1 | 0.518947162 | 0.0000108 |
| ENSG00000155099 | PIP4P2 | 0.519228767 | 0.017533639 |
| ENSG00000130940 | CASZ1 | 0.52014418 | 0.002721014 |
| ENSG00000118518 | RNF146 | 0.520144692 | 0.00367805 |
| ENSG00000138668 | HNRNPD | 0.520206755 | 0.000000849 |
| ENSG00000104960 | PTOV1 | 0.52041425 | 0.0000594 |
| ENSG00000181450 | ZNF678 | 0.520536127 | 0.026926758 |
| ENSG00000113163 | COL4A3BP | 0.520607376 | 0.000000755 |
| ENSG00000168701 | TMEM208 | 0.521279619 | 0.003855962 |
| ENSG00000242071 | RPL7AP6 | 0.521434799 | 0.0000012 |
| ENSG00000126214 | KLC1 | 0.52152648 | 0.000266923 |
| ENSG00000164733 | CTSB | 0.521715347 | 0.000000092 |
| ENSG00000111676 | ATN1 | 0.52202549 | 0.0000434 |
| ENSG00000204628 | RACK1 | 0.52210783 | 7.27E-08 |
| ENSG00000178053 | MLF1 | 0.522199881 | 0.000192158 |
| ENSG00000029993 | HMGB3 | 0.522963957 | 0.000832152 |
| ENSG00000147421 | HMBOX1 | 0.523166529 | 0.030478213 |
| ENSG00000102172 | SMS | 0.523446111 | 0.00000959 |
| ENSG00000171475 | WIPF2 | 0.523520645 | 0.0000148 |
| ENSG00000161021 | MAML1 | 0.524421142 | 0.0000107 |
| ENSG00000141543 | EIF4A3 | 0.5245322 | 0.00000452 |
| ENSG00000119787 | ATL2 | 0.524627573 | 0.001311971 |
| ENSG00000100311 | PDGFB | 0.525012385 | 0.007732292 |
| ENSG00000109390 | NDUFC1 | 0.525146089 | 0.000683289 |
| ENSG00000154380 | ENAH | 0.525463489 | 0.0000674 |
| ENSG00000068383 | INPP5A | 0.525613361 | 0.000672735 |
| ENSG00000182154 | MRPL41 | 0.525699388 | 0.000385861 |
| ENSG00000109320 | NFKB1 | 0.525825113 | 0.000628279 |
| ENSG00000055609 | KMT2C | 0.526313632 | 0.030551604 |
| ENSG00000158089 | GALNT14 | 0.52656808 | 0.000203129 |
| ENSG00000167671 | UBXN6 | 0.526922749 | 0.0000232 |
| ENSG00000121579 | NAA50 | 0.526942795 | 0.00000966 |
| ENSG00000167861 | HID1 | 0.527192355 | 0.000287539 |
| ENSG00000058673 | ZC3H11A | 0.527800517 | 0.0000137 |
| ENSG00000265681 | RPL17 | 0.528138531 | 0.007120742 |
| ENSG00000061676 | NCKAP1 | 0.528432425 | 1.7E-10 |
| ENSG00000142330 | CAPN10 | 0.528458618 | 0.000112155 |
| ENSG00000153310 | FAM49B | 0.528625707 | 0.000000486 |
| ENSG00000117143 | UAP1 | 0.528695707 | 0.000202772 |
| ENSG00000000419 | DPM1 | 0.52879214 | 0.0000428 |
| ENSG00000182117 | NOP10 | 0.529015522 | 0.000142518 |
| ENSG00000111859 | NEDD9 | 0.529020756 | 0.0000485 |
| ENSG00000125249 | RAP2A | 0.529090435 | 0.000395533 |
| ENSG00000232388 | SMIM26 | 0.529241059 | 0.001095921 |
| ENSG00000125844 | RRBP1 | 0.52929665 | 0.000159085 |
| ENSG00000182742 | HOXB4 | 0.529627505 | 0.000374109 |
| ENSG00000120137 | PANK3 | 0.529748104 | 0.000531981 |
| ENSG00000163349 | HIPK1 | 0.529818549 | 0.0000121 |
| ENSG00000280858 |  | 0.529948527 | 0.048275635 |
| ENSG00000084623 | EIF3I | 0.529956619 | 0.0000109 |
| ENSG00000100092 | SH3BP1 | 0.530326695 | 0.000403689 |
| ENSG00000125898 | FAM110A | 0.530449508 | 0.0000552 |
| ENSG00000176105 | YES1 | 0.530749287 | 0.000000152 |
| ENSG00000229117 | RPL41 | 0.530766169 | 0.00000335 |
| ENSG00000004961 | HCCS | 0.530819883 | 0.000130178 |
| ENSG00000100354 | TNRC6B | 0.530945875 | 0.003011405 |
| ENSG00000100410 | PHF5A | 0.530977242 | 0.001555558 |
| ENSG00000167315 | ACAA2 | 0.531132701 | 0.00000022 |
| ENSG00000087087 | SRRT | 0.531511973 | 2.87E-08 |
| ENSG00000197728 | RPS26 | 0.531684334 | 0.00000245 |
| ENSG00000070761 | CFAP20 | 0.531763479 | 0.000792585 |
| ENSG00000151287 | TEX30 | 0.53188494 | 0.019534314 |
| ENSG00000224971 | SUMO2P3 | 0.53237141 | 0.001656319 |
| ENSG00000066322 | ELOVL1 | 0.532878191 | 0.0000107 |
| ENSG00000196505 | GDAP2 | 0.532924608 | 0.000931601 |
| ENSG00000226950 | DANCR | 0.533004801 | 0.000355268 |
| ENSG00000159377 | PSMB4 | 0.533184083 | 0.0000213 |
| ENSG00000008282 | SYPL1 | 0.533222163 | 0.000000567 |
| ENSG00000164117 | FBXO8 | 0.533231129 | 0.001992835 |
| ENSG00000130159 | ECSIT | 0.533330496 | 0.000179657 |
| ENSG00000167283 | ATP5MG | 0.533522629 | 0.000176928 |
| ENSG00000147905 | ZCCHC7 | 0.534216328 | 0.000599872 |
| ENSG00000109911 | ELP4 | 0.534651792 | 0.000215595 |
| ENSG00000184220 | CMSS1 | 0.534692153 | 0.000542906 |
| ENSG00000100804 | PSMB5 | 0.534704844 | 0.000103362 |
| ENSG00000189171 | S100A13 | 0.534975852 | 0.004997772 |
| ENSG00000159256 | MORC3 | 0.535059118 | 0.00000258 |
| ENSG00000133818 | RRAS2 | 0.535187936 | 0.000000161 |
| ENSG00000153914 | SREK1 | 0.53531415 | 0.0000617 |
| ENSG00000142871 | CYR61 | 0.536098476 | 0.040037841 |
| ENSG00000104635 | SLC39A14 | 0.536259291 | 0.0000516 |
| ENSG00000108819 | PPP1R9B | 0.536443385 | 0.00000208 |
| ENSG00000073008 | PVR | 0.536498903 | 0.00065489 |
| ENSG00000112305 | SMAP1 | 0.536912428 | 0.000435984 |
| ENSG00000114742 | WDR48 | 0.536964485 | 0.00000784 |
| ENSG00000158417 | EIF5B | 0.537004435 | 0.000455579 |
| ENSG00000133398 | MED10 | 0.537081668 | 0.000138495 |
| ENSG00000117139 | KDM5B | 0.537231395 | 0.001297695 |
| ENSG00000175334 | BANF1 | 0.537307015 | 0.000493167 |
| ENSG00000106346 | USP42 | 0.537430544 | 0.000140348 |
| ENSG00000108468 | CBX1 | 0.537443517 | 0.004148044 |
| ENSG00000204524 | ZNF805 | 0.537663768 | 0.022186008 |
| ENSG00000076826 | CAMSAP3 | 0.537745737 | 0.00000258 |
| ENSG00000070010 | UFD1 | 0.537960349 | 0.000530498 |
| ENSG00000150527 | CTAGE5 | 0.538136557 | 0.03715416 |
| ENSG00000197258 | EIF4BP6 | 0.538211342 | 0.008313705 |
| ENSG00000188612 | SUMO2 | 0.538695744 | 0.0000706 |
| ENSG00000148362 | PAXX | 0.53888696 | 0.000776072 |
| ENSG00000254087 | LYN | 0.539004567 | 0.02093929 |
| ENSG00000166803 | PCLAF | 0.539089893 | 0.035493042 |
| ENSG00000125089 | SH3TC1 | 0.539274927 | 0.004466477 |
| ENSG00000146247 | PHIP | 0.539285391 | 0.0000608 |
| ENSG00000169230 | PRELID1 | 0.539705053 | 0.00015699 |
| ENSG00000142541 | RPL13A | 0.539706135 | 0.000000437 |
| ENSG00000089157 | RPLP0 | 0.539851286 | 0.000000831 |
| ENSG00000167778 | SPRYD3 | 0.539928567 | 0.000129715 |
| ENSG00000026103 | FAS | 0.539936182 | 0.041191884 |
| ENSG00000148950 | IMMP1L | 0.540412807 | 0.000749892 |
| ENSG00000172428 | COPS9 | 0.540480822 | 0.002144553 |
| ENSG00000062716 | VMP1 | 0.5405597 | 0.000043 |
| ENSG00000172757 | CFL1 | 0.541056887 | 0.0000181 |
| ENSG00000213553 | RPLP0P6 | 0.54115809 | 0.000107987 |
| ENSG00000105401 | CDC37 | 0.541399307 | 1.37E-08 |
| ENSG00000268205 | AC005261.1 | 0.541698536 | 0.012424942 |
| ENSG00000197375 | SLC22A5 | 0.541773114 | 0.0000981 |
| ENSG00000143674 | MAP3K21 | 0.542612005 | 0.000160544 |
| ENSG00000115457 | IGFBP2 | 0.542985558 | 0.001281237 |
| ENSG00000170634 | ACYP2 | 0.543026926 | 0.001250237 |
| ENSG00000065600 | TMEM206 | 0.543572068 | 0.00322147 |
| ENSG00000196792 | STRN3 | 0.543575983 | 0.0000224 |
| ENSG00000145494 | NDUFS6 | 0.543647214 | 0.000411924 |
| ENSG00000100997 | ABHD12 | 0.543903914 | 0.0000013 |
| ENSG00000178307 | TMEM11 | 0.543951987 | 0.002306128 |
| ENSG00000175581 | MRPL48 | 0.544362193 | 0.0000207 |
| ENSG00000173933 | RBM4 | 0.544454997 | 0.001523101 |
| ENSG00000141522 | ARHGDIA | 0.544738108 | 0.000000164 |
| ENSG00000148120 | C9orf3 | 0.545006693 | 0.001389022 |
| ENSG00000005700 | IBTK | 0.545110991 | 3.81E-08 |
| ENSG00000198799 | LRIG2 | 0.545122445 | 0.000147128 |
| ENSG00000105355 | PLIN3 | 0.545576899 | 0.00000551 |
| ENSG00000082701 | GSK3B | 0.545922748 | 0.000000227 |
| ENSG00000006451 | RALA | 0.546521588 | 0.00000152 |
| ENSG00000137414 | FAM8A1 | 0.546948654 | 0.000287539 |
| ENSG00000107290 | SETX | 0.54716413 | 0.0000041 |
| ENSG00000124226 | RNF114 | 0.547463533 | 0.00000507 |
| ENSG00000160199 | PKNOX1 | 0.547587156 | 0.00000329 |
| ENSG00000181885 | CLDN7 | 0.547877543 | 0.0000845 |
| ENSG00000104907 | TRMT1 | 0.548171476 | 0.000015 |
| ENSG00000137970 | RPL7P9 | 0.548258764 | 0.001174243 |
| ENSG00000147649 | MTDH | 0.548627406 | 1.58E-10 |
| ENSG00000117133 | RPF1 | 0.549494925 | 0.00000914 |
| ENSG00000127554 | GFER | 0.549692574 | 0.000168782 |
| ENSG00000139793 | MBNL2 | 0.549938298 | 0.000413159 |
| ENSG00000071564 | TCF3 | 0.550370573 | 8.14E-09 |
| ENSG00000136811 | ODF2 | 0.550373968 | 0.0000044 |
| ENSG00000070756 | PABPC1 | 0.550807785 | 1.04E-10 |
| ENSG00000127184 | COX7C | 0.551206208 | 0.0000105 |
| ENSG00000170734 | POLH | 0.551776168 | 0.038670742 |
| ENSG00000261061 | AC092718.4 | 0.551888873 | 0.025328864 |
| ENSG00000008441 | NFIX | 0.552055896 | 0.0000103 |
| ENSG00000141499 | WRAP53 | 0.552256837 | 0.013222074 |
| ENSG00000064601 | CTSA | 0.552455537 | 0.000000119 |
| ENSG00000189343 | RPS2P46 | 0.552545766 | 0.000505215 |
| ENSG00000106682 | EIF4H | 0.552554862 | 2.18E-08 |
| ENSG00000127311 | HELB | 0.552844459 | 0.010153759 |
| ENSG00000234797 | RPS3AP6 | 0.552911842 | 0.019669662 |
| ENSG00000244313 | AC024293.1 | 0.553102009 | 0.015819318 |
| ENSG00000163624 | CDS1 | 0.553241416 | 0.0000038 |
| ENSG00000167977 | KCTD5 | 0.553340593 | 0.0000299 |
| ENSG00000112640 | PPP2R5D | 0.553620312 | 0.000403156 |
| ENSG00000140262 | TCF12 | 0.553730152 | 2.48E-08 |
| ENSG00000151532 | VTI1A | 0.553766491 | 0.000000312 |
| ENSG00000070444 | MNT | 0.553810408 | 0.0000278 |
| ENSG00000123374 | CDK2 | 0.553882145 | 0.004142506 |
| ENSG00000150687 | PRSS23 | 0.554005945 | 0.003647881 |
| ENSG00000220937 | HNRNPA1P41 | 0.55408485 | 0.04873105 |
| ENSG00000100316 | RPL3 | 0.554479656 | 4.62E-09 |
| ENSG00000167962 | ZNF598 | 0.554642403 | 0.000407029 |
| ENSG00000174718 | KIAA1551 | 0.554698362 | 0.000012 |
| ENSG00000176871 | WSB2 | 0.554904368 | 0.000108522 |
| ENSG00000161800 | RACGAP1 | 0.555317425 | 0.001539764 |
| ENSG00000138413 | IDH1 | 0.555396598 | 0.0000103 |
| ENSG00000152117 | AC073869.1 | 0.556020728 | 0.000858667 |
| ENSG00000172590 | MRPL52 | 0.556133605 | 0.0000415 |
| ENSG00000213551 | DNAJC9 | 0.556252466 | 0.000975597 |
| ENSG00000122042 | UBL3 | 0.556605991 | 0.00021469 |
| ENSG00000137693 | YAP1 | 0.557086603 | 8.39E-09 |
| ENSG00000118705 | RPN2 | 0.557299684 | 1.01E-09 |
| ENSG00000116786 | PLEKHM2 | 0.557640534 | 0.000000843 |
| ENSG00000101049 | SGK2 | 0.557909966 | 0.000227337 |
| ENSG00000157985 | AGAP1 | 0.558134114 | 0.0000236 |
| ENSG00000151292 | CSNK1G3 | 0.558239527 | 0.000000924 |
| ENSG00000120948 | TARDBP | 0.558547381 | 0.00000148 |
| ENSG00000135486 | HNRNPA1 | 0.55905345 | 1.35E-09 |
| ENSG00000230844 | ZNF674-AS1 | 0.55914233 | 0.039556914 |
| ENSG00000196911 | KPNA5 | 0.559346027 | 0.001589295 |
| ENSG00000177380 | PPFIA3 | 0.559678342 | 0.000350642 |
| ENSG00000198925 | ATG9A | 0.559751548 | 0.0000132 |
| ENSG00000232112 | TMA7 | 0.559906665 | 0.00021203 |
| ENSG00000163950 | SLBP | 0.560133102 | 0.002423221 |
| ENSG00000081913 | PHLPP1 | 0.560206562 | 0.0000757 |
| ENSG00000100227 | POLDIP3 | 0.560375217 | 0.000000933 |
| ENSG00000110048 | OSBP | 0.560490233 | 0.0000728 |
| ENSG00000235823 | OLMALINC | 0.56145348 | 0.004466488 |
| ENSG00000139117 | CPNE8 | 0.561710268 | 0.005880075 |
| ENSG00000007080 | CCDC124 | 0.561760027 | 0.0000196 |
| ENSG00000210082 | MT-RNR2 | 0.561803894 | 0.001258823 |
| ENSG00000121931 | LRIF1 | 0.561985983 | 0.000113864 |
| ENSG00000145293 | ENOPH1 | 0.562401229 | 0.00000311 |
| ENSG00000120727 | PAIP2 | 0.562632542 | 0.00000209 |
| ENSG00000118217 | ATF6 | 0.562711795 | 8.08E-08 |
| ENSG00000130332 | LSM7 | 0.562945186 | 0.000111785 |
| ENSG00000164258 | NDUFS4 | 0.563453717 | 0.000000646 |
| ENSG00000141580 | WDR45B | 0.563824516 | 0.000000059 |
| ENSG00000139641 | ESYT1 | 0.564211257 | 9.09E-08 |
| ENSG00000182768 | NGRN | 0.564741624 | 0.00000451 |
| ENSG00000120690 | ELF1 | 0.565104059 | 0.00000109 |
| ENSG00000185049 | NELFA | 0.565122976 | 0.0000458 |
| ENSG00000185658 | BRWD1 | 0.565270145 | 0.0000115 |
| ENSG00000164620 | RELL2 | 0.565383184 | 0.003863832 |
| ENSG00000143621 | ILF2 | 0.565663493 | 0.000422061 |
| ENSG00000204152 | TIMM23B | 0.565765473 | 0.002679086 |
| ENSG00000279483 | AC090498.1 | 0.565777767 | 0.003453226 |
| ENSG00000107745 | MICU1 | 0.565867739 | 0.00000049 |
| ENSG00000177666 | PNPLA2 | 0.565940001 | 6.88E-09 |
| ENSG00000166974 | MAPRE2 | 0.566059442 | 0.00059915 |
| ENSG00000144395 | CCDC150 | 0.566075985 | 0.029229005 |
| ENSG00000156873 | PHKG2 | 0.56620278 | 0.002022221 |
| ENSG00000197102 | DYNC1H1 | 0.566639469 | 0.014778807 |
| ENSG00000087365 | SF3B2 | 0.566862654 | 0.00000357 |
| ENSG00000168264 | IRF2BP2 | 0.567049971 | 0.000000243 |
| ENSG00000115652 | UXS1 | 0.56705967 | 5.54E-08 |
| ENSG00000068308 | OTUD5 | 0.567225505 | 0.00000002 |
| ENSG00000169018 | FEM1B | 0.567323686 | 0.000000419 |
| ENSG00000143106 | PSMA5 | 0.567736527 | 0.0000069 |
| ENSG00000198034 | RPS4X | 0.56841789 | 1.24E-08 |
| ENSG00000276345 | AC004556.1 | 0.56860424 | 0.000725004 |
| ENSG00000108510 | MED13 | 0.569088645 | 0.000371254 |
| ENSG00000143393 | PI4KB | 0.569133776 | 0.000000026 |
| ENSG00000242083 | RPL7AP31 | 0.569705314 | 0.010611988 |
| ENSG00000110497 | AMBRA1 | 0.570273937 | 1.19E-08 |
| ENSG00000141441 | GAREM1 | 0.570513689 | 0.000264513 |
| ENSG00000168275 | COA6 | 0.570905785 | 0.000850613 |
| ENSG00000173153 | ESRRA | 0.571588456 | 0.000000454 |
| ENSG00000111275 | ALDH2 | 0.571974743 | 0.000000305 |
| ENSG00000197381 | ADARB1 | 0.572062509 | 0.000817226 |
| ENSG00000247516 | MIR4458HG | 0.572726588 | 0.001444615 |
| ENSG00000130559 | CAMSAP1 | 0.572776535 | 0.000000118 |
| ENSG00000013810 | TACC3 | 0.572803117 | 0.004135733 |
| ENSG00000184979 | USP18 | 0.573761951 | 0.011501157 |
| ENSG00000114933 | INO80D | 0.574192226 | 0.000733289 |
| ENSG00000244405 | ETV5 | 0.57427058 | 0.00000418 |
| ENSG00000162191 | UBXN1 | 0.574304911 | 7.81E-09 |
| ENSG00000182197 | EXT1 | 0.574724113 | 0.000000289 |
| ENSG00000184669 | OR7E14P | 0.575067503 | 0.000919967 |
| ENSG00000186951 | PPARA | 0.57579527 | 0.00000751 |
| ENSG00000130741 | EIF2S3 | 0.575922211 | 4.08E-08 |
| ENSG00000089060 | SLC8B1 | 0.576080797 | 0.018664756 |
| ENSG00000163900 | TMEM41A | 0.577009743 | 0.000000474 |
| ENSG00000167578 | RAB4B | 0.577394797 | 0.003769537 |
| ENSG00000160799 | CCDC12 | 0.577481326 | 0.00000859 |
| ENSG00000182087 | TMEM259 | 0.57829262 | 0.000027 |
| ENSG00000164919 | COX6C | 0.578368289 | 0.000213817 |
| ENSG00000224631 | RPS27AP16 | 0.578589403 | 0.002619208 |
| ENSG00000144567 | RETREG2 | 0.578816523 | 0.0000334 |
| ENSG00000158828 | PINK1 | 0.579099172 | 0.000108488 |
| ENSG00000142507 | PSMB6 | 0.579150542 | 0.00014242 |
| ENSG00000156030 | ELMSAN1 | 0.579264814 | 0.000118338 |
| ENSG00000128607 | KLHDC10 | 0.579696538 | 5.82E-10 |
| ENSG00000174444 | RPL4 | 0.579718995 | 7.72E-11 |
| ENSG00000117859 | OSBPL9 | 0.579721115 | 6.63E-09 |
| ENSG00000177565 | TBL1XR1 | 0.580296458 | 1.47E-09 |
| ENSG00000183963 | SMTN | 0.580801068 | 0.0000444 |
| ENSG00000182957 | SPATA13 | 0.580967706 | 0.0000765 |
| ENSG00000170004 | CHD3 | 0.581295687 | 0.0000178 |
| ENSG00000181704 | YIPF6 | 0.581437045 | 0.000424578 |
| ENSG00000177879 | AP3S1 | 0.581602213 | 0.0000007 |
| ENSG00000171169 | NAIF1 | 0.581663252 | 0.0000169 |
| ENSG00000164924 | YWHAZ | 0.581938697 | 2.94E-10 |
| ENSG00000102034 | ELF4 | 0.581956262 | 1.47E-08 |
| ENSG00000059769 | DNAJC25 | 0.581972738 | 0.002594518 |
| ENSG00000276180 | HIST1H4I | 0.582268098 | 0.020112876 |
| ENSG00000151491 | EPS8 | 0.582308249 | 2.04E-09 |
| ENSG00000196470 | SIAH1 | 0.582595099 | 0.0000168 |
| ENSG00000054267 | ARID4B | 0.582613443 | 0.0000959 |
| ENSG00000122406 | RPL5 | 0.582910554 | 9.64E-09 |
| ENSG00000062485 | CS | 0.583070152 | 3.04E-09 |
| ENSG00000034677 | RNF19A | 0.583131364 | 0.0000119 |
| ENSG00000139697 | SBNO1 | 0.583341064 | 0.000000413 |
| ENSG00000126067 | PSMB2 | 0.584649284 | 0.00000448 |
| ENSG00000125356 | NDUFA1 | 0.584653728 | 0.000041 |
| ENSG00000126768 | TIMM17B | 0.585083648 | 0.0000121 |
| ENSG00000181751 | C5orf30 | 0.585508128 | 0.00000664 |
| ENSG00000100263 | RHBDD3 | 0.585607163 | 0.0000117 |
| ENSG00000221823 | PPP3R1 | 0.585769548 | 0.000000224 |
| ENSG00000119844 | AFTPH | 0.586216949 | 1.99E-09 |
| ENSG00000052795 | FNIP2 | 0.586446789 | 0.0000136 |
| ENSG00000175826 | CTDNEP1 | 0.586505082 | 0.000000013 |
| ENSG00000091490 | SEL1L3 | 0.586825191 | 0.000000486 |
| ENSG00000004975 | DVL2 | 0.587302886 | 0.001040741 |
| ENSG00000169894 | MUC3A | 0.587318661 | 0.002075681 |
| ENSG00000253785 | AC008429.2 | 0.58743628 | 0.015028058 |
| ENSG00000108578 | BLMH | 0.587489482 | 0.013819385 |
| ENSG00000091527 | CDV3 | 0.587506478 | 0.00000143 |
| ENSG00000160783 | PMF1 | 0.587546652 | 0.001079411 |
| ENSG00000100558 | PLEK2 | 0.587768319 | 0.000494344 |
| ENSG00000167460 | TPM4 | 0.587983447 | 1.04E-10 |
| ENSG00000263429 | TMEM238L | 0.58804634 | 0.002729203 |
| ENSG00000130522 | JUND | 0.588241596 | 0.000621221 |
| ENSG00000166557 | TMED3 | 0.588316505 | 0.000451344 |
| ENSG00000104979 | C19orf53 | 0.588340676 | 0.0000165 |
| ENSG00000129810 | SGO1 | 0.588353888 | 0.00587112 |
| ENSG00000196683 | TOMM7 | 0.588358505 | 0.00000158 |
| ENSG00000100883 | SRP54 | 0.588416586 | 0.000126026 |
| ENSG00000165476 | REEP3 | 0.588467626 | 0.000000338 |
| ENSG00000168078 | PBK | 0.588563824 | 0.031235475 |
| ENSG00000135002 | RFK | 0.588726893 | 0.00000608 |
| ENSG00000149564 | ESAM | 0.589043539 | 0.000935602 |
| ENSG00000198755 | RPL10A | 0.589131955 | 4.11E-09 |
| ENSG00000104852 | SNRNP70 | 0.589142726 | 0.0000895 |
| ENSG00000168036 | CTNNB1 | 0.590446639 | 1.72E-10 |
| ENSG00000273015 | AC008124.1 | 0.590610026 | 0.0000346 |
| ENSG00000151151 | IPMK | 0.590897705 | 0.000365448 |
| ENSG00000072201 | LNX1 | 0.59104031 | 0.000137993 |
| ENSG00000164603 | BMT2 | 0.59164377 | 0.0000136 |
| ENSG00000198873 | GRK5 | 0.59213978 | 0.000460134 |
| ENSG00000173193 | PARP14 | 0.592294018 | 0.000380109 |
| ENSG00000135249 | RINT1 | 0.592361616 | 0.002030976 |
| ENSG00000124067 | SLC12A4 | 0.59238297 | 0.006784568 |
| ENSG00000122126 | OCRL | 0.592728364 | 0.00003 |
| ENSG00000172939 | OXSR1 | 0.593169967 | 0.000000219 |
| ENSG00000109917 | ZPR1 | 0.593287968 | 0.00000121 |
| ENSG00000163374 | YY1AP1 | 0.593388931 | 0.00000947 |
| ENSG00000162613 | FUBP1 | 0.593402665 | 0.000000126 |
| ENSG00000129562 | DAD1 | 0.593571298 | 0.000375302 |
| ENSG00000269335 | IKBKG | 0.593578355 | 0.000000707 |
| ENSG00000160271 | RALGDS | 0.59379856 | 9.2E-09 |
| ENSG00000163902 | RPN1 | 0.593817573 | 0.000560153 |
| ENSG00000142409 | ZNF787 | 0.593875622 | 0.000000175 |
| ENSG00000121753 | ADGRB2 | 0.594017125 | 0.043683601 |
| ENSG00000165632 | TAF3 | 0.594330537 | 0.0000015 |
| ENSG00000144591 | GMPPA | 0.594453639 | 0.0000186 |
| ENSG00000175906 | ARL4D | 0.594818227 | 0.021448777 |
| ENSG00000170291 | ELP5 | 0.594822707 | 0.00017561 |
| ENSG00000131508 | UBE2D2 | 0.595426004 | 0.00000251 |
| ENSG00000140750 | ARHGAP17 | 0.595443844 | 0.0000117 |
| ENSG00000214756 | CSKMT | 0.595457832 | 0.004116214 |
| ENSG00000092199 | HNRNPC | 0.595498386 | 0.000000125 |
| ENSG00000218336 | TENM3 | 0.595591811 | 0.015497419 |
| ENSG00000108671 | PSMD11 | 0.595652989 | 0.00000376 |
| ENSG00000144909 | OSBPL11 | 0.596290959 | 0.0000127 |
| ENSG00000173273 | TNKS | 0.596479506 | 0.000390536 |
| ENSG00000183283 | DAZAP2 | 0.596655688 | 0.00000132 |
| ENSG00000088002 | SULT2B1 | 0.596766975 | 0.000191628 |
| ENSG00000114125 | RNF7 | 0.596785043 | 0.000000632 |
| ENSG00000088356 | PDRG1 | 0.597158551 | 0.00000173 |
| ENSG00000105866 | SP4 | 0.59768502 | 0.000118831 |
| ENSG00000124172 | ATP5F1E | 0.597798229 | 0.00000619 |
| ENSG00000073803 | MAP3K13 | 0.59815471 | 0.000000133 |
| ENSG00000147383 | NSDHL | 0.598305182 | 0.001304739 |
| ENSG00000141905 | NFIC | 0.59841829 | 0.0000117 |
| ENSG00000159335 | PTMS | 0.598548226 | 0.000000101 |
| ENSG00000141664 | ZCCHC2 | 0.599200522 | 0.000110447 |
| ENSG00000259781 | HMGB1P6 | 0.599441471 | 0.000633037 |
| ENSG00000158050 | DUSP2 | 0.599678125 | 0.000911814 |
| ENSG00000159082 | SYNJ1 | 0.599871857 | 0.005507672 |
| ENSG00000159399 | HK2 | 0.599890772 | 0.000860338 |
| ENSG00000225178 | RPSAP58 | 0.600167042 | 0.000244211 |
| ENSG00000145365 | TIFA | 0.600716485 | 0.001409627 |
| ENSG00000156860 | FBRS | 0.6009699 | 0.000000952 |
| ENSG00000174579 | MSL2 | 0.601026994 | 0.000000176 |
| ENSG00000114767 | RRP9 | 0.601434265 | 5.25E-08 |
| ENSG00000157456 | CCNB2 | 0.601733136 | 0.000275154 |
| ENSG00000166925 | TSC22D4 | 0.601754657 | 1.75E-09 |
| ENSG00000117614 | SYF2 | 0.602000084 | 2.3E-10 |
| ENSG00000171150 | SOCS5 | 0.602180688 | 6.58E-09 |
| ENSG00000165802 | NSMF | 0.602426393 | 5.69E-10 |
| ENSG00000107863 | ARHGAP21 | 0.602437938 | 0.0000109 |
| ENSG00000196531 | NACA | 0.602482012 | 2.15E-10 |
| ENSG00000172354 | GNB2 | 0.602693615 | 0.00000288 |
| ENSG00000107771 | CCSER2 | 0.60278675 | 0.000000215 |
| ENSG00000154146 | NRGN | 0.602809504 | 0.0000115 |
| ENSG00000153774 | CFDP1 | 0.603340997 | 0.0000125 |
| ENSG00000063046 | EIF4B | 0.603362518 | 1.48E-08 |
| ENSG00000165637 | VDAC2 | 0.603366736 | 0.000000701 |
| ENSG00000147676 | MAL2 | 0.603407259 | 0.0000016 |
| ENSG00000100908 | EMC9 | 0.604225184 | 0.002778727 |
| ENSG00000156795 | WDYHV1 | 0.604396907 | 0.0000038 |
| ENSG00000214182 | PTMAP5 | 0.604655238 | 0.005037485 |
| ENSG00000063169 | BICRA | 0.604745551 | 0.00055731 |
| ENSG00000197958 | RPL12 | 0.605055232 | 2.84E-10 |
| ENSG00000136943 | CTSV | 0.605094224 | 0.000654873 |
| ENSG00000132967 | HMGB1P5 | 0.605169754 | 0.002107429 |
| ENSG00000103404 | USP31 | 0.605275468 | 0.00000364 |
| ENSG00000105185 | PDCD5 | 0.605769942 | 0.000022 |
| ENSG00000065618 | COL17A1 | 0.605961641 | 0.00000638 |
| ENSG00000169100 | SLC25A6 | 0.60645772 | 7.9E-09 |
| ENSG00000143641 | GALNT2 | 0.606683828 | 2.77E-11 |
| ENSG00000184012 | TMPRSS2 | 0.606796673 | 0.00000504 |
| ENSG00000175866 | BAIAP2 | 0.60686099 | 0.00000057 |
| ENSG00000244687 | UBE2V1 | 0.606865938 | 0.030096523 |
| ENSG00000196449 | YRDC | 0.606892905 | 0.000000159 |
| ENSG00000175354 | PTPN2 | 0.606971125 | 6.05E-08 |
| ENSG00000156970 | BUB1B | 0.607068319 | 0.009243091 |
| ENSG00000130675 | MNX1 | 0.607097232 | 0.003116826 |
| ENSG00000167513 | CDT1 | 0.607182414 | 0.007474178 |
| ENSG00000131174 | COX7B | 0.607278871 | 0.001880397 |
| ENSG00000241878 | PISD | 0.607446501 | 0.0000612 |
| ENSG00000254858 | MPV17L2 | 0.607529591 | 0.008310823 |
| ENSG00000169217 | CD2BP2 | 0.60768446 | 2.34E-10 |
| ENSG00000263266 | RPS7P1 | 0.607953053 | 0.001687392 |
| ENSG00000123728 | RAP2C | 0.608125589 | 0.000000058 |
| ENSG00000130787 | HIP1R | 0.608254472 | 8.85E-10 |
| ENSG00000159202 | UBE2Z | 0.608595457 | 0.000000125 |
| ENSG00000168288 | MMADHC | 0.608673034 | 1.05E-09 |
| ENSG00000168159 | RNF187 | 0.608839828 | 3.21E-08 |
| ENSG00000080802 | CNOT4 | 0.608867067 | 0.00000674 |
| ENSG00000153147 | SMARCA5 | 0.609201949 | 4.13E-10 |
| ENSG00000197857 | ZNF44 | 0.609281092 | 0.000647457 |
| ENSG00000144848 | ATG3 | 0.609281415 | 0.000000315 |
| ENSG00000184990 | SIVA1 | 0.609526615 | 0.00540359 |
| ENSG00000119718 | EIF2B2 | 0.609566911 | 0.0000728 |
| ENSG00000171863 | RPS7 | 0.609691114 | 0.00000215 |
| ENSG00000099341 | PSMD8 | 0.610137027 | 0.0000285 |
| ENSG00000164032 | H2AFZ | 0.610148154 | 0.001037331 |
| ENSG00000197249 | SERPINA1 | 0.610246898 | 0.0000012 |
| ENSG00000214026 | MRPL23 | 0.610292595 | 0.005472235 |
| ENSG00000198901 | PRC1 | 0.610819037 | 0.001116248 |
| ENSG00000251022 | THAP9-AS1 | 0.611247303 | 0.00000513 |
| ENSG00000115425 | PECR | 0.611492353 | 0.000281682 |
| ENSG00000169679 | BUB1 | 0.611772242 | 0.018504598 |
| ENSG00000138180 | CEP55 | 0.611782792 | 0.000163346 |
| ENSG00000143537 | ADAM15 | 0.611850106 | 0.000000202 |
| ENSG00000164346 | NSA2 | 0.611895936 | 0.000000406 |
| ENSG00000246705 | H2AFJ | 0.612309128 | 0.000169395 |
| ENSG00000163479 | SSR2 | 0.612349833 | 0.000000418 |
| ENSG00000137876 | RSL24D1 | 0.612520241 | 4.75E-08 |
| ENSG00000107263 | RAPGEF1 | 0.612564842 | 0.000000336 |
| ENSG00000132254 | ARFIP2 | 0.612806466 | 0.00017965 |
| ENSG00000134531 | EMP1 | 0.612863171 | 0.001232632 |
| ENSG00000087903 | RFX2 | 0.613113863 | 0.000276841 |
| ENSG00000139496 | NUP58 | 0.613364744 | 0.00000126 |
| ENSG00000166575 | TMEM135 | 0.613579968 | 0.000000755 |
| ENSG00000183337 | BCOR | 0.614274903 | 0.0000196 |
| ENSG00000115275 | MOGS | 0.614462614 | 0.00000225 |
| ENSG00000112695 | COX7A2 | 0.614704954 | 0.0000762 |
| ENSG00000074800 | ENO1 | 0.614855778 | 0.0000029 |
| ENSG00000164587 | RPS14 | 0.615234036 | 1.62E-08 |
| ENSG00000100319 | ZMAT5 | 0.615371465 | 0.00047912 |
| ENSG00000079739 | PGM1 | 0.615400184 | 0.000568088 |
| ENSG00000181894 | ZNF329 | 0.61554079 | 0.001718065 |
| ENSG00000225031 | EIF4BP7 | 0.615992122 | 0.004087331 |
| ENSG00000197111 | PCBP2 | 0.61616423 | 3.49E-11 |
| ENSG00000167987 | VPS37C | 0.61629194 | 3.37E-10 |
| ENSG00000129696 | TTI2 | 0.616398104 | 0.003780253 |
| ENSG00000101871 | MID1 | 0.616604719 | 0.007476143 |
| ENSG00000282458 | WASH5P | 0.616645097 | 0.007280868 |
| ENSG00000076924 | XAB2 | 0.616988594 | 0.0000485 |
| ENSG00000176731 | C8orf59 | 0.617120707 | 0.0000954 |
| ENSG00000113328 | CCNG1 | 0.617310054 | 0.00000523 |
| ENSG00000121104 | FAM117A | 0.618370024 | 0.011569367 |
| ENSG00000009954 | BAZ1B | 0.618385288 | 6.75E-09 |
| ENSG00000108774 | RAB5C | 0.618853222 | 0.00000012 |
| ENSG00000115677 | HDLBP | 0.619285465 | 0.00000146 |
| ENSG00000131941 | RHPN2 | 0.619456876 | 9.85E-08 |
| ENSG00000156467 | UQCRB | 0.620081196 | 0.000000387 |
| ENSG00000166347 | CYB5A | 0.620435098 | 0.000000128 |
| ENSG00000100300 | TSPO | 0.620443661 | 0.00000242 |
| ENSG00000128944 | KNSTRN | 0.620619915 | 0.00268119 |
| ENSG00000134352 | IL6ST | 0.62120837 | 1.73E-11 |
| ENSG00000168273 | SMIM4 | 0.622318287 | 0.00507547 |
| ENSG00000069424 | KCNAB2 | 0.622620361 | 0.000103949 |
| ENSG00000168028 | RPSA | 0.622669559 | 0.00000899 |
| ENSG00000089006 | SNX5 | 0.622794963 | 6.27E-09 |
| ENSG00000103145 | HCFC1R1 | 0.622966998 | 0.000402239 |
| ENSG00000281766 |  | 0.623099982 | 0.002313983 |
| ENSG00000100811 | YY1 | 0.623112793 | 3.19E-10 |
| ENSG00000167081 | PBX3 | 0.623234398 | 0.002888174 |
| ENSG00000119820 | YIPF4 | 0.623352444 | 1.96E-11 |
| ENSG00000106245 | BUD31 | 0.623523815 | 0.0000522 |
| ENSG00000198242 | RPL23A | 0.623670833 | 3.35E-08 |
| ENSG00000004700 | RECQL | 0.623754522 | 0.000650545 |
| ENSG00000100911 | PSME2 | 0.623964718 | 0.000000348 |
| ENSG00000162244 | RPL29 | 0.624447076 | 0.000000861 |
| ENSG00000174567 | GOLT1A | 0.624665951 | 0.000180863 |
| ENSG00000198258 | UBL5 | 0.624793346 | 0.0000186 |
| ENSG00000184752 | NDUFA12 | 0.624838767 | 0.00000521 |
| ENSG00000166200 | COPS2 | 0.624951501 | 1.57E-08 |
| ENSG00000174010 | KLHL15 | 0.625922361 | 0.000507692 |
| ENSG00000118260 | CREB1 | 0.625968871 | 1.64E-09 |
| ENSG00000157741 | UBN2 | 0.625979444 | 0.0000423 |
| ENSG00000131759 | RARA | 0.625983319 | 0.000150935 |
| ENSG00000105372 | RPS19 | 0.626587356 | 3.25E-08 |
| ENSG00000006125 | AP2B1 | 0.626636676 | 2.3E-09 |
| ENSG00000175895 | PLEKHF2 | 0.626686973 | 0.00000121 |
| ENSG00000082269 | FAM135A | 0.626885101 | 0.00000251 |
| ENSG00000227097 | RPS28P7 | 0.627053319 | 0.00000344 |
| ENSG00000110697 | PITPNM1 | 0.627286369 | 3.05E-11 |
| ENSG00000236552 | RPL13AP5 | 0.627304629 | 0.00002 |
| ENSG00000115762 | PLEKHB2 | 0.627322041 | 0.00000156 |
| ENSG00000140471 | LINS1 | 0.627422472 | 0.00000219 |
| ENSG00000160124 | CCDC58 | 0.627553121 | 0.0000254 |
| ENSG00000153250 | RBMS1 | 0.628318862 | 8.11E-08 |
| ENSG00000128789 | PSMG2 | 0.628351358 | 0.000113544 |
| ENSG00000184432 | COPB2 | 0.62854157 | 0.00000684 |
| ENSG00000108582 | CPD | 0.62887189 | 0.000000242 |
| ENSG00000091583 | APOH | 0.628884305 | 0.00000068 |
| ENSG00000090061 | CCNK | 0.628966965 | 0.000207344 |
| ENSG00000110090 | CPT1A | 0.62899773 | 1.96E-08 |
| ENSG00000168143 | FAM83B | 0.629146975 | 0.000503776 |
| ENSG00000176225 | RTTN | 0.629263332 | 0.002779975 |
| ENSG00000101132 | PFDN4 | 0.629439216 | 0.00000529 |
| ENSG00000115233 | PSMD14 | 0.629624445 | 0.00000104 |
| ENSG00000118369 | USP35 | 0.629815744 | 0.000114414 |
| ENSG00000073792 | IGF2BP2 | 0.629941435 | 2.14E-08 |
| ENSG00000196961 | AP2A1 | 0.630550872 | 0.0000314 |
| ENSG00000227656 |  | 0.630692859 | 0.0000195 |
| ENSG00000101444 | AHCY | 0.630742475 | 0.0000012 |
| ENSG00000075218 | GTSE1 | 0.631161762 | 0.001599735 |
| ENSG00000108511 | HOXB6 | 0.631167889 | 0.0000287 |
| ENSG00000089685 | BIRC5 | 0.631699715 | 0.007472833 |
| ENSG00000147548 | NSD3 | 0.631962305 | 0.00000304 |
| ENSG00000112996 | MRPS30 | 0.632378026 | 0.000000108 |
| ENSG00000126432 | PRDX5 | 0.63279319 | 2.36E-11 |
| ENSG00000185019 | UBOX5 | 0.632884292 | 0.0000289 |
| ENSG00000242445 | RPL7AP11 | 0.6328981 | 0.000473172 |
| ENSG00000132964 | CDK8 | 0.633027568 | 0.00000334 |
| ENSG00000224531 | SMIM13 | 0.633465788 | 0.0000554 |
| ENSG00000117592 | PRDX6 | 0.634099148 | 0.00000258 |
| ENSG00000142279 | WTIP | 0.634253414 | 0.0337913 |
| ENSG00000104957 | CCDC130 | 0.634278936 | 0.0000703 |
| ENSG00000166851 | PLK1 | 0.634385832 | 0.001875913 |
| ENSG00000120709 | FAM53C | 0.634667736 | 0.000068 |
| ENSG00000118482 | PHF3 | 0.635109869 | 0.00000112 |
| ENSG00000198837 | DENND4B | 0.635416557 | 0.0000246 |
| ENSG00000181788 | SIAH2 | 0.635730252 | 0.0000104 |
| ENSG00000111328 | CDK2AP1 | 0.635762262 | 0.0000347 |
| ENSG00000123349 | PFDN5 | 0.635854432 | 0.0000192 |
| ENSG00000175582 | RAB6A | 0.636409139 | 4.66E-12 |
| ENSG00000104388 | RAB2A | 0.636700382 | 9.07E-08 |
| ENSG00000114480 | GBE1 | 0.637292646 | 0.005486615 |
| ENSG00000105063 | PPP6R1 | 0.637617592 | 0.000000785 |
| ENSG00000132359 | RAP1GAP2 | 0.637790095 | 0.00010657 |
| ENSG00000147853 | AK3 | 0.637931809 | 0.000000343 |
| ENSG00000159720 | ATP6V0D1 | 0.638135374 | 0.000000137 |
| ENSG00000066027 | PPP2R5A | 0.63814111 | 0.0000541 |
| ENSG00000120437 | ACAT2 | 0.638284833 | 0.017756111 |
| ENSG00000147604 | RPL7 | 0.639549414 | 1.07E-11 |
| ENSG00000101182 | PSMA7 | 0.639683849 | 9.95E-08 |
| ENSG00000101138 | CSTF1 | 0.639850644 | 0.00000494 |
| ENSG00000214765 | SEPT7P2 | 0.640126715 | 0.002153759 |
| ENSG00000121957 | GPSM2 | 0.641105703 | 0.0007547 |
| ENSG00000147677 | EIF3H | 0.641133402 | 1.75E-12 |
| ENSG00000134697 | GNL2 | 0.641373956 | 0.000000548 |
| ENSG00000113810 | SMC4 | 0.641856548 | 0.000047 |
| ENSG00000169826 | CSGALNACT2 | 0.641859 | 0.0000491 |
| ENSG00000164405 | UQCRQ | 0.641937203 | 0.0000315 |
| ENSG00000118181 | RPS25 | 0.642062652 | 0.005678648 |
| ENSG00000131931 | THAP1 | 0.642220791 | 0.00033323 |
| ENSG00000258634 | AL160006.1 | 0.642241346 | 0.007995087 |
| ENSG00000171224 | FAM241B | 0.64232418 | 0.000251359 |
| ENSG00000092140 | G2E3 | 0.642460937 | 0.00018902 |
| ENSG00000162545 | CAMK2N1 | 0.642514414 | 3.44E-09 |
| ENSG00000024862 | CCDC28A | 0.643009293 | 0.00000362 |
| ENSG00000115128 | SF3B6 | 0.643795941 | 2.39E-08 |
| ENSG00000106554 | CHCHD3 | 0.643901879 | 1.07E-09 |
| ENSG00000149196 | HIKESHI | 0.643992901 | 0.000000539 |
| ENSG00000164093 | PITX2 | 0.644580641 | 0.000251006 |
| ENSG00000101421 | CHMP4B | 0.644663229 | 7.37E-14 |
| ENSG00000109220 | CHIC2 | 0.644982183 | 0.000972587 |
| ENSG00000164938 | TP53INP1 | 0.645088917 | 0.003971676 |
| ENSG00000173653 | RCE1 | 0.645241821 | 0.000000264 |
| ENSG00000126756 | UXT | 0.645494373 | 0.00000207 |
| ENSG00000145194 | ECE2 | 0.645502915 | 0.003972445 |
| ENSG00000086015 | MAST2 | 0.645601135 | 1.14E-08 |
| ENSG00000172270 | BSG | 0.645639028 | 1.18E-09 |
| ENSG00000084733 | RAB10 | 0.64564293 | 4.36E-13 |
| ENSG00000141568 | FOXK2 | 0.646019733 | 4.56E-08 |
| ENSG00000100320 | RBFOX2 | 0.646023103 | 0.005023825 |
| ENSG00000243678 | NME2 | 0.646027905 | 0.000134874 |
| ENSG00000108559 | NUP88 | 0.646106388 | 0.000146308 |
| ENSG00000163597 | SNHG16 | 0.646453732 | 0.000000015 |
| ENSG00000115966 | ATF2 | 0.646577931 | 2.97E-08 |
| ENSG00000197579 | TOPORS | 0.646602098 | 0.000000273 |
| ENSG00000243554 | AC004967.1 | 0.647285641 | 0.00007 |
| ENSG00000108590 | MED31 | 0.647756435 | 0.025691609 |
| ENSG00000163399 | ATP1A1 | 0.648361985 | 1.09E-11 |
| ENSG00000149269 | PAK1 | 0.648413961 | 1.19E-08 |
| ENSG00000147202 | DIAPH2 | 0.648460557 | 4.75E-08 |
| ENSG00000136021 | SCYL2 | 0.649025185 | 1.53E-12 |
| ENSG00000153975 | ZUFSP | 0.649196727 | 0.0000015 |
| ENSG00000108518 | PFN1 | 0.649316386 | 0.00000002 |
| ENSG00000183018 | SPNS2 | 0.64999671 | 0.00000274 |
| ENSG00000163755 | HPS3 | 0.650469985 | 0.000416996 |
| ENSG00000156171 | DRAM2 | 0.650978743 | 0.00000323 |
| ENSG00000132507 | EIF5A | 0.651217179 | 0.000000211 |
| ENSG00000133612 | AGAP3 | 0.651412161 | 2.13E-12 |
| ENSG00000155329 | ZCCHC10 | 0.65141233 | 0.00000482 |
| ENSG00000165288 | BRWD3 | 0.651776961 | 0.00000898 |
| ENSG00000173660 | UQCRH | 0.651811725 | 0.00000113 |
| ENSG00000101443 | WFDC2 | 0.65181606 | 0.001146379 |
| ENSG00000108424 | KPNB1 | 0.651838962 | 0.00000378 |
| ENSG00000187607 | ZNF286A | 0.651851819 | 0.001988187 |
| ENSG00000233276 | GPX1 | 0.651880202 | 0.0000653 |
| ENSG00000228502 | EEF1A1P11 | 0.652131632 | 0.002208746 |
| ENSG00000122550 | KLHL7 | 0.652260463 | 0.0000119 |
| ENSG00000104375 | STK3 | 0.652324447 | 0.000000344 |
| ENSG00000164715 | LMTK2 | 0.652848191 | 0.000000143 |
| ENSG00000101474 | APMAP | 0.653352921 | 7.8E-09 |
| ENSG00000100567 | PSMA3 | 0.653668998 | 0.000000788 |
| ENSG00000138709 | LARP1B | 0.653870898 | 0.0000375 |
| ENSG00000143158 | MPC2 | 0.653923733 | 0.002238913 |
| ENSG00000168710 | AHCYL1 | 0.654025676 | 0.0000048 |
| ENSG00000044115 | CTNNA1 | 0.654289318 | 2.59E-14 |
| ENSG00000232472 | EEF1B2P3 | 0.655065969 | 0.006315725 |
| ENSG00000123989 | CHPF | 0.655436864 | 0.00000266 |
| ENSG00000165215 | CLDN3 | 0.655493683 | 0.000000353 |
| ENSG00000164647 | STEAP1 | 0.655596012 | 0.003661466 |
| ENSG00000126775 | ATG14 | 0.656229094 | 0.0000038 |
| ENSG00000100614 | PPM1A | 0.656309519 | 1.26E-10 |
| ENSG00000153046 | CDYL | 0.656629825 | 1.1E-11 |
| ENSG00000001036 | FUCA2 | 0.656719539 | 0.000000273 |
| ENSG00000131725 | WDR44 | 0.656846688 | 4.62E-09 |
| ENSG00000169223 | LMAN2 | 0.656908753 | 0.00000134 |
| ENSG00000107223 | EDF1 | 0.657384211 | 0.00000147 |
| ENSG00000248593 | DSTNP2 | 0.657424809 | 0.000282789 |
| ENSG00000139343 | SNRPF | 0.657630172 | 0.0000239 |
| ENSG00000243679 | AC018638.5 | 0.657776235 | 0.003246046 |
| ENSG00000229119 | AC026403.1 | 0.658040932 | 0.00000904 |
| ENSG00000103995 | CEP152 | 0.658049894 | 0.005005947 |
| ENSG00000226084 | AC113935.1 | 0.65823012 | 0.00916348 |
| ENSG00000083845 | RPS5 | 0.658257847 | 0.00000187 |
| ENSG00000116815 | CD58 | 0.658365702 | 0.0004945 |
| ENSG00000175854 | SWI5 | 0.658503782 | 0.0000806 |
| ENSG00000243156 | MICAL3 | 0.659067998 | 0.000447941 |
| ENSG00000156831 | NSMCE2 | 0.659149937 | 9.06E-09 |
| ENSG00000125633 | CCDC93 | 0.659163439 | 0.00000028 |
| ENSG00000126767 | ELK1 | 0.65951136 | 0.00000454 |
| ENSG00000183864 | TOB2 | 0.659920555 | 3.11E-10 |
| ENSG00000204253 | HNRNPCP2 | 0.660150105 | 0.020028981 |
| ENSG00000136149 | RPL13AP25 | 0.660507638 | 0.00000154 |
| ENSG00000167526 | RPL13 | 0.661259921 | 3.86E-10 |
| ENSG00000037637 | FBXO42 | 0.66128902 | 0.0000178 |
| ENSG00000279095 | AC243964.3 | 0.662307627 | 0.003187082 |
| ENSG00000138785 | INTS12 | 0.662644515 | 0.0000149 |
| ENSG00000017260 | ATP2C1 | 0.663390006 | 2.04E-13 |
| ENSG00000111775 | COX6A1 | 0.664367398 | 0.00000547 |
| ENSG00000154229 | PRKCA | 0.66465125 | 0.0000927 |
| ENSG00000136908 | DPM2 | 0.664652637 | 0.0000871 |
| ENSG00000163110 | PDLIM5 | 0.665030514 | 5.12E-11 |
| ENSG00000133247 | KMT5C | 0.665486958 | 0.001630149 |
| ENSG00000125746 | EML2 | 0.66574376 | 0.00000121 |
| ENSG00000076864 | RAP1GAP | 0.666018586 | 0.000000848 |
| ENSG00000189159 | JPT1 | 0.66610906 | 0.0000291 |
| ENSG00000091436 | MAP3K20 | 0.666348712 | 0.0000348 |
| ENSG00000198862 | LTN1 | 0.666446232 | 0.000000164 |
| ENSG00000266028 | SRGAP2 | 0.666694277 | 2.74E-09 |
| ENSG00000143751 | SDE2 | 0.668067707 | 0.000000128 |
| ENSG00000184305 | CCSER1 | 0.668087049 | 0.00000772 |
| ENSG00000133884 | DPF2 | 0.668297063 | 0.0000036 |
| ENSG00000197114 | ZGPAT | 0.668528704 | 0.000152627 |
| ENSG00000065485 | PDIA5 | 0.668815842 | 9.26E-08 |
| ENSG00000173846 | PLK3 | 0.669737617 | 0.004807295 |
| ENSG00000155959 | VBP1 | 0.670591605 | 0.00000143 |
| ENSG00000163681 | SLMAP | 0.670678514 | 3.02E-12 |
| ENSG00000147416 | ATP6V1B2 | 0.670782701 | 4.23E-10 |
| ENSG00000104859 | CLASRP | 0.670825161 | 0.000000134 |
| ENSG00000114391 | RPL24 | 0.670899032 | 1.51E-08 |
| ENSG00000078902 | TOLLIP | 0.671508193 | 9.69E-13 |
| ENSG00000154813 | DPH3 | 0.671678155 | 0.0000216 |
| ENSG00000157514 | TSC22D3 | 0.672119843 | 0.002784508 |
| ENSG00000122696 | SLC25A51 | 0.672508307 | 0.000425903 |
| ENSG00000131143 | COX4I1 | 0.672538452 | 0.000000769 |
| ENSG00000234498 | RPL13AP20 | 0.672630338 | 0.032236848 |
| ENSG00000130706 | ADRM1 | 0.673022331 | 0.00000632 |
| ENSG00000150093 | ITGB1 | 0.673494171 | 8.1E-19 |
| ENSG00000164109 | MAD2L1 | 0.673633028 | 0.00793823 |
| ENSG00000213058 | AL365357.1 | 0.674598589 | 0.000895689 |
| ENSG00000106400 | ZNHIT1 | 0.674686885 | 0.00000106 |
| ENSG00000160753 | RUSC1 | 0.675572503 | 0.00000121 |
| ENSG00000140534 | TICRR | 0.676015957 | 0.017833799 |
| ENSG00000277377 |  | 0.676052251 | 7.63E-09 |
| ENSG00000048649 | RSF1 | 0.676077405 | 0.000000372 |
| ENSG00000152484 | USP12 | 0.676215954 | 7.87E-08 |
| ENSG00000150787 | PTS | 0.676367493 | 0.000000259 |
| ENSG00000143569 | UBAP2L | 0.676697922 | 2.27E-08 |
| ENSG00000261068 | AL512274.1 | 0.676983515 | 0.046832309 |
| ENSG00000148200 | NR6A1 | 0.677406601 | 0.002127567 |
| ENSG00000166228 | PCBD1 | 0.677556545 | 0.000000892 |
| ENSG00000171530 | TBCA | 0.677624571 | 0.000000169 |
| ENSG00000189067 | LITAF | 0.677677844 | 5.71E-11 |
| ENSG00000124313 | IQSEC2 | 0.677974269 | 0.000000147 |
| ENSG00000138434 | SSFA2 | 0.678359409 | 4.41E-11 |
| ENSG00000167173 | C15orf39 | 0.678671478 | 5.77E-08 |
| ENSG00000172594 | SMPDL3A | 0.678813684 | 0.007700274 |
| ENSG00000108389 | MTMR4 | 0.679161976 | 0.000207298 |
| ENSG00000178464 | RPL10P16 | 0.679382455 | 0.0000358 |
| ENSG00000106367 | AP1S1 | 0.679632369 | 0.000000623 |
| ENSG00000104517 | UBR5 | 0.679655067 | 0.000000456 |
| ENSG00000172375 | C2CD2L | 0.679947936 | 0.00000384 |
| ENSG00000111678 | C12orf57 | 0.679962797 | 0.0000273 |
| ENSG00000148943 | LIN7C | 0.680174237 | 0.0000075 |
| ENSG00000138867 | GUCD1 | 0.680582054 | 0.000082 |
| ENSG00000111669 | TPI1 | 0.680833521 | 0.000000362 |
| ENSG00000144895 | EIF2A | 0.681732946 | 3.25E-12 |
| ENSG00000169241 | SLC50A1 | 0.68201736 | 0.0000891 |
| ENSG00000102908 | NFAT5 | 0.682213147 | 0.008210384 |
| ENSG00000102158 | MAGT1 | 0.682351737 | 0.00000222 |
| ENSG00000113732 | ATP6V0E1 | 0.682758251 | 3.91E-09 |
| ENSG00000084207 | GSTP1 | 0.682848176 | 2.16E-08 |
| ENSG00000165895 | ARHGAP42 | 0.683235378 | 0.00000706 |
| ENSG00000125691 | RPL23 | 0.683470048 | 7.99E-12 |
| ENSG00000204899 | MZT1 | 0.683793984 | 0.001484085 |
| ENSG00000087586 | AURKA | 0.68422499 | 0.002614247 |
| ENSG00000087088 | BAX | 0.684436599 | 7.46E-08 |
| ENSG00000154582 | ELOC | 0.684549877 | 0.00000468 |
| ENSG00000215030 | RPL13P12 | 0.684633695 | 0.000000107 |
| ENSG00000180817 | PPA1 | 0.685460746 | 3.38E-13 |
| ENSG00000101544 | ADNP2 | 0.685554864 | 1.28E-11 |
| ENSG00000163444 | TMEM183A | 0.685663954 | 0.0000323 |
| ENSG00000120265 | PCMT1 | 0.685883675 | 0.00000112 |
| ENSG00000135953 | MFSD9 | 0.685994242 | 2.34E-10 |
| ENSG00000233913 | RPL10P9 | 0.686060011 | 0.009863701 |
| ENSG00000142937 | RPS8 | 0.686348601 | 1.13E-11 |
| ENSG00000111481 | COPZ1 | 0.686956399 | 0.0000189 |
| ENSG00000177030 | DEAF1 | 0.68709839 | 0.0000147 |
| ENSG00000125952 | MAX | 0.687158719 | 2.22E-09 |
| ENSG00000166337 | TAF10 | 0.687407672 | 7.49E-09 |
| ENSG00000001167 | NFYA | 0.687472498 | 4.32E-10 |
| ENSG00000162298 | SYVN1 | 0.68811447 | 0.000214374 |
| ENSG00000124214 | STAU1 | 0.688144108 | 3.56E-17 |
| ENSG00000118816 | CCNI | 0.688402434 | 3.92E-12 |
| ENSG00000188522 | FAM83G | 0.688628166 | 1.85E-12 |
| ENSG00000108298 | RPL19 | 0.688980562 | 2.1E-11 |
| ENSG00000138674 | SEC31A | 0.689116598 | 7.18E-12 |
| ENSG00000154874 | CCDC144B | 0.689366861 | 0.034702028 |
| ENSG00000234608 | MAPKAPK5-AS1 | 0.689594068 | 0.00000932 |
| ENSG00000108010 | GLRX3 | 0.689925848 | 0.000000176 |
| ENSG00000166002 | SMCO4 | 0.690126049 | 0.000388949 |
| ENSG00000072849 | DERL2 | 0.690160391 | 0.00000701 |
| ENSG00000117523 | PRRC2C | 0.69016932 | 0.0000516 |
| ENSG00000272888 | LINC01578 | 0.690346012 | 4.18E-09 |
| ENSG00000165502 | RPL36AL | 0.690533775 | 4.22E-09 |
| ENSG00000141858 | SAMD1 | 0.691155188 | 0.000000257 |
| ENSG00000230202 | AL450405.1 | 0.69165832 | 0.003722132 |
| ENSG00000160999 | SH2B2 | 0.691800555 | 0.021558019 |
| ENSG00000144713 | RPL32 | 0.692199072 | 9.69E-09 |
| ENSG00000115307 | AUP1 | 0.692389618 | 0.00000279 |
| ENSG00000128578 | STRIP2 | 0.693306937 | 0.004507556 |
| ENSG00000197747 | S100A10 | 0.693386656 | 0.0000128 |
| ENSG00000228106 | AL392172.1 | 0.693542868 | 0.009230433 |
| ENSG00000121073 | SLC35B1 | 0.69387692 | 0.000000552 |
| ENSG00000242299 | AC073861.1 | 0.694413193 | 0.000062 |
| ENSG00000038382 | TRIO | 0.695028638 | 0.000000134 |
| ENSG00000130703 | OSBPL2 | 0.695102405 | 0.00000134 |
| ENSG00000137154 | RPS6 | 0.69552397 | 7.9E-09 |
| ENSG00000114942 | EEF1B2 | 0.695582842 | 7.28E-10 |
| ENSG00000113013 | HSPA9 | 0.695621485 | 1.42E-11 |
| ENSG00000228474 | OST4 | 0.695768633 | 0.0000255 |
| ENSG00000173915 | ATP5MD | 0.695816424 | 0.000000211 |
| ENSG00000184661 | CDCA2 | 0.696202983 | 0.003571089 |
| ENSG00000134684 | YARS | 0.696274521 | 3.25E-08 |
| ENSG00000117862 | TXNDC12 | 0.696526992 | 0.00000826 |
| ENSG00000116406 | EDEM3 | 0.696641207 | 0.000000202 |
| ENSG00000185246 | PRPF39 | 0.696702789 | 2.51E-08 |
| ENSG00000244716 | BX679664.3 | 0.697055163 | 0.016502847 |
| ENSG00000067992 | PDK3 | 0.697198806 | 0.029238607 |
| ENSG00000023318 | ERP44 | 0.697263385 | 0.00000161 |
| ENSG00000139438 | FAM222A | 0.697695349 | 0.022019012 |
| ENSG00000110651 | CD81 | 0.697732632 | 4.78E-09 |
| ENSG00000279407 | AC007191.1 | 0.697867435 | 0.015786542 |
| ENSG00000107872 | FBXL15 | 0.698375988 | 0.00000364 |
| ENSG00000101194 | SLC17A9 | 0.698987949 | 0.000143501 |
| ENSG00000160408 | ST6GALNAC6 | 0.699180097 | 4.37E-09 |
| ENSG00000137251 | TINAG | 0.699567126 | 0.036512093 |
| ENSG00000185641 | AC034236.1 | 0.69962401 | 2.96E-10 |
| ENSG00000134193 | REG4 | 0.700808027 | 0.004341563 |
| ENSG00000108523 | RNF167 | 0.700835634 | 0.00000446 |
| ENSG00000170425 | ADORA2B | 0.700868352 | 0.000159974 |
| ENSG00000090020 | SLC9A1 | 0.700929531 | 0.000512534 |
| ENSG00000185896 | LAMP1 | 0.70101435 | 4.95E-19 |
| ENSG00000171497 | PPID | 0.701849444 | 1.53E-09 |
| ENSG00000085999 | RAD54L | 0.701966318 | 0.021164104 |
| ENSG00000217716 | RPS10P3 | 0.70198457 | 0.004529219 |
| ENSG00000109171 | SLAIN2 | 0.702399305 | 1.29E-12 |
| ENSG00000265808 | SEC22B | 0.702460533 | 0.0000211 |
| ENSG00000174437 | ATP2A2 | 0.702653998 | 0.00000159 |
| ENSG00000230456 |  | 0.702873969 | 0.000000563 |
| ENSG00000143947 | RPS27A | 0.70325692 | 1.35E-11 |
| ENSG00000130827 | PLXNA3 | 0.703272944 | 0.0000219 |
| ENSG00000106780 | MEGF9 | 0.703400112 | 0.00000586 |
| ENSG00000101166 | PRELID3B | 0.703493337 | 2.28E-11 |
| ENSG00000231991 | ANXA2P2 | 0.703522843 | 0.000000341 |
| ENSG00000240342 | RPS2P5 | 0.703826822 | 0.000000121 |
| ENSG00000170260 | ZNF212 | 0.70418879 | 0.000000299 |
| ENSG00000103275 | UBE2I | 0.704228019 | 0.000000489 |
| ENSG00000142534 | RPS11 | 0.70428788 | 3.92E-11 |
| ENSG00000165775 | FUNDC2 | 0.70521174 | 2.76E-08 |
| ENSG00000272620 | AFAP1-AS1 | 0.705390652 | 0.000000319 |
| ENSG00000185650 | ZFP36L1 | 0.705480349 | 1.17E-11 |
| ENSG00000114030 | KPNA1 | 0.705932518 | 3.53E-11 |
| ENSG00000129195 | PIMREG | 0.706220187 | 0.000919713 |
| ENSG00000124357 | NAGK | 0.706387142 | 0.0000281 |
| ENSG00000158161 | EYA3 | 0.707188305 | 0.00000847 |
| ENSG00000186468 | RPS23 | 0.707551154 | 4.32E-12 |
| ENSG00000071553 | ATP6AP1 | 0.707562615 | 8.71E-12 |
| ENSG00000140988 | RPS2 | 0.707924398 | 0.00000264 |
| ENSG00000170832 | USP32 | 0.708565779 | 0.00000066 |
| ENSG00000077044 | DGKD | 0.708844497 | 1.13E-10 |
| ENSG00000132613 | MTSS1L | 0.709184816 | 0.00000181 |
| ENSG00000158710 | TAGLN2 | 0.709211511 | 0.000000493 |
| ENSG00000151327 | FAM177A1 | 0.709488025 | 1.76E-09 |
| ENSG00000122566 | HNRNPA2B1 | 0.709504414 | 0.00000126 |
| ENSG00000180535 | BHLHA15 | 0.709549337 | 0.009314587 |
| ENSG00000167658 | EEF2 | 0.709625258 | 1.37E-19 |
| ENSG00000196656 | AC004057.1 | 0.710099224 | 0.000933927 |
| ENSG00000115339 | GALNT3 | 0.710145113 | 0.0000473 |
| ENSG00000114573 | ATP6V1A | 0.71043445 | 0.000000104 |
| ENSG00000138768 | USO1 | 0.710485201 | 6.19E-18 |
| ENSG00000149273 | RPS3 | 0.710783424 | 1.64E-11 |
| ENSG00000188846 | RPL14 | 0.710875496 | 8.11E-12 |
| ENSG00000106683 | LIMK1 | 0.710920792 | 6.53E-09 |
| ENSG00000196204 | RNF216P1 | 0.710975551 | 0.0000381 |
| ENSG00000185158 | LRRC37B | 0.711092303 | 0.001468326 |
| ENSG00000180879 | SSR4 | 0.71111513 | 2.17E-08 |
| ENSG00000108262 | GIT1 | 0.711489982 | 6.75E-09 |
| ENSG00000182718 | ANXA2 | 0.711666778 | 0.000000012 |
| ENSG00000138698 | RAP1GDS1 | 0.711737341 | 6.65E-10 |
| ENSG00000149591 | TAGLN | 0.71200315 | 0.002245761 |
| ENSG00000127824 | TUBA4A | 0.712310182 | 0.000612606 |
| ENSG00000165828 | PRAP1 | 0.712791468 | 0.000393345 |
| ENSG00000159346 | ADIPOR1 | 0.712854644 | 0.00000615 |
| ENSG00000213178 | RPL22P1 | 0.71295773 | 0.004661669 |
| ENSG00000011347 | SYT7 | 0.713040336 | 2.38E-10 |
| ENSG00000122026 | RPL21 | 0.713062401 | 0.00000119 |
| ENSG00000175505 | CLCF1 | 0.713471058 | 0.003201079 |
| ENSG00000136888 | ATP6V1G1 | 0.713583178 | 3.92E-13 |
| ENSG00000180398 | MCFD2 | 0.714454242 | 1.48E-10 |
| ENSG00000171612 | SLC25A33 | 0.714575758 | 9.88E-08 |
| ENSG00000100554 | ATP6V1D | 0.715386618 | 7.61E-11 |
| ENSG00000163795 | ZNF513 | 0.715620252 | 0.000000268 |
| ENSG00000138594 | TMOD3 | 0.715715696 | 2.94E-17 |
| ENSG00000100526 | CDKN3 | 0.715900352 | 0.002221546 |
| ENSG00000085274 | MYNN | 0.71597028 | 2.85E-10 |
| ENSG00000152104 | PTPN14 | 0.716336365 | 0.00000183 |
| ENSG00000103035 | PSMD7 | 0.716378964 | 1.64E-09 |
| ENSG00000116095 | PLEKHA3 | 0.716577557 | 0.0000981 |
| ENSG00000188786 | MTF1 | 0.717611413 | 2.67E-11 |
| ENSG00000174749 | FAM241A | 0.717862687 | 0.000107448 |
| ENSG00000006712 | PAF1 | 0.718379079 | 2.84E-11 |
| ENSG00000129084 | PSMA1 | 0.719246193 | 0.00000228 |
| ENSG00000178913 | TAF7 | 0.71946772 | 5.06E-14 |
| ENSG00000219023 | AL033519.2 | 0.719763329 | 0.029456273 |
| ENSG00000147403 | RPL10 | 0.719814854 | 8.8E-13 |
| ENSG00000102221 | JADE3 | 0.7208747 | 1.74E-11 |
| ENSG00000138326 | RPS24 | 0.721002231 | 5.32E-13 |
| ENSG00000111252 | SH2B3 | 0.721064051 | 0.007811908 |
| ENSG00000109475 | RPL34 | 0.721217844 | 1.24E-10 |
| ENSG00000099139 | PCSK5 | 0.72142653 | 0.0000179 |
| ENSG00000180758 | GPR157 | 0.721617593 | 0.001437713 |
| ENSG00000003393 | ALS2 | 0.721668754 | 1.05E-08 |
| ENSG00000187531 | SIRT7 | 0.722337448 | 3.43E-08 |
| ENSG00000000460 | C1orf112 | 0.722410635 | 0.001503978 |
| ENSG00000023287 | RB1CC1 | 0.722946541 | 3E-14 |
| ENSG00000058262 | SEC61A1 | 0.723081232 | 2.23E-11 |
| ENSG00000121644 | DESI2 | 0.723365381 | 0.0000262 |
| ENSG00000047365 | ARAP2 | 0.723965949 | 0.00000967 |
| ENSG00000115268 | RPS15 | 0.724200625 | 1.36E-11 |
| ENSG00000101057 | MYBL2 | 0.724705544 | 0.000446911 |
| ENSG00000126603 | GLIS2 | 0.725613587 | 0.039397422 |
| ENSG00000073605 | GSDMB | 0.725819825 | 0.0000374 |
| ENSG00000166126 | AMN | 0.726561653 | 0.009606592 |
| ENSG00000231889 | TRAF3IP2-AS1 | 0.7266363 | 0.033184786 |
| ENSG00000250182 | EEF1A1P13 | 0.726726437 | 0.000559526 |
| ENSG00000214389 | RPS3AP26 | 0.726789712 | 0.000330593 |
| ENSG00000071859 | FAM50A | 0.727539171 | 6.95E-12 |
| ENSG00000170873 | MTSS1 | 0.727621143 | 0.00000737 |
| ENSG00000161533 | ACOX1 | 0.72783369 | 2.7E-10 |
| ENSG00000111885 | MAN1A1 | 0.727969483 | 0.000000273 |
| ENSG00000126070 | AGO3 | 0.728028883 | 0.00000278 |
| ENSG00000113649 | TCERG1 | 0.728054063 | 2.31E-09 |
| ENSG00000137504 | CREBZF | 0.728367615 | 4.36E-14 |
| ENSG00000205339 | IPO7 | 0.728648531 | 1.21E-15 |
| ENSG00000170325 | PRDM10 | 0.728698861 | 0.00003 |
| ENSG00000162522 | KIAA1522 | 0.72892787 | 2.07E-09 |
| ENSG00000091542 | ALKBH5 | 0.729371648 | 0.000002 |
| ENSG00000101400 | SNTA1 | 0.729492831 | 0.012557774 |
| ENSG00000212719 | C17orf51 | 0.729554125 | 0.01511496 |
| ENSG00000133606 | MKRN1 | 0.730317284 | 5.49E-14 |
| ENSG00000119707 | RBM25 | 0.730378104 | 2.01E-11 |
| ENSG00000269867 | AC010326.3 | 0.730410944 | 0.049703778 |
| ENSG00000188994 | ZNF292 | 0.730444169 | 5.57E-08 |
| ENSG00000140319 | SRP14 | 0.730660617 | 2.21E-11 |
| ENSG00000181004 | BBS12 | 0.730801177 | 0.004186386 |
| ENSG00000143033 | MTF2 | 0.730830845 | 0.0000562 |
| ENSG00000156958 | GALK2 | 0.731359924 | 2.29E-10 |
| ENSG00000174996 | KLC2 | 0.731719965 | 0.000000216 |
| ENSG00000137947 | GTF2B | 0.732327323 | 0.0000026 |
| ENSG00000082153 | BZW1 | 0.732589736 | 1.99E-11 |
| ENSG00000196236 | XPNPEP3 | 0.73273907 | 0.0000297 |
| ENSG00000111276 | CDKN1B | 0.732815165 | 3.53E-08 |
| ENSG00000197956 | S100A6 | 0.732891627 | 1.22E-09 |
| ENSG00000168646 | AXIN2 | 0.732986681 | 0.0000214 |
| ENSG00000274272 | AC069281.2 | 0.733180015 | 0.011198889 |
| ENSG00000236824 | BCYRN1 | 0.734120964 | 0.043852676 |
| ENSG00000104671 | DCTN6 | 0.734363319 | 0.000000647 |
| ENSG00000115616 | SLC9A2 | 0.734535846 | 0.006170148 |
| ENSG00000244398 | AC116533.1 | 0.734722165 | 1.48E-08 |
| ENSG00000124107 | SLPI | 0.734747253 | 0.048692188 |
| ENSG00000105438 | KDELR1 | 0.735172943 | 1.51E-11 |
| ENSG00000224861 | YBX1P1 | 0.73517744 | 0.007461115 |
| ENSG00000233927 | RPS28 | 0.73519346 | 6.62E-11 |
| ENSG00000127578 | WFIKKN1 | 0.73521072 | 0.018400674 |
| ENSG00000240036 | AC104563.1 | 0.735787444 | 5.25E-09 |
| ENSG00000124614 | RPS10 | 0.735895961 | 0.000000264 |
| ENSG00000100902 | PSMA6 | 0.736120131 | 0.0000256 |
| ENSG00000263057 |  | 0.736235284 | 0.036368772 |
| ENSG00000180089 | TMEM86B | 0.736291553 | 0.006926285 |
| ENSG00000237214 | AL080243.2 | 0.736911549 | 0.00000365 |
| ENSG00000184047 | DIABLO | 0.737041884 | 0.000688486 |
| ENSG00000137203 | TFAP2A | 0.737703972 | 0.001537437 |
| ENSG00000119778 | ATAD2B | 0.737724219 | 0.00000392 |
| ENSG00000111696 | NT5DC3 | 0.737959303 | 0.000000348 |
| ENSG00000156639 | ZFAND3 | 0.738035784 | 4.87E-13 |
| ENSG00000134030 | CTIF | 0.738359526 | 5.6E-09 |
| ENSG00000170653 | ATF7 | 0.738572057 | 0.00000201 |
| ENSG00000005022 | SLC25A5 | 0.739096774 | 5.51E-09 |
| ENSG00000148572 | NRBF2 | 0.740120082 | 8.55E-11 |
| ENSG00000164543 | STK17A | 0.740252154 | 0.00000031 |
| ENSG00000006453 | BAIAP2L1 | 0.740439746 | 1.62E-13 |
| ENSG00000160588 | MPZL3 | 0.740656711 | 1.09E-09 |
| ENSG00000182899 | RPL35A | 0.740870929 | 2.35E-13 |
| ENSG00000091844 | RGS17 | 0.7408901 | 0.000398964 |
| ENSG00000102007 | PLP2 | 0.741000866 | 1.98E-11 |
| ENSG00000087301 | TXNDC16 | 0.741516018 | 0.006877189 |
| ENSG00000105357 | MYH14 | 0.742349898 | 6.04E-08 |
| ENSG00000185033 | SEMA4B | 0.742600161 | 1.02E-11 |
| ENSG00000108448 | TRIM16L | 0.742726312 | 0.0000437 |
| ENSG00000109445 | ZNF330 | 0.74277825 | 1.18E-10 |
| ENSG00000097021 | ACOT7 | 0.743040873 | 0.000000154 |
| ENSG00000182324 | KCNJ14 | 0.743063269 | 0.001384074 |
| ENSG00000138073 | PREB | 0.744185805 | 0.00000737 |
| ENSG00000113716 | HMGXB3 | 0.744328437 | 1.83E-10 |
| ENSG00000141741 | MIEN1 | 0.745035802 | 0.000000573 |
| ENSG00000169905 | TOR1AIP2 | 0.745073682 | 4.61E-09 |
| ENSG00000140832 | MARVELD3 | 0.745200877 | 0.0000213 |
| ENSG00000084234 | APLP2 | 0.745527401 | 1.49E-15 |
| ENSG00000274229 |  | 0.745934067 | 0.0000253 |
| ENSG00000135541 | AHI1 | 0.74619076 | 0.00000321 |
| ENSG00000136238 | RAC1 | 0.746529417 | 3.63E-16 |
| ENSG00000176340 | COX8A | 0.746970745 | 0.0000183 |
| ENSG00000101670 | LIPG | 0.747080202 | 0.001316667 |
| ENSG00000168259 | DNAJC7 | 0.747670966 | 5.95E-15 |
| ENSG00000152492 | CCDC50 | 0.747714833 | 4.2E-10 |
| ENSG00000140526 | ABHD2 | 0.747979655 | 0.0000111 |
| ENSG00000110723 | EXPH5 | 0.748252434 | 0.000072 |
| ENSG00000167642 | SPINT2 | 0.748393557 | 7.91E-11 |
| ENSG00000101224 | CDC25B | 0.748605044 | 0.00000238 |
| ENSG00000134755 | DSC2 | 0.749318863 | 2.97E-11 |
| ENSG00000069535 | MAOB | 0.749609788 | 0.002469539 |
| ENSG00000173418 | NAA20 | 0.749761542 | 6.53E-09 |
| ENSG00000060138 | YBX3 | 0.749969318 | 1.58E-11 |
| ENSG00000130733 | YIPF2 | 0.750570457 | 0.0000209 |
| ENSG00000132661 | NXT1 | 0.751367917 | 0.0000131 |
| ENSG00000197647 | ZNF433 | 0.752498892 | 0.032100639 |
| ENSG00000078699 | CBFA2T2 | 0.752534751 | 0.000000598 |
| ENSG00000049860 | HEXB | 0.752879506 | 3.46E-10 |
| ENSG00000108239 | TBC1D12 | 0.752909831 | 0.0000146 |
| ENSG00000111640 | GAPDH | 0.753125398 | 0.000000119 |
| ENSG00000173221 | GLRX | 0.753497157 | 0.0000217 |
| ENSG00000230897 | RPS18P12 | 0.753544703 | 0.0000949 |
| ENSG00000280670 | CCDC163 | 0.753628251 | 0.00806551 |
| ENSG00000147894 | C9orf72 | 0.753809902 | 0.000139694 |
| ENSG00000067057 | PFKP | 0.754375467 | 4.64E-11 |
| ENSG00000183856 | IQGAP3 | 0.754626492 | 0.000276641 |
| ENSG00000140299 | BNIP2 | 0.755039366 | 1.65E-09 |
| ENSG00000113575 | PPP2CA | 0.755710612 | 1.95E-13 |
| ENSG00000145781 | COMMD10 | 0.755908719 | 0.0000382 |
| ENSG00000177600 | RPLP2 | 0.756095889 | 0.000000246 |
| ENSG00000034713 | GABARAPL2 | 0.756281705 | 0.000000571 |
| ENSG00000163814 | CDCP1 | 0.75681533 | 1.47E-12 |
| ENSG00000172071 | EIF2AK3 | 0.756925012 | 0.00000832 |
| ENSG00000168214 | RBPJ | 0.757128663 | 1.91E-14 |
| ENSG00000171940 | ZNF217 | 0.757776761 | 5.52E-12 |
| ENSG00000134109 | EDEM1 | 0.758078404 | 5.32E-12 |
| ENSG00000145782 | ATG12 | 0.758519657 | 1.65E-12 |
| ENSG00000134825 | TMEM258 | 0.758544693 | 0.000000665 |
| ENSG00000172301 | COPRS | 0.758612342 | 0.000000297 |
| ENSG00000087884 | AAMDC | 0.759444748 | 0.00000406 |
| ENSG00000130589 | HELZ2 | 0.759769436 | 0.0000148 |
| ENSG00000100241 | SBF1 | 0.759858412 | 2.27E-11 |
| ENSG00000102531 | FNDC3A | 0.759985666 | 6.9E-13 |
| ENSG00000169228 | RAB24 | 0.760100066 | 0.0000221 |
| ENSG00000181444 | ZNF467 | 0.760144181 | 0.009315873 |
| ENSG00000095794 | CREM | 0.760272988 | 0.0000261 |
| ENSG00000108669 | CYTH1 | 0.760641764 | 1.33E-10 |
| ENSG00000012211 | PRICKLE3 | 0.761343195 | 0.00000377 |
| ENSG00000162231 | NXF1 | 0.761465534 | 1.05E-10 |
| ENSG00000175061 | LRRC75A-AS1 | 0.761499605 | 2.57E-11 |
| ENSG00000279010 |  | 0.761641847 | 0.034577846 |
| ENSG00000164615 | CAMLG | 0.761707941 | 6.88E-10 |
| ENSG00000213420 | GPC2 | 0.76173964 | 0.027159347 |
| ENSG00000092841 | MYL6 | 0.761842056 | 5.7E-12 |
| ENSG00000122643 | NT5C3A | 0.762227358 | 1.47E-15 |
| ENSG00000197077 | KIAA1671 | 0.762826482 | 0.000000211 |
| ENSG00000138764 | CCNG2 | 0.763156304 | 0.0000117 |
| ENSG00000128881 | TTBK2 | 0.763298651 | 0.000000107 |
| ENSG00000205659 | LIN52 | 0.763939145 | 0.000116974 |
| ENSG00000151332 | MBIP | 0.764166367 | 0.002601208 |
| ENSG00000090273 | NUDC | 0.764512244 | 8.85E-12 |
| ENSG00000146677 | AC004453.1 | 0.76452982 | 0.023663282 |
| ENSG00000100523 | DDHD1 | 0.765127618 | 0.000000112 |
| ENSG00000142634 | EFHD2 | 0.765588184 | 9.38E-11 |
| ENSG00000137818 | RPLP1 | 0.76589465 | 2.85E-10 |
| ENSG00000141391 | PRELID3A | 0.767223787 | 0.00012588 |
| ENSG00000128683 | GAD1 | 0.767305774 | 0.0015339 |
| ENSG00000124222 | STX16 | 0.767650967 | 4.92E-12 |
| ENSG00000068366 | ACSL4 | 0.768285823 | 8.72E-10 |
| ENSG00000249264 | EEF1A1P9 | 0.768316477 | 0.000335398 |
| ENSG00000170606 | HSPA4 | 0.768432074 | 1.63E-09 |
| ENSG00000175556 | LONRF3 | 0.768931445 | 0.00000116 |
| ENSG00000130164 | LDLR | 0.769670715 | 2.03E-09 |
| ENSG00000120913 | PDLIM2 | 0.769681257 | 0.002377396 |
| ENSG00000180357 | ZNF609 | 0.769791918 | 0.000000196 |
| ENSG00000076382 | SPAG5 | 0.770078642 | 0.000831424 |
| ENSG00000184164 | CRELD2 | 0.770189033 | 0.000160286 |
| ENSG00000121716 | PILRB | 0.77021267 | 0.003398074 |
| ENSG00000118508 | RAB32 | 0.77055358 | 0.000220716 |
| ENSG00000185825 | BCAP31 | 0.770846704 | 3.17E-12 |
| ENSG00000164951 | PDP1 | 0.771484127 | 1.68E-08 |
| ENSG00000142046 | TMEM91 | 0.771604965 | 0.010862623 |
| ENSG00000211584 | SLC48A1 | 0.771665041 | 0.000000308 |
| ENSG00000163683 | SMIM14 | 0.772126825 | 3.37E-12 |
| ENSG00000156482 | RPL30 | 0.772357655 | 1.05E-12 |
| ENSG00000120963 | ZNF706 | 0.77239474 | 3.18E-09 |
| ENSG00000183779 | ZNF703 | 0.772700304 | 2.47E-08 |
| ENSG00000055118 | KCNH2 | 0.772772542 | 0.00000984 |
| ENSG00000170037 | CNTROB | 0.773017067 | 3.75E-09 |
| ENSG00000186591 | UBE2H | 0.773035347 | 2.02E-15 |
| ENSG00000166794 | PPIB | 0.773237211 | 1.29E-08 |
| ENSG00000147862 | NFIB | 0.773475589 | 4.07E-12 |
| ENSG00000136942 | RPL35 | 0.773603239 | 3.14E-08 |
| ENSG00000181061 | HIGD1A | 0.773737354 | 0.000000042 |
| ENSG00000157106 | SMG1 | 0.774138045 | 0.0000242 |
| ENSG00000141985 | SH3GL1 | 0.774152241 | 0.000000832 |
| ENSG00000100330 | MTMR3 | 0.774715562 | 0.0000634 |
| ENSG00000225484 | NUTM2B-AS1 | 0.774769464 | 1.83E-08 |
| ENSG00000239264 | TXNDC5 | 0.774841464 | 0.00000169 |
| ENSG00000130725 | UBE2M | 0.776033592 | 0.00000118 |
| ENSG00000166441 | RPL27A | 0.776117716 | 7.34E-10 |
| ENSG00000221983 | UBA52 | 0.776163166 | 8.07E-11 |
| ENSG00000144118 | RALB | 0.776458377 | 3.68E-17 |
| ENSG00000089916 | GPATCH2L | 0.776617242 | 4.62E-11 |
| ENSG00000006576 | PHTF2 | 0.776745822 | 0.000123469 |
| ENSG00000276482 |  | 0.776796336 | 0.000000016 |
| ENSG00000156508 | EEF1A1 | 0.77715563 | 2.88E-14 |
| ENSG00000109332 | UBE2D3 | 0.777220989 | 1.5E-11 |
| ENSG00000165175 | MID1IP1 | 0.777369575 | 7.08E-15 |
| ENSG00000090975 | PITPNM2 | 0.777626718 | 0.00100219 |
| ENSG00000172809 | RPL38 | 0.77786472 | 4.63E-09 |
| ENSG00000101752 | MIB1 | 0.77806219 | 3.14E-09 |
| ENSG00000096746 | HNRNPH3 | 0.778380877 | 4.64E-15 |
| ENSG00000056972 | TRAF3IP2 | 0.778386174 | 0.000000012 |
| ENSG00000101972 | STAG2 | 0.778394591 | 1.74E-13 |
| ENSG00000213064 | SFT2D2 | 0.778874033 | 0.000000977 |
| ENSG00000253570 | RNF5P1 | 0.779604277 | 0.008097748 |
| ENSG00000161970 | RPL26 | 0.7796109 | 2.6E-11 |
| ENSG00000172932 | ANKRD13D | 0.779615265 | 4.9E-13 |
| ENSG00000228232 | GAPDHP1 | 0.779803896 | 0.041992454 |
| ENSG00000148498 | PARD3 | 0.780242999 | 1.96E-19 |
| ENSG00000165280 | VCP | 0.781077762 | 1.77E-10 |
| ENSG00000136930 | PSMB7 | 0.781319788 | 3.85E-09 |
| ENSG00000178999 | AURKB | 0.781927939 | 0.001569949 |
| ENSG00000160182 | TFF1 | 0.781967855 | 0.01538768 |
| ENSG00000247556 | OIP5-AS1 | 0.78278595 | 5.88E-08 |
| ENSG00000213937 | CLDN9 | 0.782934806 | 0.003895706 |
| ENSG00000173275 | ZNF449 | 0.78416192 | 0.0000282 |
| ENSG00000083828 | ZNF586 | 0.784656779 | 2.98E-09 |
| ENSG00000025800 | KPNA6 | 0.78523186 | 1.05E-10 |
| ENSG00000107862 | GBF1 | 0.785414588 | 2.83E-08 |
| ENSG00000148110 | MFSD14B | 0.785470091 | 5.87E-08 |
| ENSG00000158769 | F11R | 0.785853311 | 3.78E-12 |
| ENSG00000161016 | RPL8 | 0.786111548 | 7.33E-11 |
| ENSG00000134970 | TMED7 | 0.786124736 | 1.72E-13 |
| ENSG00000146477 | SLC22A3 | 0.786306358 | 0.003380202 |
| ENSG00000096717 | SIRT1 | 0.786691077 | 8.08E-15 |
| ENSG00000226415 | TPI1P1 | 0.786792486 | 0.008585362 |
| ENSG00000251369 | ZNF550 | 0.786858303 | 0.001944495 |
| ENSG00000145592 | RPL37 | 0.788160241 | 7.35E-13 |
| ENSG00000008710 | PKD1 | 0.788176158 | 0.0000499 |
| ENSG00000181035 | SLC25A42 | 0.788605987 | 0.0000596 |
| ENSG00000131469 | RPL27 | 0.788893462 | 4.32E-09 |
| ENSG00000101266 | CSNK2A1 | 0.789410636 | 1.62E-09 |
| ENSG00000133065 | SLC41A1 | 0.789643956 | 1.68E-11 |
| ENSG00000122965 | RBM19 | 0.789680335 | 1.54E-09 |
| ENSG00000197756 | RPL37A | 0.789890232 | 7.52E-13 |
| ENSG00000126821 | SGPP1 | 0.789999906 | 3.08E-11 |
| ENSG00000117525 | F3 | 0.790762668 | 0.000166 |
| ENSG00000196205 | EEF1A1P5 | 0.791261468 | 0.000000485 |
| ENSG00000237296 | SMG1P1 | 0.791431486 | 0.005740359 |
| ENSG00000175073 | VCPIP1 | 0.791481241 | 1.69E-10 |
| ENSG00000088325 | TPX2 | 0.791716978 | 0.00000482 |
| ENSG00000114331 | ACAP2 | 0.791730684 | 5.21E-12 |
| ENSG00000118046 | STK11 | 0.793171759 | 9.75E-09 |
| ENSG00000100814 | CCNB1IP1 | 0.793289711 | 8.19E-15 |
| ENSG00000220472 | AL139095.2 | 0.793374795 | 0.017211374 |
| ENSG00000197265 | GTF2E2 | 0.79397266 | 1.85E-08 |
| ENSG00000153815 | CMIP | 0.794409658 | 4.3E-09 |
| ENSG00000173156 | RHOD | 0.794456904 | 0.00000483 |
| ENSG00000180385 | EMC3-AS1 | 0.794712286 | 0.005346984 |
| ENSG00000011478 | QPCTL | 0.794769823 | 0.0000206 |
| ENSG00000182774 | RPS17 | 0.79526321 | 0.007577884 |
| ENSG00000104497 | SNX16 | 0.795318292 | 0.00000207 |
| ENSG00000143753 | DEGS1 | 0.796104892 | 0.00000112 |
| ENSG00000224114 | AL591846.1 | 0.796215127 | 0.000493001 |
| ENSG00000137767 | SQOR | 0.796720238 | 0.0000993 |
| ENSG00000159147 | DONSON | 0.796895845 | 0.000128524 |
| ENSG00000165272 | AQP3 | 0.796896121 | 0.0000391 |
| ENSG00000102543 | CDADC1 | 0.797140369 | 0.00000175 |
| ENSG00000164626 | KCNK5 | 0.797667291 | 0.00000741 |
| ENSG00000183955 | KMT5A | 0.797713459 | 2.12E-08 |
| ENSG00000130479 | MAP1S | 0.797733805 | 3.25E-11 |
| ENSG00000169359 | SLC33A1 | 0.797830514 | 3.99E-15 |
| ENSG00000213315 | AL122020.1 | 0.798540869 | 0.000111367 |
| ENSG00000234753 | FOXP4-AS1 | 0.798795278 | 0.00230799 |
| ENSG00000121578 | B4GALT4 | 0.798925221 | 6.04E-08 |
| ENSG00000010818 | HIVEP2 | 0.799455784 | 0.000120579 |
| ENSG00000139946 | PELI2 | 0.799854749 | 0.00000446 |
| ENSG00000112812 | PRSS16 | 0.799870732 | 0.000162402 |
| ENSG00000143367 | TUFT1 | 0.799882598 | 4.03E-09 |
| ENSG00000115524 | SF3B1 | 0.80032919 | 1.14E-14 |
| ENSG00000171943 | SRGAP2C | 0.800458525 | 0.0000213 |
| ENSG00000106462 | EZH2 | 0.800504175 | 0.0000233 |
| ENSG00000181472 | ZBTB2 | 0.800923378 | 1.18E-12 |
| ENSG00000143514 | TP53BP2 | 0.801053613 | 6.61E-09 |
| ENSG00000100284 | TOM1 | 0.801461887 | 1.13E-09 |
| ENSG00000147065 | MSN | 0.801797673 | 7.12E-23 |
| ENSG00000134419 | RPS15A | 0.801799057 | 1.33E-10 |
| ENSG00000119888 | EPCAM | 0.802112574 | 7.17E-14 |
| ENSG00000162148 | PPP1R32 | 0.802456688 | 0.033144418 |
| ENSG00000063177 | RPL18 | 0.802732612 | 3.24E-13 |
| ENSG00000137747 | TMPRSS13 | 0.803696184 | 0.0000665 |
| ENSG00000154710 | RABGEF1 | 0.803731928 | 0.000352573 |
| ENSG00000087460 | GNAS | 0.803775105 | 2.73E-14 |
| ENSG00000136436 | CALCOCO2 | 0.803830151 | 2.15E-11 |
| ENSG00000107960 | STN1 | 0.804333341 | 0.000000384 |
| ENSG00000231500 | RPS18 | 0.804623091 | 0.030800195 |
| ENSG00000162878 | PKDCC | 0.804895611 | 0.0000189 |
| ENSG00000174804 | FZD4 | 0.805333613 | 0.0000893 |
| ENSG00000034510 | TMSB10 | 0.805340404 | 8.92E-12 |
| ENSG00000075413 | MARK3 | 0.806146227 | 1.73E-13 |
| ENSG00000224398 |  | 0.806661637 | 0.000026 |
| ENSG00000111897 | SERINC1 | 0.807224423 | 5.81E-19 |
| ENSG00000141452 | RMC1 | 0.807461282 | 1.42E-08 |
| ENSG00000241506 | PSMC1P1 | 0.807754454 | 0.039162098 |
| ENSG00000143324 | XPR1 | 0.807766863 | 4.36E-12 |
| ENSG00000168268 | NT5DC2 | 0.807787838 | 0.00000481 |
| ENSG00000130340 | SNX9 | 0.807886064 | 4.37E-15 |
| ENSG00000186806 | VSIG10L | 0.808039545 | 3.36E-09 |
| ENSG00000069966 | GNB5 | 0.808921886 | 1.07E-10 |
| ENSG00000130338 | TULP4 | 0.809655606 | 0.00000251 |
| ENSG00000128512 | DOCK4 | 0.809716201 | 0.000165229 |
| ENSG00000142192 | APP | 0.809847214 | 9.43E-21 |
| ENSG00000187257 | RSBN1L | 0.810067351 | 5.66E-12 |
| ENSG00000186162 | CIDECP | 0.810207472 | 0.001559403 |
| ENSG00000138018 | SELENOI | 0.810519798 | 1.84E-10 |
| ENSG00000169249 | ZRSR2 | 0.810580803 | 0.000000291 |
| ENSG00000105723 | GSK3A | 0.810617979 | 2.08E-11 |
| ENSG00000164542 | KIAA0895 | 0.810947441 | 0.000000474 |
| ENSG00000100483 | VCPKMT | 0.81119023 | 0.00000953 |
| ENSG00000151849 | CENPJ | 0.811437339 | 0.000426353 |
| ENSG00000226328 | NUP50-AS1 | 0.811690327 | 0.001784751 |
| ENSG00000125945 | ZNF436 | 0.812262413 | 0.000000855 |
| ENSG00000077585 | GPR137B | 0.81265555 | 0.000000583 |
| ENSG00000108107 | RPL28 | 0.812818337 | 7.86E-14 |
| ENSG00000151694 | ADAM17 | 0.812851216 | 0.000000476 |
| ENSG00000160336 | ZNF761 | 0.81296837 | 0.000000627 |
| ENSG00000161692 | DBF4B | 0.812971646 | 0.001111116 |
| ENSG00000104805 | NUCB1 | 0.812986574 | 1.09E-09 |
| ENSG00000166398 | KIAA0355 | 0.813375917 | 3.17E-08 |
| ENSG00000175573 | C11orf68 | 0.813620928 | 0.000000044 |
| ENSG00000131981 | LGALS3 | 0.813786366 | 1.7E-11 |
| ENSG00000115944 | COX7A2L | 0.813963097 | 2.36E-11 |
| ENSG00000130255 | RPL36 | 0.81404778 | 1.64E-10 |
| ENSG00000233230 | AC079807.1 | 0.814258528 | 0.01417706 |
| ENSG00000130734 | ATG4D | 0.814805809 | 0.000000119 |
| ENSG00000183444 | OR7E38P | 0.814944932 | 0.000014 |
| ENSG00000184863 | RBM33 | 0.8154452 | 0.000000907 |
| ENSG00000135362 | PRR5L | 0.815626455 | 1.47E-11 |
| ENSG00000179119 | SPTY2D1 | 0.81592104 | 1.44E-11 |
| ENSG00000102100 | SLC35A2 | 0.816134878 | 1.17E-08 |
| ENSG00000163516 | ANKZF1 | 0.816377474 | 5.67E-08 |
| ENSG00000148158 | SNX30 | 0.816595068 | 0.00000582 |
| ENSG00000068903 | SIRT2 | 0.816980186 | 1.14E-11 |
| ENSG00000260260 | SNHG19 | 0.817018984 | 0.000000133 |
| ENSG00000061936 | SFSWAP | 0.817187735 | 7.73E-15 |
| ENSG00000259295 | CSPG4P12 | 0.818245555 | 0.000356362 |
| ENSG00000105472 | CLEC11A | 0.818585568 | 0.00542032 |
| ENSG00000155158 | TTC39B | 0.818608695 | 0.00000724 |
| ENSG00000166582 | CENPV | 0.81874985 | 0.000000013 |
| ENSG00000246582 | AC100861.1 | 0.818931722 | 0.014643241 |
| ENSG00000225648 | SBDSP1 | 0.820017137 | 5.42E-08 |
| ENSG00000115216 | NRBP1 | 0.820395221 | 2.92E-11 |
| ENSG00000189227 | C15orf61 | 0.820641288 | 0.000150918 |
| ENSG00000226976 | COX6A1P2 | 0.820746715 | 0.029238607 |
| ENSG00000136240 | KDELR2 | 0.820931438 | 0.0000114 |
| ENSG00000134222 | PSRC1 | 0.820932547 | 0.000433377 |
| ENSG00000127922 | SEM1 | 0.820937803 | 2.22E-09 |
| ENSG00000227615 | AP001324.1 | 0.82133664 | 0.000194064 |
| ENSG00000169967 | MAP3K2 | 0.821374256 | 1.52E-16 |
| ENSG00000131389 | SLC6A6 | 0.821829638 | 0.0000493 |
| ENSG00000213741 | RPS29 | 0.821834279 | 4.19E-11 |
| ENSG00000225697 | SLC26A6 | 0.822009141 | 0.000000148 |
| ENSG00000151893 | CACUL1 | 0.822379978 | 2.15E-14 |
| ENSG00000140455 | USP3 | 0.82245446 | 2.82E-17 |
| ENSG00000149806 | FAU | 0.822456722 | 6.18E-12 |
| ENSG00000089177 | KIF16B | 0.822619006 | 4.6E-09 |
| ENSG00000127452 | FBXL12 | 0.822722136 | 2.39E-10 |
| ENSG00000198909 | MAP3K3 | 0.823425381 | 0.0000343 |
| ENSG00000006007 | GDE1 | 0.823941933 | 7.86E-11 |
| ENSG00000119655 | NPC2 | 0.824058874 | 4.74E-10 |
| ENSG00000275118 |  | 0.824727531 | 0.032010013 |
| ENSG00000162300 | ZFPL1 | 0.824963066 | 0.00000383 |
| ENSG00000104904 | OAZ1 | 0.82558921 | 7.47E-08 |
| ENSG00000214199 | EEF1A1P12 | 0.826125372 | 0.004795919 |
| ENSG00000116191 | RALGPS2 | 0.826339994 | 9.73E-12 |
| ENSG00000011243 | AKAP8L | 0.826418638 | 6.16E-13 |
| ENSG00000169567 | HINT1 | 0.826472396 | 2.26E-10 |
| ENSG00000135404 | CD63 | 0.826512099 | 8.03E-13 |
| ENSG00000184575 | XPOT | 0.826537183 | 8.2E-09 |
| ENSG00000177425 | PAWR | 0.826573629 | 1.18E-18 |
| ENSG00000168883 | USP39 | 0.826740832 | 0.000000311 |
| ENSG00000132963 | POMP | 0.827227398 | 0.000000165 |
| ENSG00000123352 | SPATS2 | 0.8275102 | 1.04E-15 |
| ENSG00000070081 | NUCB2 | 0.827539175 | 2.16E-08 |
| ENSG00000092445 | TYRO3 | 0.827564777 | 4.47E-08 |
| ENSG00000154359 | LONRF1 | 0.827571997 | 0.000517216 |
| ENSG00000198792 | TMEM184B | 0.827729984 | 4.2E-18 |
| ENSG00000167470 | MIDN | 0.828536687 | 3.15E-08 |
| ENSG00000225151 | GOLGA2P7 | 0.828703288 | 1.61E-13 |
| ENSG00000152242 | C18orf25 | 0.828722042 | 0.000000132 |
| ENSG00000205746 | AC126755.1 | 0.829093792 | 0.001062662 |
| ENSG00000227081 | AC005912.1 | 0.829389995 | 0.000116229 |
| ENSG00000164897 | TMUB1 | 0.830776055 | 1.14E-09 |
| ENSG00000213638 | ADAT3 | 0.830934267 | 0.013624227 |
| ENSG00000240854 | AC117409.1 | 0.831036738 | 0.032877177 |
| ENSG00000243829 | AC011495.1 | 0.831387114 | 8.17E-08 |
| ENSG00000197261 | C6orf141 | 0.831504855 | 0.0000158 |
| ENSG00000177888 | ZBTB41 | 0.832082432 | 0.00000191 |
| ENSG00000136003 | ISCU | 0.832671155 | 1.76E-09 |
| ENSG00000112742 | TTK | 0.832708481 | 0.001517547 |
| ENSG00000232346 | Z74021.1 | 0.832983539 | 6.07E-09 |
| ENSG00000023734 | STRAP | 0.83333908 | 2.23E-11 |
| ENSG00000225616 | AL138785.1 | 0.833857851 | 0.021396496 |
| ENSG00000171747 | LGALS4 | 0.835063878 | 5.16E-12 |
| ENSG00000198900 | TOP1 | 0.835440498 | 6.78E-22 |
| ENSG00000197157 | SND1 | 0.835533749 | 2.6E-20 |
| ENSG00000166340 | TPP1 | 0.836154225 | 2.63E-12 |
| ENSG00000184602 | SNN | 0.83632418 | 1.37E-13 |
| ENSG00000205534 | SMG1P2 | 0.83683484 | 0.000563143 |
| ENSG00000187961 | KLHL17 | 0.837453469 | 0.000000498 |
| ENSG00000090615 | GOLGA3 | 0.837564965 | 0.0000011 |
| ENSG00000233954 | UQCRHL | 0.838342473 | 0.00064013 |
| ENSG00000105640 | RPL18A | 0.838384304 | 1.44E-09 |
| ENSG00000123739 | PLA2G12A | 0.839135681 | 4.45E-14 |
| ENSG00000006704 | GTF2IRD1 | 0.839803176 | 2.7E-11 |
| ENSG00000228205 | AC131235.1 | 0.840416368 | 0.037153183 |
| ENSG00000245205 | EEF1A1P4 | 0.840895849 | 0.048816468 |
| ENSG00000232888 | RPS11P5 | 0.841033564 | 0.000140169 |
| ENSG00000112541 | PDE10A | 0.841499257 | 6.14E-12 |
| ENSG00000100852 | ARHGAP5 | 0.841649948 | 7.04E-11 |
| ENSG00000105193 | RPS16 | 0.841822795 | 1.8E-11 |
| ENSG00000119669 | IRF2BPL | 0.842374347 | 1.02E-11 |
| ENSG00000065911 | MTHFD2 | 0.842685862 | 0.0000017 |
| ENSG00000160072 | ATAD3B | 0.842745113 | 0.000000771 |
| ENSG00000197714 | ZNF460 | 0.843237398 | 0.000187123 |
| ENSG00000108840 | HDAC5 | 0.843284233 | 5.74E-21 |
| ENSG00000138767 | CNOT6L | 0.843474625 | 2.4E-16 |
| ENSG00000110888 | CAPRIN2 | 0.843864734 | 6.63E-08 |
| ENSG00000198604 | BAZ1A | 0.843950744 | 1.67E-11 |
| ENSG00000131871 | SELENOS | 0.844125853 | 1.99E-08 |
| ENSG00000218175 | AC016739.1 | 0.844587148 | 0.0000815 |
| ENSG00000104946 | TBC1D17 | 0.844709152 | 1.47E-16 |
| ENSG00000159461 | AMFR | 0.844798709 | 1.89E-12 |
| ENSG00000104221 | BRF2 | 0.845094879 | 0.004918032 |
| ENSG00000070770 | CSNK2A2 | 0.845218347 | 1.09E-20 |
| ENSG00000092531 | SNAP23 | 0.845727487 | 0.00000338 |
| ENSG00000170442 | KRT86 | 0.846079075 | 0.035590665 |
| ENSG00000066855 | MTFR1 | 0.846864459 | 2.28E-09 |
| ENSG00000019995 | ZRANB1 | 0.846975812 | 0.000000113 |
| ENSG00000111674 | ENO2 | 0.847368576 | 1.6E-14 |
| ENSG00000131446 | MGAT1 | 0.8473816 | 3.1E-10 |
| ENSG00000230629 | RPS23P8 | 0.847536706 | 0.000118434 |
| ENSG00000198478 | SH3BGRL2 | 0.8480147 | 1.08E-18 |
| ENSG00000117707 | PROX1 | 0.848048738 | 0.005486253 |
| ENSG00000102081 | FMR1 | 0.848183773 | 4.04E-15 |
| ENSG00000096696 | DSP | 0.848394745 | 0.00000031 |
| ENSG00000062194 | GPBP1 | 0.849365532 | 1.15E-19 |
| ENSG00000171960 | PPIH | 0.851400327 | 0.000185126 |
| ENSG00000148400 | NOTCH1 | 0.851547497 | 4.78E-09 |
| ENSG00000121481 | RNF2 | 0.85180338 | 4.74E-11 |
| ENSG00000270804 | AC010326.4 | 0.852100188 | 0.022701745 |
| ENSG00000110700 | RPS13 | 0.85219612 | 2.07E-15 |
| ENSG00000169442 | CD52 | 0.85283315 | 0.040851053 |
| ENSG00000235162 | C12orf75 | 0.852908032 | 3.83E-11 |
| ENSG00000141542 | RAB40B | 0.853338311 | 8.18E-11 |
| ENSG00000138757 | G3BP2 | 0.853734945 | 7.84E-14 |
| ENSG00000133138 | TBC1D8B | 0.854087396 | 2.73E-08 |
| ENSG00000125772 | GPCPD1 | 0.854097835 | 0.00468633 |
| ENSG00000166451 | CENPN | 0.854797867 | 0.000132054 |
| ENSG00000206341 | HLA-H | 0.855192464 | 0.034672842 |
| ENSG00000166136 | NDUFB8 | 0.856252669 | 0.00000364 |
| ENSG00000130707 | ASS1 | 0.856339821 | 0.000389457 |
| ENSG00000099250 | NRP1 | 0.856680732 | 2.75E-10 |
| ENSG00000014164 | ZC3H3 | 0.857091322 | 2.59E-08 |
| ENSG00000172273 | HINFP | 0.857416754 | 9.16E-13 |
| ENSG00000100417 | PMM1 | 0.857707673 | 0.00000513 |
| ENSG00000143321 | HDGF | 0.857971899 | 1.47E-12 |
| ENSG00000131100 | ATP6V1E1 | 0.859097174 | 1.24E-09 |
| ENSG00000008988 | RPS20 | 0.859388069 | 5.03E-20 |
| ENSG00000118620 | ZNF430 | 0.859415472 | 0.000000676 |
| ENSG00000241015 | TPM3P9 | 0.859451701 | 0.00000079 |
| ENSG00000172840 | PDP2 | 0.859815669 | 0.00000631 |
| ENSG00000218426 | AL590867.2 | 0.859932178 | 0.00000192 |
| ENSG00000260916 | CCPG1 | 0.861405064 | 0.000000146 |
| ENSG00000237506 | RPSAP15 | 0.861505052 | 0.000964375 |
| ENSG00000115170 | ACVR1 | 0.861630218 | 1.77E-17 |
| ENSG00000167378 | IRGQ | 0.861846192 | 0.00000345 |
| ENSG00000197021 | CXorf40B | 0.861973626 | 1.67E-09 |
| ENSG00000275131 | AC241952.1 | 0.862708427 | 0.011850485 |
| ENSG00000256393 | RPL41P5 | 0.862794567 | 0.00623444 |
| ENSG00000143457 | GOLPH3L | 0.863716649 | 0.000000106 |
| ENSG00000105991 | HOXA1 | 0.863825463 | 0.00000719 |
| ENSG00000140264 | SERF2 | 0.86402406 | 1.33E-11 |
| ENSG00000101096 | NFATC2 | 0.864913939 | 0.000465485 |
| ENSG00000071082 | RPL31 | 0.864966035 | 7.01E-18 |
| ENSG00000169032 | MAP2K1 | 0.865128229 | 3.29E-15 |
| ENSG00000197635 | DPP4 | 0.865265568 | 0.000127626 |
| ENSG00000065978 | YBX1 | 0.865803757 | 0.000000044 |
| ENSG00000214293 | APTR | 0.866374865 | 0.000000353 |
| ENSG00000213326 | RPS7P11 | 0.866569992 | 0.03157483 |
| ENSG00000148634 | HERC4 | 0.866585555 | 2.79E-11 |
| ENSG00000241484 | ARHGAP8 | 0.866875496 | 0.029781814 |
| ENSG00000173465 | SSSCA1 | 0.866916588 | 0.00000011 |
| ENSG00000198918 | RPL39 | 0.867312529 | 3.66E-13 |
| ENSG00000101665 | SMAD7 | 0.867882636 | 0.000000268 |
| ENSG00000103222 | ABCC1 | 0.867998293 | 0.00000106 |
| ENSG00000173692 | PSMD1 | 0.868261383 | 2.51E-14 |
| ENSG00000239305 | RNF103 | 0.868310736 | 4.01E-09 |
| ENSG00000115590 | IL1R2 | 0.869023803 | 0.008417246 |
| ENSG00000141367 | CLTC | 0.869218377 | 1.64E-14 |
| ENSG00000109854 | HTATIP2 | 0.869618172 | 2.69E-15 |
| ENSG00000198961 | PJA2 | 0.869642536 | 5.19E-10 |
| ENSG00000014914 | MTMR11 | 0.869989237 | 1.59E-12 |
| ENSG00000188064 | WNT7B | 0.870689421 | 3.55E-15 |
| ENSG00000198856 | OSTC | 0.871375945 | 4.44E-09 |
| ENSG00000177954 | RPS27 | 0.872125404 | 1.27E-17 |
| ENSG00000269743 | SLC25A53 | 0.872232268 | 0.000135026 |
| ENSG00000008323 | PLEKHG6 | 0.8723809 | 5.72E-11 |
| ENSG00000240370 | RPL13P5 | 0.872416462 | 0.000017 |
| ENSG00000274950 |  | 0.872659475 | 0.00000123 |
| ENSG00000111647 | UHRF1BP1L | 0.873851563 | 0.000000786 |
| ENSG00000169435 | RASSF6 | 0.873993096 | 1.3E-09 |
| ENSG00000171681 | ATF7IP | 0.875239364 | 9.11E-13 |
| ENSG00000105855 | ITGB8 | 0.875321118 | 2.97E-08 |
| ENSG00000164086 | DUSP7 | 0.875828966 | 5.4E-09 |
| ENSG00000187954 | CYHR1 | 0.876226026 | 6.35E-18 |
| ENSG00000243147 | MRPL33 | 0.87672451 | 3.23E-09 |
| ENSG00000139239 | RPL14P1 | 0.876789224 | 0.000128839 |
| ENSG00000158092 | NCK1 | 0.877072244 | 1.5E-10 |
| ENSG00000114770 | ABCC5 | 0.877422908 | 0.00000697 |
| ENSG00000102119 | EMD | 0.878167639 | 5.84E-08 |
| ENSG00000226221 | RPL26P19 | 0.87819998 | 0.004383921 |
| ENSG00000146083 | RNF44 | 0.878232547 | 3.3E-16 |
| ENSG00000158850 | B4GALT3 | 0.878235481 | 3.55E-15 |
| ENSG00000173113 | TRMT112 | 0.878626454 | 1.73E-08 |
| ENSG00000140374 | ETFA | 0.879217954 | 2.71E-14 |
| ENSG00000224699 | LAMTOR5-AS1 | 0.879377819 | 0.047822094 |
| ENSG00000166750 | SLFN5 | 0.880087867 | 0.00000217 |
| ENSG00000136802 | LRRC8A | 0.881859397 | 9.57E-21 |
| ENSG00000197943 | PLCG2 | 0.882159523 | 0.005266953 |
| ENSG00000188211 | NCR3LG1 | 0.882731351 | 0.00000208 |
| ENSG00000105656 | ELL | 0.883124658 | 3.56E-14 |
| ENSG00000230409 | TCEA1P2 | 0.883357711 | 0.0000289 |
| ENSG00000180867 | PDIA3P1 | 0.884522801 | 0.003072977 |
| ENSG00000249859 | PVT1 | 0.884551 | 2.04E-10 |
| ENSG00000010810 | FYN | 0.884939041 | 0.00000013 |
| ENSG00000011021 | CLCN6 | 0.885199121 | 8.95E-10 |
| ENSG00000138069 | RAB1A | 0.885883821 | 1.15E-12 |
| ENSG00000024048 | UBR2 | 0.886583482 | 2.58E-11 |
| ENSG00000102287 | GABRE | 0.886627695 | 0.0000625 |
| ENSG00000223463 |  | 0.886801152 | 0.004161747 |
| ENSG00000163947 | ARHGEF3 | 0.886878594 | 9.56E-15 |
| ENSG00000186174 | BCL9L | 0.887043553 | 4.36E-10 |
| ENSG00000187210 | GCNT1 | 0.887333987 | 0.003275719 |
| ENSG00000128272 | ATF4 | 0.887339685 | 1.55E-14 |
| ENSG00000161647 | MPP3 | 0.888033748 | 0.000157556 |
| ENSG00000109466 | KLHL2 | 0.889098759 | 4.08E-14 |
| ENSG00000213866 | YBX1P10 | 0.889314208 | 0.005345146 |
| ENSG00000132475 | H3F3B | 0.889797882 | 4.33E-12 |
| ENSG00000172613 | RAD9A | 0.88979861 | 0.00000125 |
| ENSG00000233476 | EEF1A1P6 | 0.890180668 | 0.00000369 |
| ENSG00000155744 | FAM126B | 0.890936262 | 1.11E-08 |
| ENSG00000141447 | OSBPL1A | 0.891492304 | 1.36E-16 |
| ENSG00000072042 | RDH11 | 0.891970798 | 7.02E-08 |
| ENSG00000112576 | CCND3 | 0.892633218 | 1.23E-11 |
| ENSG00000154370 | TRIM11 | 0.892824143 | 3.84E-13 |
| ENSG00000106541 | AGR2 | 0.892898684 | 0.00000239 |
| ENSG00000205542 | TMSB4X | 0.893103576 | 2.57E-18 |
| ENSG00000163359 | COL6A3 | 0.89395783 | 0.045824368 |
| ENSG00000029153 | ARNTL2 | 0.89439262 | 6.15E-08 |
| ENSG00000281406 | BLACAT1 | 0.894610211 | 0.004009656 |
| ENSG00000156011 | PSD3 | 0.894821376 | 3.28E-08 |
| ENSG00000109790 | KLHL5 | 0.895407818 | 0.00000169 |
| ENSG00000212802 | RPL15P3 | 0.896739486 | 0.0000967 |
| ENSG00000115839 | RAB3GAP1 | 0.897367738 | 5.83E-17 |
| ENSG00000131263 | RLIM | 0.897682163 | 6.59E-09 |
| ENSG00000077312 | SNRPA | 0.897996341 | 2.62E-14 |
| ENSG00000111540 | RAB5B | 0.898299155 | 1.03E-11 |
| ENSG00000173575 | CHD2 | 0.898582888 | 7.8E-10 |
| ENSG00000163468 | CCT3 | 0.899634144 | 1.65E-17 |
| ENSG00000142945 | KIF2C | 0.899868504 | 0.000193147 |
| ENSG00000175376 | EIF1AD | 0.900208878 | 2.11E-08 |
| ENSG00000156976 | EIF4A2 | 0.900313318 | 2.16E-15 |
| ENSG00000197780 | TAF13 | 0.901085207 | 9.23E-09 |
| ENSG00000167767 | KRT80 | 0.901138625 | 0.000000456 |
| ENSG00000249936 | RAC1P2 | 0.901771431 | 0.000120513 |
| ENSG00000106636 | YKT6 | 0.902037845 | 1.6E-10 |
| ENSG00000235174 | RPL39P3 | 0.902261675 | 0.00000377 |
| ENSG00000112763 | BTN2A1 | 0.902289447 | 1.08E-12 |
| ENSG00000153879 | CEBPG | 0.902487336 | 2.92E-10 |
| ENSG00000139517 | LNX2 | 0.902518114 | 1.16E-10 |
| ENSG00000172795 | DCP2 | 0.902669353 | 8.85E-11 |
| ENSG00000135480 | KRT7 | 0.903775275 | 0.001326059 |
| ENSG00000117450 | PRDX1 | 0.903951048 | 0.0000013 |
| ENSG00000282086 |  | 0.904089228 | 0.000358474 |
| ENSG00000185043 | CIB1 | 0.904550441 | 2.49E-09 |
| ENSG00000205423 | CNEP1R1 | 0.904632872 | 0.000056 |
| ENSG00000143622 | RIT1 | 0.905472051 | 3.61E-14 |
| ENSG00000162642 | C1orf52 | 0.906170789 | 3.71E-10 |
| ENSG00000166595 | FAM96B | 0.906478683 | 0.000000191 |
| ENSG00000138495 | COX17 | 0.907775251 | 0.000000167 |
| ENSG00000165655 | ZNF503 | 0.908092164 | 0.00000176 |
| ENSG00000104976 | SNAPC2 | 0.908652065 | 3.75E-12 |
| ENSG00000120686 | UFM1 | 0.908712259 | 2.03E-16 |
| ENSG00000185485 | SDHAP1 | 0.909969944 | 0.0000042 |
| ENSG00000114850 | SSR3 | 0.910113264 | 7.5E-13 |
| ENSG00000088280 | ASAP3 | 0.911097413 | 0.000168217 |
| ENSG00000143870 | PDIA6 | 0.911213965 | 0.000000407 |
| ENSG00000126458 | RRAS | 0.911538292 | 5.35E-12 |
| ENSG00000120254 | MTHFD1L | 0.912009515 | 3.97E-17 |
| ENSG00000062582 | MRPS24 | 0.913799955 | 0.038816385 |
| ENSG00000157483 | MYO1E | 0.913814273 | 5.03E-12 |
| ENSG00000142676 | RPL11 | 0.914384263 | 1.17E-20 |
| ENSG00000134057 | CCNB1 | 0.914485852 | 0.000000565 |
| ENSG00000165006 | UBAP1 | 0.914857084 | 2.14E-24 |
| ENSG00000187735 | TCEA1 | 0.915531226 | 1.49E-14 |
| ENSG00000149503 | INCENP | 0.915882121 | 0.017381789 |
| ENSG00000125868 | DSTN | 0.915911662 | 3.66E-12 |
| ENSG00000260114 | AC120114.2 | 0.916565069 | 0.005403281 |
| ENSG00000171813 | PWWP2B | 0.916636427 | 1.03E-15 |
| ENSG00000006194 | ZNF263 | 0.916736441 | 5.57E-18 |
| ENSG00000141646 | SMAD4 | 0.91676463 | 0.00000259 |
| ENSG00000226742 | HSBP1L1 | 0.916979299 | 9.17E-16 |
| ENSG00000181744 | C3orf58 | 0.917253829 | 0.000000475 |
| ENSG00000128294 | TPST2 | 0.918370103 | 0.00000143 |
| ENSG00000173120 | KDM2A | 0.919031261 | 7.09E-19 |
| ENSG00000065923 | SLC9A7 | 0.919222849 | 3.54E-14 |
| ENSG00000131051 | RBM39 | 0.91997672 | 4.89E-18 |
| ENSG00000115310 | RTN4 | 0.920332087 | 1.37E-24 |
| ENSG00000099917 | MED15 | 0.920379454 | 9.29E-18 |
| ENSG00000169972 | PUSL1 | 0.920841934 | 0.000000214 |
| ENSG00000276182 | AL163051.2 | 0.920950771 | 0.013188435 |
| ENSG00000036054 | TBC1D23 | 0.921125283 | 1.23E-08 |
| ENSG00000101367 | MAPRE1 | 0.921444335 | 5.52E-22 |
| ENSG00000214413 | BBIP1 | 0.921549547 | 2.33E-08 |
| ENSG00000157764 | BRAF | 0.922482809 | 3.89E-20 |
| ENSG00000170113 | NIPA1 | 0.924549148 | 9.44E-12 |
| ENSG00000198911 | SREBF2 | 0.925248473 | 6.15E-16 |
| ENSG00000164182 | NDUFAF2 | 0.92553057 | 8.12E-08 |
| ENSG00000084636 | COL16A1 | 0.926003554 | 0.00000815 |
| ENSG00000119280 | C1orf198 | 0.926441011 | 1.3E-16 |
| ENSG00000196141 | SPATS2L | 0.92736963 | 6.22E-12 |
| ENSG00000144747 | TMF1 | 0.928328218 | 1.18E-09 |
| ENSG00000257086 | AP001453.4 | 0.928360695 | 0.045969433 |
| ENSG00000013375 | PGM3 | 0.928588571 | 2.78E-09 |
| ENSG00000135297 | MTO1 | 0.928732627 | 3.37E-08 |
| ENSG00000137878 | GCOM1 | 0.929168224 | 0.0007547 |
| ENSG00000101844 | ATG4A | 0.930043518 | 7.51E-11 |
| ENSG00000086544 | ITPKC | 0.930117557 | 5.32E-13 |
| ENSG00000163577 | EIF5A2 | 0.930994566 | 2.23E-08 |
| ENSG00000123843 | C4BPB | 0.93154067 | 0.000625609 |
| ENSG00000078246 | TULP3 | 0.932026555 | 9.22E-10 |
| ENSG00000152642 | GPD1L | 0.932627567 | 4.67E-13 |
| ENSG00000234585 | CCT6P3 | 0.932760832 | 0.00000175 |
| ENSG00000142186 | SCYL1 | 0.933541531 | 2.8E-10 |
| ENSG00000162222 | TTC9C | 0.933597759 | 0.00000022 |
| ENSG00000162909 | CAPN2 | 0.934063229 | 2.96E-10 |
| ENSG00000121621 | KIF18A | 0.934272822 | 0.000000871 |
| ENSG00000105669 | COPE | 0.934303831 | 4.34E-11 |
| ENSG00000164099 | PRSS12 | 0.934533116 | 1.89E-12 |
| ENSG00000156110 | ADK | 0.934677131 | 1.34E-08 |
| ENSG00000196569 | LAMA2 | 0.934948222 | 0.039008657 |
| ENSG00000236540 | AC006547.1 | 0.93501266 | 0.0000186 |
| ENSG00000143862 | ARL8A | 0.935411074 | 2.16E-10 |
| ENSG00000231767 | AL136454.1 | 0.935448792 | 0.0070406 |
| ENSG00000097096 | SYDE2 | 0.93553832 | 0.000147272 |
| ENSG00000110330 | BIRC2 | 0.935670176 | 7.69E-19 |
| ENSG00000185753 | CXorf38 | 0.937240264 | 6.46E-11 |
| ENSG00000112208 | BAG2 | 0.937913449 | 1.27E-08 |
| ENSG00000076108 | BAZ2A | 0.938435496 | 2.39E-12 |
| ENSG00000112306 | RPS12 | 0.938890511 | 7.04E-17 |
| ENSG00000145247 | OCIAD2 | 0.939249816 | 1.06E-13 |
| ENSG00000128641 | MYO1B | 0.939558552 | 2.97E-08 |
| ENSG00000135334 | AKIRIN2 | 0.939668698 | 1.34E-13 |
| ENSG00000040487 | PQLC2 | 0.939752339 | 2.89E-10 |
| ENSG00000170677 | SOCS6 | 0.941790526 | 1.37E-24 |
| ENSG00000158411 | MITD1 | 0.942562477 | 2.35E-13 |
| ENSG00000198912 | C1orf174 | 0.942726194 | 0.0000279 |
| ENSG00000091651 | ORC6 | 0.943429797 | 0.000114414 |
| ENSG00000127774 | EMC6 | 0.943586701 | 0.001555414 |
| ENSG00000241990 | PRR34-AS1 | 0.943864505 | 0.015878516 |
| ENSG00000115163 | CENPA | 0.944507209 | 0.000137325 |
| ENSG00000133112 | TPT1 | 0.944814253 | 6.61E-15 |
| ENSG00000101928 | MOSPD1 | 0.94562944 | 3.66E-12 |
| ENSG00000150867 | PIP4K2A | 0.946342769 | 7.54E-13 |
| ENSG00000125505 | MBOAT7 | 0.94638686 | 0.011731944 |
| ENSG00000196850 | PPTC7 | 0.946633978 | 1.13E-22 |
| ENSG00000127191 | TRAF2 | 0.946877314 | 1.16E-09 |
| ENSG00000185551 | NR2F2 | 0.947010389 | 3.57E-15 |
| ENSG00000171858 | RPS21 | 0.947020594 | 1.31E-10 |
| ENSG00000078114 | NEBL | 0.948114311 | 0.000889627 |
| ENSG00000197712 | FAM114A1 | 0.948183085 | 9.77E-10 |
| ENSG00000120306 | CYSTM1 | 0.948334622 | 6.52E-16 |
| ENSG00000136048 | DRAM1 | 0.949094598 | 3.67E-09 |
| ENSG00000065054 | SLC9A3R2 | 0.950337499 | 1.34E-14 |
| ENSG00000183684 | ALYREF | 0.950843292 | 8.57E-08 |
| ENSG00000113558 | SKP1 | 0.951209964 | 6.44E-12 |
| ENSG00000117984 | CTSD | 0.951518184 | 1.19E-14 |
| ENSG00000131979 | GCH1 | 0.951808219 | 1.13E-14 |
| ENSG00000223482 | NUTM2A-AS1 | 0.952631859 | 3.64E-14 |
| ENSG00000163738 | MTHFD2L | 0.952943976 | 7.08E-10 |
| ENSG00000213442 | RPL18AP3 | 0.95308264 | 0.00000383 |
| ENSG00000124151 | NCOA3 | 0.953258802 | 1.61E-10 |
| ENSG00000058668 | ATP2B4 | 0.953552835 | 4.02E-17 |
| ENSG00000008394 | MGST1 | 0.953912232 | 1.35E-09 |
| ENSG00000188342 | GTF2F2 | 0.954073938 | 3.68E-20 |
| ENSG00000152518 | ZFP36L2 | 0.954449465 | 2.14E-12 |
| ENSG00000170412 | GPRC5C | 0.955364916 | 2.59E-14 |
| ENSG00000117505 | DR1 | 0.955478877 | 2.89E-18 |
| ENSG00000118939 | UCHL3 | 0.956041805 | 0.000000058 |
| ENSG00000230834 | AC068580.2 | 0.956058909 | 0.001990404 |
| ENSG00000007392 | LUC7L | 0.956304085 | 0.00000002 |
| ENSG00000198625 | MDM4 | 0.956708664 | 1.36E-17 |
| ENSG00000100644 | HIF1A | 0.956720322 | 1.43E-27 |
| ENSG00000119862 | LGALSL | 0.956905902 | 1.47E-08 |
| ENSG00000107130 | NCS1 | 0.957879787 | 9.5E-09 |
| ENSG00000152944 | MED21 | 0.957904905 | 1.6E-13 |
| ENSG00000206501 |  | 0.958038535 | 0.000490527 |
| ENSG00000133872 | SARAF | 0.958618415 | 1.56E-14 |
| ENSG00000198382 | UVRAG | 0.958863713 | 3.8E-22 |
| ENSG00000171604 | CXXC5 | 0.9596638 | 1.42E-15 |
| ENSG00000198435 | NRARP | 0.959795768 | 3.78E-16 |
| ENSG00000165233 | CARD19 | 0.960099564 | 5.62E-08 |
| ENSG00000196937 | FAM3C | 0.960450873 | 2.55E-18 |
| ENSG00000101199 | ARFGAP1 | 0.960772516 | 0.000000256 |
| ENSG00000151247 | EIF4E | 0.963006298 | 8.36E-10 |
| ENSG00000114739 | ACVR2B | 0.963130565 | 1.06E-11 |
| ENSG00000180773 | SLC36A4 | 0.963540021 | 0.0000265 |
| ENSG00000156735 | BAG4 | 0.964168496 | 0.000000511 |
| ENSG00000137409 | MTCH1 | 0.964438353 | 8.19E-10 |
| ENSG00000178821 | TMEM52 | 0.965564665 | 0.012972912 |
| ENSG00000123908 | AGO2 | 0.965912532 | 2.35E-12 |
| ENSG00000146067 | FAM193B | 0.966819989 | 9.88E-09 |
| ENSG00000272779 | AC245060.4 | 0.967162063 | 0.002035472 |
| ENSG00000047249 | ATP6V1H | 0.968064365 | 5.13E-16 |
| ENSG00000132950 | ZMYM5 | 0.969292596 | 3.73E-14 |
| ENSG00000115484 | CCT4 | 0.969302059 | 1.02E-14 |
| ENSG00000132849 | PATJ | 0.970027959 | 3.83E-19 |
| ENSG00000168564 | CDKN2AIP | 0.971030181 | 1.81E-16 |
| ENSG00000179456 | ZBTB18 | 0.971073711 | 9.94E-12 |
| ENSG00000214135 | AC132008.2 | 0.972045624 | 3.33E-10 |
| ENSG00000187164 | SHTN1 | 0.972205028 | 3.99E-26 |
| ENSG00000107242 | PIP5K1B | 0.973865447 | 8.66E-08 |
| ENSG00000156026 | MCU | 0.974583014 | 1.14E-10 |
| ENSG00000111665 | CDCA3 | 0.974851642 | 0.022221144 |
| ENSG00000240376 | AC010343.1 | 0.975580523 | 0.00245188 |
| ENSG00000133794 | ARNTL | 0.975746678 | 4.87E-14 |
| ENSG00000165410 | CFL2 | 0.975763568 | 0.0000432 |
| ENSG00000176845 | METRNL | 0.975768108 | 1.63E-10 |
| ENSG00000012232 | EXTL3 | 0.976999219 | 1.75E-09 |
| ENSG00000235986 |  | 0.977117387 | 0.003451021 |
| ENSG00000068697 | LAPTM4A | 0.978120048 | 6.16E-14 |
| ENSG00000011132 | APBA3 | 0.978873583 | 5.61E-08 |
| ENSG00000135870 | RC3H1 | 0.979224231 | 1.51E-13 |
| ENSG00000134910 | STT3A | 0.979277179 | 1.63E-14 |
| ENSG00000067082 | KLF6 | 0.979304632 | 2.12E-08 |
| ENSG00000163960 | UBXN7 | 0.97976981 | 8.47E-16 |
| ENSG00000147996 | CBWD5 | 0.979858689 | 9.28E-10 |
| ENSG00000104413 | ESRP1 | 0.980344937 | 1.47E-19 |
| ENSG00000112245 | PTP4A1 | 0.980660521 | 2.84E-16 |
| ENSG00000105829 | BET1 | 0.981355863 | 1.17E-13 |
| ENSG00000134324 | LPIN1 | 0.98156794 | 4.96E-11 |
| ENSG00000128524 | ATP6V1F | 0.981950096 | 1.21E-13 |
| ENSG00000140464 | PML | 0.982088775 | 1.07E-19 |
| ENSG00000156869 | FRRS1 | 0.982196062 | 3.07E-12 |
| ENSG00000267321 | LINC02001 | 0.982322797 | 0.000000252 |
| ENSG00000134375 | TIMM17A | 0.982837421 | 1.51E-11 |
| ENSG00000235065 | RPL24P2 | 0.983178587 | 0.007195695 |
| ENSG00000198826 | ARHGAP11A | 0.984780193 | 0.002413686 |
| ENSG00000015153 | YAF2 | 0.984824307 | 3.91E-13 |
| ENSG00000197594 | ENPP1 | 0.985188914 | 0.011549662 |
| ENSG00000158156 | XKR8 | 0.985880085 | 0.000000286 |
| ENSG00000169902 | TPST1 | 0.985949447 | 0.00360384 |
| ENSG00000117360 | PRPF3 | 0.986069714 | 2.55E-10 |
| ENSG00000165806 | CASP7 | 0.986930828 | 1.81E-12 |
| ENSG00000142002 | DPP9 | 0.986978074 | 1.22E-13 |
| ENSG00000175592 | FOSL1 | 0.98718163 | 0.047475172 |
| ENSG00000180628 | PCGF5 | 0.988375075 | 8.96E-13 |
| ENSG00000136026 | CKAP4 | 0.989534491 | 0.000000167 |
| ENSG00000187446 | CHP1 | 0.989697053 | 1.32E-22 |
| ENSG00000213867 | AL049830.1 | 0.989977286 | 0.038761327 |
| ENSG00000099326 | MZF1 | 0.992286817 | 8.2E-18 |
| ENSG00000164305 | CASP3 | 0.992696241 | 1.35E-13 |
| ENSG00000031698 | SARS | 0.993329129 | 2.88E-13 |
| ENSG00000160741 | CRTC2 | 0.993792185 | 8.72E-13 |
| ENSG00000104142 | VPS18 | 0.993834708 | 2.93E-18 |
| ENSG00000146242 | TPBG | 0.995526303 | 6.06E-11 |
| ENSG00000105409 | ATP1A3 | 0.995635115 | 0.015348351 |
| ENSG00000231834 |  | 0.996431125 | 8.49E-09 |
| ENSG00000186529 | CYP4F3 | 0.997408153 | 0.026270153 |
| ENSG00000253313 | C1orf210 | 0.997936086 | 2.11E-13 |
| ENSG00000213923 | CSNK1E | 0.998026854 | 3.47E-18 |
| ENSG00000185591 | SP1 | 1.000237115 | 5.63E-17 |
| ENSG00000187800 | PEAR1 | 1.000271114 | 0.001258758 |
| ENSG00000173039 | RELA | 1.000840326 | 2.08E-18 |
| ENSG00000167110 | GOLGA2 | 1.001662447 | 1.3E-12 |
| ENSG00000080371 | RAB21 | 1.001793225 | 1.66E-20 |
| ENSG00000146963 | LUC7L2 | 1.002261133 | 1.49E-14 |
| ENSG00000173917 | HOXB2 | 1.002999838 | 0.0000174 |
| ENSG00000133477 | FAM83F | 1.003153291 | 1.53E-08 |
| ENSG00000159884 | CCDC107 | 1.003413198 | 0.000181449 |
| ENSG00000167004 | PDIA3 | 1.003480613 | 1.23E-13 |
| ENSG00000152700 | SAR1B | 1.003877159 | 0.000000116 |
| ENSG00000173239 | LIPM | 1.003890707 | 0.013124535 |
| ENSG00000164663 | USP49 | 1.00404503 | 7.68E-08 |
| ENSG00000177150 | FAM210A | 1.004094818 | 1.15E-14 |
| ENSG00000128346 | C22orf23 | 1.004589538 | 0.00966267 |
| ENSG00000083444 | PLOD1 | 1.005520897 | 1.81E-10 |
| ENSG00000221869 | CEBPD | 1.005840582 | 7.47E-17 |
| ENSG00000197019 | SERTAD1 | 1.006013895 | 0.00000315 |
| ENSG00000101236 | RNF24 | 1.006560728 | 7.52E-12 |
| ENSG00000211445 | GPX3 | 1.00712435 | 1.38E-09 |
| ENSG00000007047 | MARK4 | 1.007125435 | 3.15E-20 |
| ENSG00000154429 | CCSAP | 1.00715262 | 0.000000786 |
| ENSG00000269028 | MTRNR2L12 | 1.008530484 | 0.0000402 |
| ENSG00000137936 | BCAR3 | 1.009076732 | 9.18E-19 |
| ENSG00000196705 | ZNF431 | 1.010671675 | 4.08E-09 |
| ENSG00000128342 | LIF | 1.011853299 | 0.000000866 |
| ENSG00000183604 | SMG1P5 | 1.012799939 | 0.00380666 |
| ENSG00000173889 | PHC3 | 1.013196302 | 2.23E-08 |
| ENSG00000278978 | AC092611.3 | 1.013883712 | 0.011810088 |
| ENSG00000102098 | SCML2 | 1.014431259 | 0.00000621 |
| ENSG00000184014 | DENND5A | 1.014677532 | 5.91E-13 |
| ENSG00000171097 | KYAT1 | 1.014832579 | 0.000034 |
| ENSG00000131470 | PSMC3IP | 1.015687283 | 0.0000275 |
| ENSG00000163682 | RPL9 | 1.015819838 | 3.99E-12 |
| ENSG00000106153 | CHCHD2 | 1.016543973 | 8.82E-10 |
| ENSG00000109089 | CDR2L | 1.017519383 | 0.0000034 |
| ENSG00000162889 | MAPKAPK2 | 1.017563424 | 3.42E-23 |
| ENSG00000188215 | DCUN1D3 | 1.017975411 | 8.29E-11 |
| ENSG00000234648 | AL162151.2 | 1.018950294 | 0.021037207 |
| ENSG00000070759 | TESK2 | 1.019494123 | 0.000017 |
| ENSG00000185436 | IFNLR1 | 1.0196933 | 0.000164931 |
| ENSG00000125733 | TRIP10 | 1.021137799 | 3.53E-18 |
| ENSG00000114023 | FAM162A | 1.021289315 | 2.94E-13 |
| ENSG00000175471 | MCTP1 | 1.021472879 | 2.61E-09 |
| ENSG00000101782 | RIOK3 | 1.021933696 | 2.08E-19 |
| ENSG00000113504 | SLC12A7 | 1.024838751 | 2.5E-12 |
| ENSG00000119777 | TMEM214 | 1.024840561 | 0.000000323 |
| ENSG00000231312 | AC007388.1 | 1.024913106 | 0.00000803 |
| ENSG00000020577 | SAMD4A | 1.025439077 | 3.11E-11 |
| ENSG00000228887 | EEF1DP1 | 1.02569531 | 0.0000106 |
| ENSG00000039523 | RIPOR1 | 1.025741193 | 1.08E-17 |
| ENSG00000175931 | UBE2O | 1.025749106 | 4.14E-24 |
| ENSG00000072778 | ACADVL | 1.026983981 | 2.98E-17 |
| ENSG00000172943 | PHF8 | 1.027394332 | 4.14E-11 |
| ENSG00000173530 | TNFRSF10D | 1.029270984 | 7.6E-09 |
| ENSG00000140511 | HAPLN3 | 1.029349129 | 0.022245286 |
| ENSG00000140320 | BAHD1 | 1.029672109 | 4.67E-13 |
| ENSG00000147642 | SYBU | 1.029838451 | 1.29E-09 |
| ENSG00000113448 | PDE4D | 1.031113731 | 4.83E-13 |
| ENSG00000101493 | ZNF516 | 1.032581447 | 1.29E-15 |
| ENSG00000057019 | DCBLD2 | 1.032911381 | 5.2E-27 |
| ENSG00000162928 | PEX13 | 1.033350576 | 1.14E-15 |
| ENSG00000151746 | BICD1 | 1.033421651 | 1.63E-20 |
| ENSG00000181467 | RAP2B | 1.033530639 | 5.37E-20 |
| ENSG00000091136 | LAMB1 | 1.034353763 | 1.94E-13 |
| ENSG00000213533 | STIMATE | 1.035359231 | 0.019732079 |
| ENSG00000182831 | C16orf72 | 1.035911321 | 1.05E-19 |
| ENSG00000278183 |  | 1.036134978 | 0.00000273 |
| ENSG00000203288 | TDRKH-AS1 | 1.03658768 | 0.001769534 |
| ENSG00000124588 | NQO2 | 1.036842963 | 4.88E-16 |
| ENSG00000205744 | DENND1C | 1.037970279 | 3.39E-10 |
| ENSG00000170540 | ARL6IP1 | 1.038056769 | 1.08E-08 |
| ENSG00000106258 | CYP3A5 | 1.038095638 | 1.11E-10 |
| ENSG00000143761 | ARF1 | 1.038576866 | 3.82E-13 |
| ENSG00000172115 | CYCS | 1.039195929 | 1.04E-12 |
| ENSG00000249855 | EEF1A1P19 | 1.039937799 | 0.0000241 |
| ENSG00000100380 | ST13 | 1.03994577 | 4.81E-20 |
| ENSG00000170385 | SLC30A1 | 1.040117384 | 7.37E-11 |
| ENSG00000127022 | CANX | 1.04038649 | 1.1E-26 |
| ENSG00000151914 | DST | 1.040643924 | 9.17E-08 |
| ENSG00000119729 | RHOQ | 1.040846992 | 1.74E-10 |
| ENSG00000239306 | RBM14 | 1.041405652 | 7.53E-11 |
| ENSG00000120910 | PPP3CC | 1.041618189 | 0.00000119 |
| ENSG00000114354 | TFG | 1.041959753 | 5.53E-18 |
| ENSG00000149925 | ALDOA | 1.04221793 | 7.96E-11 |
| ENSG00000109511 | ANXA10 | 1.042391978 | 2.55E-12 |
| ENSG00000176142 | TMEM39A | 1.042540883 | 6.67E-20 |
| ENSG00000007944 | MYLIP | 1.042607043 | 3.63E-11 |
| ENSG00000188833 | ENTPD8 | 1.042755346 | 0.037696614 |
| ENSG00000103855 | CD276 | 1.044160519 | 6.08E-22 |
| ENSG00000245970 | AP003352.1 | 1.044321973 | 0.020298764 |
| ENSG00000166145 | SPINT1 | 1.044376507 | 2.26E-25 |
| ENSG00000051128 | HOMER3 | 1.044489782 | 0.000210448 |
| ENSG00000129245 | FXR2 | 1.044819716 | 2.06E-16 |
| ENSG00000135932 | CAB39 | 1.045068002 | 1.53E-32 |
| ENSG00000228158 | TLE1P1 | 1.045728161 | 0.029175965 |
| ENSG00000122515 | ZMIZ2 | 1.046160701 | 3.56E-14 |
| ENSG00000274173 | AL035661.1 | 1.046557703 | 3.62E-15 |
| ENSG00000124788 | ATXN1 | 1.047272282 | 2.47E-16 |
| ENSG00000101447 | FAM83D | 1.04759822 | 0.004216304 |
| ENSG00000182934 | SRPRA | 1.047858326 | 1.35E-09 |
| ENSG00000282641 |  | 1.04822441 | 0.00000904 |
| ENSG00000182287 | AP1S2 | 1.048379388 | 4.95E-09 |
| ENSG00000129911 | KLF16 | 1.048557986 | 7.2E-17 |
| ENSG00000112033 | PPARD | 1.048663531 | 1.04E-21 |
| ENSG00000082641 | NFE2L1 | 1.048857891 | 7.75E-21 |
| ENSG00000105649 | RAB3A | 1.049416311 | 0.000586821 |
| ENSG00000101210 | EEF1A2 | 1.049482433 | 3.03E-11 |
| ENSG00000019505 | SYT13 | 1.050515853 | 0.00000286 |
| ENSG00000259976 | AC093010.3 | 1.051774688 | 0.014468877 |
| ENSG00000119318 | RAD23B | 1.051933597 | 6.54E-36 |
| ENSG00000197978 | GOLGA6L9 | 1.05211947 | 0.0000817 |
| ENSG00000168488 | ATXN2L | 1.053795806 | 7.41E-18 |
| ENSG00000159873 | CCDC117 | 1.054608332 | 0.00000001 |
| ENSG00000178104 | PDE4DIP | 1.056007818 | 0.000000604 |
| ENSG00000133226 | SRRM1 | 1.056547742 | 1.24E-11 |
| ENSG00000134317 | GRHL1 | 1.057283584 | 1.12E-09 |
| ENSG00000165568 | AKR1E2 | 1.057321432 | 0.000102878 |
| ENSG00000077152 | UBE2T | 1.057375561 | 0.021309331 |
| ENSG00000035862 | TIMP2 | 1.05807834 | 3.33E-10 |
| ENSG00000143190 | POU2F1 | 1.058330273 | 3.48E-15 |
| ENSG00000253320 | AZIN1-AS1 | 1.058348136 | 0.00808351 |
| ENSG00000175104 | TRAF6 | 1.058629625 | 2.45E-15 |
| ENSG00000197961 | ZNF121 | 1.058867927 | 2.63E-15 |
| ENSG00000109083 | IFT20 | 1.059233379 | 1.63E-09 |
| ENSG00000205413 | SAMD9 | 1.060234129 | 0.031417604 |
| ENSG00000160201 | U2AF1 | 1.061850658 | 0.000000715 |
| ENSG00000186007 | LEMD1 | 1.063301164 | 0.000560153 |
| ENSG00000166889 | PATL1 | 1.064663402 | 3.99E-13 |
| ENSG00000139734 | DIAPH3 | 1.064908675 | 4.35E-11 |
| ENSG00000167755 | KLK6 | 1.065667827 | 2.68E-19 |
| ENSG00000170312 | CDK1 | 1.065940538 | 0.003093814 |
| ENSG00000227715 |  | 1.066475932 | 0.000239334 |
| ENSG00000170919 | TPT1-AS1 | 1.067153036 | 0.0000567 |
| ENSG00000261578 | AP003119.3 | 1.07013871 | 0.000365119 |
| ENSG00000074410 | CA12 | 1.070763889 | 2.54E-24 |
| ENSG00000161013 | MGAT4B | 1.071214219 | 1.88E-19 |
| ENSG00000075785 | RAB7A | 1.07154446 | 1.13E-31 |
| ENSG00000178184 | PARD6G | 1.072506138 | 0.021219699 |
| ENSG00000271614 | ATP2B1-AS1 | 1.072944068 | 0.031235475 |
| ENSG00000181019 | NQO1 | 1.072995812 | 8.58E-23 |
| ENSG00000136155 | SCEL | 1.073738441 | 0.00018491 |
| ENSG00000273814 |  | 1.074084795 | 4.16E-13 |
| ENSG00000197885 | NKIRAS1 | 1.074325854 | 0.00000577 |
| ENSG00000186432 | KPNA4 | 1.074562636 | 1.72E-28 |
| ENSG00000107140 | TESK1 | 1.074824038 | 5.4E-10 |
| ENSG00000142910 | TINAGL1 | 1.076064539 | 4.83E-15 |
| ENSG00000237984 | PTENP1 | 1.076140256 | 0.015453449 |
| ENSG00000114315 | HES1 | 1.076399378 | 1.37E-11 |
| ENSG00000174021 | GNG5 | 1.076589108 | 3.82E-16 |
| ENSG00000008324 | SS18L2 | 1.076786237 | 2.11E-10 |
| ENSG00000127481 | UBR4 | 1.076898934 | 5.4E-09 |
| ENSG00000147044 | CASK | 1.076976021 | 1.54E-25 |
| ENSG00000057657 | PRDM1 | 1.077345667 | 0.003136453 |
| ENSG00000083312 | TNPO1 | 1.079360285 | 8.54E-23 |
| ENSG00000146670 | CDCA5 | 1.079655972 | 0.006397558 |
| ENSG00000134108 | ARL8B | 1.079759224 | 2.28E-19 |
| ENSG00000197822 | OCLN | 1.079994163 | 2.92E-09 |
| ENSG00000155304 | HSPA13 | 1.080273515 | 3.19E-19 |
| ENSG00000227008 | AL009174.1 | 1.080849213 | 0.018314984 |
| ENSG00000175155 | YPEL2 | 1.08176686 | 4.5E-16 |
| ENSG00000224597 | SVIL-AS1 | 1.08281675 | 0.0000169 |
| ENSG00000137474 | MYO7A | 1.083596882 | 0.00000563 |
| ENSG00000198960 | ARMCX6 | 1.086205493 | 3.88E-09 |
| ENSG00000170315 | UBB | 1.086723835 | 3.1E-15 |
| ENSG00000172780 | RAB43 | 1.087040503 | 0.000000132 |
| ENSG00000175416 | CLTB | 1.087135407 | 1.69E-19 |
| ENSG00000133858 | ZFC3H1 | 1.087445517 | 7.82E-24 |
| ENSG00000124209 | RAB22A | 1.087539278 | 1.68E-31 |
| ENSG00000117500 | TMED5 | 1.087681354 | 6.38E-16 |
| ENSG00000126705 | AHDC1 | 1.087865542 | 3.15E-16 |
| ENSG00000196781 | TLE1 | 1.08805087 | 2.53E-23 |
| ENSG00000161960 | EIF4A1 | 1.088128189 | 0.000454702 |
| ENSG00000154608 | CEP170P1 | 1.088128532 | 0.028815065 |
| ENSG00000002586 | CD99 | 1.088456126 | 0.003253002 |
| ENSG00000120053 | GOT1 | 1.088787763 | 1.92E-12 |
| ENSG00000099194 | SCD | 1.08911432 | 5.53E-14 |
| ENSG00000247700 |  | 1.089926221 | 1.51E-10 |
| ENSG00000177169 | ULK1 | 1.090221876 | 4.07E-12 |
| ENSG00000103316 | CRYM | 1.090276638 | 0.00015499 |
| ENSG00000104695 | PPP2CB | 1.090586582 | 1.24E-20 |
| ENSG00000123395 | ATG101 | 1.091571474 | 3.17E-15 |
| ENSG00000075426 | FOSL2 | 1.092152971 | 5.01E-17 |
| ENSG00000278882 |  | 1.092215426 | 0.001435865 |
| ENSG00000125398 | SOX9 | 1.092735096 | 3.51E-16 |
| ENSG00000277998 | AC107075.1 | 1.092869913 | 0.004116214 |
| ENSG00000113734 | BNIP1 | 1.093116812 | 2.7E-11 |
| ENSG00000126787 | DLGAP5 | 1.093170663 | 7.54E-08 |
| ENSG00000099804 | CDC34 | 1.093320891 | 2.44E-11 |
| ENSG00000203993 | ARRDC1-AS1 | 1.093899882 | 1.4E-13 |
| ENSG00000184678 | HIST2H2BE | 1.094606273 | 0.000418736 |
| ENSG00000162236 | STX5 | 1.094892226 | 9.61E-11 |
| ENSG00000115239 | ASB3 | 1.095140067 | 0.0000684 |
| ENSG00000215717 | TMEM167B | 1.095397263 | 1.39E-11 |
| ENSG00000168894 | RNF181 | 1.097304854 | 1.82E-14 |
| ENSG00000147854 | UHRF2 | 1.099756669 | 5.97E-14 |
| ENSG00000164104 | HMGB2 | 1.099873318 | 1.09E-09 |
| ENSG00000129083 | COPB1 | 1.100437145 | 7.92E-18 |
| ENSG00000188643 | S100A16 | 1.100676197 | 1.53E-14 |
| ENSG00000249456 | AL731577.2 | 1.101249321 | 0.002926111 |
| ENSG00000275234 | AC010503.4 | 1.101650411 | 2.33E-12 |
| ENSG00000184009 | ACTG1 | 1.102319326 | 1.98E-16 |
| ENSG00000143320 | CRABP2 | 1.102734124 | 2.75E-12 |
| ENSG00000269893 | SNHG8 | 1.103629886 | 5.35E-18 |
| ENSG00000163251 | FZD5 | 1.104214297 | 3.54E-19 |
| ENSG00000188459 | WASF4P | 1.104248048 | 0.029095811 |
| ENSG00000147454 | SLC25A37 | 1.104361999 | 1.61E-15 |
| ENSG00000202337 | RNU6-8 | 1.104589409 | 0.040757888 |
| ENSG00000183506 | PI4KAP2 | 1.104669484 | 0.000000376 |
| ENSG00000100290 | BIK | 1.104921282 | 0.000331097 |
| ENSG00000142657 | PGD | 1.104949428 | 1.1E-22 |
| ENSG00000127314 | RAP1B | 1.104954151 | 1.55E-24 |
| ENSG00000185624 | P4HB | 1.105342701 | 3.96E-17 |
| ENSG00000171132 | PRKCE | 1.105745407 | 2.27E-17 |
| ENSG00000093167 | LRRFIP2 | 1.10631542 | 7.05E-16 |
| ENSG00000198060 | 44260 | 1.106556561 | 7.05E-33 |
| ENSG00000164221 | CCDC112 | 1.106802128 | 5.55E-13 |
| ENSG00000282276 |  | 1.107143928 | 0.021608686 |
| ENSG00000187653 | TMSB4XP8 | 1.107782136 | 0.017069506 |
| ENSG00000159479 | MED8 | 1.108484071 | 1.62E-10 |
| ENSG00000155096 | AZIN1 | 1.108745071 | 4.23E-21 |
| ENSG00000130821 | SLC6A8 | 1.10886273 | 1.65E-13 |
| ENSG00000197119 | SLC25A29 | 1.109745891 | 5.25E-13 |
| ENSG00000104369 | JPH1 | 1.109782557 | 0.00000366 |
| ENSG00000104824 | HNRNPL | 1.110493113 | 8.5E-13 |
| ENSG00000197329 | PELI1 | 1.110768784 | 2.34E-17 |
| ENSG00000135338 | LCA5 | 1.111033232 | 0.000000122 |
| ENSG00000150991 | UBC | 1.111509455 | 1.01E-19 |
| ENSG00000246273 | SBF2-AS1 | 1.112168018 | 0.0000393 |
| ENSG00000094804 | CDC6 | 1.112530569 | 5.35E-08 |
| ENSG00000176018 | LYSMD3 | 1.113501268 | 5.81E-13 |
| ENSG00000206505 |  | 1.114145335 | 0.000000389 |
| ENSG00000259623 | AC125257.1 | 1.115418131 | 3.4E-10 |
| ENSG00000237599 |  | 1.115460959 | 0.029368133 |
| ENSG00000110395 | CBL | 1.115875683 | 1.09E-16 |
| ENSG00000166439 | RNF169 | 1.115902058 | 0.000000154 |
| ENSG00000164105 | SAP30 | 1.116689003 | 0.00000192 |
| ENSG00000117479 | SLC19A2 | 1.116796491 | 2.29E-08 |
| ENSG00000134248 | LAMTOR5 | 1.116970407 | 2.47E-10 |
| ENSG00000189143 | CLDN4 | 1.117301288 | 3.29E-24 |
| ENSG00000196369 | SRGAP2B | 1.117340735 | 4.19E-12 |
| ENSG00000133985 | TTC9 | 1.117391732 | 0.004913355 |
| ENSG00000241360 | PDXP | 1.11780543 | 0.00000606 |
| ENSG00000226354 |  | 1.119086922 | 0.000000657 |
| ENSG00000115756 | HPCAL1 | 1.119142151 | 6.48E-19 |
| ENSG00000173451 | THAP2 | 1.119600095 | 1.21E-08 |
| ENSG00000104863 | LIN7B | 1.12007458 | 0.000012 |
| ENSG00000116017 | ARID3A | 1.120199601 | 5.57E-18 |
| ENSG00000171735 | CAMTA1 | 1.121045868 | 5.82E-15 |
| ENSG00000196083 | IL1RAP | 1.121551054 | 2.58E-11 |
| ENSG00000145386 | CCNA2 | 1.122562627 | 0.013344854 |
| ENSG00000130545 | CRB3 | 1.122790199 | 7.64E-23 |
| ENSG00000070404 | FSTL3 | 1.12440254 | 0.000000065 |
| ENSG00000122884 | P4HA1 | 1.124577636 | 2.16E-16 |
| ENSG00000163898 | LIPH | 1.125040472 | 7.22E-20 |
| ENSG00000105085 | MED26 | 1.125142148 | 2.43E-08 |
| ENSG00000204842 | ATXN2 | 1.125386065 | 1.97E-24 |
| ENSG00000189325 | C6orf222 | 1.129677669 | 0.030519164 |
| ENSG00000126012 | KDM5C | 1.130500655 | 4.84E-28 |
| ENSG00000158195 | WASF2 | 1.131016898 | 4.44E-32 |
| ENSG00000123485 | HJURP | 1.13103982 | 0.005509994 |
| ENSG00000085063 | CD59 | 1.131125961 | 9.94E-35 |
| ENSG00000167604 | NFKBID | 1.131381153 | 0.00000064 |
| ENSG00000172530 | BANP | 1.131423844 | 5.48E-11 |
| ENSG00000135926 | TMBIM1 | 1.13196687 | 2.38E-32 |
| ENSG00000099942 | CRKL | 1.132630019 | 7.15E-12 |
| ENSG00000221995 | TIAF1 | 1.133324305 | 0.032417572 |
| ENSG00000128487 | SPECC1 | 1.133466871 | 1.47E-22 |
| ENSG00000166128 | RAB8B | 1.133814937 | 5.52E-13 |
| ENSG00000213347 | MXD3 | 1.133903989 | 0.009006826 |
| ENSG00000142961 | MOB3C | 1.13457317 | 4.67E-11 |
| ENSG00000230979 | AC079250.1 | 1.135444402 | 0.00000762 |
| ENSG00000125875 | TBC1D20 | 1.136047068 | 8.18E-20 |
| ENSG00000006634 | DBF4 | 1.136053614 | 0.001927727 |
| ENSG00000231259 | AC125232.1 | 1.136107367 | 0.000000343 |
| ENSG00000276924 |  | 1.136174027 | 0.036813865 |
| ENSG00000075702 | WDR62 | 1.136182048 | 0.005416901 |
| ENSG00000103121 | CMC2 | 1.136190871 | 0.000000108 |
| ENSG00000121749 | TBC1D15 | 1.136813147 | 3.14E-11 |
| ENSG00000273038 | AL365203.2 | 1.13687357 | 0.040309031 |
| ENSG00000279427 | AC007490.1 | 1.137544805 | 0.00881258 |
| ENSG00000245910 | SNHG6 | 1.137748167 | 1.22E-22 |
| ENSG00000278616 | BEND3P3 | 1.139114351 | 0.012475722 |
| ENSG00000274605 | AL355338.1 | 1.139386552 | 3.7E-09 |
| ENSG00000143387 | CTSK | 1.140204164 | 0.000270569 |
| ENSG00000196924 | FLNA | 1.140318928 | 1.03E-11 |
| ENSG00000188015 | S100A3 | 1.140711262 | 0.02741723 |
| ENSG00000257704 | INAFM1 | 1.140787769 | 9.69E-09 |
| ENSG00000260196 | AC124798.1 | 1.14110929 | 0.000215849 |
| ENSG00000277879 | AL391988.1 | 1.141170166 | 0.003956913 |
| ENSG00000137571 | SLCO5A1 | 1.141868132 | 0.015093528 |
| ENSG00000105321 | CCDC9 | 1.14205494 | 2.76E-11 |
| ENSG00000162959 | MEMO1 | 1.142208055 | 2.42E-08 |
| ENSG00000102096 | PIM2 | 1.142360192 | 1.05E-10 |
| ENSG00000107404 | DVL1 | 1.142542219 | 3.08E-18 |
| ENSG00000144566 | RAB5A | 1.142631194 | 3.06E-25 |
| ENSG00000151553 | FAM160B1 | 1.145848408 | 1.36E-13 |
| ENSG00000240849 | TMEM189 | 1.145930311 | 2.29E-19 |
| ENSG00000129657 | SEC14L1 | 1.145961917 | 9.72E-26 |
| ENSG00000269858 | EGLN2 | 1.146032944 | 1.88E-15 |
| ENSG00000160285 | LSS | 1.147774098 | 4.17E-09 |
| ENSG00000263563 | AC138761.1 | 1.149075882 | 0.005794874 |
| ENSG00000089775 | ZBTB25 | 1.149148704 | 2.22E-12 |
| ENSG00000163291 | PAQR3 | 1.149942283 | 3.06E-13 |
| ENSG00000169710 | FASN | 1.150512355 | 5.33E-11 |
| ENSG00000106829 | TLE4 | 1.150726293 | 6.43E-10 |
| ENSG00000159885 | ZNF222 | 1.151361887 | 0.000000138 |
| ENSG00000162738 | VANGL2 | 1.151921738 | 4.22E-10 |
| ENSG00000145860 | RNF145 | 1.152255793 | 4.25E-27 |
| ENSG00000186300 | ZNF555 | 1.152540354 | 0.00000281 |
| ENSG00000105376 | ICAM5 | 1.152874169 | 0.013466974 |
| ENSG00000134285 | FKBP11 | 1.152972704 | 8.4E-09 |
| ENSG00000227366 |  | 1.153779246 | 0.02944891 |
| ENSG00000075420 | FNDC3B | 1.154218473 | 1.06E-20 |
| ENSG00000166900 | STX3 | 1.154526281 | 8.64E-25 |
| ENSG00000196754 | S100A2 | 1.155852372 | 7.65E-09 |
| ENSG00000166471 | TMEM41B | 1.156494653 | 2.43E-13 |
| ENSG00000203879 | GDI1 | 1.157588232 | 1.92E-31 |
| ENSG00000164687 | FABP5 | 1.157941596 | 0.000000158 |
| ENSG00000255182 | AC084125.2 | 1.158106405 | 0.019049276 |
| ENSG00000100068 | LRP5L | 1.158675281 | 0.00000191 |
| ENSG00000102580 | DNAJC3 | 1.15870888 | 7.72E-15 |
| ENSG00000178980 | SELENOW | 1.158803556 | 7.83E-09 |
| ENSG00000148346 | LCN2 | 1.158868089 | 2.79E-17 |
| ENSG00000254893 | AC113404.3 | 1.160522642 | 0.00000699 |
| ENSG00000148835 | TAF5 | 1.162095142 | 0.0000258 |
| ENSG00000078804 | TP53INP2 | 1.162333421 | 1.13E-15 |
| ENSG00000006652 | IFRD1 | 1.162424845 | 1.56E-11 |
| ENSG00000182534 | MXRA7 | 1.162465541 | 0.000199083 |
| ENSG00000172766 | NAA16 | 1.163448141 | 1.51E-13 |
| ENSG00000197182 | MIRLET7BHG | 1.163578013 | 0.000141052 |
| ENSG00000104880 | ARHGEF18 | 1.164102347 | 7.05E-12 |
| ENSG00000128567 | PODXL | 1.164154189 | 2.47E-19 |
| ENSG00000129467 | ADCY4 | 1.16586449 | 0.001215722 |
| ENSG00000229215 |  | 1.167131055 | 0.001099856 |
| ENSG00000137804 | NUSAP1 | 1.167491978 | 0.000000139 |
| ENSG00000105287 | PRKD2 | 1.168256759 | 3.72E-32 |
| ENSG00000272767 | JMJD1C-AS1 | 1.169004528 | 0.017023759 |
| ENSG00000197238 | HIST1H4J | 1.16983359 | 0.046186512 |
| ENSG00000025156 | HSF2 | 1.170152092 | 8.44E-10 |
| ENSG00000132522 | GPS2 | 1.170345735 | 0.000616144 |
| ENSG00000273352 |  | 1.170896984 | 0.005550994 |
| ENSG00000204160 | ZDHHC18 | 1.17124553 | 3.53E-20 |
| ENSG00000163171 | CDC42EP3 | 1.171337158 | 3.5E-20 |
| ENSG00000152778 | IFIT5 | 1.171803943 | 1.6E-09 |
| ENSG00000131019 | ULBP3 | 1.172627521 | 7.14E-08 |
| ENSG00000198363 | ASPH | 1.172777626 | 3.9E-27 |
| ENSG00000197409 | HIST1H3D | 1.17352896 | 0.041769928 |
| ENSG00000028839 | TBPL1 | 1.174165222 | 3.41E-14 |
| ENSG00000100934 | SEC23A | 1.174837228 | 9.76E-13 |
| ENSG00000131669 | NINJ1 | 1.174840704 | 7.45E-13 |
| ENSG00000163412 | EIF4E3 | 1.174857645 | 0.0000103 |
| ENSG00000197746 | PSAP | 1.175983829 | 3.99E-37 |
| ENSG00000196312 | MFSD14C | 1.178180717 | 1.68E-09 |
| ENSG00000090520 | DNAJB11 | 1.178730613 | 1.95E-19 |
| ENSG00000156966 | B3GNT7 | 1.179214616 | 0.000544178 |
| ENSG00000011426 | ANLN | 1.18000363 | 0.000000831 |
| ENSG00000151715 | TMEM45B | 1.180118407 | 1.55E-22 |
| ENSG00000215126 | CBWD6 | 1.180723292 | 0.000000134 |
| ENSG00000112984 | KIF20A | 1.180903501 | 0.00000129 |
| ENSG00000166848 | TERF2IP | 1.18204112 | 2.32E-23 |
| ENSG00000164916 | FOXK1 | 1.18250512 | 3.89E-25 |
| ENSG00000161010 | MRNIP | 1.18250825 | 2.76E-12 |
| ENSG00000253626 | EIF5AL1 | 1.182580404 | 0.004202962 |
| ENSG00000279541 | AC005261.5 | 1.184541807 | 0.017571654 |
| ENSG00000165416 | SUGT1 | 1.185070152 | 1.58E-25 |
| ENSG00000228218 | ATF4P3 | 1.185091348 | 0.013992753 |
| ENSG00000157020 | SEC13 | 1.185397113 | 0.0000003 |
| ENSG00000106397 | PLOD3 | 1.185663164 | 1.48E-25 |
| ENSG00000006459 | KDM7A | 1.185677376 | 8.85E-14 |
| ENSG00000135018 | UBQLN1 | 1.18572293 | 5.48E-32 |
| ENSG00000110921 | MVK | 1.186963619 | 0.004549933 |
| ENSG00000177426 | TGIF1 | 1.187197717 | 1.93E-18 |
| ENSG00000203697 | CAPN8 | 1.187418244 | 1.72E-08 |
| ENSG00000261526 | AC012615.1 | 1.18904654 | 0.001815841 |
| ENSG00000178764 | ZHX2 | 1.189331445 | 2.47E-22 |
| ENSG00000172432 | GTPBP2 | 1.18999756 | 1.48E-13 |
| ENSG00000181790 | ADGRB1 | 1.190122789 | 0.042994779 |
| ENSG00000105173 | CCNE1 | 1.193684744 | 1.74E-11 |
| ENSG00000135924 | DNAJB2 | 1.193981659 | 6.19E-25 |
| ENSG00000106004 | HOXA5 | 1.194036673 | 1.43E-29 |
| ENSG00000147119 | CHST7 | 1.194784611 | 0.001706086 |
| ENSG00000267523 | AC008735.2 | 1.19653044 | 0.00684306 |
| ENSG00000206503 | HLA-A | 1.197450394 | 1.1E-32 |
| ENSG00000119986 | AVPI1 | 1.198021188 | 1.06E-11 |
| ENSG00000065970 | FOXJ2 | 1.198265132 | 6.66E-18 |
| ENSG00000081019 | RSBN1 | 1.198397814 | 5.39E-13 |
| ENSG00000136868 | SLC31A1 | 1.198554504 | 8.69E-11 |
| ENSG00000006432 | MAP3K9 | 1.198763416 | 2.04E-10 |
| ENSG00000239857 | GET4 | 1.200631203 | 1.91E-14 |
| ENSG00000168374 | ARF4 | 1.201211981 | 2.77E-10 |
| ENSG00000170802 | FOXN2 | 1.202897198 | 1.23E-25 |
| ENSG00000168003 | SLC3A2 | 1.203070427 | 4.95E-09 |
| ENSG00000116604 | MEF2D | 1.203887355 | 2.74E-28 |
| ENSG00000223803 | RPS20P14 | 1.204464146 | 4.01E-09 |
| ENSG00000267681 | AC135721.1 | 1.206005411 | 0.009523679 |
| ENSG00000114416 | FXR1 | 1.206327099 | 4.79E-32 |
| ENSG00000179454 | KLHL28 | 1.206610901 | 2.33E-14 |
| ENSG00000165813 | CCDC186 | 1.208231949 | 1.21E-22 |
| ENSG00000164244 | PRRC1 | 1.208776539 | 1E-12 |
| ENSG00000166562 | SEC11C | 1.208916716 | 8.66E-10 |
| ENSG00000168795 | ZBTB5 | 1.210096399 | 5.93E-17 |
| ENSG00000243477 | NAT6 | 1.211984063 | 0.00000593 |
| ENSG00000203760 | CENPW | 1.212230673 | 1.51E-09 |
| ENSG00000214357 | NEURL1B | 1.212260141 | 0.000267878 |
| ENSG00000138834 | MAPK8IP3 | 1.213267135 | 8.98E-17 |
| ENSG00000264918 |  | 1.214385838 | 0.0056379 |
| ENSG00000230667 | SETSIP | 1.214783654 | 0.030970957 |
| ENSG00000182247 | UBE2E2 | 1.215907259 | 2.58E-14 |
| ENSG00000125826 | RBCK1 | 1.216146107 | 1.8E-20 |
| ENSG00000167680 | SEMA6B | 1.216998125 | 0.015158416 |
| ENSG00000143376 | SNX27 | 1.217039672 | 7.22E-20 |
| ENSG00000105971 | CAV2 | 1.219261303 | 5.33E-17 |
| ENSG00000264522 | OTUD7B | 1.219743711 | 5.39E-29 |
| ENSG00000135744 | AGT | 1.220723197 | 0.002090626 |
| ENSG00000154065 | ANKRD29 | 1.220886972 | 0.00000615 |
| ENSG00000181544 | FANCB | 1.221155593 | 0.00041152 |
| ENSG00000227296 |  | 1.221227591 | 0.012716681 |
| ENSG00000164713 | BRI3 | 1.221646439 | 5.26E-28 |
| ENSG00000185947 | ZNF267 | 1.221801911 | 6.6E-11 |
| ENSG00000069011 | PITX1 | 1.222403194 | 6.62E-14 |
| ENSG00000273572 |  | 1.223063365 | 0.0000794 |
| ENSG00000167468 | GPX4 | 1.223795101 | 3.86E-17 |
| ENSG00000169714 | CNBP | 1.224537146 | 4.24E-26 |
| ENSG00000023330 | ALAS1 | 1.224543918 | 1.2E-30 |
| ENSG00000159023 | EPB41 | 1.224584614 | 1.87E-16 |
| ENSG00000198929 | NOS1AP | 1.225503249 | 4.71E-09 |
| ENSG00000240449 | AC005586.1 | 1.225823953 | 0.000615999 |
| ENSG00000197930 | ERO1A | 1.226782571 | 6.83E-22 |
| ENSG00000129484 | PARP2 | 1.226861898 | 0.0000012 |
| ENSG00000074695 | LMAN1 | 1.227426181 | 1.67E-15 |
| ENSG00000170525 | PFKFB3 | 1.228259039 | 9.74E-31 |
| ENSG00000108474 | PIGL | 1.228846626 | 2.93E-09 |
| ENSG00000150556 | LYPD6B | 1.228983547 | 0.000207378 |
| ENSG00000100401 | RANGAP1 | 1.229707614 | 8.85E-14 |
| ENSG00000243927 | MRPS6 | 1.229867633 | 3.23E-09 |
| ENSG00000140545 | MFGE8 | 1.231555814 | 4.78E-16 |
| ENSG00000203814 | HIST2H2BF | 1.232686758 | 0.048420204 |
| ENSG00000183655 | KLHL25 | 1.233073003 | 1.31E-11 |
| ENSG00000164442 | CITED2 | 1.234607867 | 4.67E-18 |
| ENSG00000158201 | ABHD3 | 1.234761975 | 3.51E-20 |
| ENSG00000117410 | ATP6V0B | 1.235442465 | 1.11E-12 |
| ENSG00000250731 | TPM3P6 | 1.235494283 | 0.043304586 |
| ENSG00000175182 | FAM131A | 1.236045084 | 5.81E-13 |
| ENSG00000147852 | VLDLR | 1.236754868 | 0.0000247 |
| ENSG00000110987 | BCL7A | 1.238371597 | 1.78E-22 |
| ENSG00000104267 | CA2 | 1.238500653 | 4.45E-09 |
| ENSG00000235489 | DBF4P1 | 1.238689152 | 0.01141893 |
| ENSG00000170561 | IRX2 | 1.238908285 | 2.73E-17 |
| ENSG00000173209 | AHSA2P | 1.239354421 | 3.54E-09 |
| ENSG00000233841 |  | 1.239407109 | 0.022186008 |
| ENSG00000238103 | RPL9P7 | 1.239504061 | 0.002218674 |
| ENSG00000123562 | MORF4L2 | 1.239511818 | 3.77E-15 |
| ENSG00000119048 | UBE2B | 1.2407553 | 1.86E-20 |
| ENSG00000090013 | BLVRB | 1.241040228 | 7.69E-15 |
| ENSG00000175198 | PCCA | 1.241299059 | 4.68E-19 |
| ENSG00000214274 | ANG | 1.243177425 | 0.002271272 |
| ENSG00000198517 | MAFK | 1.244111276 | 4.34E-21 |
| ENSG00000168310 | IRF2 | 1.244850838 | 5.96E-13 |
| ENSG00000151135 | TMEM263 | 1.245753346 | 2.38E-18 |
| ENSG00000080822 | CLDND1 | 1.2459497 | 8.17E-16 |
| ENSG00000275400 | AC006001.4 | 1.247148354 | 0.000000421 |
| ENSG00000128595 | CALU | 1.247337222 | 3.24E-13 |
| ENSG00000117013 | KCNQ4 | 1.249014484 | 0.00000106 |
| ENSG00000139354 | GAS2L3 | 1.250970881 | 1.69E-09 |
| ENSG00000068137 | PLEKHH3 | 1.251143441 | 3.04E-11 |
| ENSG00000239719 | AC004890.1 | 1.251989029 | 0.036461987 |
| ENSG00000026652 | AGPAT4 | 1.25242564 | 0.000000108 |
| ENSG00000168917 | SLC35G2 | 1.252498037 | 0.028134551 |
| ENSG00000114120 | SLC25A36 | 1.253110128 | 1.63E-24 |
| ENSG00000259250 | AC018904.1 | 1.254697345 | 0.013079703 |
| ENSG00000273472 | AC096733.2 | 1.254831625 | 0.006441408 |
| ENSG00000196756 | SNHG17 | 1.254874746 | 2.41E-19 |
| ENSG00000220804 | LINC01881 | 1.255049988 | 0.0000489 |
| ENSG00000178074 | C2orf69 | 1.25531408 | 8.11E-16 |
| ENSG00000224032 | EPB41L4A-AS1 | 1.255398693 | 2.64E-28 |
| ENSG00000142669 | SH3BGRL3 | 1.257477318 | 5.24E-24 |
| ENSG00000103642 | LACTB | 1.257515095 | 1.94E-26 |
| ENSG00000112715 | VEGFA | 1.259951535 | 2.08E-14 |
| ENSG00000119640 | ACYP1 | 1.260350942 | 6.82E-12 |
| ENSG00000167508 | MVD | 1.26191679 | 5.4E-09 |
| ENSG00000147050 | KDM6A | 1.262498723 | 1.51E-39 |
| ENSG00000115520 | COQ10B | 1.262590977 | 1.68E-21 |
| ENSG00000163082 | SGPP2 | 1.263254691 | 9.29E-37 |
| ENSG00000141867 | BRD4 | 1.264853543 | 4.48E-16 |
| ENSG00000166579 | NDEL1 | 1.266132923 | 3.67E-17 |
| ENSG00000100941 | PNN | 1.2673049 | 3.26E-21 |
| ENSG00000176473 | WDR25 | 1.269182422 | 1.08E-08 |
| ENSG00000090861 | AARS | 1.269203169 | 9.35E-18 |
| ENSG00000188895 | MSL1 | 1.269231606 | 4.66E-18 |
| ENSG00000106052 | TAX1BP1 | 1.270105573 | 3.78E-43 |
| ENSG00000111816 | FRK | 1.270411499 | 7.5E-17 |
| ENSG00000143882 | ATP6V1C2 | 1.27137705 | 0.0000212 |
| ENSG00000115419 | GLS | 1.271911981 | 1.15E-39 |
| ENSG00000160213 | CSTB | 1.273657742 | 5.26E-27 |
| ENSG00000172893 | DHCR7 | 1.273919489 | 1.03E-08 |
| ENSG00000143429 | AC116050.1 | 1.274205893 | 1.51E-10 |
| ENSG00000259917 | HNRNPLP2 | 1.274293515 | 0.006239811 |
| ENSG00000136840 | ST6GALNAC4 | 1.275150982 | 2.34E-17 |
| ENSG00000213085 | CFAP45 | 1.275397286 | 0.009128019 |
| ENSG00000105993 | DNAJB6 | 1.276306488 | 1.05E-41 |
| ENSG00000239569 | KMT2E-AS1 | 1.277073869 | 0.0000146 |
| ENSG00000111412 | C12orf49 | 1.27777402 | 3.89E-30 |
| ENSG00000269821 | KCNQ1OT1 | 1.278125064 | 0.00000403 |
| ENSG00000120438 | TCP1 | 1.279189766 | 4.3E-18 |
| ENSG00000108861 | DUSP3 | 1.279839919 | 2.63E-46 |
| ENSG00000116350 | SRSF4 | 1.28129975 | 4.77E-16 |
| ENSG00000115306 | SPTBN1 | 1.281486279 | 9.52E-26 |
| ENSG00000235245 | AL360181.2 | 1.282521349 | 0.011357627 |
| ENSG00000131400 | NAPSA | 1.28311941 | 0.028030731 |
| ENSG00000108828 | VAT1 | 1.283551434 | 1.51E-30 |
| ENSG00000181826 | RELL1 | 1.284006767 | 0.00000722 |
| ENSG00000155660 | PDIA4 | 1.284383069 | 2.09E-11 |
| ENSG00000131471 | AOC3 | 1.284532271 | 0.010648012 |
| ENSG00000237991 | RPL35P1 | 1.285801329 | 0.002323714 |
| ENSG00000083937 | CHMP2B | 1.286491831 | 1.24E-33 |
| ENSG00000134470 | IL15RA | 1.289898058 | 8.17E-20 |
| ENSG00000041988 | THAP3 | 1.290497702 | 5.18E-10 |
| ENSG00000182827 | ACBD3 | 1.29133204 | 8.89E-20 |
| ENSG00000106665 | CLIP2 | 1.292415808 | 8.55E-37 |
| ENSG00000005893 | LAMP2 | 1.293269644 | 3.61E-40 |
| ENSG00000105329 | TGFB1 | 1.29377664 | 1.53E-20 |
| ENSG00000105245 | NUMBL | 1.293954383 | 5.97E-22 |
| ENSG00000158062 | UBXN11 | 1.294512749 | 6.48E-16 |
| ENSG00000237022 |  | 1.294568216 | 3.32E-17 |
| ENSG00000099625 | CBARP | 1.294580011 | 1.52E-15 |
| ENSG00000160223 | ICOSLG | 1.294683253 | 0.000000047 |
| ENSG00000272079 | AC004233.3 | 1.295209271 | 0.000000222 |
| ENSG00000278270 |  | 1.295615044 | 0.0000628 |
| ENSG00000198855 | FICD | 1.295850912 | 4.04E-09 |
| ENSG00000143702 | CEP170 | 1.297368865 | 0.005182743 |
| ENSG00000145819 | ARHGAP26 | 1.299849362 | 5.33E-32 |
| ENSG00000165678 | GHITM | 1.300698216 | 6.04E-31 |
| ENSG00000106537 | TSPAN13 | 1.301094113 | 7.19E-16 |
| ENSG00000142549 | IGLON5 | 1.301336541 | 0.001752753 |
| ENSG00000254612 | AP001000.1 | 1.301965953 | 0.000215368 |
| ENSG00000131791 | PRKAB2 | 1.30248313 | 1.59E-12 |
| ENSG00000165997 | ARL5B | 1.302923925 | 9.27E-25 |
| ENSG00000151229 | SLC2A13 | 1.303539481 | 0.000000211 |
| ENSG00000277048 |  | 1.30414019 | 0.043545477 |
| ENSG00000161921 | CXCL16 | 1.304685175 | 3.56E-22 |
| ENSG00000140941 | MAP1LC3B | 1.30498328 | 2.5E-17 |
| ENSG00000165704 | HPRT1 | 1.30541619 | 3.61E-13 |
| ENSG00000113742 | CPEB4 | 1.30716727 | 7.61E-19 |
| ENSG00000143614 | GATAD2B | 1.309052747 | 0.000102559 |
| ENSG00000170889 | RPS9 | 1.30952936 | 0.000497913 |
| ENSG00000127334 | DYRK2 | 1.309692392 | 7.45E-24 |
| ENSG00000127666 | TICAM1 | 1.309844994 | 8.46E-19 |
| ENSG00000138604 | GLCE | 1.309861339 | 1.87E-30 |
| ENSG00000116871 | MAP7D1 | 1.312523342 | 3.74E-32 |
| ENSG00000107341 | UBE2R2 | 1.314162642 | 3.38E-31 |
| ENSG00000163961 | RNF168 | 1.314260594 | 3.49E-19 |
| ENSG00000110958 | PTGES3 | 1.31469151 | 8.9E-13 |
| ENSG00000280206 | AC026401.3 | 1.314815741 | 0.0000638 |
| ENSG00000237238 | BMS1P10 | 1.317197321 | 6.16E-12 |
| ENSG00000163362 | INAVA | 1.318971668 | 1.32E-33 |
| ENSG00000068878 | PSME4 | 1.319193112 | 8.01E-30 |
| ENSG00000234741 | GAS5 | 1.321066265 | 2.93E-22 |
| ENSG00000198729 | PPP1R14C | 1.321336778 | 0.000609885 |
| ENSG00000121060 | TRIM25 | 1.321961524 | 1.04E-29 |
| ENSG00000117461 | PIK3R3 | 1.323240201 | 7.76E-34 |
| ENSG00000106803 | SEC61B | 1.323248973 | 2.08E-20 |
| ENSG00000183421 | RIPK4 | 1.323437963 | 7.57E-27 |
| ENSG00000257315 | ZBED6 | 1.323510783 | 0.000000035 |
| ENSG00000176407 | KCMF1 | 1.324106621 | 6.63E-25 |
| ENSG00000049323 | LTBP1 | 1.327268466 | 0.0000113 |
| ENSG00000133687 | TMTC1 | 1.327364117 | 0.000211604 |
| ENSG00000113240 | CLK4 | 1.328532049 | 1.58E-13 |
| ENSG00000277105 |  | 1.329508493 | 0.010380103 |
| ENSG00000215182 | MUC5AC | 1.331711424 | 0.036291644 |
| ENSG00000254615 | AC027031.2 | 1.332011556 | 0.00000595 |
| ENSG00000181690 | PLAG1 | 1.332963805 | 1.07E-09 |
| ENSG00000121900 | TMEM54 | 1.333258087 | 2.09E-17 |
| ENSG00000266931 | AC092651.2 | 1.333394281 | 0.00000267 |
| ENSG00000153561 | RMND5A | 1.333434563 | 4.24E-32 |
| ENSG00000030110 | BAK1 | 1.33420926 | 3.47E-17 |
| ENSG00000092871 | RFFL | 1.334471387 | 9.85E-31 |
| ENSG00000168785 | TSPAN5 | 1.336492797 | 8.48E-33 |
| ENSG00000225259 | ST13P6 | 1.337133669 | 0.009820607 |
| ENSG00000105520 | PLPPR2 | 1.338087353 | 2.34E-14 |
| ENSG00000169692 | AGPAT2 | 1.339868338 | 1.7E-28 |
| ENSG00000174307 | PHLDA3 | 1.340249672 | 1.69E-13 |
| ENSG00000101255 | TRIB3 | 1.34058154 | 1.78E-09 |
| ENSG00000070190 | DAPP1 | 1.340950122 | 7.67E-24 |
| ENSG00000119326 | CTNNAL1 | 1.34096031 | 2.11E-12 |
| ENSG00000233239 |  | 1.34410802 | 0.007280401 |
| ENSG00000128791 | TWSG1 | 1.344401302 | 3.85E-21 |
| ENSG00000108829 | LRRC59 | 1.345431894 | 2.79E-18 |
| ENSG00000140105 | WARS | 1.345473395 | 3.77E-16 |
| ENSG00000130303 | BST2 | 1.346596233 | 1.14E-21 |
| ENSG00000148842 | CNNM2 | 1.34666262 | 1.17E-22 |
| ENSG00000180747 | SMG1P3 | 1.347570705 | 6.78E-08 |
| ENSG00000109920 | FNBP4 | 1.34867853 | 3.09E-28 |
| ENSG00000248503 | AL356235.1 | 1.348747423 | 0.013429781 |
| ENSG00000125798 | FOXA2 | 1.351135065 | 6.9E-14 |
| ENSG00000130449 | ZSWIM6 | 1.351522711 | 2.64E-27 |
| ENSG00000103089 | FA2H | 1.352366871 | 2.23E-27 |
| ENSG00000280211 | AC106886.4 | 1.352433611 | 0.028218945 |
| ENSG00000276248 | AL442125.1 | 1.353397298 | 0.012177392 |
| ENSG00000197976 | AKAP17A | 1.353716038 | 3.02E-26 |
| ENSG00000134955 | SLC37A2 | 1.354326887 | 0.037057481 |
| ENSG00000104687 | GSR | 1.355011356 | 2.95E-17 |
| ENSG00000269378 | ITGB1P1 | 1.355254497 | 0.001402107 |
| ENSG00000155265 | GOLGA7B | 1.355676526 | 0.000000578 |
| ENSG00000204256 | BRD2 | 1.355968462 | 5.41E-10 |
| ENSG00000172197 | MBOAT1 | 1.356395234 | 0.000000337 |
| ENSG00000103187 | COTL1 | 1.356792064 | 1.08E-14 |
| ENSG00000104447 | TRPS1 | 1.357950423 | 2E-23 |
| ENSG00000135378 | PRRG4 | 1.358378957 | 2.88E-10 |
| ENSG00000196542 | SPTSSB | 1.358934161 | 0.0000195 |
| ENSG00000197061 | HIST1H4C | 1.36029602 | 0.011863381 |
| ENSG00000232559 |  | 1.361375958 | 1.93E-11 |
| ENSG00000115977 | AAK1 | 1.361907155 | 5.8E-22 |
| ENSG00000233016 | SNHG7 | 1.362614902 | 4.32E-31 |
| ENSG00000226650 | KIF4B | 1.362742394 | 0.037235838 |
| ENSG00000196428 | TSC22D2 | 1.363057142 | 4.55E-34 |
| ENSG00000132432 | SEC61G | 1.363362102 | 1.05E-20 |
| ENSG00000221914 | PPP2R2A | 1.364015787 | 1.68E-28 |
| ENSG00000135709 | KIAA0513 | 1.365700145 | 2.12E-11 |
| ENSG00000149428 | HYOU1 | 1.365906542 | 0.000000583 |
| ENSG00000142168 | SOD1 | 1.366371883 | 5.1E-21 |
| ENSG00000164970 | FAM219A | 1.366667499 | 2.66E-12 |
| ENSG00000121274 | PAPD5 | 1.36699615 | 1.11E-28 |
| ENSG00000130584 | ZBTB46 | 1.36699819 | 5.68E-25 |
| ENSG00000135253 | KCP | 1.367186339 | 0.00031275 |
| ENSG00000254682 | AP002387.2 | 1.367800976 | 7.77E-11 |
| ENSG00000187605 | TET3 | 1.367906438 | 2.26E-17 |
| ENSG00000215093 | EEF1A1P29 | 1.369065968 | 0.032503885 |
| ENSG00000167306 | MYO5B | 1.369234586 | 2.56E-35 |
| ENSG00000165434 | PGM2L1 | 1.369576157 | 1.19E-21 |
| ENSG00000119771 | KLHL29 | 1.370642033 | 0.00000172 |
| ENSG00000196923 | PDLIM7 | 1.370765073 | 1.49E-10 |
| ENSG00000008128 | CDK11A | 1.37155206 | 0.00000384 |
| ENSG00000197608 | ZNF841 | 1.371559648 | 2.3E-20 |
| ENSG00000175063 | UBE2C | 1.371694912 | 7.87E-10 |
| ENSG00000117650 | NEK2 | 1.372964329 | 1.03E-10 |
| ENSG00000185262 | UBALD2 | 1.373499809 | 1.29E-10 |
| ENSG00000235587 | GAPDHP65 | 1.37363802 | 0.006159383 |
| ENSG00000282183 |  | 1.375256551 | 0.006404972 |
| ENSG00000258940 | AL132639.2 | 1.377122135 | 0.008422498 |
| ENSG00000230795 | HLA-K | 1.379337898 | 0.019888459 |
| ENSG00000276561 |  | 1.380088896 | 5.06E-10 |
| ENSG00000079332 | SAR1A | 1.380997851 | 5.92E-21 |
| ENSG00000065491 | TBC1D22B | 1.381883531 | 3.7E-28 |
| ENSG00000133789 | SWAP70 | 1.382337986 | 5.81E-42 |
| ENSG00000077616 | NAALAD2 | 1.383325089 | 0.015279961 |
| ENSG00000229252 |  | 1.383745087 | 0.021746128 |
| ENSG00000184840 | TMED9 | 1.384854678 | 1.18E-13 |
| ENSG00000267940 | AC022762.2 | 1.385324956 | 0.001091851 |
| ENSG00000106615 | RHEB | 1.386453202 | 1.4E-18 |
| ENSG00000071054 | MAP4K4 | 1.387062773 | 4.91E-09 |
| ENSG00000153234 | NR4A2 | 1.388256107 | 7.04E-13 |
| ENSG00000096654 | ZNF184 | 1.389162805 | 2.97E-19 |
| ENSG00000137819 | PAQR5 | 1.389228007 | 7.03E-48 |
| ENSG00000064300 | NGFR | 1.390192994 | 0.034637344 |
| ENSG00000189223 | PAX8-AS1 | 1.391356855 | 1.4E-17 |
| ENSG00000105559 | PLEKHA4 | 1.391960593 | 1.21E-08 |
| ENSG00000148154 | UGCG | 1.392993254 | 7.68E-46 |
| ENSG00000233966 | UBE2SP1 | 1.393002244 | 0.004149651 |
| ENSG00000120539 | MASTL | 1.393628852 | 3.14E-09 |
| ENSG00000178177 | LCORL | 1.394629584 | 5.17E-15 |
| ENSG00000095397 | WHRN | 1.394836412 | 0.030864686 |
| ENSG00000140451 | PIF1 | 1.397243051 | 5.39E-08 |
| ENSG00000255769 | GOLGA2P10 | 1.397835302 | 9.83E-08 |
| ENSG00000152766 | ANKRD22 | 1.398438643 | 1.42E-18 |
| ENSG00000267216 | AC020915.1 | 1.399928321 | 0.04706458 |
| ENSG00000182481 | KPNA2 | 1.400004077 | 0.0000241 |
| ENSG00000107249 | GLIS3 | 1.400177842 | 4.67E-13 |
| ENSG00000183741 | CBX6 | 1.400276586 | 1.11E-22 |
| ENSG00000140450 | ARRDC4 | 1.400302033 | 1.56E-10 |
| ENSG00000171241 | SHCBP1 | 1.40146371 | 6.16E-10 |
| ENSG00000123595 | RAB9A | 1.401932612 | 7.11E-20 |
| ENSG00000197696 | NMB | 1.401951201 | 3.63E-16 |
| ENSG00000234704 |  | 1.403442179 | 0.034682867 |
| ENSG00000101084 | C20orf24 | 1.403518834 | 7.66E-13 |
| ENSG00000196550 | FAM72A | 1.406765297 | 4.03E-08 |
| ENSG00000150403 | TMCO3 | 1.407689876 | 9.43E-36 |
| ENSG00000260853 | AC109460.2 | 1.408669283 | 0.041314653 |
| ENSG00000052344 | PRSS8 | 1.409386898 | 9.73E-30 |
| ENSG00000080644 | CHRNA3 | 1.409761292 | 0.022880394 |
| ENSG00000170017 | ALCAM | 1.409799364 | 3.41E-37 |
| ENSG00000240891 | PLCXD2 | 1.410817834 | 0.0000278 |
| ENSG00000147155 | EBP | 1.411323599 | 0.000914413 |
| ENSG00000179134 | SAMD4B | 1.41159078 | 8.42E-63 |
| ENSG00000150907 | FOXO1 | 1.412387149 | 5.41E-22 |
| ENSG00000198496 | NBR2 | 1.413746479 | 3.8E-15 |
| ENSG00000187122 | SLIT1 | 1.413816779 | 0.016204045 |
| ENSG00000168528 | SERINC2 | 1.414869699 | 1.46E-48 |
| ENSG00000174028 | FAM3C2 | 1.415894794 | 1.73E-11 |
| ENSG00000124882 | EREG | 1.416585078 | 1.17E-09 |
| ENSG00000228409 | CCT6P1 | 1.41734912 | 7.33E-10 |
| ENSG00000248641 | HMGA1P2 | 1.418456776 | 0.000931601 |
| ENSG00000244144 | AC128688.1 | 1.419159624 | 0.03677542 |
| ENSG00000153094 | BCL2L11 | 1.419441449 | 8.14E-18 |
| ENSG00000120742 | SERP1 | 1.422563559 | 6.99E-21 |
| ENSG00000225691 |  | 1.422644841 | 1.63E-39 |
| ENSG00000115112 | TFCP2L1 | 1.422802453 | 0.049285365 |
| ENSG00000173812 | EIF1 | 1.423861908 | 3.51E-29 |
| ENSG00000071575 | TRIB2 | 1.424099616 | 0.000532479 |
| ENSG00000105821 | DNAJC2 | 1.424278095 | 1.91E-36 |
| ENSG00000196821 | C6orf106 | 1.425228462 | 1.27E-21 |
| ENSG00000273993 |  | 1.425555032 | 0.020252997 |
| ENSG00000144674 | GOLGA4 | 1.425813875 | 1.05E-25 |
| ENSG00000165861 | ZFYVE1 | 1.42738848 | 3.34E-29 |
| ENSG00000132196 | HSD17B7 | 1.428521265 | 0.000000253 |
| ENSG00000109321 | AREG | 1.429249367 | 7.86E-11 |
| ENSG00000268713 | AC005261.3 | 1.429487178 | 0.0000114 |
| ENSG00000121671 | CRY2 | 1.42984848 | 3.82E-15 |
| ENSG00000178502 | KLHL11 | 1.430982063 | 8.77E-10 |
| ENSG00000162924 | REL | 1.432141937 | 1.31E-10 |
| ENSG00000228299 |  | 1.432268482 | 4.27E-27 |
| ENSG00000118689 | FOXO3 | 1.432901632 | 1.24E-37 |
| ENSG00000248971 | KRT8P46 | 1.433080386 | 0.001291151 |
| ENSG00000196878 | LAMB3 | 1.433433332 | 7.77E-35 |
| ENSG00000196843 | ARID5A | 1.437435062 | 1.22E-09 |
| ENSG00000130956 | HABP4 | 1.43835812 | 5.85E-34 |
| ENSG00000276966 | HIST1H4E | 1.438498401 | 0.029910667 |
| ENSG00000188483 | IER5L | 1.439057298 | 3.13E-17 |
| ENSG00000198742 | SMURF1 | 1.440237771 | 1.02E-27 |
| ENSG00000198576 | ARC | 1.441036407 | 4.04E-10 |
| ENSG00000115548 | KDM3A | 1.441370221 | 4.06E-27 |
| ENSG00000273802 | HIST1H2BG | 1.441468172 | 0.000466102 |
| ENSG00000199753 | SNORD104 | 1.442415504 | 0.001656319 |
| ENSG00000139163 | ETNK1 | 1.442652239 | 4.13E-31 |
| ENSG00000125148 | MT2A | 1.443876599 | 2.4E-13 |
| ENSG00000108960 | MMD | 1.44390409 | 7.97E-36 |
| ENSG00000081087 | OSTM1 | 1.444776294 | 1.86E-19 |
| ENSG00000166949 | SMAD3 | 1.446066743 | 1.53E-39 |
| ENSG00000116754 | SRSF11 | 1.448564209 | 3.58E-50 |
| ENSG00000127124 | HIVEP3 | 1.449862913 | 0.0000891 |
| ENSG00000168439 | STIP1 | 1.449986121 | 1.74E-36 |
| ENSG00000102393 | GLA | 1.450395632 | 3.7E-10 |
| ENSG00000120889 | TNFRSF10B | 1.450687411 | 1.01E-29 |
| ENSG00000187288 | CIDEC | 1.451244072 | 0.000612052 |
| ENSG00000066422 | ZBTB11 | 1.452423091 | 3.76E-45 |
| ENSG00000185222 | TCEAL9 | 1.452674707 | 1.05E-13 |
| ENSG00000107362 | ABHD17B | 1.453635358 | 9.02E-24 |
| ENSG00000219665 | ZNF433-AS1 | 1.454093267 | 5.73E-13 |
| ENSG00000138798 | EGF | 1.454399666 | 0.00000847 |
| ENSG00000086061 | DNAJA1 | 1.4563725 | 1.68E-42 |
| ENSG00000142867 | BCL10 | 1.456403802 | 2.56E-35 |
| ENSG00000140743 | CDR2 | 1.457979011 | 2.01E-18 |
| ENSG00000173276 | ZBTB21 | 1.458323874 | 4.93E-24 |
| ENSG00000170498 | KISS1 | 1.459524836 | 0.001212799 |
| ENSG00000109929 | SC5D | 1.460195243 | 2.49E-11 |
| ENSG00000144802 | NFKBIZ | 1.461118433 | 3.63E-16 |
| ENSG00000170786 | SDR16C5 | 1.463760862 | 4.45E-14 |
| ENSG00000151726 | ACSL1 | 1.464591936 | 2.9E-17 |
| ENSG00000213822 | CEACAM18 | 1.465761155 | 0.0365287 |
| ENSG00000173207 | CKS1B | 1.468031156 | 6.49E-11 |
| ENSG00000226471 | Z93930.2 | 1.468292501 | 0.000204697 |
| ENSG00000259429 | UBE2Q2P2 | 1.469321874 | 0.005294433 |
| ENSG00000179532 | DNHD1 | 1.469916069 | 2.55E-11 |
| ENSG00000231742 | LINC01273 | 1.470050916 | 0.048816468 |
| ENSG00000198774 | RASSF9 | 1.470621064 | 0.0000382 |
| ENSG00000164142 | FAM160A1 | 1.472222216 | 1.76E-11 |
| ENSG00000227669 |  | 1.47350127 | 0.00000206 |
| ENSG00000162434 | JAK1 | 1.473856354 | 2.42E-56 |
| ENSG00000147889 | CDKN2A | 1.474092763 | 4.57E-20 |
| ENSG00000112335 | SNX3 | 1.474196884 | 9.48E-27 |
| ENSG00000259820 | AC083843.3 | 1.47421717 | 0.00000247 |
| ENSG00000143878 | RHOB | 1.475809339 | 3.94E-30 |
| ENSG00000069956 | MAPK6 | 1.47603986 | 4.39E-44 |
| ENSG00000103460 | TOX3 | 1.476272313 | 7.85E-14 |
| ENSG00000230551 | AC021078.1 | 1.47692323 | 1.19E-15 |
| ENSG00000084734 | GCKR | 1.477099079 | 0.000292393 |
| ENSG00000137145 | DENND4C | 1.478843241 | 4.84E-39 |
| ENSG00000132002 | DNAJB1 | 1.479742845 | 9.91E-34 |
| ENSG00000235657 |  | 1.480037655 | 2.76E-09 |
| ENSG00000236699 | ARHGEF38 | 1.482388494 | 1.97E-15 |
| ENSG00000172927 | MYEOV | 1.482642963 | 4.72E-41 |
| ENSG00000103489 | XYLT1 | 1.482830915 | 0.013582109 |
| ENSG00000206452 |  | 1.485113528 | 0.000323822 |
| ENSG00000272034 | SNORD14A | 1.485478057 | 0.036389189 |
| ENSG00000170340 | B3GNT2 | 1.486857451 | 7.59E-44 |
| ENSG00000231170 | AC002451.1 | 1.488746079 | 0.03375557 |
| ENSG00000258445 | AL132777.1 | 1.489136967 | 0.04269531 |
| ENSG00000164611 | PTTG1 | 1.489623759 | 6.29E-13 |
| ENSG00000268942 | CKS1BP3 | 1.490924048 | 0.003913348 |
| ENSG00000101460 | MAP1LC3A | 1.493632883 | 1.16E-18 |
| ENSG00000006831 | ADIPOR2 | 1.494460811 | 1.28E-27 |
| ENSG00000128564 | VGF | 1.494760525 | 1.65E-09 |
| ENSG00000230069 | LRRC37A15P | 1.496171261 | 0.005056675 |
| ENSG00000231701 | BMS1P13 | 1.50064999 | 0.003948661 |
| ENSG00000273113 | AC133528.1 | 1.50070849 | 0.002136212 |
| ENSG00000115289 | PCGF1 | 1.501158707 | 8.8E-31 |
| ENSG00000232527 | AC245595.1 | 1.501681953 | 0.0000173 |
| ENSG00000104825 | NFKBIB | 1.502045025 | 1.56E-40 |
| ENSG00000112658 | SRF | 1.503214663 | 8.99E-36 |
| ENSG00000182704 | TSKU | 1.503434413 | 1.91E-16 |
| ENSG00000280385 | AP000648.3 | 1.505459818 | 1.34E-10 |
| ENSG00000104312 | RIPK2 | 1.505712586 | 5.44E-40 |
| ENSG00000141232 | TOB1 | 1.508919823 | 2.68E-46 |
| ENSG00000154803 | FLCN | 1.512343692 | 1.02E-23 |
| ENSG00000025772 | TOMM34 | 1.512474328 | 1.65E-40 |
| ENSG00000176678 | FOXL1 | 1.512563217 | 0.000309989 |
| ENSG00000071537 | SEL1L | 1.512770821 | 3.02E-19 |
| ENSG00000278311 | GGNBP2 | 1.513292473 | 0.032742929 |
| ENSG00000132510 | KDM6B | 1.515072491 | 2.73E-16 |
| ENSG00000144366 | GULP1 | 1.515197758 | 3.41E-28 |
| ENSG00000155367 | PPM1J | 1.517338767 | 0.00000469 |
| ENSG00000108176 | DNAJC12 | 1.518451805 | 1.53E-10 |
| ENSG00000114784 | EIF1B | 1.52211338 | 5.59E-31 |
| ENSG00000155366 | RHOC | 1.52307359 | 7.53E-36 |
| ENSG00000113552 | GNPDA1 | 1.524005171 | 5.68E-26 |
| ENSG00000166710 | B2M | 1.524197632 | 1.42E-34 |
| ENSG00000258102 | MAP1LC3B2 | 1.524275715 | 0.0000557 |
| ENSG00000177125 | ZBTB34 | 1.525076985 | 1.31E-22 |
| ENSG00000263069 | AC124319.2 | 1.525532693 | 0.027947415 |
| ENSG00000254595 | AC084337.1 | 1.526594542 | 0.019630845 |
| ENSG00000202198 | RF00100 | 1.527353728 | 0.016403396 |
| ENSG00000232442 | MHENCR | 1.527425615 | 5.16E-12 |
| ENSG00000165121 | AL353743.1 | 1.527534637 | 0.00000348 |
| ENSG00000164266 | SPINK1 | 1.527655212 | 1.25E-32 |
| ENSG00000175691 | ZNF77 | 1.528907681 | 1.08E-10 |
| ENSG00000270761 | AL355353.1 | 1.531798235 | 0.01897027 |
| ENSG00000145375 | SPATA5 | 1.531994927 | 9.05E-13 |
| ENSG00000168556 | ING2 | 1.53367536 | 1.16E-14 |
| ENSG00000043591 | ADRB1 | 1.535123362 | 0.0000111 |
| ENSG00000160211 | G6PD | 1.536658029 | 8.96E-47 |
| ENSG00000140332 | TLE3 | 1.536888209 | 2.51E-48 |
| ENSG00000167779 | IGFBP6 | 1.537231563 | 8.64E-17 |
| ENSG00000100139 | MICALL1 | 1.538258652 | 9.72E-53 |
| ENSG00000124743 | KLHL31 | 1.538434702 | 0.011113589 |
| ENSG00000137673 | MMP7 | 1.538547692 | 8.17E-16 |
| ENSG00000134186 | PRPF38B | 1.53856991 | 4.77E-46 |
| ENSG00000221963 | APOL6 | 1.540625474 | 6.67E-24 |
| ENSG00000234130 | AL359263.1 | 1.542532147 | 0.03204497 |
| ENSG00000168743 | NPNT | 1.542735315 | 2.21E-14 |
| ENSG00000267519 | AC020916.1 | 1.542933481 | 0.000000572 |
| ENSG00000115963 | RND3 | 1.543915236 | 4.5E-58 |
| ENSG00000169635 | HIC2 | 1.545005871 | 5.15E-21 |
| ENSG00000229959 |  | 1.545060152 | 0.00000992 |
| ENSG00000095209 | TMEM38B | 1.545387066 | 1.28E-14 |
| ENSG00000266402 | SNHG25 | 1.545509546 | 0.00000248 |
| ENSG00000185883 | ATP6V0C | 1.54555526 | 0.0153714 |
| ENSG00000174738 | NR1D2 | 1.545606015 | 1.05E-38 |
| ENSG00000152684 | PELO | 1.545688303 | 4.55E-31 |
| ENSG00000114270 | COL7A1 | 1.546448056 | 0.014788689 |
| ENSG00000123240 | OPTN | 1.546777224 | 1.56E-32 |
| ENSG00000119953 | SMNDC1 | 1.547030398 | 4.08E-29 |
| ENSG00000179674 | ARL14 | 1.549497674 | 0.000000108 |
| ENSG00000100100 | PIK3IP1 | 1.552135743 | 1.08E-09 |
| ENSG00000231607 | DLEU2 | 1.553454448 | 9.99E-10 |
| ENSG00000115641 | FHL2 | 1.55427758 | 1.3E-29 |
| ENSG00000180263 | FGD6 | 1.555259788 | 8.88E-40 |
| ENSG00000222041 | CYTOR | 1.556440919 | 9.23E-17 |
| ENSG00000277443 | MARCKS | 1.557070983 | 7.43E-28 |
| ENSG00000253948 | AC104986.2 | 1.557218432 | 0.0000452 |
| ENSG00000235499 | AC073046.1 | 1.557663365 | 0.000000031 |
| ENSG00000130695 | CEP85 | 1.557940132 | 1.08E-15 |
| ENSG00000042286 | AIFM2 | 1.557965227 | 9.08E-44 |
| ENSG00000280527 |  | 1.559475877 | 0.022732187 |
| ENSG00000265972 | TXNIP | 1.559926691 | 1.22E-19 |
| ENSG00000092148 | HECTD1 | 1.56011851 | 2.71E-24 |
| ENSG00000156232 | WHAMM | 1.562816239 | 2.37E-32 |
| ENSG00000005486 | RHBDD2 | 1.564926699 | 8.98E-25 |
| ENSG00000111711 | GOLT1B | 1.565355058 | 5E-14 |
| ENSG00000163659 | TIPARP | 1.56846706 | 5.35E-29 |
| ENSG00000270069 | MIR222HG | 1.570190259 | 0.001192024 |
| ENSG00000125845 | BMP2 | 1.572314608 | 8.71E-15 |
| ENSG00000137309 | HMGA1 | 1.572514409 | 1.71E-45 |
| ENSG00000160685 | ZBTB7B | 1.573488695 | 5.44E-39 |
| ENSG00000175106 | TVP23C | 1.573566599 | 1.32E-09 |
| ENSG00000213700 | RPL17P50 | 1.574106884 | 0.013756512 |
| ENSG00000114737 | CISH | 1.575507047 | 0.000787207 |
| ENSG00000144655 | CSRNP1 | 1.578041778 | 1.8E-19 |
| ENSG00000186395 | KRT10 | 1.578217256 | 2.63E-16 |
| ENSG00000065665 | SEC61A2 | 1.579521232 | 3.06E-12 |
| ENSG00000074842 | MYDGF | 1.580294212 | 1.36E-20 |
| ENSG00000141504 | SAT2 | 1.581101731 | 8.36E-16 |
| ENSG00000179218 | CALR | 1.58129422 | 0.00000287 |
| ENSG00000236830 | CBR3-AS1 | 1.583502127 | 0.008131665 |
| ENSG00000143322 | ABL2 | 1.583662641 | 4.96E-24 |
| ENSG00000171928 | TVP23B | 1.584736517 | 1.85E-32 |
| ENSG00000108179 | PPIF | 1.587618282 | 6.48E-16 |
| ENSG00000101384 | JAG1 | 1.587768863 | 2.69E-15 |
| ENSG00000088826 | SMOX | 1.587771694 | 2.95E-21 |
| ENSG00000101888 | NXT2 | 1.589935803 | 1.25E-25 |
| ENSG00000242247 | ARFGAP3 | 1.590357898 | 3.78E-28 |
| ENSG00000153291 | SLC25A27 | 1.590418435 | 2.6E-10 |
| ENSG00000246451 | AL049840.1 | 1.591017969 | 0.003361024 |
| ENSG00000179833 | SERTAD2 | 1.591348816 | 9.11E-38 |
| ENSG00000131370 | SH3BP5 | 1.592655995 | 0.003213873 |
| ENSG00000257831 | AL136418.1 | 1.593204041 | 0.0000503 |
| ENSG00000163584 | RPL22L1 | 1.595639805 | 3.53E-34 |
| ENSG00000170684 | ZNF296 | 1.598594691 | 4.74E-14 |
| ENSG00000234231 | AC093616.1 | 1.598712547 | 1.17E-08 |
| ENSG00000139537 | CCDC65 | 1.600312402 | 0.002330358 |
| ENSG00000234964 | FABP5P7 | 1.600830644 | 0.025127205 |
| ENSG00000261609 | GAN | 1.603386098 | 8.88E-15 |
| ENSG00000257605 | AC073611.1 | 1.603577269 | 0.019928771 |
| ENSG00000280099 | AL603750.1 | 1.605098083 | 0.004302187 |
| ENSG00000145685 | LHFPL2 | 1.605437552 | 2.51E-48 |
| ENSG00000237218 |  | 1.605563704 | 0.016149956 |
| ENSG00000102359 | SRPX2 | 1.606613549 | 0.0000517 |
| ENSG00000175730 | BAK1P1 | 1.60675389 | 0.047683788 |
| ENSG00000147010 | SH3KBP1 | 1.60739955 | 5.18E-46 |
| ENSG00000163931 | TKT | 1.607493218 | 3.36E-30 |
| ENSG00000268621 | IGFL2-AS1 | 1.608757561 | 0.00000143 |
| ENSG00000245532 | NEAT1 | 1.609010198 | 6.18E-20 |
| ENSG00000186577 | SMIM29 | 1.609143221 | 2.08E-18 |
| ENSG00000134326 | CMPK2 | 1.609603233 | 0.022779911 |
| ENSG00000250764 | AC025178.1 | 1.611459005 | 0.021843045 |
| ENSG00000135241 | PNPLA8 | 1.611814964 | 2.21E-54 |
| ENSG00000143333 | RGS16 | 1.612268606 | 5.39E-14 |
| ENSG00000137575 | SDCBP | 1.613598703 | 6.46E-57 |
| ENSG00000272106 | AL691432.2 | 1.613598724 | 8.74E-12 |
| ENSG00000135862 | LAMC1 | 1.614812412 | 2.22E-49 |
| ENSG00000112186 | CAP2 | 1.615061746 | 4.3E-09 |
| ENSG00000185950 | IRS2 | 1.61519647 | 1.24E-21 |
| ENSG00000223980 |  | 1.615378883 | 4.32E-09 |
| ENSG00000271933 | AL603756.1 | 1.615982812 | 0.002242594 |
| ENSG00000060558 | GNA15 | 1.616115607 | 4.05E-32 |
| ENSG00000066697 | MSANTD3 | 1.61637884 | 1.63E-20 |
| ENSG00000164920 | OSR2 | 1.617017163 | 0.0000206 |
| ENSG00000163660 | CCNL1 | 1.617756472 | 4.33E-28 |
| ENSG00000197461 | PDGFA | 1.618484309 | 1.09E-20 |
| ENSG00000154447 | SH3RF1 | 1.618797973 | 4.81E-37 |
| ENSG00000182158 | CREB3L2 | 1.620197857 | 7.09E-29 |
| ENSG00000226651 |  | 1.620390286 | 0.009136897 |
| ENSG00000169914 | OTUD3 | 1.620660237 | 7.28E-22 |
| ENSG00000138448 | ITGAV | 1.62079264 | 6.99E-48 |
| ENSG00000143061 | IGSF3 | 1.620944969 | 4.09E-41 |
| ENSG00000251138 | AC090502.1 | 1.625280255 | 0.042624335 |
| ENSG00000278948 | AL031587.5 | 1.626709294 | 0.000000388 |
| ENSG00000108106 | UBE2S | 1.628578747 | 2.79E-14 |
| ENSG00000282514 |  | 1.629215832 | 1.6E-10 |
| ENSG00000188206 |  | 1.634987601 | 0.0000352 |
| ENSG00000163993 | S100P | 1.635642365 | 4.14E-30 |
| ENSG00000175550 | DRAP1 | 1.63756466 | 8.99E-28 |
| ENSG00000274849 | AC023043.4 | 1.638920838 | 0.032236848 |
| ENSG00000131069 | ACSS2 | 1.640673416 | 2.6E-11 |
| ENSG00000100226 | GTPBP1 | 1.64170703 | 7.86E-43 |
| ENSG00000177855 | CACYBPP2 | 1.644703288 | 0.0000306 |
| ENSG00000112406 | HECA | 1.644888265 | 1.56E-28 |
| ENSG00000273759 | AL117379.1 | 1.645370835 | 0.028736782 |
| ENSG00000232708 |  | 1.646449422 | 0.0000242 |
| ENSG00000100036 | SLC35E4 | 1.64700267 | 7.36E-18 |
| ENSG00000120063 | GNA13 | 1.65073701 | 2.63E-52 |
| ENSG00000172965 | MIR4435-2HG | 1.651602947 | 8.67E-15 |
| ENSG00000148841 | ITPRIP | 1.653541466 | 8.89E-29 |
| ENSG00000279031 | AC004232.3 | 1.654178841 | 0.016293663 |
| ENSG00000079459 | FDFT1 | 1.654317691 | 1.01E-12 |
| ENSG00000116161 | CACYBP | 1.654352222 | 1.35E-23 |
| ENSG00000255198 | SNHG9 | 1.655734578 | 0.0000183 |
| ENSG00000113916 | BCL6 | 1.655827941 | 6.8E-24 |
| ENSG00000205426 | KRT81 | 1.657123035 | 0.028307536 |
| ENSG00000064932 | SBNO2 | 1.657600737 | 5.1E-09 |
| ENSG00000177542 | SLC25A22 | 1.658002928 | 8.27E-53 |
| ENSG00000104808 | DHDH | 1.660130057 | 0.000976668 |
| ENSG00000224126 | UBE2SP2 | 1.661402504 | 0.0000614 |
| ENSG00000120149 | MSX2 | 1.661651293 | 4.08E-12 |
| ENSG00000155252 | PI4K2A | 1.663765201 | 9.72E-26 |
| ENSG00000163644 | PPM1K | 1.666255599 | 6.13E-23 |
| ENSG00000132471 | WBP2 | 1.666811739 | 4.13E-46 |
| ENSG00000108551 | RASD1 | 1.667856164 | 3.66E-08 |
| ENSG00000165935 | SMCO2 | 1.669251166 | 0.000770278 |
| ENSG00000136810 | TXN | 1.669938361 | 6.16E-22 |
| ENSG00000156875 | MFSD14A | 1.670637581 | 4.93E-36 |
| ENSG00000184897 | H1FX | 1.67141354 | 1.09E-27 |
| ENSG00000172137 | CALB2 | 1.671704499 | 4.54E-16 |
| ENSG00000275903 |  | 1.673404467 | 0.024285886 |
| ENSG00000172216 | CEBPB | 1.677893639 | 2.65E-23 |
| ENSG00000158615 | PPP1R15B | 1.678286109 | 2.2E-33 |
| ENSG00000242125 | SNHG3 | 1.680742007 | 2.14E-25 |
| ENSG00000152527 | PLEKHH2 | 1.680936379 | 3.89E-31 |
| ENSG00000068323 | TFE3 | 1.682416372 | 7.94E-59 |
| ENSG00000123091 | RNF11 | 1.683990338 | 5.58E-64 |
| ENSG00000102174 | PHEX | 1.684353304 | 0.010847997 |
| ENSG00000100097 | LGALS1 | 1.685968188 | 9.08E-20 |
| ENSG00000219470 | AL355802.1 | 1.686627277 | 0.027149373 |
| ENSG00000081320 | STK17B | 1.68726211 | 2.05E-26 |
| ENSG00000173210 | ABLIM3 | 1.688209866 | 1.07E-65 |
| ENSG00000099992 | TBC1D10A | 1.688371609 | 1.05E-19 |
| ENSG00000137261 | KIAA0319 | 1.689375021 | 0.048737138 |
| ENSG00000155330 | C16orf87 | 1.689652768 | 7.48E-28 |
| ENSG00000170899 | GSTA4 | 1.691408094 | 1.83E-27 |
| ENSG00000141458 | NPC1 | 1.691653619 | 5.16E-27 |
| ENSG00000116954 | RRAGC | 1.692248647 | 5.48E-26 |
| ENSG00000181026 | AEN | 1.693976733 | 2.33E-39 |
| ENSG00000043355 | ZIC2 | 1.695308294 | 0.001890328 |
| ENSG00000005001 | PRSS22 | 1.698721128 | 2.91E-26 |
| ENSG00000172361 | CFAP53 | 1.700590961 | 0.001786591 |
| ENSG00000274070 | CASTOR2 | 1.700670941 | 5.5E-09 |
| ENSG00000164430 | CGAS | 1.701340136 | 5.09E-32 |
| ENSG00000157224 | CLDN12 | 1.701369733 | 1.29E-53 |
| ENSG00000187066 | TMEM262 | 1.701686359 | 0.00964239 |
| ENSG00000167280 | ENGASE | 1.701785718 | 2.76E-21 |
| ENSG00000101353 | MROH8 | 1.702037385 | 0.005929479 |
| ENSG00000232931 | LINC00342 | 1.702959194 | 0.000338282 |
| ENSG00000166478 | ZNF143 | 1.703970067 | 4.52E-34 |
| ENSG00000165886 | UBTD1 | 1.704932421 | 7.13E-30 |
| ENSG00000166012 | TAF1D | 1.708681396 | 1.39E-49 |
| ENSG00000143369 | ECM1 | 1.711160521 | 1.61E-11 |
| ENSG00000106948 | AKNA | 1.711705644 | 5.73E-27 |
| ENSG00000164111 | ANXA5 | 1.711963557 | 4.62E-40 |
| ENSG00000100591 | AHSA1 | 1.712214976 | 1.23E-26 |
| ENSG00000274602 | PI4KAP1 | 1.712462253 | 3.56E-08 |
| ENSG00000167766 | ZNF83 | 1.714366827 | 6.57E-22 |
| ENSG00000248780 | ARL4AP2 | 1.71707997 | 0.030420029 |
| ENSG00000267898 | AC026803.2 | 1.717632852 | 0.005535296 |
| ENSG00000070540 | WIPI1 | 1.717966496 | 2.77E-15 |
| ENSG00000253972 | MAL2-AS1 | 1.719118994 | 0.014273764 |
| ENSG00000163874 | ZC3H12A | 1.719695271 | 2.58E-22 |
| ENSG00000166819 | PLIN1 | 1.719791846 | 0.00000581 |
| ENSG00000277476 | AC005332.6 | 1.72213509 | 0.00000235 |
| ENSG00000118496 | FBXO30 | 1.723261028 | 2E-35 |
| ENSG00000197860 | SGTB | 1.723525466 | 1.34E-32 |
| ENSG00000168005 | SPINDOC | 1.727146932 | 7.84E-23 |
| ENSG00000152795 | HNRNPDL | 1.727419005 | 9.04E-40 |
| ENSG00000117399 | CDC20 | 1.729578169 | 1.2E-19 |
| ENSG00000229036 | VDAC1P8 | 1.734786635 | 0.000000707 |
| ENSG00000175793 | SFN | 1.738717297 | 1.49E-18 |
| ENSG00000197063 | MAFG | 1.73876689 | 2.25E-73 |
| ENSG00000143479 | DYRK3 | 1.740948787 | 0.0000426 |
| ENSG00000153487 | ING1 | 1.741485432 | 3.96E-41 |
| ENSG00000189334 | S100A14 | 1.742734944 | 2.04E-47 |
| ENSG00000137955 | RABGGTB | 1.743361985 | 2.73E-56 |
| ENSG00000078399 | HOXA9 | 1.745614151 | 8.93E-30 |
| ENSG00000153933 | DGKE | 1.746451243 | 7.53E-30 |
| ENSG00000088899 | LZTS3 | 1.746455658 | 1.04E-27 |
| ENSG00000153721 | CNKSR3 | 1.74769992 | 2.22E-15 |
| ENSG00000059728 | MXD1 | 1.747901712 | 1.41E-39 |
| ENSG00000182196 | ARL6IP4 | 1.748353301 | 0.017981958 |
| ENSG00000188610 | FAM72B | 1.749138328 | 0.000000115 |
| ENSG00000166598 | HSP90B1 | 1.750480631 | 5.19E-30 |
| ENSG00000136689 | IL1RN | 1.752146318 | 1.08E-19 |
| ENSG00000135451 | TROAP | 1.752313897 | 1.05E-19 |
| ENSG00000164211 | STARD4 | 1.752682044 | 0.000000771 |
| ENSG00000181218 | HIST3H2A | 1.753131477 | 8.17E-45 |
| ENSG00000237749 | AL034379.1 | 1.75319767 | 0.020826056 |
| ENSG00000205763 | RP9P | 1.7540891 | 3.15E-14 |
| ENSG00000170542 | SERPINB9 | 1.759478781 | 2.27E-25 |
| ENSG00000111331 | OAS3 | 1.761159588 | 2.6E-16 |
| ENSG00000143256 | PFDN2 | 1.761438924 | 3.78E-46 |
| ENSG00000102804 | TSC22D1 | 1.763131684 | 2.24E-86 |
| ENSG00000047634 | SCML1 | 1.764037892 | 3.17E-15 |
| ENSG00000132823 | OSER1 | 1.764054143 | 1.45E-23 |
| ENSG00000087842 | PIR | 1.765722098 | 3.47E-17 |
| ENSG00000138207 | RBP4 | 1.768143864 | 0.000000289 |
| ENSG00000183199 | HSP90AB3P | 1.76819524 | 0.000000915 |
| ENSG00000113615 | SEC24A | 1.769081841 | 2.14E-30 |
| ENSG00000110721 | CHKA | 1.773669762 | 2.3E-25 |
| ENSG00000197933 | ZNF823 | 1.774719032 | 6.79E-20 |
| ENSG00000083799 | CYLD | 1.776434015 | 2.34E-47 |
| ENSG00000167106 | FAM102A | 1.776519744 | 7.39E-53 |
| ENSG00000164181 | ELOVL7 | 1.777541249 | 2.37E-35 |
| ENSG00000165125 | TRPV6 | 1.777618548 | 0.000077 |
| ENSG00000054967 | RELT | 1.779362171 | 4.79E-15 |
| ENSG00000144136 | SLC20A1 | 1.785341586 | 3.04E-55 |
| ENSG00000231663 | AL355472.1 | 1.789203755 | 0.001546211 |
| ENSG00000262188 | LINC01978 | 1.791452469 | 1.65E-09 |
| ENSG00000186866 | POFUT2 | 1.792298884 | 3.19E-27 |
| ENSG00000215784 | FAM72D | 1.793626061 | 1.42E-08 |
| ENSG00000117519 | CNN3 | 1.795997099 | 5.57E-16 |
| ENSG00000155090 | KLF10 | 1.799694974 | 5.28E-15 |
| ENSG00000205639 | MFSD2B | 1.800309383 | 1.48E-15 |
| ENSG00000279641 | AC120057.3 | 1.801173444 | 0.020267811 |
| ENSG00000226396 | AL031727.1 | 1.802591691 | 0.000556129 |
| ENSG00000226964 | RHEBP2 | 1.806407705 | 5.32E-09 |
| ENSG00000171223 | JUNB | 1.807069019 | 7.41E-20 |
| ENSG00000213390 | ARHGAP19 | 1.80914431 | 3.83E-14 |
| ENSG00000225400 | RAB28P5 | 1.812318045 | 0.010638999 |
| ENSG00000107611 | CUBN | 1.814255858 | 0.031447726 |
| ENSG00000164136 | IL15 | 1.814639907 | 9.59E-14 |
| ENSG00000166669 | ATF7IP2 | 1.818064595 | 0.0000113 |
| ENSG00000140961 | OSGIN1 | 1.818152868 | 2.78E-24 |
| ENSG00000180878 | C11orf42 | 1.818245361 | 0.002896232 |
| ENSG00000197536 | C5orf56 | 1.818942377 | 9.76E-16 |
| ENSG00000279331 | RBM12B-AS1 | 1.820021595 | 0.025703726 |
| ENSG00000010438 | PRSS3 | 1.82049065 | 3.23E-27 |
| ENSG00000117036 | ETV3 | 1.82277418 | 4.23E-29 |
| ENSG00000166311 | SMPD1 | 1.823365994 | 2.19E-23 |
| ENSG00000096384 | HSP90AB1 | 1.82433384 | 5.87E-37 |
| ENSG00000072682 | P4HA2 | 1.825114405 | 3.72E-39 |
| ENSG00000212232 | SNORD17 | 1.826370041 | 0.001997133 |
| ENSG00000177410 | ZFAS1 | 1.826560412 | 2.88E-69 |
| ENSG00000179598 | PLD6 | 1.826798816 | 1.17E-13 |
| ENSG00000168209 | DDIT4 | 1.828725523 | 4.48E-16 |
| ENSG00000134954 | ETS1 | 1.829385486 | 2.59E-37 |
| ENSG00000051108 | HERPUD1 | 1.829654514 | 8.31E-23 |
| ENSG00000259305 | ZHX1-C8orf76 | 1.829733507 | 0.003205945 |
| ENSG00000167797 | CDK2AP2 | 1.829916937 | 1.33E-28 |
| ENSG00000158747 | NBL1 | 1.830293952 | 3.44E-21 |
| ENSG00000105516 | DBP | 1.831550941 | 9.94E-22 |
| ENSG00000234745 | HLA-B | 1.832938347 | 3.28E-10 |
| ENSG00000109107 | ALDOC | 1.841732205 | 0.00000436 |
| ENSG00000219200 | RNASEK | 1.841755329 | 9.15E-08 |
| ENSG00000162734 | PEA15 | 1.842977715 | 3.06E-22 |
| ENSG00000117595 | IRF6 | 1.84303759 | 2.67E-48 |
| ENSG00000005073 | HOXA11 | 1.843820794 | 1.52E-36 |
| ENSG00000163545 | NUAK2 | 1.843922339 | 3.76E-23 |
| ENSG00000081181 | ARG2 | 1.844526667 | 1.17E-10 |
| ENSG00000224745 | AC063965.1 | 1.847723848 | 0.047251897 |
| ENSG00000165389 | SPTSSA | 1.852230638 | 2.44E-55 |
| ENSG00000153208 | MERTK | 1.854835337 | 2.73E-22 |
| ENSG00000198832 | SELENOM | 1.85618698 | 0.000000387 |
| ENSG00000173166 | RAPH1 | 1.856591159 | 1.92E-25 |
| ENSG00000126709 | IFI6 | 1.857457489 | 6.68E-11 |
| ENSG00000157601 | MX1 | 1.859212107 | 0.000173456 |
| ENSG00000015479 | MATR3 | 1.859231959 | 0.000937071 |
| ENSG00000261596 | AC005632.2 | 1.860368501 | 0.040531881 |
| ENSG00000059145 | UNKL | 1.86214023 | 1.08E-20 |
| ENSG00000104081 | BMF | 1.862996348 | 3.61E-36 |
| ENSG00000171621 | SPSB1 | 1.863088579 | 2.34E-41 |
| ENSG00000181722 | ZBTB20 | 1.863218932 | 0.000000102 |
| ENSG00000113161 | HMGCR | 1.864528459 | 5.51E-17 |
| ENSG00000247373 | AC055713.1 | 1.869565313 | 0.0000583 |
| ENSG00000278238 | AL359513.1 | 1.874664022 | 0.000145418 |
| ENSG00000189060 | H1F0 | 1.876471619 | 2.49E-36 |
| ENSG00000279191 | AC068491.4 | 1.876584765 | 0.006916076 |
| ENSG00000158786 | PLA2G2F | 1.878048882 | 0.000000201 |
| ENSG00000214226 | C17orf67 | 1.878416118 | 6.83E-16 |
| ENSG00000141582 | CBX4 | 1.879781219 | 1.24E-29 |
| ENSG00000233589 | AL138789.1 | 1.882434711 | 0.039556914 |
| ENSG00000258645 | HSPE1P2 | 1.882443299 | 0.034092914 |
| ENSG00000198805 | PNP | 1.883049781 | 3.82E-28 |
| ENSG00000100342 | APOL1 | 1.883489591 | 7.92E-44 |
| ENSG00000113811 | SELENOK | 1.884030459 | 5.33E-28 |
| ENSG00000274538 |  | 1.884559717 | 0.00000642 |
| ENSG00000169174 | PCSK9 | 1.886631814 | 3.59E-09 |
| ENSG00000137267 | TUBB2A | 1.886988701 | 1.69E-19 |
| ENSG00000035664 | DAPK2 | 1.890510876 | 1.71E-09 |
| ENSG00000164983 | TMEM65 | 1.892646271 | 3.35E-78 |
| ENSG00000229692 | SOS1-IT1 | 1.893100979 | 0.000091 |
| ENSG00000028137 | TNFRSF1B | 1.893274767 | 0.001162159 |
| ENSG00000158887 | MPZ | 1.893897084 | 0.00000256 |
| ENSG00000142920 | AZIN2 | 1.895836038 | 0.000323538 |
| ENSG00000142178 | SIK1 | 1.896632844 | 6.58E-10 |
| ENSG00000169155 | ZBTB43 | 1.897136767 | 3.13E-38 |
| ENSG00000068028 | RASSF1 | 1.899310743 | 5.12E-29 |
| ENSG00000150961 | SEC24D | 1.899945799 | 3.09E-32 |
| ENSG00000280287 | AC131212.3 | 1.902196237 | 0.00000219 |
| ENSG00000115295 | CLIP4 | 1.903961274 | 4.73E-16 |
| ENSG00000105717 | PBX4 | 1.904632167 | 1.4E-11 |
| ENSG00000174945 | AMZ1 | 1.905600384 | 0.046085143 |
| ENSG00000262251 | AC087388.1 | 1.906635634 | 1.08E-09 |
| ENSG00000102760 | RGCC | 1.907804048 | 3.06E-36 |
| ENSG00000009950 | MLXIPL | 1.907923593 | 0.00000309 |
| ENSG00000165782 | PIP4P1 | 1.909022866 | 6.89E-26 |
| ENSG00000105835 | NAMPT | 1.909705541 | 4.04E-39 |
| ENSG00000274735 |  | 1.909755202 | 0.033341544 |
| ENSG00000005238 | FAM214B | 1.910450907 | 2.43E-63 |
| ENSG00000215018 | COL28A1 | 1.918923858 | 0.036006991 |
| ENSG00000135631 | RAB11FIP5 | 1.919173858 | 1.29E-30 |
| ENSG00000177606 | JUN | 1.920086383 | 2.92E-20 |
| ENSG00000084764 | MAPRE3 | 1.92258982 | 2.65E-39 |
| ENSG00000143217 | NECTIN4 | 1.922868566 | 8.66E-37 |
| ENSG00000230678 |  | 1.922960446 | 0.006240873 |
| ENSG00000001630 | CYP51A1 | 1.924538266 | 8.9E-14 |
| ENSG00000198814 | GK | 1.92456817 | 5.75E-13 |
| ENSG00000257681 | AC025265.1 | 1.928426041 | 0.022016288 |
| ENSG00000249193 | HSPD1P5 | 1.928922376 | 0.021254823 |
| ENSG00000099875 | MKNK2 | 1.929694453 | 4.22E-34 |
| ENSG00000217128 | FNIP1 | 1.929927777 | 7.5E-40 |
| ENSG00000175003 | SLC22A1 | 1.931518117 | 0.03021321 |
| ENSG00000139625 | MAP3K12 | 1.931715462 | 5.29E-09 |
| ENSG00000171310 | CHST11 | 1.934069939 | 0.00000012 |
| ENSG00000188152 | NUTM2G | 1.934098272 | 0.00025162 |
| ENSG00000180998 | GPR137C | 1.934107451 | 0.008030283 |
| ENSG00000163141 | BNIPL | 1.934365131 | 0.000815336 |
| ENSG00000279159 | AC003681.1 | 1.938897351 | 0.000186797 |
| ENSG00000040608 | RTN4R | 1.939370109 | 3.92E-31 |
| ENSG00000106089 | STX1A | 1.941300099 | 5.7E-54 |
| ENSG00000204525 | HLA-C | 1.941781682 | 4.21E-10 |
| ENSG00000203875 | SNHG5 | 1.942653778 | 2.84E-67 |
| ENSG00000166016 | ABTB2 | 1.943563122 | 1.44E-51 |
| ENSG00000261114 | AC012181.1 | 1.945556772 | 0.001967414 |
| ENSG00000198380 | GFPT1 | 1.947467477 | 1.03E-50 |
| ENSG00000116514 | RNF19B | 1.947528885 | 1.99E-32 |
| ENSG00000174501 | ANKRD36C | 1.947946919 | 1.06E-16 |
| ENSG00000158246 | FAM46B | 1.948596807 | 0.00000427 |
| ENSG00000154640 | BTG3 | 1.948727293 | 1.65E-31 |
| ENSG00000213139 | CRYGS | 1.950123775 | 0.010704866 |
| ENSG00000136842 | TMOD1 | 1.950344624 | 0.000432696 |
| ENSG00000231683 | AL033397.1 | 1.951966335 | 0.00000132 |
| ENSG00000225614 | ZNF469 | 1.95211035 | 0.009642842 |
| ENSG00000124102 | PI3 | 1.955389186 | 0.000000388 |
| ENSG00000275966 | AC110285.6 | 1.957487459 | 0.000345278 |
| ENSG00000179361 | ARID3B | 1.957831948 | 2.44E-32 |
| ENSG00000137801 | THBS1 | 1.960803924 | 2.58E-26 |
| ENSG00000001084 | GCLC | 1.961033543 | 6.05E-25 |
| ENSG00000253307 | AC011676.1 | 1.965594829 | 0.000169784 |
| ENSG00000162413 | KLHL21 | 1.968661708 | 5.42E-30 |
| ENSG00000111011 | RSRC2 | 1.973822526 | 5.06E-60 |
| ENSG00000273686 |  | 1.978069145 | 4.11E-17 |
| ENSG00000256356 | HSPA8P5 | 1.978461212 | 0.01718985 |
| ENSG00000119801 | YPEL5 | 1.979222319 | 1.58E-43 |
| ENSG00000276517 | AL133243.3 | 1.98119573 | 0.009385793 |
| ENSG00000186567 | CEACAM19 | 1.982955713 | 1.38E-08 |
| ENSG00000145901 | TNIP1 | 1.988882254 | 2.87E-121 |
| ENSG00000260160 | AC011468.1 | 1.990717344 | 0.001021681 |
| ENSG00000166483 | WEE1 | 1.991656486 | 3.59E-39 |
| ENSG00000143590 | EFNA3 | 1.996182918 | 0.00000229 |
| ENSG00000229091 | HSPA8P8 | 1.998068904 | 0.00672922 |
| ENSG00000184205 | TSPYL2 | 2.001246418 | 6.74E-46 |
| ENSG00000135750 | KCNK1 | 2.002478935 | 3.04E-36 |
| ENSG00000116273 | PHF13 | 2.004751331 | 1.01E-49 |
| ENSG00000129355 | CDKN2D | 2.009855928 | 1.03E-19 |
| ENSG00000225201 |  | 2.010217817 | 0.001096831 |
| ENSG00000174529 | TMEM81 | 2.011119532 | 2.84E-15 |
| ENSG00000196507 | TCEAL3 | 2.011151373 | 4.43E-19 |
| ENSG00000179335 | CLK3 | 2.011422829 | 1.01E-37 |
| ENSG00000095739 | BAMBI | 2.012810927 | 1.97E-33 |
| ENSG00000229207 | SERPINH1P1 | 2.013653919 | 0.032004777 |
| ENSG00000156510 | HKDC1 | 2.015827329 | 4.5E-35 |
| ENSG00000171658 | NMRAL2P | 2.017620544 | 0.00000687 |
| ENSG00000159128 | IFNGR2 | 2.017811237 | 2.12E-41 |
| ENSG00000105388 | CEACAM5 | 2.018131887 | 1.8E-21 |
| ENSG00000182759 | MAFA | 2.019112675 | 0.00542032 |
| ENSG00000280138 | AC027290.2 | 2.021725269 | 0.0000001 |
| ENSG00000271303 | SRXN1 | 2.022174616 | 1.21E-11 |
| ENSG00000229152 | ANKRD10-IT1 | 2.022668562 | 2.34E-21 |
| ENSG00000116016 | EPAS1 | 2.022975151 | 3.4E-54 |
| ENSG00000013441 | CLK1 | 2.023067121 | 2.26E-29 |
| ENSG00000131773 | KHDRBS3 | 2.026160123 | 2.42E-08 |
| ENSG00000174775 | HRAS | 2.027485251 | 0.002389506 |
| ENSG00000104888 | SLC17A7 | 2.031526524 | 0.00000436 |
| ENSG00000128228 | SDF2L1 | 2.033251138 | 9.12E-17 |
| ENSG00000273373 | AL355488.1 | 2.034443045 | 0.0000398 |
| ENSG00000205277 | MUC12 | 2.03533785 | 6.21E-08 |
| ENSG00000143669 | LYST | 2.040712365 | 3.13E-18 |
| ENSG00000145050 | MANF | 2.043462374 | 5.78E-09 |
| ENSG00000124224 | PPP4R1L | 2.044644467 | 2.2E-12 |
| ENSG00000213782 | DDX47 | 2.050003419 | 0.015786542 |
| ENSG00000175482 | POLD4 | 2.050322155 | 3.43E-39 |
| ENSG00000106853 | PTGR1 | 2.05068788 | 3.95E-33 |
| ENSG00000128335 | APOL2 | 2.052052707 | 9.11E-63 |
| ENSG00000124145 | SDC4 | 2.052888668 | 1.06E-43 |
| ENSG00000113369 | ARRDC3 | 2.055070205 | 1.46E-30 |
| ENSG00000163435 | ELF3 | 2.056210802 | 2.2E-64 |
| ENSG00000134278 | SPIRE1 | 2.060841071 | 4.27E-46 |
| ENSG00000105856 | HBP1 | 2.062949008 | 1.62E-30 |
| ENSG00000086619 | ERO1B | 2.06338949 | 0.000000109 |
| ENSG00000058085 | LAMC2 | 2.06425593 | 5.32E-09 |
| ENSG00000235748 | SEPT14P12 | 2.065665977 | 0.02849599 |
| ENSG00000117616 | RSRP1 | 2.067389743 | 1.56E-57 |
| ENSG00000106080 | FKBP14 | 2.070048081 | 0.000000513 |
| ENSG00000231721 | LINC-PINT | 2.070401413 | 3.02E-15 |
| ENSG00000106070 | GRB10 | 2.070822191 | 3.29E-18 |
| ENSG00000221926 | TRIM16 | 2.072639788 | 3.26E-40 |
| ENSG00000053747 | LAMA3 | 2.073586598 | 1.75E-44 |
| ENSG00000172059 | KLF11 | 2.074048081 | 3.53E-52 |
| ENSG00000160233 | LRRC3 | 2.075410198 | 0.012897563 |
| ENSG00000115657 | ABCB6 | 2.075954821 | 3.14E-19 |
| ENSG00000183486 | MX2 | 2.077590694 | 0.001333324 |
| ENSG00000197355 | UAP1L1 | 2.07867771 | 2.34E-42 |
| ENSG00000273319 | AC058791.1 | 2.082756035 | 0.047735081 |
| ENSG00000178809 | TRIM73 | 2.086939079 | 0.006931844 |
| ENSG00000103044 | HAS3 | 2.090752514 | 0.00000168 |
| ENSG00000258011 | HMGA1P3 | 2.091296687 | 0.002694625 |
| ENSG00000228109 | MELTF-AS1 | 2.091329523 | 0.003435108 |
| ENSG00000102401 | ARMCX3 | 2.093398916 | 1.43E-63 |
| ENSG00000233429 | HOTAIRM1 | 2.09575284 | 1.3E-27 |
| ENSG00000171970 | ZNF57 | 2.096234795 | 1.06E-18 |
| ENSG00000170485 | NPAS2 | 2.098447153 | 7.39E-76 |
| ENSG00000167565 | SERTAD3 | 2.101305973 | 3.04E-63 |
| ENSG00000114529 | C3orf52 | 2.103126285 | 1.61E-29 |
| ENSG00000188385 | JAKMIP3 | 2.104638246 | 0.032587255 |
| ENSG00000227440 | ATP5MC1P4 | 2.106253885 | 4.81E-08 |
| ENSG00000281183 | NPTN-IT1 | 2.109569201 | 0.000604955 |
| ENSG00000010404 | IDS | 2.112834507 | 2.1E-92 |
| ENSG00000234329 | AL604028.2 | 2.114509712 | 0.014554223 |
| ENSG00000105613 | MAST1 | 2.120557923 | 0.0000354 |
| ENSG00000260805 | AC092803.2 | 2.123793028 | 6.21E-08 |
| ENSG00000117228 | GBP1 | 2.127535018 | 2.37E-11 |
| ENSG00000119630 | PGF | 2.128398282 | 2.22E-09 |
| ENSG00000275993 | SIK1B | 2.13035826 | 1.37E-22 |
| ENSG00000230638 | AL445933.1 | 2.130625211 | 0.031352814 |
| ENSG00000109971 | HSPA8 | 2.13196044 | 5.77E-39 |
| ENSG00000080561 | MID2 | 2.132526358 | 2.24E-13 |
| ENSG00000178150 | ZNF114 | 2.132701197 | 0.023798914 |
| ENSG00000198431 | TXNRD1 | 2.13288708 | 9.52E-80 |
| ENSG00000182379 | NXPH4 | 2.136730006 | 0.000000289 |
| ENSG00000131503 | ANKHD1 | 2.138887846 | 5.4E-20 |
| ENSG00000228686 | AL590723.1 | 2.146366403 | 0.004282957 |
| ENSG00000171291 | ZNF439 | 2.149350065 | 0.048739164 |
| ENSG00000250271 | AC068647.2 | 2.15320451 | 0.000000677 |
| ENSG00000166289 | PLEKHF1 | 2.157624418 | 0.00000133 |
| ENSG00000235233 |  | 2.157668139 | 9.96E-17 |
| ENSG00000229644 | NAMPTP1 | 2.159839367 | 4.25E-39 |
| ENSG00000255717 | SNHG1 | 2.163967687 | 9.08E-32 |
| ENSG00000206446 |  | 2.166769134 | 0.000519236 |
| ENSG00000208028 | MIR616 | 2.16696525 | 0.011493191 |
| ENSG00000144381 | HSPD1 | 2.172663662 | 1.11E-64 |
| ENSG00000179750 | APOBEC3B | 2.177873668 | 2.13E-23 |
| ENSG00000123358 | NR4A1 | 2.177897336 | 3.42E-42 |
| ENSG00000268041 | AC010616.1 | 2.185140885 | 3.22E-08 |
| ENSG00000184005 | ST6GALNAC3 | 2.186485507 | 0.0019734 |
| ENSG00000185507 | IRF7 | 2.187092285 | 0.003333106 |
| ENSG00000256612 | CYP2B7P | 2.191056194 | 0.000000129 |
| ENSG00000129116 | PALLD | 2.193113707 | 1.2E-43 |
| ENSG00000077150 | NFKB2 | 2.198645607 | 1.87E-61 |
| ENSG00000263272 | AC004148.2 | 2.198880568 | 0.003514633 |
| ENSG00000196139 | AKR1C3 | 2.20283342 | 7.26E-27 |
| ENSG00000033327 | GAB2 | 2.203268595 | 2.48E-48 |
| ENSG00000178607 | ERN1 | 2.207122753 | 4.43E-38 |
| ENSG00000134343 | ANO3 | 2.208246698 | 0.000148735 |
| ENSG00000162407 | PLPP3 | 2.210870283 | 2.84E-29 |
| ENSG00000112149 | CD83 | 2.214644211 | 1.93E-09 |
| ENSG00000141738 | GRB7 | 2.215430008 | 2.58E-51 |
| ENSG00000091129 | NRCAM | 2.215772768 | 0.020735721 |
| ENSG00000109814 | UGDH | 2.218078707 | 3.39E-48 |
| ENSG00000242265 | PEG10 | 2.222859857 | 0.009780731 |
| ENSG00000230753 | ZNF341-AS1 | 2.224507779 | 0.010401847 |
| ENSG00000281527 |  | 2.224620759 | 0.024145 |
| ENSG00000069667 | RORA | 2.2300312 | 3.02E-09 |
| ENSG00000185022 | MAFF | 2.230394686 | 6.36E-43 |
| ENSG00000183696 | UPP1 | 2.235330197 | 1.27E-25 |
| ENSG00000221968 | FADS3 | 2.235490085 | 6.85E-46 |
| ENSG00000238120 | LINC01589 | 2.237368581 | 1.09E-08 |
| ENSG00000176463 | SLCO3A1 | 2.237859269 | 4.21E-21 |
| ENSG00000205940 | HSP90AB2P | 2.239526053 | 4.68E-14 |
| ENSG00000141854 | MISP3 | 2.24009498 | 7.82E-24 |
| ENSG00000149212 | SESN3 | 2.240746311 | 2.02E-12 |
| ENSG00000124635 | HIST1H2BJ | 2.240819096 | 1.61E-12 |
| ENSG00000065833 | ME1 | 2.2413457 | 1.05E-46 |
| ENSG00000166173 | LARP6 | 2.243222576 | 1.09E-31 |
| ENSG00000128573 | FOXP2 | 2.244040601 | 0.0000114 |
| ENSG00000118985 | ELL2 | 2.244618694 | 1.03E-94 |
| ENSG00000273284 | AP001033.2 | 2.245331943 | 1.37E-16 |
| ENSG00000281404 | LINC01176 | 2.256525006 | 0.000125439 |
| ENSG00000138678 | GPAT3 | 2.258950305 | 7.91E-65 |
| ENSG00000127948 | POR | 2.263429503 | 1.04E-60 |
| ENSG00000163710 | PCOLCE2 | 2.266354127 | 1.48E-16 |
| ENSG00000137502 | RAB30 | 2.268561334 | 3.57E-64 |
| ENSG00000123975 | CKS2 | 2.270003997 | 4.7E-23 |
| ENSG00000165113 | GKAP1 | 2.270117782 | 1.54E-15 |
| ENSG00000123689 | G0S2 | 2.27148371 | 0.00000252 |
| ENSG00000154917 | RAB6B | 2.271763974 | 6.49E-08 |
| ENSG00000162616 | DNAJB4 | 2.272189325 | 1.2E-58 |
| ENSG00000115541 | HSPE1 | 2.272898713 | 1.99E-28 |
| ENSG00000197989 | SNHG12 | 2.274744874 | 4.73E-38 |
| ENSG00000279861 | AC073548.1 | 2.276988006 | 0.002075681 |
| ENSG00000163132 | MSX1 | 2.277573058 | 0.005042865 |
| ENSG00000169213 | RAB3B | 2.281096011 | 2.37E-12 |
| ENSG00000274825 | AL023803.2 | 2.281651939 | 0.001978522 |
| ENSG00000279491 | AP003733.4 | 2.281739081 | 3.56E-12 |
| ENSG00000162783 | IER5 | 2.287752396 | 3.44E-37 |
| ENSG00000229431 | AL139289.1 | 2.288151681 | 0.001429094 |
| ENSG00000256304 | CCDC150P1 | 2.29158244 | 0.000000187 |
| ENSG00000273338 | AC103591.3 | 2.293093281 | 0.023202035 |
| ENSG00000132680 | KHDC4 | 2.29716227 | 4.95E-51 |
| ENSG00000131015 | ULBP2 | 2.301213011 | 5.92E-45 |
| ENSG00000219891 | ZSCAN12P1 | 2.303214823 | 1.91E-12 |
| ENSG00000143178 | TBX19 | 2.305037041 | 5.13E-14 |
| ENSG00000260708 | AL118516.1 | 2.30780064 | 7.65E-20 |
| ENSG00000116717 | GADD45A | 2.307897266 | 1.09E-78 |
| ENSG00000269439 | AC010618.3 | 2.308529365 | 0.000004 |
| ENSG00000156804 | FBXO32 | 2.313910004 | 1.23E-78 |
| ENSG00000227591 | AL031316.1 | 2.318429066 | 0.0000213 |
| ENSG00000110811 | P3H3 | 2.318832241 | 0.0000605 |
| ENSG00000137094 | DNAJB5 | 2.319881035 | 2.17E-18 |
| ENSG00000152409 | JMY | 2.320592466 | 8.85E-59 |
| ENSG00000168298 | HIST1H1E | 2.320631632 | 0.000957082 |
| ENSG00000163734 | CXCL3 | 2.32394422 | 2.28E-15 |
| ENSG00000255120 | OVOL1-AS1 | 2.324170832 | 0.048630659 |
| ENSG00000184260 | HIST2H2AC | 2.325370312 | 0.002617182 |
| ENSG00000196352 | CD55 | 2.32739161 | 1.18E-41 |
| ENSG00000105974 | CAV1 | 2.327782259 | 3.5E-17 |
| ENSG00000151692 | RNF144A | 2.331435317 | 0.001886567 |
| ENSG00000067064 | IDI1 | 2.331947669 | 0.000000501 |
| ENSG00000100867 | DHRS2 | 2.333264294 | 7.42E-39 |
| ENSG00000276710 | CSPG4P10 | 2.333465278 | 1.99E-12 |
| ENSG00000092607 | TBX15 | 2.333916014 | 0.014845075 |
| ENSG00000054598 | FOXC1 | 2.337907807 | 7.49E-55 |
| ENSG00000268812 | AC004264.1 | 2.341593125 | 3.77E-08 |
| ENSG00000244055 | AC007566.1 | 2.344935905 | 0.004302677 |
| ENSG00000225553 |  | 2.34663044 | 2.02E-17 |
| ENSG00000269855 | RNF225 | 2.34866518 | 0.001748214 |
| ENSG00000230204 | FTH1P5 | 2.348849943 | 0.00000875 |
| ENSG00000237330 | RNF223 | 2.350843704 | 3.58E-39 |
| ENSG00000213928 | IRF9 | 2.351251563 | 0.0000197 |
| ENSG00000171903 | CYP4F11 | 2.353022852 | 0.0000199 |
| ENSG00000107864 | CPEB3 | 2.354481389 | 4.64E-53 |
| ENSG00000203709 | MIR29B2CHG | 2.355095008 | 0.000233833 |
| ENSG00000269899 | AC025857.2 | 2.360646526 | 0.027409202 |
| ENSG00000107201 | DDX58 | 2.361104653 | 3.23E-22 |
| ENSG00000260103 | AC012435.1 | 2.362135372 | 0.038325199 |
| ENSG00000244486 | SCARF2 | 2.363597186 | 0.004160129 |
| ENSG00000225855 | RUSC1-AS1 | 2.365098178 | 4.98E-11 |
| ENSG00000133639 | BTG1 | 2.365540348 | 4.65E-56 |
| ENSG00000224411 | HSP90AA2P | 2.369972974 | 7.1E-09 |
| ENSG00000262519 | TXNP4 | 2.370212795 | 0.003659905 |
| ENSG00000146232 | NFKBIE | 2.370299741 | 8.96E-67 |
| ENSG00000160570 | DEDD2 | 2.373281231 | 3.11E-53 |
| ENSG00000171522 | PTGER4 | 2.374649778 | 0.002919942 |
| ENSG00000128590 | DNAJB9 | 2.375434908 | 7.12E-61 |
| ENSG00000187837 | HIST1H1C | 2.375593116 | 3.5E-31 |
| ENSG00000170961 | HAS2 | 2.378850355 | 0.001073617 |
| ENSG00000130203 | APOE | 2.388268649 | 0.000000216 |
| ENSG00000274290 | HIST1H2BE | 2.391370539 | 0.024651831 |
| ENSG00000112182 | BACH2 | 2.39461886 | 0.008159738 |
| ENSG00000235677 | NPM1P26 | 2.396903932 | 0.020536861 |
| ENSG00000235029 | MNX1-AS2 | 2.401214336 | 0.001975721 |
| ENSG00000232126 |  | 2.404174996 | 9.43E-13 |
| ENSG00000257576 | HSPD1P4 | 2.404387015 | 1.7E-09 |
| ENSG00000213430 | HSPD1P1 | 2.40491921 | 2.34E-19 |
| ENSG00000115226 | FNDC4 | 2.405241319 | 0.031574776 |
| ENSG00000255026 | AC136475.3 | 2.406846576 | 1.08E-30 |
| ENSG00000169469 | SPRR1B | 2.411203544 | 0.01777255 |
| ENSG00000028277 | POU2F2 | 2.41209653 | 5.63E-13 |
| ENSG00000165521 | EML5 | 2.412366729 | 0.034972226 |
| ENSG00000131016 | AKAP12 | 2.416024153 | 5.69E-29 |
| ENSG00000130513 | GDF15 | 2.417991134 | 1.23E-28 |
| ENSG00000121653 | MAPK8IP1 | 2.41955713 | 0.00000995 |
| ENSG00000225536 | STIP1P3 | 2.425737503 | 7.5E-11 |
| ENSG00000114796 | KLHL24 | 2.428219198 | 4.79E-48 |
| ENSG00000165757 | JCAD | 2.428510886 | 5.63E-39 |
| ENSG00000277224 | HIST1H2BF | 2.42969279 | 0.043887531 |
| ENSG00000267879 | AC011483.1 | 2.43212013 | 0.0000373 |
| ENSG00000180667 | YOD1 | 2.438753337 | 1.34E-22 |
| ENSG00000164463 | CREBRF | 2.439781873 | 1.09E-54 |
| ENSG00000197208 | SLC22A4 | 2.444978393 | 0.007132414 |
| ENSG00000176076 | KCNE5 | 2.44520349 | 0.000675819 |
| ENSG00000231966 | AL359853.2 | 2.448167274 | 0.005974001 |
| ENSG00000169242 | EFNA1 | 2.45768018 | 1.95E-79 |
| ENSG00000232368 | FTLP2 | 2.458022887 | 0.00290232 |
| ENSG00000276900 | AC023157.3 | 2.468540234 | 1.96E-18 |
| ENSG00000135605 | TEC | 2.470648635 | 6.39E-38 |
| ENSG00000164070 | HSPA4L | 2.47067737 | 2.92E-47 |
| ENSG00000213362 | FTH1P12 | 2.471098103 | 4.7E-13 |
| ENSG00000104549 | SQLE | 2.476018076 | 7.38E-09 |
| ENSG00000260257 | AL035071.1 | 2.482957258 | 1.44E-09 |
| ENSG00000197576 | HOXA4 | 2.482988479 | 7.99E-08 |
| ENSG00000196890 | HIST3H2BB | 2.485516686 | 2.25E-08 |
| ENSG00000184357 | HIST1H1B | 2.487681953 | 0.002092974 |
| ENSG00000275894 | AL021578.1 | 2.487810832 | 0.004451514 |
| ENSG00000169035 | KLK7 | 2.48826451 | 0.0000678 |
| ENSG00000279568 | AC093525.9 | 2.490135773 | 0.006665499 |
| ENSG00000280047 | AC091825.1 | 2.491608032 | 0.00154531 |
| ENSG00000164683 | HEY1 | 2.492236037 | 0.0000431 |
| ENSG00000183496 | MEX3B | 2.495043211 | 2.9E-28 |
| ENSG00000198074 | AKR1B10 | 2.500498108 | 8.82E-12 |
| ENSG00000112096 | SOD2 | 2.501545564 | 1.49E-122 |
| ENSG00000223552 | AC098613.1 | 2.50188907 | 1.26E-13 |
| ENSG00000136695 | IL36RN | 2.503119943 | 0.00000005 |
| ENSG00000125347 | IRF1 | 2.504065045 | 1.51E-74 |
| ENSG00000151743 | AMN1 | 2.509623178 | 8.37E-35 |
| ENSG00000174521 | TTC9B | 2.513316378 | 0.001421701 |
| ENSG00000230037 | UBBP1 | 2.514343758 | 0.006351013 |
| ENSG00000137449 | CPEB2 | 2.519918833 | 4.9E-58 |
| ENSG00000135549 | PKIB | 2.521874638 | 1.6E-95 |
| ENSG00000137285 | TUBB2B | 2.526528015 | 6.89E-11 |
| ENSG00000176428 | VPS37D | 2.527922751 | 1.06E-19 |
| ENSG00000259746 | HSPE1P3 | 2.533718094 | 0.001212793 |
| ENSG00000008294 | SPAG9 | 2.537479852 | 1.78E-85 |
| ENSG00000158470 | B4GALT5 | 2.537919356 | 5.48E-64 |
| ENSG00000223461 | AC004471.1 | 2.538224067 | 0.0000957 |
| ENSG00000170955 | CAVIN3 | 2.539132718 | 0.035920975 |
| ENSG00000138670 | RASGEF1B | 2.540745581 | 4.88E-09 |
| ENSG00000162733 | DDR2 | 2.541321344 | 0.000403689 |
| ENSG00000121743 | GJA3 | 2.542430452 | 3.37E-08 |
| ENSG00000196866 | HIST1H2AD | 2.544440235 | 0.040370715 |
| ENSG00000171444 | MCC | 2.545304684 | 0.00014494 |
| ENSG00000142156 | COL6A1 | 2.547318803 | 0.00000364 |
| ENSG00000233588 | CYP51A1P2 | 2.551353252 | 0.037450861 |
| ENSG00000025708 | TYMP | 2.552002664 | 9.48E-13 |
| ENSG00000197153 | HIST1H3J | 2.555402024 | 0.007703392 |
| ENSG00000112110 | MRPL18 | 2.556063337 | 1.57E-50 |
| ENSG00000231434 | AL365440.1 | 2.556692435 | 0.005794874 |
| ENSG00000124875 | CXCL6 | 2.559451625 | 0.000156413 |
| ENSG00000251562 | MALAT1 | 2.559831817 | 4.31E-43 |
| ENSG00000063660 | GPC1 | 2.560216366 | 9.81E-58 |
| ENSG00000273356 | LINC02019 | 2.56200173 | 0.00613894 |
| ENSG00000102057 | KCND1 | 2.563475549 | 0.0000283 |
| ENSG00000095637 | SORBS1 | 2.571467091 | 8.64E-13 |
| ENSG00000165195 | PIGA | 2.572680074 | 1.38E-29 |
| ENSG00000219747 | AL133260.1 | 2.574177458 | 0.0141759 |
| ENSG00000276916 | AL442125.2 | 2.578296789 | 0.00000671 |
| ENSG00000125046 | SSUH2 | 2.580462084 | 0.022032243 |
| ENSG00000134070 | IRAK2 | 2.582552457 | 5.64E-60 |
| ENSG00000011638 | TMEM159 | 2.583888286 | 7.75E-70 |
| ENSG00000142102 | PGGHG | 2.586423042 | 5.48E-61 |
| ENSG00000100625 | SIX4 | 2.588090635 | 4.34E-32 |
| ENSG00000245156 | AP001107.1 | 2.589192725 | 0.011569367 |
| ENSG00000225693 | LAGE3P1 | 2.591738336 | 0.037396225 |
| ENSG00000088448 | ANKRD10 | 2.592660743 | 7.49E-55 |
| ENSG00000104856 | RELB | 2.595368426 | 7.99E-58 |
| ENSG00000053108 | FSTL4 | 2.597709948 | 0.006387295 |
| ENSG00000165030 | NFIL3 | 2.603449803 | 5.76E-90 |
| ENSG00000276402 |  | 2.603875934 | 0.020014495 |
| ENSG00000100439 | ABHD4 | 2.60540694 | 2.12E-56 |
| ENSG00000070495 | JMJD6 | 2.607461751 | 8.17E-45 |
| ENSG00000272677 | AC124016.1 | 2.610252324 | 0.000120527 |
| ENSG00000091986 | CCDC80 | 2.619784462 | 0.00000245 |
| ENSG00000217801 | AL390719.1 | 2.620406873 | 5.46E-33 |
| ENSG00000223573 | TINCR | 2.626406897 | 0.017131789 |
| ENSG00000230074 | AL162231.2 | 2.629717066 | 1.41E-16 |
| ENSG00000153132 | CLGN | 2.633804211 | 3.24E-16 |
| ENSG00000216480 | AL078604.1 | 2.634329762 | 0.014349221 |
| ENSG00000261270 | AC012181.2 | 2.636985072 | 3.99E-08 |
| ENSG00000263513 | FAM72C | 2.637657386 | 0.003631651 |
| ENSG00000176383 | B3GNT4 | 2.639020783 | 0.00000111 |
| ENSG00000258947 | TUBB3 | 2.642424814 | 1.53E-08 |
| ENSG00000166963 | MAP1A | 2.653615912 | 6.07E-12 |
| ENSG00000004478 | FKBP4 | 2.656162019 | 4.56E-63 |
| ENSG00000271755 | AL031118.1 | 2.660275333 | 0.018469467 |
| ENSG00000198355 | PIM3 | 2.660335929 | 1.2E-114 |
| ENSG00000176438 | SYNE3 | 2.66721238 | 0.000000332 |
| ENSG00000273199 | AP000692.2 | 2.669585495 | 0.007231553 |
| ENSG00000222937 | SNORD63B | 2.67415177 | 0.015843405 |
| ENSG00000226608 | FTLP3 | 2.680276254 | 1.74E-24 |
| ENSG00000145911 | N4BP3 | 2.680752095 | 1.66E-38 |
| ENSG00000080824 | HSP90AA1 | 2.684123471 | 1.81E-65 |
| ENSG00000142227 | EMP3 | 2.68718977 | 2.25E-19 |
| ENSG00000135047 | CTSL | 2.696158525 | 8.77E-41 |
| ENSG00000128016 | ZFP36 | 2.703932078 | 7.71E-82 |
| ENSG00000261505 | AL031714.1 | 2.704207841 | 1.08E-19 |
| ENSG00000277075 | HIST1H2AE | 2.70995383 | 2.24E-10 |
| ENSG00000131480 | AOC2 | 2.713348258 | 7.55E-12 |
| ENSG00000269911 | FAM226B | 2.715167736 | 0.000216921 |
| ENSG00000172818 | OVOL1 | 2.716022566 | 4.45E-102 |
| ENSG00000073150 | PANX2 | 2.723057229 | 2.97E-74 |
| ENSG00000224501 |  | 2.727454755 | 1.42E-20 |
| ENSG00000087086 | FTL | 2.728412426 | 5.92E-51 |
| ENSG00000167550 | RHEBL1 | 2.737135435 | 2.7E-14 |
| ENSG00000178127 | NDUFV2 | 2.737428477 | 8.36E-14 |
| ENSG00000124466 | LYPD3 | 2.738683334 | 7.08E-16 |
| ENSG00000274976 | AC087588.2 | 2.739625388 | 0.000088 |
| ENSG00000102385 | DRP2 | 2.7426321 | 0.000770031 |
| ENSG00000237522 | NONOP2 | 2.747476109 | 0.000000378 |
| ENSG00000239636 | AC004865.2 | 2.749881182 | 0.039896669 |
| ENSG00000148339 | SLC25A25 | 2.752547369 | 3.72E-124 |
| ENSG00000162654 | GBP4 | 2.755266667 | 0.000697347 |
| ENSG00000166046 | TCP11L2 | 2.756112164 | 1.04E-60 |
| ENSG00000167995 | BEST1 | 2.758872636 | 0.000565439 |
| ENSG00000162772 | ATF3 | 2.761790189 | 1.35E-12 |
| ENSG00000188089 | PLA2G4E | 2.773307836 | 0.000308758 |
| ENSG00000162775 | RBM15 | 2.773331475 | 6.86E-32 |
| ENSG00000107731 | UNC5B | 2.776126255 | 0.00000533 |
| ENSG00000223531 |  | 2.777012309 | 0.0000917 |
| ENSG00000153714 | LURAP1L | 2.789193488 | 6.65E-36 |
| ENSG00000271869 | AC026979.2 | 2.789998822 | 0.0000314 |
| ENSG00000069399 | BCL3 | 2.793611293 | 1.2E-60 |
| ENSG00000104419 | NDRG1 | 2.793717425 | 1.89E-107 |
| ENSG00000242028 | HYPK | 2.798026322 | 4.21E-08 |
| ENSG00000270184 | AC018695.4 | 2.812447689 | 0.033880324 |
| ENSG00000118785 | SPP1 | 2.813415245 | 0.00000218 |
| ENSG00000232187 | FTH1P7 | 2.813985796 | 6.28E-58 |
| ENSG00000121281 | ADCY7 | 2.816009969 | 6.45E-21 |
| ENSG00000118194 | TNNT2 | 2.822107006 | 0.003371929 |
| ENSG00000223361 | FTH1P10 | 2.822363096 | 8.09E-20 |
| ENSG00000163739 | CXCL1 | 2.822477871 | 3.93E-17 |
| ENSG00000136457 | CHAD | 2.829099169 | 0.000389339 |
| ENSG00000213606 | AKR1B10P1 | 2.832678663 | 2.87E-20 |
| ENSG00000149201 | CCDC81 | 2.835382375 | 0.0000541 |
| ENSG00000167723 | TRPV3 | 2.835403479 | 1.09E-28 |
| ENSG00000226704 |  | 2.837123713 | 0.001195746 |
| ENSG00000087494 | PTHLH | 2.838095253 | 8.74E-17 |
| ENSG00000249992 | TMEM158 | 2.839539564 | 0.000165748 |
| ENSG00000151929 | BAG3 | 2.840895849 | 1.14E-91 |
| ENSG00000135046 | ANXA1 | 2.843871941 | 1.43E-133 |
| ENSG00000141574 | SECTM1 | 2.848858522 | 6.87E-10 |
| ENSG00000263931 |  | 2.849629768 | 0.022890233 |
| ENSG00000259380 | AC087473.1 | 2.862318402 | 0.049100988 |
| ENSG00000232713 | AC010733.1 | 2.865890882 | 0.001368056 |
| ENSG00000139725 | RHOF | 2.867092044 | 1.48E-86 |
| ENSG00000230216 | HSPB1P2 | 2.870483616 | 8.64E-11 |
| ENSG00000112511 | PHF1 | 2.872402772 | 0.011034054 |
| ENSG00000226564 | FTH1P20 | 2.875365392 | 6.79E-58 |
| ENSG00000072864 | NDE1 | 2.87626938 | 0.026096479 |
| ENSG00000105327 | BBC3 | 2.880653861 | 1.67E-83 |
| ENSG00000259771 | AC092756.1 | 2.883966353 | 0.004474914 |
| ENSG00000181359 | HSP90AA6P | 2.887327894 | 0.002970968 |
| ENSG00000005513 | SOX8 | 2.890470239 | 8.75E-16 |
| ENSG00000242960 | FTH1P23 | 2.892732147 | 8.69E-42 |
| ENSG00000225880 | LINC00115 | 2.901990473 | 7.57E-11 |
| ENSG00000125730 | C3 | 2.905418359 | 0.004490552 |
| ENSG00000147872 | PLIN2 | 2.90712833 | 3.61E-55 |
| ENSG00000263934 | SNORD3A | 2.913061241 | 0.0000448 |
| ENSG00000179111 | HES7 | 2.913137606 | 0.0000413 |
| ENSG00000279140 | AL590326.1 | 2.915650864 | 0.030301112 |
| ENSG00000270257 | AC096720.1 | 2.920165426 | 0.046194441 |
| ENSG00000041982 | TNC | 2.923791959 | 1.97E-20 |
| ENSG00000167996 | FTH1 | 2.937700855 | 4.77E-142 |
| ENSG00000262147 | AC124283.2 | 2.94528025 | 0.000324989 |
| ENSG00000023839 | ABCC2 | 2.949909875 | 0.00000144 |
| ENSG00000124194 | GDAP1L1 | 2.950519736 | 0.014623429 |
| ENSG00000120217 | CD274 | 2.956620681 | 9.02E-16 |
| ENSG00000213453 | FTH1P3 | 2.957242594 | 3.08E-15 |
| ENSG00000131018 | SYNE1 | 2.959348038 | 1.86E-44 |
| ENSG00000272512 | AL645608.8 | 2.966393762 | 0.000240034 |
| ENSG00000081189 | MEF2C | 2.971171574 | 0.002774485 |
| ENSG00000130066 | SAT1 | 2.980573452 | 1.56E-62 |
| ENSG00000110944 | IL23A | 2.985109186 | 0.00000169 |
| ENSG00000229473 | RGS17P1 | 2.986804882 | 0.000957298 |
| ENSG00000185955 | C7orf61 | 2.988043555 | 0.0000191 |
| ENSG00000231555 |  | 2.989377104 | 1.38E-10 |
| ENSG00000275530 |  | 2.992306285 | 0.0000981 |
| ENSG00000229656 | ITGB1-DT | 2.993991907 | 0.006486697 |
| ENSG00000272812 | AC004908.2 | 2.994397438 | 0.000189464 |
| ENSG00000108771 | DHX58 | 2.99781373 | 2.07E-39 |
| ENSG00000251493 | FOXD1 | 2.999662634 | 6.01E-10 |
| ENSG00000234975 | FTH1P2 | 2.999779473 | 1.04E-54 |
| ENSG00000023902 | PLEKHO1 | 3.007048089 | 0.001411434 |
| ENSG00000149257 | SERPINH1 | 3.013600494 | 2.27E-51 |
| ENSG00000090339 | ICAM1 | 3.014390102 | 3.75E-43 |
| ENSG00000143067 | ZNF697 | 3.016078423 | 1.9E-73 |
| ENSG00000188290 | HES4 | 3.021169337 | 6.39E-50 |
| ENSG00000237264 | FTH1P11 | 3.027101267 | 4.72E-36 |
| ENSG00000176597 | B3GNT5 | 3.029518239 | 2.35E-84 |
| ENSG00000243742 | RPLP0P2 | 3.0296289 | 0.000000845 |
| ENSG00000219507 | FTH1P8 | 3.033495861 | 1.49E-52 |
| ENSG00000212866 |  | 3.034279453 | 4.47E-59 |
| ENSG00000163536 | SERPINI1 | 3.034618376 | 1.08E-08 |
| ENSG00000278918 | AC080112.3 | 3.036338458 | 0.000545427 |
| ENSG00000112773 | FAM46A | 3.037197424 | 1.34E-75 |
| ENSG00000271815 | AC008897.3 | 3.038376431 | 0.034719332 |
| ENSG00000015520 | NPC1L1 | 3.041442023 | 0.000279613 |
| ENSG00000258615 | AL117187.1 | 3.042557839 | 0.035047101 |
| ENSG00000258153 | HSPE1P4 | 3.063649356 | 0.000701174 |
| ENSG00000140022 | STON2 | 3.067165455 | 2.73E-37 |
| ENSG00000110172 | CHORDC1 | 3.073287031 | 1.94E-58 |
| ENSG00000197380 | DACT3 | 3.079358622 | 0.021747708 |
| ENSG00000188042 | ARL4C | 3.079865649 | 1.74E-38 |
| ENSG00000048740 | CELF2 | 3.081476244 | 0.024099953 |
| ENSG00000125657 | TNFSF9 | 3.085515979 | 1.36E-21 |
| ENSG00000269825 | AC022150.4 | 3.09023981 | 2.11E-18 |
| ENSG00000171608 | PIK3CD | 3.10211423 | 2.99E-14 |
| ENSG00000279901 | AC092117.2 | 3.121504025 | 0.002709445 |
| ENSG00000161011 | SQSTM1 | 3.123857878 | 2.97E-258 |
| ENSG00000187498 | COL4A1 | 3.127787557 | 0.008499035 |
| ENSG00000112972 | HMGCS1 | 3.141758624 | 7.54E-12 |
| ENSG00000242992 | FTH1P4 | 3.146023044 | 0.00033178 |
| ENSG00000274810 | NPHP3-ACAD11 | 3.154596619 | 0.029761186 |
| ENSG00000280162 | AL136531.3 | 3.166399902 | 0.003640734 |
| ENSG00000105939 | ZC3HAV1 | 3.17500139 | 5.2E-158 |
| ENSG00000146278 | PNRC1 | 3.175683426 | 3.9E-106 |
| ENSG00000104140 | RHOV | 3.180209322 | 4.05E-70 |
| ENSG00000160097 | FNDC5 | 3.180347493 | 0.036968157 |
| ENSG00000260604 | AL590004.4 | 3.18144114 | 0.000117149 |
| ENSG00000115009 | CCL20 | 3.183916145 | 1.64E-10 |
| ENSG00000141682 | PMAIP1 | 3.184123941 | 7.17E-96 |
| ENSG00000135842 | FAM129A | 3.185266179 | 7.17E-85 |
| ENSG00000237499 | AL357060.1 | 3.18645203 | 0.040735544 |
| ENSG00000279821 | AC145098.2 | 3.18906827 | 1.89E-11 |
| ENSG00000085563 | ABCB1 | 3.193946702 | 0.000900112 |
| ENSG00000262074 | SNORD3B-2 | 3.197308505 | 0.001178743 |
| ENSG00000260592 | AC130456.3 | 3.205034422 | 0.009796167 |
| ENSG00000078018 | MAP2 | 3.20882206 | 1.63E-26 |
| ENSG00000124256 | ZBP1 | 3.211919564 | 0.027170795 |
| ENSG00000124575 | HIST1H1D | 3.213629377 | 0.000370849 |
| ENSG00000177173 | NAP1L4P1 | 3.216707201 | 6.47E-15 |
| ENSG00000274204 | AL138689.1 | 3.22140883 | 0.004372809 |
| ENSG00000282034 | AC106886.5 | 3.222408096 | 1.35E-12 |
| ENSG00000241322 | CDRT1 | 3.238432311 | 0.041732003 |
| ENSG00000227376 | FTH1P16 | 3.239730883 | 1.13E-23 |
| ENSG00000223532 |  | 3.241654102 | 3.78E-16 |
| ENSG00000044574 | HSPA5 | 3.250249449 | 4.78E-75 |
| ENSG00000135919 | SERPINE2 | 3.250962802 | 1.08E-94 |
| ENSG00000120129 | DUSP1 | 3.252698991 | 7.2E-17 |
| ENSG00000184844 | CYCSP45 | 3.253036448 | 0.048355183 |
| ENSG00000173389 | IQCF1 | 3.253720038 | 0.005718629 |
| ENSG00000186642 | PDE2A | 3.253905703 | 0.002400362 |
| ENSG00000152128 | TMEM163 | 3.254096873 | 0.012156098 |
| ENSG00000265112 | MIR3153 | 3.25812853 | 0.021178626 |
| ENSG00000277765 |  | 3.260992373 | 0.001257668 |
| ENSG00000242866 | STRC | 3.266580892 | 0.014306918 |
| ENSG00000277687 | AL139407.1 | 3.280348305 | 0.000188415 |
| ENSG00000023909 | GCLM | 3.286503896 | 1.91E-139 |
| ENSG00000185467 | KPNA7 | 3.286762526 | 1.25E-26 |
| ENSG00000225721 | AL592166.1 | 3.288536725 | 0.001213115 |
| ENSG00000202538 | RNU4-2 | 3.289707987 | 0.02252135 |
| ENSG00000134668 | SPOCD1 | 3.303747084 | 0.001272762 |
| ENSG00000099251 | HSD17B7P2 | 3.307884341 | 3.99E-26 |
| ENSG00000145780 | FEM1C | 3.31231309 | 2.54E-77 |
| ENSG00000229808 | AL391825.1 | 3.312475216 | 2.04E-08 |
| ENSG00000275294 | LINC02340 | 3.321458242 | 0.02210081 |
| ENSG00000151014 | NOCT | 3.33425883 | 4.38E-116 |
| ENSG00000280367 | AP002364.1 | 3.3345702 | 0.006493892 |
| ENSG00000106211 | HSPB1 | 3.353964641 | 4.05E-70 |
| ENSG00000100906 | NFKBIA | 3.355989667 | 3.18E-190 |
| ENSG00000272078 | AL139423.1 | 3.356452829 | 5.35E-12 |
| ENSG00000181016 | LSMEM1 | 3.363906948 | 1.25E-36 |
| ENSG00000087074 | PPP1R15A | 3.378618607 | 4.51E-67 |
| ENSG00000241095 | CYP51A1P1 | 3.379771238 | 0.033706739 |
| ENSG00000250746 | AC105250.1 | 3.382380445 | 2.17E-12 |
| ENSG00000124762 | CDKN1A | 3.383566835 | 1.36E-65 |
| ENSG00000265136 | AC124283.4 | 3.383983112 | 0.016821539 |
| ENSG00000260896 | LINC02170 | 3.402831875 | 0.007517591 |
| ENSG00000278811 | LINC00624 | 3.402834704 | 0.00077072 |
| ENSG00000197580 | BCO2 | 3.402931464 | 0.0000663 |
| ENSG00000218980 | FTH1P15 | 3.405650387 | 4.89E-19 |
| ENSG00000152894 | PTPRK | 3.407712076 | 0.0000641 |
| ENSG00000167216 | KATNAL2 | 3.408773765 | 0.001456516 |
| ENSG00000253227 | AC090192.2 | 3.409151746 | 0.001196376 |
| ENSG00000267270 | PARD6G-AS1 | 3.41533457 | 1.29E-33 |
| ENSG00000011422 | PLAUR | 3.426137644 | 2.61E-60 |
| ENSG00000143507 | DUSP10 | 3.432771574 | 4.96E-35 |
| ENSG00000225808 | DNAJC19P5 | 3.433439766 | 0.001898034 |
| ENSG00000279066 | HEXDC-IT1 | 3.436583286 | 0.0136832 |
| ENSG00000241255 | AL136126.1 | 3.436705994 | 0.016385679 |
| ENSG00000120094 | HOXB1 | 3.438629754 | 0.029984854 |
| ENSG00000181773 | GPR3 | 3.448547228 | 8.48E-34 |
| ENSG00000152463 | OLAH | 3.457630676 | 0.031202167 |
| ENSG00000255150 | EID3 | 3.468340618 | 0.000000378 |
| ENSG00000279247 |  | 3.468676792 | 0.00499727 |
| ENSG00000182870 | GALNT9 | 3.497929906 | 0.006440262 |
| ENSG00000204666 | AC010624.1 | 3.50489898 | 0.016143964 |
| ENSG00000267568 | AC016168.2 | 3.520847791 | 0.00129583 |
| ENSG00000152503 | TRIM36 | 3.524031289 | 9.69E-24 |
| ENSG00000162998 | FRZB | 3.52570482 | 0.004612728 |
| ENSG00000179094 | PER1 | 3.529697235 | 6.43E-53 |
| ENSG00000207524 | RNU6-33P | 3.53155865 | 0.008674934 |
| ENSG00000259799 |  | 3.532691043 | 0.010827916 |
| ENSG00000250588 |  | 3.532831627 | 0.000000159 |
| ENSG00000276867 | AC074050.4 | 3.538424632 | 0.009180671 |
| ENSG00000235790 | AC114488.2 | 3.543831075 | 0.000175198 |
| ENSG00000261087 | AP003469.4 | 3.574534713 | 1.96E-48 |
| ENSG00000229056 | AC020571.1 | 3.577844585 | 0.025729729 |
| ENSG00000180777 | ANKRD30B | 3.578073001 | 2.72E-13 |
| ENSG00000278000 | AC139100.2 | 3.589355924 | 0.000419834 |
| ENSG00000138166 | DUSP5 | 3.599761135 | 6.58E-71 |
| ENSG00000235586 | AC011247.2 | 3.609582879 | 0.011966409 |
| ENSG00000214145 | LINC00887 | 3.616251155 | 0.000000986 |
| ENSG00000011590 | ZBTB32 | 3.621397736 | 0.030990666 |
| ENSG00000116701 | NCF2 | 3.622683484 | 0.028678978 |
| ENSG00000095752 | IL11 | 3.628006832 | 1.52E-13 |
| ENSG00000184494 |  | 3.628478958 | 0.000000399 |
| ENSG00000263847 | AP005899.1 | 3.640078621 | 0.00000397 |
| ENSG00000153814 | JAZF1 | 3.645029787 | 0.0000104 |
| ENSG00000276523 | AC025287.3 | 3.645882911 | 0.040064726 |
| ENSG00000274267 | HIST1H3B | 3.648499536 | 0.001606614 |
| ENSG00000225339 | AL354740.1 | 3.649904233 | 0.031611891 |
| ENSG00000259726 | CSPG4P11 | 3.650595221 | 0.000508933 |
| ENSG00000249867 | AC090833.1 | 3.656716016 | 0.018469187 |
| ENSG00000279283 | AC131009.4 | 3.657232579 | 0.000352157 |
| ENSG00000206625 | RNU6-1 | 3.659375056 | 0.028553165 |
| ENSG00000234176 | HSPA8P1 | 3.662987071 | 0.000447123 |
| ENSG00000232034 | AC092168.2 | 3.684619366 | 0.04489569 |
| ENSG00000259630 | AC104046.1 | 3.705703018 | 0.002144553 |
| ENSG00000126368 | NR1D1 | 3.710089958 | 8.99E-72 |
| ENSG00000215328 |  | 3.712184485 | 7.65E-14 |
| ENSG00000165312 | OTUD1 | 3.716038413 | 1.81E-84 |
| ENSG00000111729 | CLEC4A | 3.725322233 | 0.010279244 |
| ENSG00000260051 | AL031600.1 | 3.733110456 | 0.000159262 |
| ENSG00000243498 | UBA52P5 | 3.734941302 | 0.043314619 |
| ENSG00000136826 | KLF4 | 3.737528867 | 2.04E-29 |
| ENSG00000277548 | AC018926.3 | 3.739531039 | 0.031191431 |
| ENSG00000139112 | GABARAPL1 | 3.742949854 | 4.67E-199 |
| ENSG00000136002 | ARHGEF4 | 3.743586895 | 0.004109451 |
| ENSG00000279110 | AL022323.4 | 3.7452782 | 0.014435743 |
| ENSG00000236849 | LINC01474 | 3.746260153 | 0.032236848 |
| ENSG00000236047 | AC073410.1 | 3.749037586 | 0.001363087 |
| ENSG00000231904 |  | 3.751890408 | 0.036067572 |
| ENSG00000185338 | SOCS1 | 3.756393666 | 3.24E-28 |
| ENSG00000146966 | DENND2A | 3.758357108 | 0.035397452 |
| ENSG00000269463 | AP001160.4 | 3.760254165 | 0.033544743 |
| ENSG00000260459 | FTLP14 | 3.766003571 | 1.67E-13 |
| ENSG00000108821 | COL1A1 | 3.768119658 | 2.73E-40 |
| ENSG00000075618 | FSCN1 | 3.771511221 | 5.68E-78 |
| ENSG00000213089 | PDCL3P5 | 3.772363203 | 0.019356871 |
| ENSG00000253686 | LINC01484 | 3.7760136 | 0.032987218 |
| ENSG00000089127 | OAS1 | 3.776573344 | 2.51E-111 |
| ENSG00000175197 | DDIT3 | 3.792726235 | 3.86E-79 |
| ENSG00000184497 | TMEM255B | 3.796246387 | 5.13E-08 |
| ENSG00000232804 |  | 3.796667124 | 2.96E-31 |
| ENSG00000199053 | MIR324 | 3.799135191 | 0.000130178 |
| ENSG00000185607 | ACTBP7 | 3.80076583 | 0.005355425 |
| ENSG00000245904 | AC025164.1 | 3.804751076 | 0.036901746 |
| ENSG00000237686 | AL109615.3 | 3.806738442 | 8.17E-16 |
| ENSG00000229261 | AL596223.1 | 3.809687939 | 0.000354882 |
| ENSG00000250917 | AL035458.2 | 3.811366388 | 0.036077342 |
| ENSG00000219529 | AP000580.1 | 3.814104354 | 0.000223717 |
| ENSG00000081041 | CXCL2 | 3.822140928 | 3.64E-42 |
| ENSG00000120211 | INSL4 | 3.82228661 | 0.000263038 |
| ENSG00000223374 | AC005104.1 | 3.833560856 | 0.006036047 |
| ENSG00000140403 | DNAJA4 | 3.83739857 | 2.15E-108 |
| ENSG00000166592 | RRAD | 3.840297472 | 0.0000854 |
| ENSG00000257097 | CLIP1-AS1 | 3.840315055 | 0.038326217 |
| ENSG00000117318 | ID3 | 3.853491237 | 0.00000805 |
| ENSG00000275149 |  | 3.859388087 | 0.040906823 |
| ENSG00000127588 | GNG13 | 3.872657651 | 0.034060083 |
| ENSG00000130222 | GADD45G | 3.877103147 | 1.88E-21 |
| ENSG00000227782 | AC002553.1 | 3.884027422 | 0.000790018 |
| ENSG00000004799 | PDK4 | 3.896860857 | 3.36E-250 |
| ENSG00000205189 | ZBTB10 | 3.898556814 | 1.37E-152 |
| ENSG00000262652 | AC124283.3 | 3.909986867 | 0.011681285 |
| ENSG00000140678 | ITGAX | 3.921353824 | 0.002452542 |
| ENSG00000279691 | AC113410.3 | 3.93151467 | 0.000677494 |
| ENSG00000244151 | AC010973.2 | 3.933047217 | 6.88E-11 |
| ENSG00000129757 | CDKN1C | 3.934898783 | 9.37E-20 |
| ENSG00000115602 | IL1RL1 | 3.9357561 | 0.002121949 |
| ENSG00000182459 | TEX19 | 3.942910849 | 2.11E-18 |
| ENSG00000197406 | DIO3 | 3.956097713 | 0.00000115 |
| ENSG00000128482 | RNF112 | 3.958480643 | 0.005124604 |
| ENSG00000182308 | DCAF4L1 | 3.973763456 | 5.96E-10 |
| ENSG00000204103 | MAFB | 3.978934319 | 1.81E-34 |
| ENSG00000120694 | HSPH1 | 3.980321221 | 8.02E-130 |
| ENSG00000207041 | RNU6-3P | 3.985442473 | 0.027879643 |
| ENSG00000250508 | AP000808.1 | 3.987880615 | 0.0000791 |
| ENSG00000187094 | CCK | 3.993278384 | 0.00000694 |
| ENSG00000172602 | RND1 | 3.998761148 | 6.57E-80 |
| ENSG00000244219 | TMEM225B | 4.002533379 | 0.04840016 |
| ENSG00000140853 | NLRC5 | 4.0057005 | 6.75E-28 |
| ENSG00000278309 |  | 4.009522574 | 1.51E-14 |
| ENSG00000178381 | ZFAND2A | 4.010904914 | 9.83E-145 |
| ENSG00000106366 | SERPINE1 | 4.021817988 | 4.62E-14 |
| ENSG00000279897 | BIRC6-AS2 | 4.023148451 | 0.031828488 |
| ENSG00000273443 | AL645608.9 | 4.023527394 | 0.000615842 |
| ENSG00000237232 | ZNF295-AS1 | 4.038735613 | 0.021529555 |
| ENSG00000279765 | AC013394.1 | 4.039301222 | 0.00000507 |
| ENSG00000255964 | HSPD1P12 | 4.041984074 | 0.013081191 |
| ENSG00000124216 | SNAI1 | 4.042698652 | 8.87E-54 |
| ENSG00000186480 | INSIG1 | 4.046174902 | 1.05E-19 |
| ENSG00000167994 | RAB3IL1 | 4.062593983 | 1.5E-19 |
| ENSG00000127561 | SYNGR3 | 4.068196738 | 2.22E-37 |
| ENSG00000235481 | UBE2R2-AS1 | 4.068473296 | 0.033880324 |
| ENSG00000078081 | LAMP3 | 4.071919087 | 8.76E-12 |
| ENSG00000266446 | AL449423.1 | 4.07301414 | 0.000865618 |
| ENSG00000207923 | MIR559 | 4.086874458 | 0.022699274 |
| ENSG00000144824 | PHLDB2 | 4.090599611 | 7.06E-29 |
| ENSG00000279584 | AC005593.1 | 4.090715794 | 0.035281482 |
| ENSG00000052802 | MSMO1 | 4.094776109 | 1.44E-14 |
| ENSG00000259238 | AC092755.2 | 4.113984646 | 0.00000243 |
| ENSG00000276002 | RF00017 | 4.119963273 | 0.049389479 |
| ENSG00000226738 | U62317.1 | 4.138854359 | 0.048428234 |
| ENSG00000271153 | RPL23AP88 | 4.143833482 | 0.007764793 |
| ENSG00000137965 | IFI44 | 4.146134951 | 1.05E-14 |
| ENSG00000103313 | MEFV | 4.147209259 | 0.01143318 |
| ENSG00000119138 | KLF9 | 4.148392886 | 7.81E-74 |
| ENSG00000115844 | DLX2 | 4.152291471 | 8.32E-11 |
| ENSG00000280300 | AC117503.4 | 4.157710027 | 0.026641766 |
| ENSG00000275092 | AL031710.2 | 4.176311458 | 4.7E-09 |
| ENSG00000259657 | PIGHP1 | 4.183424568 | 0.00000516 |
| ENSG00000177688 | SUMO4 | 4.187579816 | 5.82E-08 |
| ENSG00000073737 | DHRS9 | 4.18923669 | 8.17E-61 |
| ENSG00000229677 | AC018644.1 | 4.190433752 | 0.043237226 |
| ENSG00000236658 | AL161733.1 | 4.190955815 | 0.033172982 |
| ENSG00000262202 | AC007952.4 | 4.197978249 | 0.019966234 |
| ENSG00000261604 | AC114947.2 | 4.198014166 | 0.0000119 |
| ENSG00000178934 | LGALS7B | 4.201762595 | 8.85E-27 |
| ENSG00000275126 | HIST1H4L | 4.203976801 | 0.032442128 |
| ENSG00000159231 | CBR3 | 4.206988388 | 0.000224397 |
| ENSG00000267056 | AC005336.1 | 4.217841744 | 0.032906656 |
| ENSG00000163499 | CRYBA2 | 4.232539379 | 0.024565412 |
| ENSG00000105642 | KCNN1 | 4.237715653 | 0.010248549 |
| ENSG00000275379 | HIST1H3I | 4.245847378 | 0.005542859 |
| ENSG00000167281 | RBFOX3 | 4.250613873 | 0.003486782 |
| ENSG00000253264 |  | 4.254560744 | 0.0000256 |
| ENSG00000160318 | CLDND2 | 4.264848371 | 1.57E-40 |
| ENSG00000174226 | SNX31 | 4.26693608 | 0.037361797 |
| ENSG00000122224 | LY9 | 4.266965404 | 0.005359331 |
| ENSG00000111335 | OAS2 | 4.270915432 | 2.26E-08 |
| ENSG00000215895 | AL354702.1 | 4.272184726 | 4.97E-09 |
| ENSG00000166793 | YPEL4 | 4.272291857 | 0.037860399 |
| ENSG00000125775 | SDCBP2 | 4.274845977 | 3.73E-202 |
| ENSG00000185565 | LSAMP | 4.276191363 | 0.012578322 |
| ENSG00000118495 | PLAGL1 | 4.281276657 | 0.000628506 |
| ENSG00000265962 | GACAT2 | 4.289762457 | 0.017032525 |
| ENSG00000167754 | KLK5 | 4.292514312 | 0.004050139 |
| ENSG00000187608 | ISG15 | 4.299825282 | 1.19E-99 |
| ENSG00000137745 | MMP13 | 4.303236213 | 2.12E-09 |
| ENSG00000160712 | IL6R | 4.306945327 | 1.31E-105 |
| ENSG00000227471 | AKR1B15 | 4.327394016 | 0.002001452 |
| ENSG00000241749 | RPSAP52 | 4.328287008 | 6.26E-09 |
| ENSG00000228653 | HNRNPCP7 | 4.336967221 | 0.000123616 |
| ENSG00000275441 | AC020765.2 | 4.337228712 | 0.000115741 |
| ENSG00000278463 | HIST1H2AB | 4.355583297 | 0.028088069 |
| ENSG00000026751 | SLAMF7 | 4.364896588 | 0.049561822 |
| ENSG00000279762 | AC005899.8 | 4.368180055 | 0.021966993 |
| ENSG00000268366 | AC010271.1 | 4.383508517 | 0.010748058 |
| ENSG00000108387 | 44443 | 4.39070406 | 0.0000287 |
| ENSG00000203721 | LINC00862 | 4.395703038 | 0.025744594 |
| ENSG00000278705 | HIST1H4B | 4.398873266 | 0.016850687 |
| ENSG00000260284 | TPSP2 | 4.400910137 | 0.015056154 |
| ENSG00000110324 | IL10RA | 4.402665432 | 0.00023176 |
| ENSG00000123612 | ACVR1C | 4.416204204 | 2.64E-08 |
| ENSG00000169429 | CXCL8 | 4.41792952 | 2.25E-96 |
| ENSG00000137628 | DDX60 | 4.426778526 | 9.25E-10 |
| ENSG00000163209 | SPRR3 | 4.428461345 | 0.005704362 |
| ENSG00000225760 | LINC00431 | 4.445476216 | 6.27E-18 |
| ENSG00000172183 | ISG20 | 4.448970065 | 3.18E-86 |
| ENSG00000197279 | ZNF165 | 4.457176805 | 3.57E-31 |
| ENSG00000179148 | ALOXE3 | 4.461097218 | 9.19E-12 |
| ENSG00000269729 | AC006262.2 | 4.461729018 | 0.008283466 |
| ENSG00000241217 | RN7SL809P | 4.461988986 | 0.028891931 |
| ENSG00000272009 | AL121944.1 | 4.475658743 | 1.72E-57 |
| ENSG00000119917 | IFIT3 | 4.481320969 | 4.51E-87 |
| ENSG00000228446 | AC073052.1 | 4.48833787 | 0.0370737 |
| ENSG00000205076 | LGALS7 | 4.493694973 | 6.59E-16 |
| ENSG00000249947 | XBP1P1 | 4.495199162 | 0.013822813 |
| ENSG00000115267 | IFIH1 | 4.504822391 | 3.91E-196 |
| ENSG00000214915 | BX546450.1 | 4.505533989 | 0.009935658 |
| ENSG00000134107 | BHLHE40 | 4.506054864 | 3.29E-184 |
| ENSG00000099860 | GADD45B | 4.51612361 | 4.67E-103 |
| ENSG00000183508 | FAM46C | 4.516498905 | 2.01E-13 |
| ENSG00000198535 | C2CD4A | 4.54224708 | 0.000805605 |
| ENSG00000128422 | KRT17 | 4.547866064 | 3.35E-41 |
| ENSG00000204520 | MICA | 4.549116404 | 0.000131761 |
| ENSG00000270948 | MTDHP1 | 4.550431584 | 0.036727831 |
| ENSG00000184414 | IRS3P | 4.556376475 | 0.00517059 |
| ENSG00000213212 | NCLP1 | 4.559640753 | 0.000396078 |
| ENSG00000236060 | HSPB1P1 | 4.560653113 | 0.001707073 |
| ENSG00000164236 | ANKRD33B | 4.563278392 | 0.046496097 |
| ENSG00000277011 | AC148477.4 | 4.565544511 | 0.028374138 |
| ENSG00000176170 | SPHK1 | 4.573154609 | 5.83E-94 |
| ENSG00000130643 | CALY | 4.576448265 | 0.023203961 |
| ENSG00000124253 | PCK1 | 4.58259934 | 0.000116229 |
| ENSG00000228964 |  | 4.588309703 | 0.028046127 |
| ENSG00000257595 | LINC02356 | 4.599246124 | 0.00554937 |
| ENSG00000234584 | AC019186.1 | 4.599846917 | 0.00000251 |
| ENSG00000137193 | PIM1 | 4.616330656 | 3.63E-159 |
| ENSG00000006283 | CACNA1G | 4.61952461 | 2.22E-15 |
| ENSG00000120885 | CLU | 4.624335803 | 1.37E-188 |
| ENSG00000255533 | AC136475.6 | 4.626888441 | 0.005144274 |
| ENSG00000012124 | CD22 | 4.627897791 | 0.000000168 |
| ENSG00000279164 | AL118508.3 | 4.643402769 | 0.000395058 |
| ENSG00000259475 | AC036108.1 | 4.645697956 | 0.034048645 |
| ENSG00000167749 | KLK4 | 4.646322107 | 0.009263341 |
| ENSG00000227676 | LINC01068 | 4.64853757 | 0.00271821 |
| ENSG00000250116 | AC018682.1 | 4.653044284 | 0.020364804 |
| ENSG00000278330 | AC018529.2 | 4.661301424 | 0.003114407 |
| ENSG00000258469 | CHMP4BP1 | 4.663563465 | 0.00023488 |
| ENSG00000146453 | PNLDC1 | 4.675688232 | 0.001016194 |
| ENSG00000137825 | ITPKA | 4.67702673 | 3.92E-109 |
| ENSG00000251537 | AC005324.3 | 4.691035686 | 0.018336525 |
| ENSG00000273707 |  | 4.692508186 | 0.013198813 |
| ENSG00000085662 | AKR1B1 | 4.693750062 | 1.56E-17 |
| ENSG00000173261 | PLAC8L1 | 4.693877234 | 0.020811391 |
| ENSG00000130487 | KLHDC7B | 4.694035559 | 0.0091071 |
| ENSG00000112599 | GUCA1B | 4.697384449 | 0.000000325 |
| ENSG00000203722 | RAET1G | 4.719433643 | 5.57E-12 |
| ENSG00000236337 | FMR1-IT1 | 4.729926669 | 0.005956493 |
| ENSG00000224608 |  | 4.744402991 | 2.94E-24 |
| ENSG00000136514 | RTP4 | 4.746034469 | 0.001448684 |
| ENSG00000282355 |  | 4.757492033 | 0.021254453 |
| ENSG00000273703 | HIST1H2BM | 4.773842699 | 0.000924733 |
| ENSG00000105784 | RUNDC3B | 4.775497229 | 0.008533685 |
| ENSG00000280039 | RN7SKP23 | 4.790360141 | 0.031363093 |
| ENSG00000264229 | RNU4ATAC | 4.794015483 | 0.029120455 |
| ENSG00000169876 | MUC17 | 4.800815299 | 0.0000014 |
| ENSG00000166833 | NAV2 | 4.807217874 | 1.38E-15 |
| ENSG00000226598 |  | 4.808834227 | 0.021980275 |
| ENSG00000260186 | LINC02137 | 4.841252004 | 0.009222503 |
| ENSG00000125675 | GRIA3 | 4.84503006 | 0.000472618 |
| ENSG00000152137 | HSPB8 | 4.856619071 | 0.000213108 |
| ENSG00000137403 |  | 4.857915716 | 0.012696032 |
| ENSG00000138311 | ZNF365 | 4.864414008 | 0.043961314 |
| ENSG00000104951 | IL4I1 | 4.867197089 | 7.2E-33 |
| ENSG00000279766 | AC067931.1 | 4.868819053 | 1.32E-56 |
| ENSG00000122641 | INHBA | 4.871399312 | 0.009026766 |
| ENSG00000122861 | PLAU | 4.873266673 | 6.27E-25 |
| ENSG00000248394 | FOSL1P1 | 4.898205227 | 0.049794462 |
| ENSG00000224715 | Z82186.1 | 4.905904496 | 0.02880414 |
| ENSG00000274210 | RF00003 | 4.917652691 | 0.000477093 |
| ENSG00000237643 | AL365226.2 | 4.91986704 | 0.032987218 |
| ENSG00000261407 | AC013565.3 | 4.92096602 | 0.006965161 |
| ENSG00000215861 | AC245297.1 | 4.926375199 | 0.000233833 |
| ENSG00000186832 | KRT16 | 4.934754229 | 0.005929479 |
| ENSG00000227300 | KRT16P2 | 4.936540538 | 0.015697074 |
| ENSG00000229512 | AC068580.1 | 4.937446669 | 2.48E-29 |
| ENSG00000274322 | AL136531.2 | 4.941047477 | 0.000356789 |
| ENSG00000184270 | HIST2H2AB | 4.946431615 | 0.008342274 |
| ENSG00000169474 | SPRR1A | 4.948410996 | 9.9E-09 |
| ENSG00000148175 | STOM | 4.958719155 | 0.0000903 |
| ENSG00000079393 | DUSP13 | 4.967030749 | 0.0000478 |
| ENSG00000275538 | RNVU1-19 | 4.972750355 | 0.000808366 |
| ENSG00000119508 | NR4A3 | 4.97840304 | 0.00000329 |
| ENSG00000152377 | SPOCK1 | 4.987064941 | 0.030053309 |
| ENSG00000266304 | LIVAR | 5.011317872 | 0.0000616 |
| ENSG00000274591 | AC025031.3 | 5.034042918 | 0.034566193 |
| ENSG00000206450 |  | 5.043002229 | 1.67E-22 |
| ENSG00000198286 | CARD11 | 5.04857356 | 0.014240358 |
| ENSG00000261411 | AL161938.1 | 5.059879221 | 0.000637279 |
| ENSG00000173237 | C11orf86 | 5.076043981 | 8.67E-10 |
| ENSG00000225420 | AC104134.1 | 5.096521453 | 0.026183988 |
| ENSG00000143603 | KCNN3 | 5.099142312 | 0.026205556 |
| ENSG00000277290 | AC136475.10 | 5.130773395 | 0.000417962 |
| ENSG00000159495 | TGM7 | 5.166083712 | 0.044613475 |
| ENSG00000144130 | NT5DC4 | 5.176556892 | 0.0000396 |
| ENSG00000267592 | AC004134.1 | 5.179627936 | 0.000120322 |
| ENSG00000197588 | KLKP1 | 5.19015393 | 0.00053645 |
| ENSG00000249885 | ARHGEF38-IT1 | 5.198073959 | 0.000148496 |
| ENSG00000196611 | MMP1 | 5.199484956 | 4.47E-08 |
| ENSG00000231290 | APCDD1L-AS1 | 5.202499273 | 6.54E-69 |
| ENSG00000189410 | SH2D5 | 5.215358987 | 4.74E-18 |
| ENSG00000230387 | AL118508.1 | 5.221132433 | 0.006217723 |
| ENSG00000186831 | KRT17P2 | 5.232207057 | 0.00067382 |
| ENSG00000048052 | HDAC9 | 5.239811912 | 6.18E-62 |
| ENSG00000264301 | LINC01444 | 5.253690711 | 0.026922311 |
| ENSG00000184557 | SOCS3 | 5.260685194 | 2.51E-40 |
| ENSG00000235027 | AC068580.3 | 5.267074823 | 2.47E-44 |
| ENSG00000278493 | AC039056.2 | 5.310420194 | 9.58E-25 |
| ENSG00000232331 |  | 5.311886375 | 0.022724721 |
| ENSG00000224431 | AC063976.2 | 5.312833856 | 0.001667484 |
| ENSG00000238266 | LINC00707 | 5.313554187 | 0.004938933 |
| ENSG00000274213 | AC015912.3 | 5.323036465 | 2.46E-87 |
| ENSG00000243649 | CFB | 5.32327716 | 0.005954944 |
| ENSG00000231969 | AC007364.1 | 5.336213121 | 0.000000893 |
| ENSG00000182013 | PNMA8A | 5.339693071 | 0.001589621 |
| ENSG00000145777 | TSLP | 5.351390755 | 0.019854384 |
| ENSG00000259712 | AC023906.5 | 5.360190669 | 0.002598301 |
| ENSG00000249379 | AL033397.2 | 5.362417822 | 0.000903793 |
| ENSG00000152689 | RASGRP3 | 5.367404489 | 0.000000256 |
| ENSG00000217825 | AC099552.1 | 5.369478539 | 0.002345987 |
| ENSG00000256128 | LINC00944 | 5.371059472 | 0.00000269 |
| ENSG00000116183 | PAPPA2 | 5.397887113 | 0.000116071 |
| ENSG00000151632 | AKR1C2 | 5.405532817 | 6.25E-31 |
| ENSG00000255248 | MIR100HG | 5.419683797 | 0.025399419 |
| ENSG00000237417 | XRCC6P1 | 5.422076499 | 0.0000393 |
| ENSG00000118407 | FILIP1 | 5.437811578 | 0.00000112 |
| ENSG00000237596 | AL138828.1 | 5.450999218 | 0.0000626 |
| ENSG00000119922 | IFIT2 | 5.452582041 | 1.19E-39 |
| ENSG00000148926 | ADM | 5.466496757 | 0 |
| ENSG00000196358 | NTNG2 | 5.492318724 | 0.000688879 |
| ENSG00000164363 | SLC6A18 | 5.508612426 | 0.006098026 |
| ENSG00000259211 | AC013356.2 | 5.512607806 | 0.022560106 |
| ENSG00000105499 | PLA2G4C | 5.527284523 | 7.4E-11 |
| ENSG00000168685 | IL7R | 5.537501364 | 0.001212474 |
| ENSG00000167476 | JSRP1 | 5.54714655 | 5.4E-11 |
| ENSG00000200879 | SNORD14E | 5.584350198 | 0.0000125 |
| ENSG00000226145 | KRT16P6 | 5.613774136 | 0.005908719 |
| ENSG00000248323 | LUCAT1 | 5.621458832 | 7.58E-11 |
| ENSG00000170965 | PLAC1 | 5.62541794 | 0.00000277 |
| ENSG00000260386 | LINC01225 | 5.63710746 | 0.001057935 |
| ENSG00000249884 | RNF103-CHMP3 | 5.651929798 | 4.02E-15 |
| ENSG00000235899 | LINC01564 | 5.659649512 | 2.86E-28 |
| ENSG00000163273 | NPPC | 5.673520269 | 0.00257113 |
| ENSG00000006210 | CX3CL1 | 5.674616639 | 2.06E-40 |
| ENSG00000240476 | LINC00973 | 5.677649876 | 7.73E-125 |
| ENSG00000188536 | HBA2 | 5.700065548 | 0.007714009 |
| ENSG00000269560 | AC010422.4 | 5.720840919 | 0.010080844 |
| ENSG00000259005 | AC005479.1 | 5.737303998 | 0.00000427 |
| ENSG00000134802 | SLC43A3 | 5.737342143 | 0.0000371 |
| ENSG00000184371 | CSF1 | 5.760237488 | 1.88E-27 |
| ENSG00000104804 | TULP2 | 5.766519258 | 0.00015874 |
| ENSG00000111452 | ADGRD1 | 5.766745485 | 0.000517544 |
| ENSG00000167772 | ANGPTL4 | 5.769373804 | 5.36E-36 |
| ENSG00000272666 | U62317.2 | 5.794668423 | 0.000481522 |
| ENSG00000170925 | TEX13B | 5.795983111 | 0.000198694 |
| ENSG00000276305 |  | 5.873182217 | 0.001975721 |
| ENSG00000185745 | IFIT1 | 5.883534568 | 3.77E-52 |
| ENSG00000187134 | AKR1C1 | 5.896553445 | 7.57E-72 |
| ENSG00000188100 | FAM25A | 5.947126002 | 0.000347655 |
| ENSG00000060566 | CREB3L3 | 5.955589129 | 0.001114471 |
| ENSG00000198768 | APCDD1L | 5.956075325 | 0.0000082 |
| ENSG00000155918 | RAET1L | 5.978272667 | 2.42E-192 |
| ENSG00000277892 | MIR6746 | 6.005356803 | 0.003665115 |
| ENSG00000270000 | AC005479.2 | 6.015515332 | 0.0000139 |
| ENSG00000134571 | MYBPC3 | 6.023555273 | 0.0000938 |
| ENSG00000214803 | AC090921.1 | 6.048638014 | 0.000102559 |
| ENSG00000135114 | OASL | 6.052520499 | 5.34E-43 |
| ENSG00000067842 | ATP2B3 | 6.086335688 | 0.0000051 |
| ENSG00000164949 | GEM | 6.144680708 | 1.24E-191 |
| ENSG00000138829 | FBN2 | 6.21144641 | 0.000000238 |
| ENSG00000170801 | HTRA3 | 6.366653404 | 0.000000113 |
| ENSG00000138623 | SEMA7A | 6.372572525 | 2.16E-208 |
| ENSG00000135604 | STX11 | 6.489926301 | 0.00000103 |
| ENSG00000258791 | LINC00520 | 6.524960637 | 0.0000133 |
| ENSG00000023445 | BIRC3 | 6.533321845 | 0 |
| ENSG00000171855 | IFNB1 | 6.534213427 | 0.00000157 |
| ENSG00000155962 | CLIC2 | 6.575289506 | 1.44E-38 |
| ENSG00000160963 | COL26A1 | 6.579581125 | 0.00000875 |
| ENSG00000136999 | NOV | 6.632757445 | 3.5E-10 |
| ENSG00000260314 | MRC1 | 6.641913769 | 0.000000781 |
| ENSG00000166670 | MMP10 | 6.65228469 | 2.29E-09 |
| ENSG00000237512 | UNC5B-AS1 | 6.665819764 | 0.000000365 |
| ENSG00000227363 |  | 6.827371823 | 0.006884008 |
| ENSG00000279602 | AC109326.1 | 6.838127095 | 3.53E-18 |
| ENSG00000224251 | AL391427.1 | 6.843179818 | 0.000000151 |
| ENSG00000138646 | HERC5 | 6.84846117 | 2.04E-26 |
| ENSG00000135406 | PRPH | 6.872741014 | 0.000000944 |
| ENSG00000163121 | NEURL3 | 7.209718007 | 1.1E-100 |
| ENSG00000228203 | RNF144A-AS1 | 7.242584696 | 0.000000202 |
| ENSG00000100292 | HMOX1 | 7.3795621 | 1.03E-61 |
| ENSG00000257732 | AC089983.1 | 7.415052776 | 1.41E-10 |
| ENSG00000144834 | TAGLN3 | 7.458489966 | 9.12E-09 |
| ENSG00000118503 | TNFAIP3 | 7.487334877 | 7.49E-197 |
| ENSG00000183709 | IFNL2 | 7.683982793 | 3.32E-10 |
| ENSG00000128591 | FLNC | 7.977360428 | 3.21E-26 |
| ENSG00000008517 | IL32 | 8.062502663 | 2.44E-77 |
| ENSG00000197110 | IFNL3 | 8.134843495 | 5.98E-11 |
| ENSG00000166831 | RBPMS2 | 8.310658489 | 4.63E-57 |
| ENSG00000049249 | TNFRSF9 | 8.621881521 | 2.42E-11 |
| ENSG00000175183 | CSRP2 | 8.743014687 | 6.52E-33 |
| ENSG00000087085 | ACHE | 8.751936764 | 3.85E-12 |
| ENSG00000206013 | IFITM5 | 9.059175183 | 1.93E-13 |
| ENSG00000134321 | RSAD2 | 9.128644894 | 9.03E-22 |
| ENSG00000268223 | ARL14EPL | 9.247665563 | 0.000284575 |
| ENSG00000164400 | CSF2 | 9.442581778 | 2.75E-23 |
| ENSG00000269826 | AC092327.2 | 9.555255357 | 1.42E-14 |
| ENSG00000102962 | CCL22 | 9.800759133 | 2.1E-13 |
| ENSG00000169245 | CXCL10 | 9.843335193 | 3.75E-24 |
| ENSG00000182393 | IFNL1 | 9.965437744 | 2.68E-43 |
| ENSG00000169248 | CXCL11 | 10.19088406 | 1.22E-19 |
| ENSG00000225217 | HSPA7 | 11.90813691 | 1.29E-27 |
| ENSG00000173110 | HSPA6 | 12.01136379 | 1.97E-87 |
| ENSG00000109846 | CRYAB | 14.49105846 | 2.9E-10 |
| ENSG00000181819 | KCTD9P2 | 17.75633517 | 0.000000105 |
| ENSG00000154451 | GBP5 | 27.5571623 | 1.76E-09 |

**List of down regulated genes (p value＜0.05)**

| Gene ID | Gene symbol | log2FoldChange | padj |
| --- | --- | --- | --- |
| ENSG00000237788 | AL162615.1 | -29.71577588 | 3.83E-38 |
| ENSG00000243514 | RPL32P33 | -28.30377763 | 5.93E-24 |
| ENSG00000175329 | ISX | -6.325218637 | 0.0000237 |
| ENSG00000233124 | LINC00456 | -6.223091084 | 0.000200337 |
| ENSG00000212939 | Z97192.1 | -6.143964681 | 0.0000548 |
| ENSG00000114251 | WNT5A | -6.041474995 | 0.0000391 |
| ENSG00000265107 | GJA5 | -6.007912146 | 0.00000268 |
| ENSG00000261324 | AC010168.2 | -5.898327189 | 0.0000424 |
| ENSG00000273783 | AL136040.1 | -5.870922183 | 0.000147272 |
| ENSG00000257108 | NHLRC4 | -5.827620475 | 0.000142913 |
| ENSG00000148204 | CRB2 | -5.767239023 | 1.5E-09 |
| ENSG00000183309 | ZNF623 | -5.70785369 | 0.000143303 |
| ENSG00000259319 | AF111167.2 | -5.671482059 | 0.000165187 |
| ENSG00000233817 | AL162727.1 | -5.666550394 | 0.003155719 |
| ENSG00000248144 | ADH1C | -5.641443979 | 0.000037 |
| ENSG00000277639 | AC007906.2 | -5.594579115 | 0.000142898 |
| ENSG00000253616 | AC107959.3 | -5.592227808 | 0.000110473 |
| ENSG00000214553 | LRRC37A11P | -5.585468633 | 0.0000678 |
| ENSG00000279806 | AC018629.1 | -5.500212171 | 4.72E-24 |
| ENSG00000258701 | LINC00638 | -5.453654777 | 0.00000266 |
| ENSG00000278385 | AC121338.2 | -5.449077851 | 0.0000518 |
| ENSG00000187624 | C17orf97 | -5.372532044 | 0.000369767 |
| ENSG00000119283 | TRIM67 | -5.342004479 | 0.007833942 |
| ENSG00000260841 |  | -5.316343807 | 0.000532125 |
| ENSG00000254799 | SLC25A47P1 | -5.31599335 | 0.00012358 |
| ENSG00000186017 | ZNF566 | -5.311576819 | 0.0000432 |
| ENSG00000254396 | AL355432.1 | -5.298223284 | 0.002386051 |
| ENSG00000227582 | ADGRF5P1 | -5.275844922 | 0.000197764 |
| ENSG00000263893 | AC080037.1 | -5.256436367 | 0.000408563 |
| ENSG00000251152 | AC025539.1 | -5.22494879 | 0.005294433 |
| ENSG00000214797 | AP002358.1 | -5.201913886 | 0.0001227 |
| ENSG00000124334 | IL9R | -5.20046673 | 0.000788217 |
| ENSG00000261737 | AL049597.2 | -5.196604755 | 0.000623943 |
| ENSG00000260874 | AC106820.5 | -5.194310446 | 0.001366729 |
| ENSG00000203857 | HSD3B1 | -5.171439769 | 0.000126409 |
| ENSG00000270175 | AC023509.4 | -5.167705065 | 0.000411348 |
| ENSG00000205488 | CALML3-AS1 | -5.13557548 | 0.004222613 |
| ENSG00000237807 | AC022034.2 | -5.103071283 | 0.001311248 |
| ENSG00000260274 | AC068338.2 | -5.079715165 | 0.000018 |
| ENSG00000229388 | LINC01715 | -5.074646416 | 0.00325577 |
| ENSG00000187013 | C17orf82 | -5.035538007 | 0.0000562 |
| ENSG00000226200 | SGMS1-AS1 | -5.033983731 | 0.0000026 |
| ENSG00000183153 | GJD3 | -5.017307075 | 0.00410512 |
| ENSG00000244515 | KRT18P34 | -5.002923316 | 0.010823574 |
| ENSG00000260105 | AOC4P | -4.989454998 | 6.55E-09 |
| ENSG00000213754 | AL356317.1 | -4.959663481 | 0.00535173 |
| ENSG00000182791 | CCDC87 | -4.953001512 | 0.000140672 |
| ENSG00000223343 | AC137630.1 | -4.94016907 | 0.004910692 |
| ENSG00000173404 | INSM1 | -4.901634952 | 0.002369652 |
| ENSG00000261229 | AC021483.2 | -4.896703646 | 0.004438217 |
| ENSG00000235387 | SPAAR | -4.892507481 | 0.000824939 |
| ENSG00000260572 | AC069224.1 | -4.882631282 | 0.015307909 |
| ENSG00000182612 | TSPAN10 | -4.85281704 | 0.000344511 |
| ENSG00000133488 | SEC14L4 | -4.805828458 | 0.011784688 |
| ENSG00000197046 | SIGLEC15 | -4.802595466 | 3.08E-10 |
| ENSG00000243836 | WDR86-AS1 | -4.79864303 | 0.01448862 |
| ENSG00000225439 | BOLA3-AS1 | -4.796778232 | 0.004529219 |
| ENSG00000280217 | AC126564.1 | -4.758829894 | 0.002645072 |
| ENSG00000184730 | APOBR | -4.758777957 | 0.000561024 |
| ENSG00000216915 | GPR89P | -4.745052494 | 0.018733316 |
| ENSG00000278995 | Z69720.2 | -4.738229999 | 0.041362863 |
| ENSG00000104524 | PYCR3 | -4.733287843 | 0.028387416 |
| ENSG00000169515 | CCDC8 | -4.731456725 | 0.004161747 |
| ENSG00000272902 | TBC1D8-AS1 | -4.710349266 | 0.000238559 |
| ENSG00000261754 | AC008555.1 | -4.70672041 | 0.00680115 |
| ENSG00000140835 | CHST4 | -4.703330283 | 1.89E-08 |
| ENSG00000282331 |  | -4.69726896 | 0.000179521 |
| ENSG00000245552 | AP000787.1 | -4.695762177 | 0.009984726 |
| ENSG00000205086 | C2orf91 | -4.648241008 | 0.014758956 |
| ENSG00000180347 | CCDC129 | -4.644177247 | 0.013136393 |
| ENSG00000236975 | AL137793.1 | -4.621958137 | 0.041681698 |
| ENSG00000230092 | AL669831.4 | -4.616176631 | 0.006229511 |
| ENSG00000120156 | TEK | -4.607522292 | 2.46E-17 |
| ENSG00000270179 | AP002840.2 | -4.605498063 | 0.00337792 |
| ENSG00000230445 | LRRC37A6P | -4.592777075 | 0.004005266 |
| ENSG00000262209 | PCDHGB3 | -4.591677214 | 0.01520023 |
| ENSG00000185008 | ROBO2 | -4.578954214 | 0.01591677 |
| ENSG00000247796 | AC008966.1 | -4.547972907 | 4.8E-11 |
| ENSG00000187185 | AC092118.1 | -4.544766589 | 0.025645715 |
| ENSG00000228906 | AL353804.1 | -4.543132367 | 0.005754108 |
| ENSG00000260989 | AL133297.2 | -4.509017838 | 0.049539202 |
| ENSG00000237685 | AL139039.3 | -4.507686167 | 0.007522511 |
| ENSG00000275964 | AL355001.2 | -4.502559108 | 3.43E-13 |
| ENSG00000233396 | LINC01719 | -4.498106266 | 0.007114441 |
| ENSG00000269837 | IPO5P1 | -4.496113089 | 0.003976955 |
| ENSG00000260063 | AL512408.1 | -4.483382363 | 0.005018913 |
| ENSG00000228172 | AL020996.1 | -4.461893128 | 0.015698735 |
| ENSG00000261685 |  | -4.450004787 | 0.007278647 |
| ENSG00000146216 | TTBK1 | -4.445120323 | 0.00000303 |
| ENSG00000260328 | AC104024.2 | -4.441526527 | 5.01E-09 |
| ENSG00000253731 | PCDHGA6 | -4.441489885 | 0.001658902 |
| ENSG00000260339 | HEXA-AS1 | -4.406603212 | 0.012187861 |
| ENSG00000168004 | HRASLS5 | -4.403128464 | 0.006754105 |
| ENSG00000119699 | TGFB3 | -4.394081848 | 0.00000224 |
| ENSG00000225302 |  | -4.386167027 | 8.46E-11 |
| ENSG00000255568 | BRWD1-AS2 | -4.374189477 | 0.014964395 |
| ENSG00000139292 | LGR5 | -4.371573019 | 4.07E-28 |
| ENSG00000175065 | DSG4 | -4.368708156 | 0.008108328 |
| ENSG00000189129 | PLAC9 | -4.36634488 | 0.01189225 |
| ENSG00000230359 | TPI1P2 | -4.358603326 | 0.005542859 |
| ENSG00000260193 | AL138781.1 | -4.336353069 | 0.010704866 |
| ENSG00000159761 | C16orf86 | -4.329875057 | 0.003566219 |
| ENSG00000275106 | AC025594.3 | -4.317149318 | 0.016348339 |
| ENSG00000091972 | CD200 | -4.289568157 | 7.84E-10 |
| ENSG00000174586 | ZNF497 | -4.282515869 | 0.000000153 |
| ENSG00000262837 | AC027801.5 | -4.282363166 | 0.030543657 |
| ENSG00000271857 | AL096865.1 | -4.279000756 | 0.003011405 |
| ENSG00000237126 | AC073254.1 | -4.268339437 | 0.00672922 |
| ENSG00000273387 | AC005005.3 | -4.262883726 | 0.014084269 |
| ENSG00000272384 | AC016405.3 | -4.258631916 | 0.022046069 |
| ENSG00000226754 | AL606760.1 | -4.257547551 | 0.005550994 |
| ENSG00000246465 | AC138904.1 | -4.256472328 | 0.00367805 |
| ENSG00000184564 | SLITRK6 | -4.249888471 | 5.26E-68 |
| ENSG00000254694 | AP001893.1 | -4.245985759 | 0.029311541 |
| ENSG00000254389 | RHPN1-AS1 | -4.239056516 | 0.000239415 |
| ENSG00000272444 | AL118558.4 | -4.231991993 | 0.048545263 |
| ENSG00000249574 | AC226118.1 | -4.226612097 | 0.045395544 |
| ENSG00000257191 | AC090709.1 | -4.218841919 | 0.015669594 |
| ENSG00000243885 | AC108751.4 | -4.214299483 | 0.0380879 |
| ENSG00000279741 | AC007342.8 | -4.214074555 | 0.003089063 |
| ENSG00000227188 | MGAT3-AS1 | -4.209510193 | 0.037778915 |
| ENSG00000280426 | AC084876.2 | -4.204487466 | 0.036273595 |
| ENSG00000184995 | IFNE | -4.199664506 | 0.000414446 |
| ENSG00000246022 | ALDH1L1-AS2 | -4.182241112 | 0.0110236 |
| ENSG00000164220 | F2RL2 | -4.182188686 | 0.033329218 |
| ENSG00000279368 | AL031123.3 | -4.173078258 | 0.001878504 |
| ENSG00000277475 | AC213203.1 | -4.169783675 | 0.016320324 |
| ENSG00000266680 | AL135905.1 | -4.168752232 | 0.000876123 |
| ENSG00000230438 | SERPINB9P1 | -4.167391773 | 0.005227807 |
| ENSG00000234286 | AC006026.3 | -4.161664063 | 0.001399215 |
| ENSG00000279024 | AC112255.1 | -4.160847773 | 0.041630354 |
| ENSG00000276728 | AC142472.1 | -4.15226685 | 0.0000757 |
| ENSG00000187554 | TLR5 | -4.151140796 | 0.000657588 |
| ENSG00000251023 | AC114980.1 | -4.141093803 | 0.002615346 |
| ENSG00000277013 | AC008556.1 | -4.139984799 | 0.024386415 |
| ENSG00000273249 | BX649632.1 | -4.136859641 | 0.049561822 |
| ENSG00000271020 | AC112220.2 | -4.1197367 | 0.00000158 |
| ENSG00000260273 | AL359711.2 | -4.119337076 | 0.03808635 |
| ENSG00000162711 | NLRP3 | -4.117529872 | 0.002564188 |
| ENSG00000189045 | ANKDD1B | -4.115340629 | 0.027877022 |
| ENSG00000277496 | AL357033.4 | -4.111534029 | 6.48E-16 |
| ENSG00000133980 | VRTN | -4.091592302 | 8.73E-18 |
| ENSG00000261584 | AL513548.1 | -4.072899819 | 0.011863517 |
| ENSG00000229155 | LINC02038 | -4.071299075 | 3.11E-12 |
| ENSG00000125384 | PTGER2 | -4.061823186 | 0.045802794 |
| ENSG00000279064 | FP236315.1 | -4.049223664 | 0.038252171 |
| ENSG00000117834 | SLC5A9 | -4.047296777 | 0.000239253 |
| ENSG00000267764 | AC093567.1 | -4.046029047 | 0.017922221 |
| ENSG00000181754 | AMIGO1 | -4.033601386 | 6.28E-12 |
| ENSG00000259985 | AC017100.1 | -4.033011656 | 0.001498585 |
| ENSG00000143847 | PPFIA4 | -4.020587529 | 0.011626627 |
| ENSG00000168497 | CAVIN2 | -4.016247121 | 0.00000108 |
| ENSG00000277159 | AL139384.2 | -4.011428418 | 0.047746346 |
| ENSG00000135835 | KIAA1614 | -3.998282133 | 0.010097288 |
| ENSG00000074966 | TXK | -3.988866532 | 4.67E-31 |
| ENSG00000261659 | Z92544.2 | -3.987193512 | 0.00000064 |
| ENSG00000153930 | ANKFN1 | -3.982614595 | 1.58E-08 |
| ENSG00000215493 | AC007731.2 | -3.9798695 | 0.019650506 |
| ENSG00000259205 | PRKXP1 | -3.979212275 | 0.01054457 |
| ENSG00000266962 | AC067852.2 | -3.976051433 | 0.00000154 |
| ENSG00000232859 | LYRM9 | -3.963365382 | 0.035445197 |
| ENSG00000275198 | AL512791.2 | -3.962864216 | 0.0416904 |
| ENSG00000188451 | SRP72P2 | -3.960279176 | 0.043579566 |
| ENSG00000187792 | ZNF70 | -3.952513712 | 1.12E-10 |
| ENSG00000150594 | ADRA2A | -3.94025382 | 4.04E-34 |
| ENSG00000240184 | PCDHGC3 | -3.940071425 | 0.00000634 |
| ENSG00000196659 | TTC30B | -3.936243119 | 5.92E-27 |
| ENSG00000182747 | SLC35D3 | -3.934657164 | 0.032017957 |
| ENSG00000213221 | DNLZ | -3.930631877 | 0.030413388 |
| ENSG00000235374 | SSR4P1 | -3.920792519 | 0.027159347 |
| ENSG00000274922 | AL139384.1 | -3.920235915 | 0.000395131 |
| ENSG00000158683 | PKD1L1 | -3.916893704 | 0.031487566 |
| ENSG00000100583 | SAMD15 | -3.915863722 | 0.003607372 |
| ENSG00000254876 | AL590705.5 | -3.90746459 | 0.0000778 |
| ENSG00000265203 | RBP3 | -3.885646863 | 0.041722875 |
| ENSG00000135625 | EGR4 | -3.87395421 | 0.0000029 |
| ENSG00000180785 | OR51E1 | -3.865084725 | 1.06E-11 |
| ENSG00000232160 | RAP2C-AS1 | -3.864055643 | 0.022211968 |
| ENSG00000261211 | AL031123.2 | -3.861390909 | 0.005500814 |
| ENSG00000118526 | TCF21 | -3.851582587 | 0.00000199 |
| ENSG00000257354 | AC048341.1 | -3.84476321 | 0.00000168 |
| ENSG00000254122 | PCDHGB7 | -3.844138419 | 0.011785594 |
| ENSG00000170927 | PKHD1 | -3.834259015 | 0.010126242 |
| ENSG00000229344 | MTCO2P12 | -3.831330322 | 0.001864875 |
| ENSG00000255561 | FDXACB1 | -3.831246443 | 0.000000044 |
| ENSG00000184809 | B3GALT5-AS1 | -3.824112349 | 0.00044134 |
| ENSG00000259498 | TPM1-AS | -3.798230692 | 0.022299349 |
| ENSG00000173698 | ADGRG2 | -3.796626988 | 0.010373497 |
| ENSG00000163520 | FBLN2 | -3.795904907 | 3.15E-08 |
| ENSG00000196169 | KIF19 | -3.794892443 | 0.019688263 |
| ENSG00000265688 | MAFG-AS1 | -3.793796496 | 5.11E-22 |
| ENSG00000186976 | EFCAB6 | -3.7852164 | 0.035595542 |
| ENSG00000230896 | AL604028.1 | -3.777359713 | 0.0000122 |
| ENSG00000205670 | SMIM11A | -3.775643019 | 0.049367032 |
| ENSG00000042062 | RIPOR3 | -3.773846979 | 0.003995002 |
| ENSG00000280417 | AC096887.2 | -3.772963829 | 0.001960993 |
| ENSG00000243701 | DUBR | -3.764393925 | 0.043359201 |
| ENSG00000081277 | PKP1 | -3.764216154 | 0.000168113 |
| ENSG00000151490 | PTPRO | -3.761624347 | 0.00000239 |
| ENSG00000255774 | AP000439.2 | -3.758769926 | 0.030801853 |
| ENSG00000222033 | LINC01124 | -3.756969725 | 3.52E-14 |
| ENSG00000210151 | MT-TS1 | -3.751057687 | 0.027419451 |
| ENSG00000269054 | AC012313.6 | -3.745012361 | 0.032030462 |
| ENSG00000244503 | AC108751.5 | -3.739550208 | 1.78E-10 |
| ENSG00000197557 | TTC30A | -3.731416049 | 9.9E-18 |
| ENSG00000172955 | ADH6 | -3.726245907 | 0.00000136 |
| ENSG00000230513 | THAP7-AS1 | -3.724841312 | 0.002965482 |
| ENSG00000227268 | KLLN | -3.722305837 | 0.000198752 |
| ENSG00000165376 | CLDN2 | -3.720435316 | 6.91E-17 |
| ENSG00000270001 | AL121894.2 | -3.719632337 | 0.009723862 |
| ENSG00000273270 | AC090114.2 | -3.713087249 | 4.26E-15 |
| ENSG00000077063 | CTTNBP2 | -3.712712064 | 9.39E-08 |
| ENSG00000262714 | AC007342.5 | -3.70442725 | 0.00000633 |
| ENSG00000241935 | HOGA1 | -3.699877818 | 0.000907625 |
| ENSG00000214654 | B3GNT10 | -3.697727974 | 1.01E-10 |
| ENSG00000171766 | GATM | -3.691649145 | 0.002534466 |
| ENSG00000267500 | ZNF887P | -3.691011248 | 0.015228633 |
| ENSG00000227252 | AC105760.2 | -3.69075592 | 0.042845146 |
| ENSG00000279949 | AC022784.8 | -3.690459384 | 0.042910062 |
| ENSG00000222017 | AC011997.1 | -3.690053537 | 0.027133959 |
| ENSG00000168062 | BATF2 | -3.690000125 | 2.87E-19 |
| ENSG00000105851 | PIK3CG | -3.6847895 | 1.11E-12 |
| ENSG00000175536 | LIPT2 | -3.679858139 | 5.11E-17 |
| ENSG00000228549 | BX284668.2 | -3.679850371 | 1.03E-09 |
| ENSG00000280623 | PCAT14 | -3.672641729 | 0.000017 |
| ENSG00000261934 | PCDHGA9 | -3.666942838 | 0.002340168 |
| ENSG00000203799 | CCDC162P | -3.666566454 | 0.03464341 |
| ENSG00000171234 | UGT2B7 | -3.648380098 | 0.003825866 |
| ENSG00000259583 | AC015712.2 | -3.638991431 | 4.01E-14 |
| ENSG00000110665 | C11orf21 | -3.629691411 | 0.000000427 |
| ENSG00000186496 | ZNF396 | -3.629308658 | 0.013751629 |
| ENSG00000109705 | NKX3-2 | -3.624276141 | 0.049565382 |
| ENSG00000233527 | ZNF529-AS1 | -3.619650536 | 0.036498182 |
| ENSG00000090534 | THPO | -3.614976934 | 0.010090251 |
| ENSG00000269896 | AL513477.1 | -3.610247938 | 1.87E-09 |
| ENSG00000274021 | AC024909.2 | -3.589778912 | 0.000474293 |
| ENSG00000277283 | AC004812.2 | -3.584484688 | 0.00000147 |
| ENSG00000165511 | C10orf25 | -3.579110822 | 0.00000117 |
| ENSG00000127954 | STEAP4 | -3.578410482 | 1.29E-48 |
| ENSG00000176049 | JAKMIP2 | -3.575947062 | 0.001317383 |
| ENSG00000134716 | CYP2J2 | -3.571743089 | 0.002369843 |
| ENSG00000270959 | LPP-AS2 | -3.560416231 | 0.0000231 |
| ENSG00000272189 | AL024508.2 | -3.554653926 | 1.06E-12 |
| ENSG00000176125 | UFSP1 | -3.552993479 | 8.07E-11 |
| ENSG00000182327 | GLTPD2 | -3.546193823 | 0.047077784 |
| ENSG00000177352 | CCDC71 | -3.537070755 | 2.68E-62 |
| ENSG00000260091 | AC093752.3 | -3.52578207 | 0.006341614 |
| ENSG00000100228 | RAB36 | -3.525111253 | 0.000000989 |
| ENSG00000164342 | TLR3 | -3.515465014 | 3.79E-24 |
| ENSG00000225675 | LINC01771 | -3.493161618 | 0.006133677 |
| ENSG00000278613 |  | -3.491179806 | 0.035155087 |
| ENSG00000266783 | AP005136.2 | -3.484311813 | 0.00000648 |
| ENSG00000278224 | PRICKLE4 | -3.483730756 | 0.009892984 |
| ENSG00000240889 | NDUFB2-AS1 | -3.483468195 | 0.004280142 |
| ENSG00000180938 | ZNF572 | -3.475968827 | 9.45E-35 |
| ENSG00000272711 | AC019069.1 | -3.470277511 | 0.00000131 |
| ENSG00000168874 | ATOH8 | -3.467860781 | 0.036371645 |
| ENSG00000248925 | AC021087.1 | -3.460332248 | 0.037790196 |
| ENSG00000272549 | LINC02538 | -3.45190148 | 0.000327267 |
| ENSG00000259974 | LINC00261 | -3.44888216 | 2.06E-21 |
| ENSG00000174370 | C11orf45 | -3.446757405 | 3.47E-10 |
| ENSG00000177459 | ERICH5 | -3.443209128 | 0.002237865 |
| ENSG00000236609 | ZNF853 | -3.44018787 | 0.042917929 |
| ENSG00000185261 | KIAA0825 | -3.440134887 | 0.008656688 |
| ENSG00000279348 | AC012513.3 | -3.439570004 | 5.52E-08 |
| ENSG00000273148 | AL035563.1 | -3.430936783 | 0.007473226 |
| ENSG00000165807 | PPP1R36 | -3.422300202 | 0.032710306 |
| ENSG00000221953 | C1orf229 | -3.421578684 | 0.030627941 |
| ENSG00000221949 | LINC01465 | -3.418777998 | 0.001422523 |
| ENSG00000183250 | LINC01547 | -3.416813737 | 0.0000562 |
| ENSG00000263327 | TAPT1-AS1 | -3.412504844 | 0.000167434 |
| ENSG00000187801 | ZFP69B | -3.397526235 | 1.41E-12 |
| ENSG00000282464 |  | -3.396675572 | 0.001268811 |
| ENSG00000272301 | AP002360.3 | -3.396028052 | 0.043123132 |
| ENSG00000280187 | AC022107.1 | -3.395648658 | 2.13E-09 |
| ENSG00000235142 | LINC02532 | -3.390997207 | 0.00039345 |
| ENSG00000126882 | FAM78A | -3.389434255 | 1.69E-13 |
| ENSG00000137393 | RNF144B | -3.383577179 | 1.24E-42 |
| ENSG00000226942 | IL9RP3 | -3.373481354 | 0.000174518 |
| ENSG00000185739 | SRL | -3.364662979 | 0.027360571 |
| ENSG00000255398 | HCAR3 | -3.363765719 | 0.002019104 |
| ENSG00000163909 | HEYL | -3.349874899 | 0.047468843 |
| ENSG00000261305 | AC005586.2 | -3.349239983 | 0.01794895 |
| ENSG00000234840 | LINC01239 | -3.341330967 | 0.00211606 |
| ENSG00000158186 | MRAS | -3.341081063 | 6.42E-09 |
| ENSG00000016602 | CLCA4 | -3.335029606 | 0.002262029 |
| ENSG00000267370 | AC008752.3 | -3.329704948 | 0.035147398 |
| ENSG00000227954 | TARID | -3.328614771 | 0.0000772 |
| ENSG00000137841 | PLCB2 | -3.321664958 | 0.047754816 |
| ENSG00000259674 | AC092868.1 | -3.312559134 | 1.13E-12 |
| ENSG00000178700 | DHFR2 | -3.312232696 | 3.28E-34 |
| ENSG00000232645 | LINC01431 | -3.304899828 | 0.048815972 |
| ENSG00000010295 | IFFO1 | -3.298674149 | 0.015550306 |
| ENSG00000279406 | AL359183.1 | -3.29819874 | 0.000164371 |
| ENSG00000225140 | AL358216.1 | -3.295047166 | 0.00007 |
| ENSG00000064201 | TSPAN32 | -3.292844991 | 0.003666819 |
| ENSG00000248727 | LINC01948 | -3.288746965 | 0.042597821 |
| ENSG00000102313 | ITIH6 | -3.288078502 | 0.006326846 |
| ENSG00000254593 | OR7E126P | -3.284930008 | 0.026671938 |
| ENSG00000137491 | SLCO2B1 | -3.275925492 | 0.000672735 |
| ENSG00000185065 | AC000068.1 | -3.269577748 | 5.53E-09 |
| ENSG00000210741 | MIR196A1 | -3.266192035 | 0.00042028 |
| ENSG00000267882 | AL031666.2 | -3.265407229 | 0.012666975 |
| ENSG00000120262 | CCDC170 | -3.264679237 | 0.001303845 |
| ENSG00000173928 | SWSAP1 | -3.253953824 | 2.49E-13 |
| ENSG00000166189 | HPS6 | -3.248670865 | 2.22E-70 |
| ENSG00000155850 | SLC26A2 | -3.244660141 | 3.39E-24 |
| ENSG00000272721 | AC131235.3 | -3.231721359 | 0.03342039 |
| ENSG00000260337 | AC091544.4 | -3.229125389 | 0.000802579 |
| ENSG00000226864 | ATE1-AS1 | -3.22808308 | 0.029164929 |
| ENSG00000259345 | AC013652.1 | -3.225528486 | 4.34E-08 |
| ENSG00000278834 | AC073508.3 | -3.209687088 | 0.00241982 |
| ENSG00000171772 | SYCE1 | -3.207671731 | 0.000914651 |
| ENSG00000166246 | C16orf71 | -3.202169911 | 0.001238078 |
| ENSG00000257135 | AC007249.2 | -3.199256847 | 0.0000136 |
| ENSG00000141642 | ELAC1 | -3.192062204 | 1.61E-12 |
| ENSG00000223473 |  | -3.182667203 | 0.002352338 |
| ENSG00000227630 | LINC01132 | -3.182340775 | 0.00000402 |
| ENSG00000197054 | ZNF763 | -3.182071958 | 0.043512697 |
| ENSG00000155016 | CYP2U1 | -3.177983553 | 3.35E-10 |
| ENSG00000196167 | COLCA1 | -3.168428981 | 3.52E-25 |
| ENSG00000267868 | AL356740.1 | -3.167287497 | 0.0000255 |
| ENSG00000280374 | AC019080.5 | -3.159180352 | 0.036503374 |
| ENSG00000233170 | AC138356.2 | -3.158229392 | 0.048703125 |
| ENSG00000122863 | CHST3 | -3.148743516 | 2.15E-19 |
| ENSG00000224536 | AC096677.1 | -3.148592142 | 0.020966694 |
| ENSG00000179044 | EXOC3L1 | -3.147718437 | 0.0000478 |
| ENSG00000249572 | AC034231.1 | -3.143279225 | 0.032610764 |
| ENSG00000171462 | DLK2 | -3.1358365 | 0.0000223 |
| ENSG00000102243 | VGLL1 | -3.130888719 | 2.04E-16 |
| ENSG00000259877 | AC009113.1 | -3.129581817 | 0.0000012 |
| ENSG00000236423 | LINC01134 | -3.120869777 | 0.004958732 |
| ENSG00000187815 | ZFP69 | -3.115178152 | 0.00000049 |
| ENSG00000182950 | ODF3L1 | -3.112523756 | 0.021634986 |
| ENSG00000146521 | LINC01558 | -3.107380538 | 0.020842295 |
| ENSG00000125848 | FLRT3 | -3.104920976 | 6.19E-31 |
| ENSG00000131746 | TNS4 | -3.099664206 | 5.46E-52 |
| ENSG00000142065 | ZFP14 | -3.09957036 | 0.008372274 |
| ENSG00000269086 | AC008555.2 | -3.09916905 | 0.006522706 |
| ENSG00000278484 | AC010998.3 | -3.097603951 | 7.35E-08 |
| ENSG00000169758 | TMEM266 | -3.094369962 | 0.02439479 |
| ENSG00000203688 | LINC02487 | -3.090213663 | 1.08E-08 |
| ENSG00000178372 | CALML5 | -3.08782404 | 0.000654197 |
| ENSG00000179023 | KLHDC7A | -3.08622797 | 7.65E-37 |
| ENSG00000277868 |  | -3.086143224 | 0.00000137 |
| ENSG00000154153 | RETREG1 | -3.086127623 | 0.019534314 |
| ENSG00000260992 | DOCK9-AS2 | -3.077988671 | 0.0000355 |
| ENSG00000111913 | RIPOR2 | -3.071591039 | 4.12E-11 |
| ENSG00000178977 | LINC00324 | -3.070087278 | 0.000721077 |
| ENSG00000188037 | CLCN1 | -3.068217406 | 1.55E-13 |
| ENSG00000165899 | OTOGL | -3.065114892 | 0.007597072 |
| ENSG00000187566 | NHLRC1 | -3.063146754 | 0.0000016 |
| ENSG00000281005 | LINC00921 | -3.061070256 | 0.039272061 |
| ENSG00000239713 | APOBEC3G | -3.059435904 | 0.0000115 |
| ENSG00000271851 | AC087501.4 | -3.039714585 | 0.012008582 |
| ENSG00000256771 | ZNF253 | -3.039537465 | 0.0000695 |
| ENSG00000154556 | SORBS2 | -3.037634394 | 1.15E-18 |
| ENSG00000251485 | AC068134.3 | -3.032510542 | 0.035185276 |
| ENSG00000213373 | LINC00671 | -3.032047484 | 0.004478535 |
| ENSG00000005379 | TSPOAP1 | -3.028945679 | 0.000109604 |
| ENSG00000167580 | AQP2 | -3.02730375 | 0.00000117 |
| ENSG00000116035 | VAX2 | -3.02489853 | 0.011167107 |
| ENSG00000203684 | IBA57-AS1 | -3.017293383 | 0.026924319 |
| ENSG00000221843 | C2orf16 | -3.017020666 | 6.46E-08 |
| ENSG00000264247 | LINC00909 | -3.016924775 | 1.48E-27 |
| ENSG00000273314 | AC005229.4 | -3.009592682 | 1.94E-09 |
| ENSG00000225968 | ELFN1 | -3.006020674 | 2.16E-08 |
| ENSG00000233421 | LINC01783 | -3.004167762 | 0.021526969 |
| ENSG00000280106 | AC008555.8 | -3.001251966 | 0.0000646 |
| ENSG00000272323 | AC026801.2 | -2.996093945 | 8.25E-08 |
| ENSG00000267296 | CEBPA-AS1 | -2.992501165 | 1.49E-09 |
| ENSG00000273456 | AC064836.3 | -2.990919704 | 0.002215625 |
| ENSG00000089335 | ZNF302 | -2.990794585 | 3.21E-21 |
| ENSG00000266208 | AC080112.1 | -2.990794573 | 0.000000161 |
| ENSG00000268157 | AC010524.1 | -2.987219216 | 0.000519324 |
| ENSG00000166359 | WDR88 | -2.983111752 | 0.000839286 |
| ENSG00000169169 | CPT1C | -2.981124448 | 0.029380373 |
| ENSG00000177640 | CASC2 | -2.974295428 | 0.00000818 |
| ENSG00000075340 | ADD2 | -2.971164055 | 0.0000129 |
| ENSG00000270127 | AC027020.2 | -2.96384314 | 0.000949948 |
| ENSG00000007952 | NOX1 | -2.956511505 | 3.4E-09 |
| ENSG00000088543 | C3orf18 | -2.950923536 | 0.0000169 |
| ENSG00000159915 | ZNF233 | -2.95037926 | 0.00000343 |
| ENSG00000188993 | LRRC66 | -2.949776569 | 5.7E-15 |
| ENSG00000164296 | TIGD6 | -2.94479537 | 2.78E-13 |
| ENSG00000254343 | AC091563.1 | -2.944633273 | 0.0000135 |
| ENSG00000171243 | SOSTDC1 | -2.939371521 | 4.72E-14 |
| ENSG00000178826 | TMEM139 | -2.926332212 | 8.2E-56 |
| ENSG00000271780 | AL118558.3 | -2.915598143 | 0.00000306 |
| ENSG00000273486 | AC096992.2 | -2.915136612 | 0.002162072 |
| ENSG00000066185 | ZMYND12 | -2.911166649 | 0.002421764 |
| ENSG00000240429 | LRRFIP1P1 | -2.908181996 | 0.00000371 |
| ENSG00000225950 | NTF4 | -2.907700392 | 0.031363093 |
| ENSG00000249464 | LINC01091 | -2.90485803 | 0.0000182 |
| ENSG00000232995 | AL592435.1 | -2.903778801 | 0.000363393 |
| ENSG00000214076 | CPSF1P1 | -2.899541396 | 0.007086042 |
| ENSG00000173080 | RXFP4 | -2.89801286 | 0.00000137 |
| ENSG00000108852 | MPP2 | -2.897764905 | 0.0000911 |
| ENSG00000168806 | LCMT2 | -2.896960823 | 2.65E-39 |
| ENSG00000198756 | COLGALT2 | -2.896339155 | 0.044452881 |
| ENSG00000152580 | IGSF10 | -2.895974655 | 3.77E-09 |
| ENSG00000159905 | ZNF221 | -2.893841858 | 0.000000193 |
| ENSG00000168939 | SPRY3 | -2.893600945 | 1.73E-09 |
| ENSG00000224046 | AC005076.1 | -2.893178079 | 0.006846475 |
| ENSG00000129048 | ACKR4 | -2.891468717 | 1.59E-12 |
| ENSG00000278291 | AL161772.1 | -2.891281512 | 0.0000126 |
| ENSG00000168907 | PLA2G4F | -2.890037194 | 1.99E-32 |
| ENSG00000236914 | LINC01852 | -2.889067084 | 0.00385146 |
| ENSG00000240571 | AC087071.2 | -2.885401833 | 0.018741816 |
| ENSG00000164404 | GDF9 | -2.877881689 | 0.005758648 |
| ENSG00000245498 | AP000866.1 | -2.876842654 | 0.002336765 |
| ENSG00000164379 | FOXQ1 | -2.876710423 | 6.52E-56 |
| ENSG00000180525 | PRR26 | -2.875289874 | 8.3E-24 |
| ENSG00000166897 | ELFN2 | -2.87429765 | 0.005221027 |
| ENSG00000210135 | MT-TN | -2.865898369 | 3.25E-15 |
| ENSG00000210127 | MT-TA | -2.862003392 | 0.000580004 |
| ENSG00000139508 | SLC46A3 | -2.860982523 | 8.6E-17 |
| ENSG00000107295 | SH3GL2 | -2.860099678 | 0.010521719 |
| ENSG00000186765 | FSCN2 | -2.855516281 | 0.003374206 |
| ENSG00000160094 | ZNF362 | -2.8520774 | 7.94E-10 |
| ENSG00000250654 | AC023794.5 | -2.851325636 | 0.009523679 |
| ENSG00000125998 | FAM83C | -2.850449803 | 0.0000652 |
| ENSG00000014257 | ACPP | -2.837243117 | 5.01E-09 |
| ENSG00000251669 | FAM86EP | -2.8273407 | 7.04E-17 |
| ENSG00000266088 | AC004585.1 | -2.826387944 | 0.001931663 |
| ENSG00000001626 | CFTR | -2.821851704 | 1.21E-09 |
| ENSG00000277782 | AC068870.2 | -2.819634761 | 0.0000211 |
| ENSG00000279673 | AC092919.2 | -2.817891853 | 2.57E-12 |
| ENSG00000076258 | FMO4 | -2.807742151 | 0.006683683 |
| ENSG00000237505 | PKN2-AS1 | -2.804793997 | 0.021881732 |
| ENSG00000165816 | VWA2 | -2.80342215 | 4.13E-31 |
| ENSG00000205559 | CHKB-AS1 | -2.801346744 | 0.035047101 |
| ENSG00000196876 | SCN8A | -2.799492762 | 0.000294697 |
| ENSG00000166509 | CLEC3A | -2.798676161 | 3.55E-31 |
| ENSG00000230189 | AC008267.2 | -2.793713037 | 0.002676229 |
| ENSG00000114853 | ZBTB47 | -2.792582214 | 0.000200107 |
| ENSG00000203739 | AL645568.1 | -2.790891297 | 0.028910232 |
| ENSG00000227775 | AL031282.1 | -2.790338229 | 1.53E-08 |
| ENSG00000070526 | ST6GALNAC1 | -2.782382768 | 3.45E-13 |
| ENSG00000235169 | SMIM1 | -2.780358826 | 0.0000522 |
| ENSG00000169955 | ZNF747 | -2.779896943 | 3.54E-11 |
| ENSG00000274750 | HIST1H3E | -2.778755729 | 0.001267093 |
| ENSG00000094755 | GABRP | -2.77710057 | 4.99E-33 |
| ENSG00000234390 | USP27X-AS1 | -2.776797758 | 0.002737201 |
| ENSG00000280254 | AC233723.2 | -2.776698825 | 0.047324145 |
| ENSG00000196917 | HCAR1 | -2.77544355 | 2.24E-44 |
| ENSG00000102924 | CBLN1 | -2.774743345 | 0.033617659 |
| ENSG00000273366 | Z83851.2 | -2.774725926 | 0.045379136 |
| ENSG00000078401 | EDN1 | -2.774528565 | 7.98E-36 |
| ENSG00000165102 | HGSNAT | -2.773989682 | 8.92E-15 |
| ENSG00000122711 | SPINK4 | -2.771449256 | 0.039177353 |
| ENSG00000271966 | AC021321.1 | -2.77084122 | 0.04976988 |
| ENSG00000184584 | TMEM173 | -2.769583071 | 1.42E-21 |
| ENSG00000157833 | GAREM2 | -2.76538966 | 0.018314764 |
| ENSG00000249626 | AC024560.2 | -2.763124358 | 0.000474199 |
| ENSG00000136869 | TLR4 | -2.759474 | 1.98E-22 |
| ENSG00000225285 | LINC01770 | -2.759324231 | 0.0000445 |
| ENSG00000257557 | PPP1R12A-AS1 | -2.758282607 | 0.001024835 |
| ENSG00000224769 | MUC20P1 | -2.755702928 | 0.000000469 |
| ENSG00000119042 | SATB2 | -2.755111238 | 0.02442748 |
| ENSG00000279329 | AC020910.5 | -2.74973626 | 0.044265312 |
| ENSG00000157429 | ZNF19 | -2.747183048 | 1.91E-08 |
| ENSG00000176753 | C15orf56 | -2.746340601 | 0.043457011 |
| ENSG00000203943 | SAMD13 | -2.746027886 | 5.78E-08 |
| ENSG00000176055 | MBLAC2 | -2.745318141 | 5.89E-20 |
| ENSG00000266921 | AC006213.1 | -2.744755245 | 0.018481153 |
| ENSG00000135374 | ELF5 | -2.743885957 | 0.0000169 |
| ENSG00000254231 | AC103760.1 | -2.743630995 | 0.007345867 |
| ENSG00000196196 | HRCT1 | -2.741522112 | 1.73E-11 |
| ENSG00000281879 |  | -2.741303732 | 0.025154282 |
| ENSG00000184208 | C22orf46 | -2.740010193 | 2.49E-15 |
| ENSG00000237149 | ZNF503-AS2 | -2.739484145 | 2.62E-13 |
| ENSG00000140323 | DISP2 | -2.737437123 | 2.75E-15 |
| ENSG00000106336 | FBXO24 | -2.73439354 | 0.047859001 |
| ENSG00000112494 | UNC93A | -2.73248327 | 0.000000597 |
| ENSG00000122859 | NEUROG3 | -2.726880091 | 0.022537743 |
| ENSG00000182405 | PGBD4 | -2.725389163 | 2.28E-14 |
| ENSG00000233175 | AC008105.1 | -2.723282219 | 0.016719802 |
| ENSG00000177191 | B3GNT8 | -2.719314424 | 0.001130172 |
| ENSG00000198133 | TMEM229B | -2.718554998 | 0.000684808 |
| ENSG00000172058 | SERF1A | -2.717839919 | 0.044489667 |
| ENSG00000237857 |  | -2.717294162 | 1.44E-10 |
| ENSG00000175920 | DOK7 | -2.714168176 | 0.000000751 |
| ENSG00000124217 | MOCS3 | -2.709798466 | 1.81E-68 |
| ENSG00000146955 | RAB19 | -2.708700247 | 0.000130367 |
| ENSG00000149582 | TMEM25 | -2.707623314 | 0.00000107 |
| ENSG00000213801 | ZNF321P | -2.705720591 | 7.02E-18 |
| ENSG00000120049 | KCNIP2 | -2.701015261 | 0.026760891 |
| ENSG00000232040 | ZBED9 | -2.700838908 | 0.000000217 |
| ENSG00000203499 | IQANK1 | -2.700341591 | 0.023997714 |
| ENSG00000223658 | C1GALT1C1L | -2.697270547 | 0.0000789 |
| ENSG00000225556 | C2CD4D | -2.692888435 | 0.00000153 |
| ENSG00000112782 | CLIC5 | -2.692509014 | 0.0000057 |
| ENSG00000231345 | BEND3P1 | -2.691879307 | 8.88E-31 |
| ENSG00000217165 | ANKRD18EP | -2.689162969 | 0.000000101 |
| ENSG00000072952 | MRVI1 | -2.686707176 | 0.016149956 |
| ENSG00000175538 | KCNE3 | -2.686583407 | 4E-66 |
| ENSG00000107954 | NEURL1 | -2.681378795 | 0.0000115 |
| ENSG00000143001 | TMEM61 | -2.681326839 | 0.048586981 |
| ENSG00000197361 | FBXL22 | -2.679946146 | 0.0000515 |
| ENSG00000237489 | C10orf143 | -2.679899345 | 0.025117692 |
| ENSG00000204584 | AC027801.1 | -2.677963111 | 0.000565858 |
| ENSG00000278611 | ZNF426-DT | -2.675831362 | 0.040144133 |
| ENSG00000226752 | CUTALP | -2.675189163 | 1.21E-39 |
| ENSG00000062370 | ZNF112 | -2.673347678 | 1.12E-10 |
| ENSG00000163515 | RETNLB | -2.670951883 | 0.003569098 |
| ENSG00000249267 | LINC00939 | -2.667434472 | 0.044422268 |
| ENSG00000138080 | EMILIN1 | -2.664634069 | 0.003853609 |
| ENSG00000110777 | POU2AF1 | -2.658155743 | 2.4E-11 |
| ENSG00000165091 | TMC1 | -2.657751923 | 0.000595417 |
| ENSG00000079308 | TNS1 | -2.653639617 | 3.91E-14 |
| ENSG00000280161 | AC022413.1 | -2.653600048 | 0.025328864 |
| ENSG00000231856 | AL162377.1 | -2.65341648 | 0.0000182 |
| ENSG00000198835 | GJC2 | -2.64940685 | 1.26E-41 |
| ENSG00000213062 | AL021068.1 | -2.64628713 | 0.00036352 |
| ENSG00000165879 | FRAT1 | -2.644920292 | 9.11E-20 |
| ENSG00000129595 | EPB41L4A | -2.644405255 | 0.0000217 |
| ENSG00000221886 | ZBED8 | -2.64429879 | 6.87E-12 |
| ENSG00000143315 | PIGM | -2.642703784 | 3.25E-27 |
| ENSG00000008853 | RHOBTB2 | -2.642454936 | 5.15E-21 |
| ENSG00000179627 | ZBTB42 | -2.640333112 | 5.16E-52 |
| ENSG00000260267 | AC026471.1 | -2.639183675 | 4.45E-11 |
| ENSG00000145476 | CYP4V2 | -2.6378602 | 1.09E-20 |
| ENSG00000005471 | ABCB4 | -2.637748053 | 0.028174457 |
| ENSG00000127084 | FGD3 | -2.63678595 | 2.71E-23 |
| ENSG00000171084 | FAM86JP | -2.63599197 | 3.71E-18 |
| ENSG00000162757 | C1orf74 | -2.634735265 | 6.77E-21 |
| ENSG00000230102 | LINC02028 | -2.632822987 | 0.008593602 |
| ENSG00000206285 |  | -2.629751845 | 0.024468085 |
| ENSG00000266947 | AC022916.1 | -2.626961846 | 0.002308723 |
| ENSG00000261437 | AC108860.2 | -2.625920808 | 3.18E-14 |
| ENSG00000240207 | AC080013.1 | -2.623123378 | 0.0000017 |
| ENSG00000249923 | AC007663.2 | -2.621406301 | 0.031137084 |
| ENSG00000159173 | TNNI1 | -2.619188716 | 3.24E-12 |
| ENSG00000074621 | SLC24A1 | -2.618767655 | 6.55E-21 |
| ENSG00000261469 | AC020978.4 | -2.617696733 | 0.037848432 |
| ENSG00000137709 | POU2F3 | -2.615741397 | 0.00000383 |
| ENSG00000174327 | SLC16A13 | -2.615366183 | 1.98E-08 |
| ENSG00000168350 | DEGS2 | -2.613631382 | 1.06E-61 |
| ENSG00000125966 | MMP24 | -2.609622457 | 6.26E-14 |
| ENSG00000255545 | AP004608.1 | -2.609337583 | 0.0000423 |
| ENSG00000144908 | ALDH1L1 | -2.608870848 | 3.66E-13 |
| ENSG00000230487 | PSMG3-AS1 | -2.608757725 | 0.000000091 |
| ENSG00000138400 | MDH1B | -2.607594729 | 0.035595542 |
| ENSG00000139531 | SUOX | -2.605862106 | 5.02E-49 |
| ENSG00000253846 | PCDHGA10 | -2.604156262 | 3.46E-10 |
| ENSG00000100373 | UPK3A | -2.602763798 | 5.47E-10 |
| ENSG00000124406 | ATP8A1 | -2.600243189 | 0.000000247 |
| ENSG00000226065 | ZBTB45P2 | -2.600226697 | 0.00000337 |
| ENSG00000128408 | RIBC2 | -2.598182235 | 0.000000404 |
| ENSG00000177465 | ACOT4 | -2.594774897 | 1.4E-10 |
| ENSG00000244617 | ASPRV1 | -2.594578236 | 0.000674968 |
| ENSG00000130433 | CACNG6 | -2.594432121 | 0.000108522 |
| ENSG00000186409 | CCDC30 | -2.590313988 | 0.000556327 |
| ENSG00000227258 | SMIM2-AS1 | -2.586171504 | 0.014695718 |
| ENSG00000226306 | NPY6R | -2.585034666 | 0.002440217 |
| ENSG00000261643 | AL590235.1 | -2.582295378 | 0.042164861 |
| ENSG00000144843 | ADPRH | -2.581723721 | 0.022785768 |
| ENSG00000176022 | B3GALT6 | -2.581318166 | 1.45E-46 |
| ENSG00000267194 |  | -2.580773618 | 0.0000358 |
| ENSG00000223298 | RNY3P8 | -2.577785934 | 0.015571582 |
| ENSG00000114654 | EFCC1 | -2.573797557 | 0.028104227 |
| ENSG00000258512 | LINC00239 | -2.573009001 | 2.91E-09 |
| ENSG00000210140 | MT-TC | -2.571881958 | 8.45E-16 |
| ENSG00000205116 | TMEM88B | -2.565622482 | 0.001687066 |
| ENSG00000282393 | AC016588.2 | -2.565423376 | 0.00000418 |
| ENSG00000258920 | FOXN3-AS1 | -2.561918218 | 0.021504323 |
| ENSG00000146243 | IRAK1BP1 | -2.559947618 | 0.001524528 |
| ENSG00000158806 | NPM2 | -2.553789729 | 0.003226212 |
| ENSG00000274979 | AC020656.2 | -2.55032417 | 0.000104345 |
| ENSG00000186666 | BCDIN3D | -2.549443531 | 1.67E-12 |
| ENSG00000042980 | ADAM28 | -2.54908426 | 0.0000194 |
| ENSG00000049192 | ADAMTS6 | -2.548077686 | 4.08E-09 |
| ENSG00000122035 | RASL11A | -2.547673166 | 2.81E-35 |
| ENSG00000272172 | AC138696.2 | -2.54508822 | 0.031369657 |
| ENSG00000197841 | ZNF181 | -2.541973607 | 7.45E-35 |
| ENSG00000004139 | SARM1 | -2.541933882 | 4.04E-16 |
| ENSG00000225345 | SNX18P3 | -2.540584936 | 0.009438465 |
| ENSG00000196981 | WDR5B | -2.540487288 | 9.64E-38 |
| ENSG00000276317 | AL357033.3 | -2.539598244 | 0.000102846 |
| ENSG00000236081 | ELFN1-AS1 | -2.536474343 | 5.75E-15 |
| ENSG00000174137 | FAM53A | -2.534972549 | 7.49E-08 |
| ENSG00000188175 | HEPACAM2 | -2.530310317 | 0.000883096 |
| ENSG00000228223 | HCG11 | -2.529718232 | 0.000330423 |
| ENSG00000225793 | AL080250.1 | -2.52969419 | 0.031176127 |
| ENSG00000280303 | ERICD | -2.527116637 | 0.024759445 |
| ENSG00000187942 | LDLRAD2 | -2.5265945 | 0.0351949 |
| ENSG00000080947 | CROCCP3 | -2.518855179 | 0.002513261 |
| ENSG00000260766 |  | -2.515581619 | 0.00000953 |
| ENSG00000131910 | NR0B2 | -2.512073716 | 1.59E-21 |
| ENSG00000129295 | LRRC6 | -2.504698014 | 0.000105877 |
| ENSG00000253873 | PCDHGA11 | -2.503941729 | 0.038821783 |
| ENSG00000172296 | SPTLC3 | -2.502299028 | 0.0000195 |
| ENSG00000163491 | NEK10 | -2.500346848 | 0.000711852 |
| ENSG00000203865 | ATP1A1-AS1 | -2.500172476 | 0.00023163 |
| ENSG00000182810 | DDX28 | -2.499606691 | 6.46E-39 |
| ENSG00000175764 | TTLL11 | -2.495863903 | 0.005199151 |
| ENSG00000182795 | C1orf116 | -2.495577603 | 2.16E-45 |
| ENSG00000188039 | NWD1 | -2.494193817 | 0.000366739 |
| ENSG00000197603 | C5orf42 | -2.48998276 | 0.0000391 |
| ENSG00000280027 | AC007342.9 | -2.486597584 | 0.005545893 |
| ENSG00000271918 | AC034236.2 | -2.484936241 | 0.017194306 |
| ENSG00000210196 | MT-TP | -2.482905672 | 6.62E-20 |
| ENSG00000227477 | STK4-AS1 | -2.482854465 | 0.042692509 |
| ENSG00000178750 | STX19 | -2.481729387 | 2.27E-14 |
| ENSG00000114646 | CSPG5 | -2.472027415 | 0.00000315 |
| ENSG00000185432 | METTL7A | -2.469159009 | 9.88E-12 |
| ENSG00000129749 | CHRNA10 | -2.468726785 | 0.000091 |
| ENSG00000187699 | C2orf88 | -2.467251716 | 0.013176761 |
| ENSG00000184254 | ALDH1A3 | -2.462570218 | 6.07E-29 |
| ENSG00000280173 | AC104447.1 | -2.461313987 | 0.00131398 |
| ENSG00000153029 | MR1 | -2.461210019 | 0.000000143 |
| ENSG00000181585 | TMIE | -2.461180334 | 0.022632228 |
| ENSG00000260704 | LINC00543 | -2.451461991 | 0.00000035 |
| ENSG00000273382 | AL356488.3 | -2.445788348 | 0.03285066 |
| ENSG00000260804 | LINC01963 | -2.444851158 | 0.00000113 |
| ENSG00000213888 | LINC01521 | -2.444013512 | 0.008654759 |
| ENSG00000261126 | RBFADN | -2.443378797 | 0.038039313 |
| ENSG00000223669 | AL357033.2 | -2.44187226 | 0.0000647 |
| ENSG00000188707 | ZBED6CL | -2.440581105 | 2.87E-21 |
| ENSG00000169783 | LINGO1 | -2.438365073 | 0.00029888 |
| ENSG00000159917 | ZNF235 | -2.434292715 | 1.91E-12 |
| ENSG00000248866 | USP46-AS1 | -2.432825989 | 0.0000443 |
| ENSG00000168661 | ZNF30 | -2.431720251 | 8.73E-25 |
| ENSG00000064787 | BCAS1 | -2.431075197 | 1.5E-59 |
| ENSG00000273084 | AC092171.5 | -2.430432557 | 0.00000187 |
| ENSG00000151882 | CCL28 | -2.428794899 | 3.8E-16 |
| ENSG00000181104 | F2R | -2.419890241 | 8.53E-35 |
| ENSG00000257829 | AC121757.1 | -2.419673101 | 0.021747708 |
| ENSG00000108242 | CYP2C18 | -2.415286096 | 0.037819029 |
| ENSG00000258738 | AL121603.2 | -2.414883581 | 0.00015188 |
| ENSG00000078814 | MYH7B | -2.414427236 | 0.004134488 |
| ENSG00000260742 | AC009962.1 | -2.412199651 | 0.034971551 |
| ENSG00000204282 | TNRC6C-AS1 | -2.410392913 | 2.7E-18 |
| ENSG00000090612 | ZNF268 | -2.408091245 | 1.9E-18 |
| ENSG00000131849 | ZNF132 | -2.406630214 | 0.000699021 |
| ENSG00000261188 | Z95115.1 | -2.402869087 | 7.26E-15 |
| ENSG00000226465 | AL390198.1 | -2.39347198 | 0.002904637 |
| ENSG00000243766 | HOTTIP | -2.391737541 | 2.16E-30 |
| ENSG00000229619 | MBNL1-AS1 | -2.388182114 | 0.000103835 |
| ENSG00000182584 | ACTL10 | -2.384602702 | 8.49E-16 |
| ENSG00000152475 | ZNF837 | -2.384573892 | 7.98E-17 |
| ENSG00000196810 | CTBP1-AS2 | -2.378521596 | 1.05E-25 |
| ENSG00000065534 | MYLK | -2.378005106 | 1.43E-22 |
| ENSG00000277200 | AC005696.4 | -2.376251995 | 0.019956081 |
| ENSG00000174514 | MFSD4A | -2.376169798 | 0.00000281 |
| ENSG00000260806 | AL163051.1 | -2.374169911 | 0.011676715 |
| ENSG00000172238 | ATOH1 | -2.371011888 | 0.000133513 |
| ENSG00000230658 | KLHL7-AS1 | -2.369835284 | 0.026183988 |
| ENSG00000275734 | AC010538.1 | -2.369797704 | 0.039538325 |
| ENSG00000224086 | AC245452.1 | -2.369558443 | 0.005326161 |
| ENSG00000277449 | CEBPB-AS1 | -2.368773588 | 0.001768722 |
| ENSG00000185630 | PBX1 | -2.36472177 | 1.03E-22 |
| ENSG00000185730 | ZNF696 | -2.361647198 | 4.34E-27 |
| ENSG00000130508 | PXDN | -2.359747246 | 0.000133759 |
| ENSG00000260966 | AP001486.2 | -2.35741057 | 0.000081 |
| ENSG00000115896 | PLCL1 | -2.356886138 | 0.0009334 |
| ENSG00000139178 | C1RL | -2.355836899 | 3.67E-24 |
| ENSG00000247934 | AC022364.1 | -2.355708524 | 0.011056157 |
| ENSG00000261235 | AC092142.1 | -2.355239887 | 0.029060813 |
| ENSG00000167653 | PSCA | -2.354889688 | 1.69E-13 |
| ENSG00000085276 | MECOM | -2.354250507 | 1.7E-45 |
| ENSG00000261804 | AC007342.4 | -2.353363486 | 0.00000458 |
| ENSG00000161328 | LRRC56 | -2.352439138 | 0.000000297 |
| ENSG00000186280 | KDM4D | -2.350643697 | 0.004160129 |
| ENSG00000196418 | ZNF124 | -2.348679797 | 7.92E-15 |
| ENSG00000162999 | DUSP19 | -2.348058595 | 0.00000264 |
| ENSG00000266405 | CBX3P2 | -2.3478438 | 0.027184266 |
| ENSG00000273893 | AL133520.1 | -2.344884133 | 0.013489948 |
| ENSG00000186272 | ZNF17 | -2.342431651 | 2.31E-20 |
| ENSG00000271109 | AC008555.5 | -2.342370755 | 0.000000117 |
| ENSG00000237721 | AF064858.3 | -2.341565978 | 0.0000825 |
| ENSG00000107984 | DKK1 | -2.338797791 | 0.000123725 |
| ENSG00000087495 | PHACTR3 | -2.338163075 | 0.004392648 |
| ENSG00000278709 | NKILA | -2.338139387 | 6.13E-08 |
| ENSG00000166816 | LDHD | -2.337042652 | 0.000129518 |
| ENSG00000240875 | LINC00886 | -2.333877004 | 0.000000365 |
| ENSG00000127329 | PTPRB | -2.333312491 | 9.11E-27 |
| ENSG00000272631 | AC067750.1 | -2.331868088 | 0.022356035 |
| ENSG00000281490 | CICP14 | -2.329229564 | 2.48E-10 |
| ENSG00000099957 | P2RX6 | -2.328808787 | 0.002447505 |
| ENSG00000197128 | ZNF772 | -2.327441296 | 0.000152035 |
| ENSG00000146700 | SSC4D | -2.327360689 | 0.004344186 |
| ENSG00000143434 | SEMA6C | -2.322556178 | 0.000500427 |
| ENSG00000167741 | GGT6 | -2.321373301 | 8.05E-13 |
| ENSG00000242498 | ARPIN | -2.319099903 | 2.07E-38 |
| ENSG00000280710 | AL139035.1 | -2.31524043 | 0.034048645 |
| ENSG00000175283 | DOLK | -2.314291292 | 1.79E-43 |
| ENSG00000269044 | AC024075.2 | -2.313062108 | 0.000382569 |
| ENSG00000254837 | AP001372.2 | -2.311559851 | 2.1E-30 |
| ENSG00000183208 | GDPGP1 | -2.310247644 | 1.71E-19 |
| ENSG00000198890 | PRMT6 | -2.310245209 | 2.42E-21 |
| ENSG00000265458 | AC132938.3 | -2.309657843 | 0.015125436 |
| ENSG00000172367 | PDZD3 | -2.309519671 | 0.0000231 |
| ENSG00000256294 | ZNF225 | -2.30923867 | 1.71E-15 |
| ENSG00000162344 | FGF19 | -2.304147609 | 1.18E-40 |
| ENSG00000274270 | AL137060.1 | -2.297933351 | 0.007957917 |
| ENSG00000188001 | TPRG1 | -2.297873713 | 0.029969779 |
| ENSG00000146205 | ANO7 | -2.29748825 | 3.66E-09 |
| ENSG00000095596 | CYP26A1 | -2.296822779 | 0.00806016 |
| ENSG00000121236 | TRIM6 | -2.291208705 | 0.02169189 |
| ENSG00000267221 | C17orf113 | -2.287180154 | 0.000000104 |
| ENSG00000129480 | DTD2 | -2.282402471 | 1.05E-17 |
| ENSG00000169507 | SLC38A11 | -2.279673486 | 2.59E-16 |
| ENSG00000274104 | AC020910.4 | -2.278952357 | 0.006970715 |
| ENSG00000234432 | AC092171.3 | -2.278299115 | 1.02E-09 |
| ENSG00000204128 | C2orf72 | -2.276292032 | 5.79E-14 |
| ENSG00000198429 | ZNF69 | -2.275376735 | 1.58E-11 |
| ENSG00000198298 | ZNF485 | -2.27531969 | 1.36E-08 |
| ENSG00000271643 | AC112220.4 | -2.275102584 | 0.000878315 |
| ENSG00000118965 | WDR35 | -2.274022638 | 6.48E-16 |
| ENSG00000179406 | LINC00174 | -2.270845749 | 0.001342513 |
| ENSG00000106477 | CEP41 | -2.268853691 | 1.03E-23 |
| ENSG00000226887 | ERVMER34-1 | -2.267997271 | 9.77E-10 |
| ENSG00000174145 | NWD2 | -2.263791181 | 0.000645056 |
| ENSG00000258056 | AC009779.2 | -2.262723205 | 2.35E-14 |
| ENSG00000272031 | ANKRD34A | -2.260451274 | 0.029116065 |
| ENSG00000030419 | IKZF2 | -2.258671691 | 6.32E-22 |
| ENSG00000254851 | AP005018.2 | -2.257675832 | 0.025430019 |
| ENSG00000272476 | AL024507.2 | -2.25699721 | 0.033329218 |
| ENSG00000279267 | AL078621.3 | -2.252311038 | 0.0000179 |
| ENSG00000132357 | CARD6 | -2.251177459 | 3.56E-10 |
| ENSG00000138380 | CARF | -2.250960253 | 1.53E-08 |
| ENSG00000171121 | KCNMB3 | -2.249173482 | 0.001031049 |
| ENSG00000164953 | TMEM67 | -2.248979656 | 1.58E-12 |
| ENSG00000228624 | HDAC2-AS2 | -2.248569924 | 0.027985771 |
| ENSG00000242207 | HOXB-AS4 | -2.247694977 | 5.66E-14 |
| ENSG00000135472 | FAIM2 | -2.245427726 | 1.06E-10 |
| ENSG00000234616 | JRK | -2.242713733 | 6.67E-28 |
| ENSG00000180884 | ZNF792 | -2.240635529 | 5.79E-27 |
| ENSG00000198513 | ATL1 | -2.238873534 | 0.023312393 |
| ENSG00000188761 | BCL2L15 | -2.238571194 | 2.65E-14 |
| ENSG00000147124 | ZNF41 | -2.238300714 | 2.2E-29 |
| ENSG00000260920 | AL031985.3 | -2.236111299 | 0.000101022 |
| ENSG00000254838 | GVINP1 | -2.236085745 | 0.000432696 |
| ENSG00000224424 | PRKAR2A-AS1 | -2.234431731 | 0.020517404 |
| ENSG00000214456 | PLIN5 | -2.233637167 | 0.000594495 |
| ENSG00000268996 | MAN1B1-AS1 | -2.232401252 | 0.0000462 |
| ENSG00000259673 | IQCH-AS1 | -2.232285955 | 1.4E-09 |
| ENSG00000249471 | ZNF324B | -2.232054682 | 1.89E-20 |
| ENSG00000282417 |  | -2.229042005 | 0.0000019 |
| ENSG00000204947 | ZNF425 | -2.227169282 | 1.7E-10 |
| ENSG00000171401 | KRT13 | -2.224538351 | 9.54E-14 |
| ENSG00000230937 | MIR205HG | -2.219863059 | 0.010998603 |
| ENSG00000271122 | AC018647.2 | -2.219403166 | 1.49E-10 |
| ENSG00000279069 | AC015813.5 | -2.218895359 | 0.00086161 |
| ENSG00000006756 | ARSD | -2.21775678 | 1.59E-35 |
| ENSG00000137486 | ARRB1 | -2.215516459 | 1.08E-57 |
| ENSG00000259642 | ST20-AS1 | -2.214095613 | 1.1E-14 |
| ENSG00000257167 | TMPO-AS1 | -2.213530213 | 0.00000032 |
| ENSG00000270055 | AC127502.2 | -2.211474872 | 0.003928796 |
| ENSG00000215190 | LINC00680 | -2.210743654 | 2.13E-11 |
| ENSG00000153292 | ADGRF1 | -2.210670544 | 4.47E-26 |
| ENSG00000124104 | SNX21 | -2.209137235 | 3.77E-16 |
| ENSG00000124160 | NCOA5 | -2.202802329 | 1.02E-46 |
| ENSG00000233017 | AL121832.1 | -2.201355187 | 0.002041348 |
| ENSG00000135697 | BCO1 | -2.199910424 | 0.000201812 |
| ENSG00000182141 | ZNF708 | -2.198652319 | 4.16E-09 |
| ENSG00000273812 | BX640514.2 | -2.198372894 | 0.000000372 |
| ENSG00000157657 | ZNF618 | -2.19678487 | 0.000978092 |
| ENSG00000182057 | OGFRP1 | -2.194719799 | 0.000358474 |
| ENSG00000125508 | SRMS | -2.193065036 | 0.0000647 |
| ENSG00000162341 | TPCN2 | -2.192408533 | 8.14E-28 |
| ENSG00000166192 | SENP8 | -2.190124043 | 1.22E-13 |
| ENSG00000076555 | ACACB | -2.189491823 | 6.9E-10 |
| ENSG00000135439 | AGAP2 | -2.188432911 | 1.73E-09 |
| ENSG00000227473 | TSSK5P | -2.186344906 | 0.000000288 |
| ENSG00000271430 |  | -2.186068104 | 0.000572013 |
| ENSG00000255306 | AC004923.4 | -2.181364144 | 0.005056675 |
| ENSG00000267395 | DM1-AS | -2.176190259 | 0.037243315 |
| ENSG00000065413 | ANKRD44 | -2.174736363 | 1.24E-11 |
| ENSG00000175548 | ALG10B | -2.173910101 | 8.47E-30 |
| ENSG00000167384 | ZNF180 | -2.172643751 | 4.34E-32 |
| ENSG00000234155 | LINC02535 | -2.171997615 | 0.00000278 |
| ENSG00000152926 | ZNF117 | -2.169506637 | 0.0000756 |
| ENSG00000185483 | ROR1 | -2.169034944 | 1.97E-22 |
| ENSG00000137868 | STRA6 | -2.168755425 | 0.00000357 |
| ENSG00000156239 | N6AMT1 | -2.16496628 | 5.49E-13 |
| ENSG00000198938 | MT-CO3 | -2.164470676 | 0.00000268 |
| ENSG00000282810 |  | -2.162905099 | 0.002074569 |
| ENSG00000260686 | AC008669.1 | -2.162535228 | 0.000359747 |
| ENSG00000260285 | AL133367.1 | -2.159022353 | 0.00000485 |
| ENSG00000265055 | AC145343.1 | -2.158516167 | 0.025627217 |
| ENSG00000129646 | QRICH2 | -2.154599158 | 0.045750411 |
| ENSG00000171757 | LRRC34 | -2.153270101 | 0.003972674 |
| ENSG00000065457 | ADAT1 | -2.152347317 | 2.01E-25 |
| ENSG00000135363 | LMO2 | -2.152268706 | 0.026192134 |
| ENSG00000116039 | ATP6V1B1 | -2.151923972 | 1.27E-21 |
| ENSG00000235888 | AF064858.1 | -2.149696679 | 0.000000554 |
| ENSG00000274349 | ZNF658 | -2.148404405 | 0.000347671 |
| ENSG00000110455 | ACCS | -2.148320217 | 3.92E-10 |
| ENSG00000235944 | ZNF815P | -2.147518309 | 0.000000958 |
| ENSG00000189157 | FAM47E | -2.146244122 | 0.000193577 |
| ENSG00000114019 | AMOTL2 | -2.144095643 | 5.24E-42 |
| ENSG00000244300 | GATA2-AS1 | -2.143477883 | 4.78E-25 |
| ENSG00000166741 | NNMT | -2.143017729 | 1.91E-15 |
| ENSG00000185085 | INTS5 | -2.142779857 | 1.35E-69 |
| ENSG00000241186 | TDGF1 | -2.140981608 | 0.000000214 |
| ENSG00000256802 | AC022613.1 | -2.140645627 | 6.04E-12 |
| ENSG00000268087 | AC008764.2 | -2.14058608 | 0.021956348 |
| ENSG00000254363 | AC011379.2 | -2.138398686 | 0.035709396 |
| ENSG00000258929 |  | -2.138365805 | 0.000099 |
| ENSG00000182901 | RGS7 | -2.136788488 | 0.01448862 |
| ENSG00000144115 | THNSL2 | -2.135929547 | 1.84E-10 |
| ENSG00000227354 | RBM26-AS1 | -2.134719733 | 0.00115564 |
| ENSG00000277662 | AL354696.1 | -2.134022264 | 0.001789593 |
| ENSG00000123453 | SARDH | -2.133231274 | 0.000000451 |
| ENSG00000269343 | ZNF587B | -2.130937691 | 7.8E-24 |
| ENSG00000177946 | CENPBD1 | -2.12824421 | 2.11E-17 |
| ENSG00000254685 | FPGT | -2.127415706 | 5.73E-20 |
| ENSG00000124593 | AL365205.1 | -2.126382243 | 3.04E-18 |
| ENSG00000168792 | ABHD15 | -2.124369127 | 1.16E-43 |
| ENSG00000007306 | CEACAM7 | -2.121564883 | 0.016543041 |
| ENSG00000267493 | CIRBP-AS1 | -2.119703244 | 0.009527346 |
| ENSG00000106025 | TSPAN12 | -2.118551138 | 3.17E-20 |
| ENSG00000144362 | PHOSPHO2 | -2.117430724 | 0.00000005 |
| ENSG00000112294 | ALDH5A1 | -2.115468219 | 6.37E-13 |
| ENSG00000170364 | SETMAR | -2.115087694 | 7.78E-16 |
| ENSG00000279382 | AC018665.1 | -2.114770658 | 0.003052536 |
| ENSG00000163884 | KLF15 | -2.114283621 | 1.4E-25 |
| ENSG00000189058 | APOD | -2.114108542 | 0.01001136 |
| ENSG00000234684 | SDCBP2-AS1 | -2.11193375 | 0.00000526 |
| ENSG00000233382 | NKAPP1 | -2.110566111 | 0.000000322 |
| ENSG00000273355 | AP000894.4 | -2.109665862 | 0.023821912 |
| ENSG00000164002 | EXO5 | -2.109320642 | 3.1E-22 |
| ENSG00000153363 | LINC00467 | -2.109162013 | 5.61E-17 |
| ENSG00000186889 | TMEM17 | -2.109077873 | 0.00002 |
| ENSG00000072858 | SIDT1 | -2.105945257 | 3.85E-22 |
| ENSG00000176933 | TOB2P1 | -2.104307109 | 0.000666237 |
| ENSG00000185774 | KCNIP4 | -2.094212513 | 0.0000489 |
| ENSG00000275765 | AC091982.3 | -2.091970891 | 0.001084405 |
| ENSG00000237061 | AC100823.1 | -2.091258559 | 0.031382203 |
| ENSG00000162543 | UBXN10 | -2.089440029 | 1.64E-10 |
| ENSG00000278416 | PMS2P2 | -2.087829207 | 0.011161427 |
| ENSG00000179240 | GVQW3 | -2.085883901 | 0.00000131 |
| ENSG00000203644 | AC083799.1 | -2.085318979 | 2.04E-09 |
| ENSG00000132623 | ANKEF1 | -2.084917222 | 9.95E-37 |
| ENSG00000132938 | MTUS2 | -2.083587571 | 0.001317383 |
| ENSG00000139291 | TMEM19 | -2.081805142 | 2.16E-21 |
| ENSG00000153064 | BANK1 | -2.080575269 | 1.94E-09 |
| ENSG00000272144 | AC025171.4 | -2.079673326 | 0.01847043 |
| ENSG00000119686 | FLVCR2 | -2.078381505 | 0.000000045 |
| ENSG00000100413 | POLR3H | -2.07693588 | 1.26E-17 |
| ENSG00000141469 | SLC14A1 | -2.073268917 | 0.000450675 |
| ENSG00000162989 | KCNJ3 | -2.069733782 | 2.99E-15 |
| ENSG00000280441 | FP236383.1 | -2.069324116 | 0.00542032 |
| ENSG00000189298 | ZKSCAN3 | -2.069274179 | 2.75E-11 |
| ENSG00000143995 | MEIS1 | -2.069088443 | 0.0000934 |
| ENSG00000158023 | WDR66 | -2.067738541 | 0.00000011 |
| ENSG00000169105 | CHST14 | -2.066935777 | 1.73E-30 |
| ENSG00000198840 | MT-ND3 | -2.065620689 | 2.92E-33 |
| ENSG00000163072 | NOSTRIN | -2.064999083 | 1.09E-08 |
| ENSG00000086159 | AQP6 | -2.064748947 | 0.040309031 |
| ENSG00000120925 | RNF170 | -2.063212043 | 1.21E-10 |
| ENSG00000257588 | AC025154.2 | -2.062014541 | 1.4E-17 |
| ENSG00000205808 | PLPP6 | -2.060018528 | 4.14E-25 |
| ENSG00000188322 | SBK1 | -2.057657921 | 9.03E-11 |
| ENSG00000241852 | C8orf58 | -2.053759353 | 0.0000112 |
| ENSG00000150773 | PIH1D2 | -2.049954721 | 0.002720169 |
| ENSG00000238005 | AL391832.2 | -2.04939812 | 0.026077319 |
| ENSG00000115423 | DNAH6 | -2.049390317 | 0.022960932 |
| ENSG00000276856 |  | -2.048270388 | 0.019018351 |
| ENSG00000126243 | LRFN3 | -2.046589733 | 1.49E-19 |
| ENSG00000159733 | ZFYVE28 | -2.045415478 | 0.000231519 |
| ENSG00000243667 | WDR92 | -2.044936665 | 0.002575736 |
| ENSG00000225329 | LHFPL3-AS2 | -2.042766335 | 0.00000616 |
| ENSG00000198712 | MT-CO2 | -2.04196193 | 0.00000461 |
| ENSG00000196189 | SEMA4A | -2.040900145 | 4.64E-19 |
| ENSG00000196391 | ZNF774 | -2.040662709 | 1.13E-10 |
| ENSG00000102967 | DHODH | -2.040611938 | 6.08E-18 |
| ENSG00000267530 | LINC01836 | -2.039886622 | 0.004580432 |
| ENSG00000170439 | METTL7B | -2.039616405 | 3.4E-13 |
| ENSG00000174672 | BRSK2 | -2.037601135 | 0.012329529 |
| ENSG00000153093 | ACOXL | -2.036877951 | 0.000000247 |
| ENSG00000160298 | C21orf58 | -2.03661304 | 1.13E-16 |
| ENSG00000165730 | STOX1 | -2.036368582 | 2.62E-09 |
| ENSG00000271270 | TMCC1-AS1 | -2.031305572 | 0.0000482 |
| ENSG00000146842 | TMEM209 | -2.031082387 | 3.65E-41 |
| ENSG00000214309 | MBLAC1 | -2.03040736 | 0.001451286 |
| ENSG00000144120 | TMEM177 | -2.029096173 | 7.65E-33 |
| ENSG00000132881 | RSG1 | -2.028578624 | 1.11E-10 |
| ENSG00000260526 | AC109347.1 | -2.025216116 | 0.013307221 |
| ENSG00000232677 | LINC00665 | -2.02416888 | 0.001464625 |
| ENSG00000181634 | TNFSF15 | -2.024073472 | 9.35E-17 |
| ENSG00000129474 | AJUBA | -2.023110794 | 1.73E-30 |
| ENSG00000102678 | FGF9 | -2.022959017 | 1.19E-19 |
| ENSG00000197363 | ZNF517 | -2.022135058 | 4.66E-14 |
| ENSG00000161551 | ZNF577 | -2.021970453 | 0.000394533 |
| ENSG00000204335 | SP5 | -2.020599438 | 2.47E-15 |
| ENSG00000226314 | ZNF192P1 | -2.020421744 | 0.000822459 |
| ENSG00000224621 | AL451042.1 | -2.019986552 | 0.012371813 |
| ENSG00000175318 | GRAMD2A | -2.019409936 | 0.006450475 |
| ENSG00000101162 | TUBB1 | -2.018571404 | 0.01095819 |
| ENSG00000272760 | AC093726.1 | -2.018205391 | 0.000416959 |
| ENSG00000241388 | HNF1A-AS1 | -2.017734553 | 5.63E-08 |
| ENSG00000143167 | GPA33 | -2.01772055 | 2.87E-11 |
| ENSG00000231770 | TMEM44-AS1 | -2.01744924 | 0.000000483 |
| ENSG00000118690 | ARMC2 | -2.015773586 | 0.00000194 |
| ENSG00000129317 | PUS7L | -2.015621424 | 8.95E-33 |
| ENSG00000237943 | PRKCQ-AS1 | -2.015568849 | 0.0000046 |
| ENSG00000140104 | CLBA1 | -2.015302635 | 8.1E-19 |
| ENSG00000104883 | PEX11G | -2.015082447 | 0.000132054 |
| ENSG00000178295 | GEN1 | -2.01198731 | 3.82E-53 |
| ENSG00000189057 | FAM111B | -2.011475714 | 2.79E-19 |
| ENSG00000276340 |  | -2.010223851 | 0.0000009 |
| ENSG00000160172 | FAM86C2P | -2.009436248 | 2.57E-09 |
| ENSG00000272455 | AL391244.3 | -2.007856125 | 0.00000302 |
| ENSG00000253598 | SLC10A5 | -2.007725228 | 0.000253149 |
| ENSG00000126562 | WNK4 | -2.006957378 | 2.48E-17 |
| ENSG00000138030 | KHK | -2.006733296 | 2.19E-10 |
| ENSG00000101751 | POLI | -2.004266146 | 4.51E-15 |
| ENSG00000019186 | CYP24A1 | -2.004157866 | 1.51E-19 |
| ENSG00000103150 | MLYCD | -2.003141431 | 1.51E-31 |
| ENSG00000226479 | TMEM185B | -2.002458953 | 7.29E-43 |
| ENSG00000153233 | PTPRR | -2.00187901 | 0.0000176 |
| ENSG00000176472 | ZNF575 | -2.000905605 | 0.000655668 |
| ENSG00000129654 | FOXJ1 | -2.000528206 | 0.000311321 |
| ENSG00000255666 | AP000943.2 | -1.998260766 | 0.000668342 |
| ENSG00000152213 | ARL11 | -1.998134805 | 0.010739101 |
| ENSG00000198804 | MT-CO1 | -1.997386344 | 5.18E-08 |
| ENSG00000089091 | DZANK1 | -1.996781033 | 0.0000213 |
| ENSG00000064651 | SLC12A2 | -1.993441597 | 6.44E-24 |
| ENSG00000155666 | KDM8 | -1.992600226 | 0.00000091 |
| ENSG00000197619 | ZNF615 | -1.987754457 | 0.0037846 |
| ENSG00000143126 | CELSR2 | -1.986595911 | 6.67E-09 |
| ENSG00000081692 | JMJD4 | -1.98443819 | 6.35E-17 |
| ENSG00000128714 | HOXD13 | -1.983352977 | 0.000809764 |
| ENSG00000240602 | AADACP1 | -1.981946069 | 3.22E-08 |
| ENSG00000225830 | ERCC6 | -1.981883811 | 1.56E-18 |
| ENSG00000151065 | DCP1B | -1.980573086 | 7.19E-14 |
| ENSG00000223806 | LINC00114 | -1.97645624 | 0.038689615 |
| ENSG00000132846 | ZBED3 | -1.976433444 | 2.36E-16 |
| ENSG00000266094 | RASSF5 | -1.974282648 | 1.3E-29 |
| ENSG00000073464 | CLCN4 | -1.97238097 | 0.000240034 |
| ENSG00000176396 | EID2 | -1.972046443 | 2.35E-28 |
| ENSG00000132763 | MMACHC | -1.971426012 | 8.85E-18 |
| ENSG00000253540 | FAM86HP | -1.970625848 | 0.002117305 |
| ENSG00000172086 | KRCC1 | -1.969928162 | 8.38E-24 |
| ENSG00000248243 | LINC02014 | -1.969003422 | 0.021660471 |
| ENSG00000238278 | ALG1L6P | -1.968527739 | 0.012345953 |
| ENSG00000124459 | ZNF45 | -1.968200171 | 9.63E-40 |
| ENSG00000228889 | UBAC2-AS1 | -1.968161179 | 0.00000127 |
| ENSG00000185875 | THNSL1 | -1.966856612 | 2.18E-24 |
| ENSG00000159184 | HOXB13 | -1.965536159 | 1.11E-21 |
| ENSG00000118523 | CTGF | -1.96347811 | 0.000000974 |
| ENSG00000144061 | NPHP1 | -1.962923194 | 1.04E-09 |
| ENSG00000235257 | ITGA9-AS1 | -1.961346061 | 0.000136983 |
| ENSG00000138639 | ARHGAP24 | -1.959341019 | 0.000906096 |
| ENSG00000121440 | PDZRN3 | -1.959324611 | 0.000000141 |
| ENSG00000205885 | C1RL-AS1 | -1.958730412 | 0.00000337 |
| ENSG00000125872 | LRRN4 | -1.957035496 | 0.007052287 |
| ENSG00000225361 | PPP1R26-AS1 | -1.956987511 | 0.00000447 |
| ENSG00000273831 |  | -1.956744285 | 0.00000129 |
| ENSG00000244165 | P2RY11 | -1.956319948 | 0.000105478 |
| ENSG00000060642 | PIGV | -1.955558789 | 4.69E-50 |
| ENSG00000076248 | UNG | -1.955282569 | 9.52E-68 |
| ENSG00000157184 | CPT2 | -1.953464157 | 4.44E-31 |
| ENSG00000116885 | OSCP1 | -1.953313637 | 0.000000375 |
| ENSG00000176714 | CCDC121 | -1.953252675 | 2.89E-12 |
| ENSG00000257740 | AC073896.3 | -1.948714755 | 0.032662238 |
| ENSG00000121797 | CCRL2 | -1.948653232 | 9.17E-17 |
| ENSG00000187860 | CCDC157 | -1.945602041 | 0.0000028 |
| ENSG00000228544 | CCDC183-AS1 | -1.943361184 | 0.001021757 |
| ENSG00000233903 | Z83851.1 | -1.940136798 | 0.005632935 |
| ENSG00000107796 | ACTA2 | -1.939885794 | 0.032053316 |
| ENSG00000188827 | SLX4 | -1.939595795 | 1.02E-30 |
| ENSG00000168040 | FADD | -1.939431092 | 1.44E-42 |
| ENSG00000258429 | PDF | -1.938995812 | 1.51E-15 |
| ENSG00000170271 | FAXDC2 | -1.93778835 | 0.000162138 |
| ENSG00000070731 | ST6GALNAC2 | -1.937678288 | 0.00000309 |
| ENSG00000134897 | BIVM | -1.937570488 | 1.19E-14 |
| ENSG00000153896 | ZNF599 | -1.937039736 | 2.39E-09 |
| ENSG00000009765 | IYD | -1.935071129 | 0.00000602 |
| ENSG00000049239 | H6PD | -1.93480539 | 3.23E-18 |
| ENSG00000132016 | C19orf57 | -1.934126118 | 0.001813731 |
| ENSG00000228395 | AL356481.1 | -1.933683726 | 0.004090164 |
| ENSG00000254973 | AC105219.4 | -1.933017128 | 0.014566122 |
| ENSG00000272341 | AL137003.2 | -1.932274192 | 0.00000918 |
| ENSG00000111752 | PHC1 | -1.931740862 | 0.000131056 |
| ENSG00000231890 | DARS-AS1 | -1.930666828 | 0.046794855 |
| ENSG00000163501 | IHH | -1.928577637 | 3.44E-19 |
| ENSG00000237276 | ANO7L1 | -1.927548017 | 0.002123121 |
| ENSG00000011677 | GABRA3 | -1.926700081 | 0.049947162 |
| ENSG00000236008 | LINC01814 | -1.923213277 | 0.0000232 |
| ENSG00000145029 | NICN1 | -1.921197458 | 5.21E-11 |
| ENSG00000155393 | HEATR3 | -1.920459393 | 1.59E-20 |
| ENSG00000215068 | AC025171.2 | -1.91741915 | 2.91E-08 |
| ENSG00000278192 | AL118505.1 | -1.914573848 | 0.000392133 |
| ENSG00000162078 | ZG16B | -1.911015536 | 6.67E-13 |
| ENSG00000176024 | ZNF613 | -1.908541859 | 1.08E-15 |
| ENSG00000183111 | ARHGEF37 | -1.905699361 | 0.003566219 |
| ENSG00000182986 | ZNF320 | -1.904355388 | 2.11E-39 |
| ENSG00000205464 | ATP6AP1L | -1.903879842 | 0.021914516 |
| ENSG00000228613 | AC141930.1 | -1.902259494 | 0.024149739 |
| ENSG00000178935 | ZNF552 | -1.90080721 | 3.08E-35 |
| ENSG00000229334 | AC046143.1 | -1.899876866 | 0.00000108 |
| ENSG00000134461 | ANKRD16 | -1.899577193 | 4.2E-15 |
| ENSG00000172828 | CES3 | -1.899509159 | 0.00000373 |
| ENSG00000250303 | AP002884.1 | -1.898617952 | 0.031864668 |
| ENSG00000166997 | CNPY4 | -1.89708458 | 1.29E-08 |
| ENSG00000198464 | ZNF480 | -1.897043923 | 1.46E-11 |
| ENSG00000159792 | PSKH1 | -1.89695616 | 6.48E-53 |
| ENSG00000148935 | GAS2 | -1.894489224 | 0.000602291 |
| ENSG00000132801 | ZSWIM3 | -1.894372395 | 5.09E-12 |
| ENSG00000163104 | SMARCAD1 | -1.894129426 | 1.25E-53 |
| ENSG00000271119 | AC026412.3 | -1.891876381 | 0.00126774 |
| ENSG00000118849 | RARRES1 | -1.891494419 | 2.19E-10 |
| ENSG00000108312 | UBTF | -1.890399319 | 5.35E-24 |
| ENSG00000259087 | AL121790.2 | -1.890144225 | 0.0000153 |
| ENSG00000277304 | AC142086.6 | -1.889684075 | 0.005476676 |
| ENSG00000149050 | ZNF214 | -1.888918488 | 1.78E-12 |
| ENSG00000102003 | SYP | -1.884718118 | 0.04368497 |
| ENSG00000221909 | FAM200A | -1.884086817 | 1.38E-19 |
| ENSG00000137501 | SYTL2 | -1.883941635 | 0.000000304 |
| ENSG00000228630 | HOTAIR | -1.882212514 | 4.32E-18 |
| ENSG00000166073 | GPR176 | -1.881941582 | 0.000416959 |
| ENSG00000186952 | TMEM232 | -1.88076678 | 0.007771921 |
| ENSG00000002587 | HS3ST1 | -1.879632896 | 2.94E-26 |
| ENSG00000180425 | C11orf71 | -1.876931993 | 1.32E-08 |
| ENSG00000177873 | ZNF619 | -1.875700316 | 0.000000365 |
| ENSG00000168906 | MAT2A | -1.875617643 | 1.73E-21 |
| ENSG00000158483 | FAM86C1 | -1.874281574 | 8.56E-08 |
| ENSG00000277311 | RF02247 | -1.873293905 | 0.038877822 |
| ENSG00000122877 | EGR2 | -1.871170321 | 0.046377621 |
| ENSG00000163521 | GLB1L | -1.871010099 | 0.00000281 |
| ENSG00000132746 | ALDH3B2 | -1.870256465 | 0.001523851 |
| ENSG00000197191 | CYSRT1 | -1.870198379 | 0.006323598 |
| ENSG00000259802 | AC012640.2 | -1.869058778 | 0.002019104 |
| ENSG00000160183 | TMPRSS3 | -1.867375134 | 0.003967498 |
| ENSG00000113790 | EHHADH | -1.865775173 | 1.5E-09 |
| ENSG00000125510 | OPRL1 | -1.863177316 | 0.008925066 |
| ENSG00000158301 | GPRASP2 | -1.862266228 | 0.000232918 |
| ENSG00000205089 | CCNI2 | -1.862000893 | 0.000000046 |
| ENSG00000134864 | GGACT | -1.861094142 | 0.000211984 |
| ENSG00000163293 | NIPAL1 | -1.859810128 | 3.9E-39 |
| ENSG00000143412 | ANXA9 | -1.859474659 | 5.03E-16 |
| ENSG00000113494 | PRLR | -1.857956561 | 0.00000137 |
| ENSG00000125257 | ABCC4 | -1.857439477 | 6.55E-18 |
| ENSG00000180066 | C10orf91 | -1.856931127 | 0.023388366 |
| ENSG00000119574 | ZBTB45 | -1.856659373 | 7.51E-40 |
| ENSG00000184616 | SPDYE12P | -1.856521966 | 0.012992912 |
| ENSG00000147912 | FBXO10 | -1.85611086 | 0.000353279 |
| ENSG00000176108 | CHMP6 | -1.855478984 | 2.24E-14 |
| ENSG00000170604 | IRF2BP1 | -1.854273231 | 6.22E-53 |
| ENSG00000274853 |  | -1.851105742 | 0.011786541 |
| ENSG00000177283 | FZD8 | -1.850531187 | 0.005112059 |
| ENSG00000173269 | MMRN2 | -1.848476102 | 6.04E-20 |
| ENSG00000137877 | SPTBN5 | -1.846341712 | 0.000176763 |
| ENSG00000254290 | AC124067.4 | -1.844588211 | 8E-17 |
| ENSG00000224259 | LINC01133 | -1.843879688 | 0.001507061 |
| ENSG00000131089 | ARHGEF9 | -1.840759443 | 1.2E-14 |
| ENSG00000166387 | PPFIBP2 | -1.840043109 | 1.19E-28 |
| ENSG00000116151 | MORN1 | -1.840025173 | 3.44E-15 |
| ENSG00000184898 | RBM43 | -1.838516164 | 0.00000119 |
| ENSG00000268001 | CARD8-AS1 | -1.83769104 | 0.000330146 |
| ENSG00000213693 | SEC14L1P1 | -1.837142997 | 0.017755736 |
| ENSG00000124120 | TTPAL | -1.835481533 | 4.46E-14 |
| ENSG00000173546 | CSPG4 | -1.83526005 | 0.00012641 |
| ENSG00000106991 | ENG | -1.834515404 | 8.94E-17 |
| ENSG00000196177 | ACADSB | -1.834243277 | 3.39E-12 |
| ENSG00000221990 | EXOC3-AS1 | -1.832230095 | 1.41E-08 |
| ENSG00000196405 | EVL | -1.831640634 | 0.000000501 |
| ENSG00000213963 | AC019080.1 | -1.831544015 | 0.012827424 |
| ENSG00000213380 | COG8 | -1.831482323 | 5.95E-25 |
| ENSG00000136883 | KIF12 | -1.82632784 | 2.32E-17 |
| ENSG00000150893 | FREM2 | -1.825907239 | 2.01E-12 |
| ENSG00000079257 | LXN | -1.825401684 | 9.15E-16 |
| ENSG00000273656 |  | -1.824354293 | 0.002781457 |
| ENSG00000144306 | SCRN3 | -1.824296136 | 8.87E-20 |
| ENSG00000144026 | ZNF514 | -1.823240369 | 1.09E-11 |
| ENSG00000197959 | DNM3 | -1.822353224 | 0.000225349 |
| ENSG00000196741 | LINC01560 | -1.8215163 | 0.0000562 |
| ENSG00000116138 | DNAJC16 | -1.819317021 | 7.88E-33 |
| ENSG00000011143 | MKS1 | -1.818960397 | 2.49E-25 |
| ENSG00000271646 | AC099343.3 | -1.816811708 | 0.004899323 |
| ENSG00000225210 | DUXAP9 | -1.812415582 | 0.001077136 |
| ENSG00000123407 | HOXC12 | -1.81087859 | 0.034370906 |
| ENSG00000155592 | ZKSCAN2 | -1.81031948 | 4.02E-11 |
| ENSG00000127720 | METTL25 | -1.810137803 | 0.0000865 |
| ENSG00000205436 | EXOC3L4 | -1.810090497 | 0.000240116 |
| ENSG00000111077 | TNS2 | -1.809830292 | 5.92E-22 |
| ENSG00000185332 | TMEM105 | -1.807473577 | 0.000000464 |
| ENSG00000254635 | WAC-AS1 | -1.805807462 | 1.32E-13 |
| ENSG00000256594 | AC010186.2 | -1.804181623 | 0.01266752 |
| ENSG00000148225 | WDR31 | -1.801294565 | 1.36E-10 |
| ENSG00000172508 | CARNS1 | -1.80129004 | 0.001247309 |
| ENSG00000134398 | ERN2 | -1.799155857 | 2.31E-11 |
| ENSG00000165556 | CDX2 | -1.797977558 | 1.28E-12 |
| ENSG00000104537 | ANXA13 | -1.795943841 | 1.37E-25 |
| ENSG00000236088 | COX10-AS1 | -1.794130666 | 0.000000859 |
| ENSG00000274803 |  | -1.793948086 | 0.006896079 |
| ENSG00000164855 | TMEM184A | -1.79377313 | 1.09E-14 |
| ENSG00000068654 | POLR1A | -1.793577778 | 4.43E-13 |
| ENSG00000114547 | ROPN1B | -1.793533289 | 0.000162241 |
| ENSG00000115841 | RMDN2 | -1.792784023 | 5.7E-11 |
| ENSG00000279519 | AC007382.1 | -1.792542387 | 0.001250237 |
| ENSG00000136319 | TTC5 | -1.791278109 | 6.95E-13 |
| ENSG00000188825 | LINC00910 | -1.791245453 | 0.000000607 |
| ENSG00000128284 | APOL3 | -1.790968575 | 0.002889758 |
| ENSG00000103494 | RPGRIP1L | -1.788175184 | 2.25E-13 |
| ENSG00000268584 | AC073389.1 | -1.786517968 | 0.041562626 |
| ENSG00000149548 | CCDC15 | -1.784337577 | 5.12E-08 |
| ENSG00000132329 | RAMP1 | -1.783881288 | 0.0000691 |
| ENSG00000173918 | C1QTNF1 | -1.779739128 | 0.00000242 |
| ENSG00000271737 | AC008608.2 | -1.779428568 | 0.002712614 |
| ENSG00000203635 | AC144450.1 | -1.776702324 | 0.017176323 |
| ENSG00000037897 | METTL1 | -1.776697987 | 3.24E-15 |
| ENSG00000196366 | C9orf163 | -1.775033899 | 0.003692571 |
| ENSG00000123388 | HOXC11 | -1.772471492 | 0.034414175 |
| ENSG00000107614 | TRDMT1 | -1.770307341 | 1.98E-10 |
| ENSG00000279253 | AL121753.2 | -1.768630175 | 0.000166425 |
| ENSG00000108813 | DLX4 | -1.767630356 | 0.00966267 |
| ENSG00000184828 | ZBTB7C | -1.767412662 | 2.13E-17 |
| ENSG00000228672 | PROB1 | -1.767050909 | 6.47E-15 |
| ENSG00000154957 | ZNF18 | -1.766725346 | 0.00000563 |
| ENSG00000089847 | ANKRD24 | -1.765809644 | 0.014781786 |
| ENSG00000135775 | COG2 | -1.765300396 | 6.2E-57 |
| ENSG00000270964 | AC016355.1 | -1.764758708 | 0.003141716 |
| ENSG00000135622 | SEMA4F | -1.763840994 | 4.92E-08 |
| ENSG00000166349 | RAG1 | -1.762799706 | 0.032369772 |
| ENSG00000178075 | GRAMD1C | -1.761626308 | 2.61E-09 |
| ENSG00000132205 | EMILIN2 | -1.761444282 | 2.79E-16 |
| ENSG00000214425 | LRRC37A4P | -1.761299485 | 0.028027172 |
| ENSG00000259439 | LINC01833 | -1.760572716 | 4.67E-17 |
| ENSG00000138613 | APH1B | -1.758460888 | 2.01E-13 |
| ENSG00000088035 | ALG6 | -1.758294782 | 3.94E-20 |
| ENSG00000176273 | SLC35G1 | -1.752289102 | 2.64E-18 |
| ENSG00000166086 | JAM3 | -1.751016213 | 0.000235551 |
| ENSG00000011258 | MBTD1 | -1.750867606 | 3E-22 |
| ENSG00000124006 | OBSL1 | -1.750423396 | 0.049848305 |
| ENSG00000135315 | CEP162 | -1.750197767 | 9.61E-09 |
| ENSG00000239789 | MRPS17 | -1.748537242 | 2.32E-14 |
| ENSG00000145882 | PCYOX1L | -1.748403335 | 3.46E-08 |
| ENSG00000234494 | SP2-AS1 | -1.748194055 | 0.000407962 |
| ENSG00000163482 | STK36 | -1.747816703 | 6.95E-13 |
| ENSG00000198131 | ZNF544 | -1.747685759 | 2.05E-27 |
| ENSG00000160051 | IQCC | -1.74715981 | 1.92E-09 |
| ENSG00000215105 | TTC3P1 | -1.746780398 | 0.000000422 |
| ENSG00000162639 | HENMT1 | -1.744544382 | 1.76E-27 |
| ENSG00000235106 | BRD3OS | -1.744500699 | 2.67E-17 |
| ENSG00000255145 | STX17-AS1 | -1.743803731 | 0.046454315 |
| ENSG00000221817 | PPP3CB-AS1 | -1.743202002 | 0.000194984 |
| ENSG00000242110 | AMACR | -1.741855359 | 0.0000637 |
| ENSG00000164053 | ATRIP | -1.74177512 | 1.21E-11 |
| ENSG00000196922 | ZNF252P | -1.741118836 | 4.06E-29 |
| ENSG00000176896 | TCEANC | -1.741050989 | 0.001413598 |
| ENSG00000184384 | MAML2 | -1.740129372 | 1.04E-14 |
| ENSG00000236859 | NIFK-AS1 | -1.738617551 | 1.59E-10 |
| ENSG00000102796 | DHRS12 | -1.737241988 | 1.27E-10 |
| ENSG00000261485 | PAN3-AS1 | -1.736123069 | 0.007127472 |
| ENSG00000177613 | CSTF2T | -1.736099104 | 1.05E-33 |
| ENSG00000224093 | AL109613.1 | -1.735729299 | 0.000000833 |
| ENSG00000128694 | OSGEPL1 | -1.735720896 | 5.7E-12 |
| ENSG00000185127 | C6orf120 | -1.734939791 | 3.68E-37 |
| ENSG00000187650 | VMAC | -1.733933473 | 0.00000193 |
| ENSG00000126217 | MCF2L | -1.733143245 | 6.47E-17 |
| ENSG00000184857 | TMEM186 | -1.732945583 | 1.82E-23 |
| ENSG00000107738 | VSIR | -1.732737293 | 1.74E-13 |
| ENSG00000133466 | C1QTNF6 | -1.732100672 | 4.97E-19 |
| ENSG00000189339 | SLC35E2B | -1.731235634 | 8.54E-28 |
| ENSG00000169683 | LRRC45 | -1.731156258 | 9.16E-40 |
| ENSG00000187240 | DYNC2H1 | -1.73011194 | 0.007236048 |
| ENSG00000183161 | FANCF | -1.72969617 | 5.92E-29 |
| ENSG00000226688 | ENTPD1-AS1 | -1.729489948 | 0.003922408 |
| ENSG00000132031 | MATN3 | -1.728966742 | 0.007676954 |
| ENSG00000256806 | C17orf100 | -1.7267674 | 8.25E-08 |
| ENSG00000164930 | FZD6 | -1.725415496 | 2.76E-37 |
| ENSG00000174306 | ZHX3 | -1.723844805 | 8.91E-17 |
| ENSG00000145416 | 44256 | -1.723588798 | 0.000000384 |
| ENSG00000054219 | LY75 | -1.723060515 | 1.89E-10 |
| ENSG00000251595 | ABCA11P | -1.7228016 | 0.005478807 |
| ENSG00000135482 | ZC3H10 | -1.722417055 | 2.54E-11 |
| ENSG00000134575 | ACP2 | -1.721967597 | 3.17E-18 |
| ENSG00000173175 | ADCY5 | -1.721684013 | 0.000516499 |
| ENSG00000179841 | AKAP5 | -1.721593447 | 0.000320741 |
| ENSG00000185303 | SFTPA2 | -1.718081013 | 0.00571971 |
| ENSG00000103599 | IQCH | -1.717355295 | 0.0000678 |
| ENSG00000250132 | AC004803.1 | -1.717245004 | 0.000523293 |
| ENSG00000115318 | LOXL3 | -1.717139813 | 0.013479802 |
| ENSG00000101311 | FERMT1 | -1.716472575 | 2.42E-31 |
| ENSG00000145217 | SLC26A1 | -1.716198548 | 0.00000103 |
| ENSG00000152582 | SPEF2 | -1.716196471 | 0.00000311 |
| ENSG00000148832 | PAOX | -1.715800646 | 2.08E-08 |
| ENSG00000261167 | AC107027.3 | -1.714375322 | 0.003643899 |
| ENSG00000072195 | SPEG | -1.713824561 | 0.00000054 |
| ENSG00000119227 | PIGZ | -1.712916502 | 2.14E-12 |
| ENSG00000176920 | FUT2 | -1.712575123 | 6.94E-23 |
| ENSG00000280202 | AC005831.1 | -1.710813174 | 6.89E-10 |
| ENSG00000158882 | TOMM40L | -1.709778886 | 1.25E-08 |
| ENSG00000178462 | TUBAL3 | -1.709668005 | 0.03936668 |
| ENSG00000171448 | ZBTB26 | -1.709373634 | 4.16E-14 |
| ENSG00000154743 | TSEN2 | -1.708887622 | 4.79E-13 |
| ENSG00000126773 | PCNX4 | -1.708429267 | 3.29E-24 |
| ENSG00000204104 | TRAF3IP1 | -1.708028343 | 1.51E-15 |
| ENSG00000176410 | DNAJC30 | -1.707807127 | 1.84E-11 |
| ENSG00000167972 | ABCA3 | -1.705914167 | 0.003236742 |
| ENSG00000111877 | MCM9 | -1.703382461 | 9.8E-18 |
| ENSG00000118322 | ATP10B | -1.702476468 | 1.22E-16 |
| ENSG00000196155 | PLEKHG4 | -1.702466984 | 1.01E-13 |
| ENSG00000013503 | POLR3B | -1.701148397 | 2.84E-14 |
| ENSG00000132122 | SPATA6 | -1.699800248 | 0.00000257 |
| ENSG00000188739 | RBM34 | -1.698489539 | 0.020034264 |
| ENSG00000186687 | LYRM7 | -1.697414434 | 9.05E-23 |
| ENSG00000134297 | PLEKHA8P1 | -1.696611098 | 8.4E-12 |
| ENSG00000113966 | ARL6 | -1.696528122 | 3.78E-17 |
| ENSG00000158125 | XDH | -1.696525403 | 0.000190882 |
| ENSG00000272086 | AC025181.2 | -1.695759491 | 0.00000102 |
| ENSG00000138131 | LOXL4 | -1.695473718 | 0.001418405 |
| ENSG00000159239 | AC005041.1 | -1.694289271 | 5.02E-10 |
| ENSG00000172382 | PRSS27 | -1.691237122 | 0.032381758 |
| ENSG00000115392 | FANCL | -1.689877423 | 4.2E-28 |
| ENSG00000259523 | AC022613.2 | -1.689830664 | 0.03182467 |
| ENSG00000251095 | AC097478.1 | -1.689784494 | 0.003477519 |
| ENSG00000275544 |  | -1.689732301 | 0.008264958 |
| ENSG00000228952 | LINC02041 | -1.688648028 | 0.046212631 |
| ENSG00000196663 | TECPR2 | -1.688005081 | 5.52E-13 |
| ENSG00000130749 | ZC3H4 | -1.686182494 | 3.77E-23 |
| ENSG00000244026 | FAM86DP | -1.686157772 | 9.19E-11 |
| ENSG00000229043 | AC091729.3 | -1.685681427 | 0.0000599 |
| ENSG00000276048 | RF02250 | -1.685058942 | 0.047621524 |
| ENSG00000134138 | MEIS2 | -1.684694052 | 1.56E-22 |
| ENSG00000105251 | SHD | -1.684510257 | 0.04873105 |
| ENSG00000261115 | TMEM178B | -1.684160241 | 2.05E-11 |
| ENSG00000100271 | TTLL1 | -1.683465278 | 0.000000413 |
| ENSG00000177788 | AL162595.1 | -1.680660998 | 0.005028525 |
| ENSG00000198556 | ZNF789 | -1.68022843 | 1.05E-15 |
| ENSG00000107485 | GATA3 | -1.680024971 | 0.0000571 |
| ENSG00000263843 | AC022211.2 | -1.678997076 | 0.001191228 |
| ENSG00000187713 | TMEM203 | -1.677603593 | 1.18E-31 |
| ENSG00000166262 | FAM227B | -1.67717101 | 0.001916595 |
| ENSG00000214290 | COLCA2 | -1.677029186 | 1.95E-21 |
| ENSG00000166188 | ZNF319 | -1.676446828 | 1.12E-09 |
| ENSG00000173581 | CCDC106 | -1.676210459 | 2.54E-10 |
| ENSG00000162415 | ZSWIM5 | -1.675060456 | 0.0000153 |
| ENSG00000273820 | USP27X | -1.675014987 | 0.00000136 |
| ENSG00000151164 | RAD9B | -1.674700854 | 0.022300444 |
| ENSG00000169989 | TIGD4 | -1.674294037 | 0.022278558 |
| ENSG00000089050 | RBBP9 | -1.674282251 | 2.9E-19 |
| ENSG00000074181 | NOTCH3 | -1.674010715 | 0.013974752 |
| ENSG00000013297 | CLDN11 | -1.673766243 | 0.014977066 |
| ENSG00000188878 | FBF1 | -1.673726143 | 0.016522547 |
| ENSG00000245573 | BDNF-AS | -1.672397386 | 0.000579366 |
| ENSG00000188242 | AC010442.1 | -1.671647707 | 8.59E-32 |
| ENSG00000131398 | KCNC3 | -1.67098161 | 0.0000121 |
| ENSG00000183773 | AIFM3 | -1.668325096 | 2.3E-13 |
| ENSG00000131779 | PEX11B | -1.667885617 | 4.82E-29 |
| ENSG00000125434 | SLC25A35 | -1.667808481 | 0.00000566 |
| ENSG00000160602 | NEK8 | -1.666874179 | 1.94E-11 |
| ENSG00000186523 | FAM86B1 | -1.666607826 | 0.000226879 |
| ENSG00000174502 | SLC26A9 | -1.66660083 | 0.0000407 |
| ENSG00000174276 | ZNHIT2 | -1.665615341 | 1.25E-23 |
| ENSG00000184860 | SDR42E1 | -1.665152673 | 3.15E-24 |
| ENSG00000281376 | ABALON | -1.662271866 | 0.00000456 |
| ENSG00000155729 | KCTD18 | -1.661757072 | 3.84E-15 |
| ENSG00000078967 | UBE2D4 | -1.661713462 | 1.15E-11 |
| ENSG00000125843 | AP5S1 | -1.659685427 | 1.4E-18 |
| ENSG00000130349 | C6orf203 | -1.659489848 | 1.17E-20 |
| ENSG00000198695 | MT-ND6 | -1.659068831 | 0.000879015 |
| ENSG00000261098 | AP000766.1 | -1.658238806 | 0.041540464 |
| ENSG00000182150 | ERCC6L2 | -1.657686321 | 1.85E-13 |
| ENSG00000267106 | ZNF561-AS1 | -1.657631693 | 0.000175565 |
| ENSG00000151364 | KCTD14 | -1.657494186 | 0.0000111 |
| ENSG00000279508 |  | -1.656937223 | 0.0000428 |
| ENSG00000132793 | LPIN3 | -1.656518928 | 1.72E-11 |
| ENSG00000236144 | TMEM147-AS1 | -1.656480254 | 4.41E-08 |
| ENSG00000155792 | DEPTOR | -1.655391983 | 8.37E-12 |
| ENSG00000197566 | ZNF624 | -1.653065031 | 0.000263139 |
| ENSG00000159784 | FAM131B | -1.652109291 | 9.81E-21 |
| ENSG00000241127 | YAE1D1 | -1.651967104 | 1.01E-18 |
| ENSG00000257800 | FNBP1P1 | -1.651177284 | 0.000398525 |
| ENSG00000057704 | TMCC3 | -1.649578482 | 5.98E-13 |
| ENSG00000055147 | FAM114A2 | -1.64888086 | 2.85E-20 |
| ENSG00000282386 | AL358472.4 | -1.648822561 | 0.007287872 |
| ENSG00000172794 | RAB37 | -1.648691074 | 0.000365275 |
| ENSG00000188033 | ZNF490 | -1.64793707 | 0.001410917 |
| ENSG00000256628 | ZBTB11-AS1 | -1.647243582 | 1.72E-08 |
| ENSG00000180745 | CLRN3 | -1.646499028 | 5E-19 |
| ENSG00000256967 | AC018653.3 | -1.646058999 | 0.035866125 |
| ENSG00000162458 | FBLIM1 | -1.644091073 | 1.53E-13 |
| ENSG00000170482 | SLC23A1 | -1.644045832 | 0.003474049 |
| ENSG00000260461 | AL133355.1 | -1.643619584 | 0.007517591 |
| ENSG00000099377 | HSD3B7 | -1.64273007 | 0.000110768 |
| ENSG00000161395 | PGAP3 | -1.641680144 | 1.24E-25 |
| ENSG00000228434 | AC004951.1 | -1.640074343 | 0.033897176 |
| ENSG00000163389 | POGLUT1 | -1.639061453 | 3.33E-10 |
| ENSG00000189164 | ZNF527 | -1.638813515 | 0.00000493 |
| ENSG00000169247 | SH3TC2 | -1.638127024 | 1.79E-17 |
| ENSG00000148734 | NPFFR1 | -1.638082418 | 0.00000191 |
| ENSG00000206195 | DUXAP8 | -1.637783566 | 2.8E-10 |
| ENSG00000260136 | AC008915.2 | -1.637434742 | 0.013861017 |
| ENSG00000198690 | FAN1 | -1.637354535 | 0.00000932 |
| ENSG00000156253 | RWDD2B | -1.635376979 | 4.9E-21 |
| ENSG00000155636 | RBM45 | -1.63487268 | 0.00000023 |
| ENSG00000168517 | HEXIM2 | -1.632715842 | 0.001145561 |
| ENSG00000167968 | DNASE1L2 | -1.63271224 | 0.005718629 |
| ENSG00000178093 | TSSK6 | -1.632708243 | 0.000239272 |
| ENSG00000245680 | ZNF585B | -1.632410519 | 0.00010793 |
| ENSG00000230650 | AC140479.2 | -1.632373959 | 0.0000443 |
| ENSG00000100473 | COCH | -1.629928379 | 0.013583686 |
| ENSG00000186603 | HPDL | -1.628659991 | 5.85E-24 |
| ENSG00000063587 | ZNF275 | -1.628218254 | 1.08E-22 |
| ENSG00000095585 | BLNK | -1.628060532 | 0.00000726 |
| ENSG00000124813 | RUNX2 | -1.627604973 | 1.9E-10 |
| ENSG00000203778 | FAM229B | -1.627292693 | 0.000532527 |
| ENSG00000248712 | CCDC153 | -1.626843876 | 0.009010908 |
| ENSG00000205583 | STAG3L1 | -1.625640459 | 0.000253061 |
| ENSG00000254286 |  | -1.624981939 | 0.000272858 |
| ENSG00000197429 | IPP | -1.624933526 | 4.96E-12 |
| ENSG00000145604 | SKP2 | -1.624643195 | 9.48E-27 |
| ENSG00000225335 | AC016027.1 | -1.621741727 | 0.001229501 |
| ENSG00000198331 | HYLS1 | -1.621156818 | 2.57E-11 |
| ENSG00000260219 | AC106782.2 | -1.620834861 | 0.005188892 |
| ENSG00000183150 | GPR19 | -1.620719403 | 0.001403954 |
| ENSG00000164048 | ZNF589 | -1.618415803 | 2.96E-14 |
| ENSG00000179941 | BBS10 | -1.618097236 | 3.9E-23 |
| ENSG00000225265 | TAF1A-AS1 | -1.617226346 | 0.001809637 |
| ENSG00000218510 | LINC00339 | -1.616851103 | 1.88E-16 |
| ENSG00000168427 | KLHL30 | -1.616416167 | 0.00000121 |
| ENSG00000166548 | TK2 | -1.614993028 | 2.7E-18 |
| ENSG00000185010 | F8 | -1.612743891 | 0.000797008 |
| ENSG00000176422 | SPRYD4 | -1.609906308 | 2.53E-20 |
| ENSG00000243479 | MNX1-AS1 | -1.609675864 | 7.78E-16 |
| ENSG00000103021 | CCDC113 | -1.609099201 | 0.0000168 |
| ENSG00000177694 | NAALADL2 | -1.608857581 | 1.39E-09 |
| ENSG00000008277 | ADAM22 | -1.608751043 | 3.47E-16 |
| ENSG00000169432 | SCN9A | -1.608525483 | 0.036087058 |
| ENSG00000148219 | ASTN2 | -1.608493127 | 0.00000154 |
| ENSG00000178927 | C17orf62 | -1.607824208 | 9.39E-32 |
| ENSG00000260220 | CCDC187 | -1.606993857 | 0.003700001 |
| ENSG00000095303 | PTGS1 | -1.606898628 | 2.85E-14 |
| ENSG00000145675 | PIK3R1 | -1.60551208 | 8.77E-16 |
| ENSG00000164187 | LMBRD2 | -1.604408054 | 1.08E-20 |
| ENSG00000187796 | CARD9 | -1.601578321 | 1.18E-12 |
| ENSG00000178386 | ZNF223 | -1.601358714 | 0.014468877 |
| ENSG00000162694 | EXTL2 | -1.601057711 | 1.83E-28 |
| ENSG00000164171 | ITGA2 | -1.599841193 | 1.43E-16 |
| ENSG00000164674 | SYTL3 | -1.599755558 | 0.000529945 |
| ENSG00000221916 | C19orf73 | -1.598990946 | 0.000435984 |
| ENSG00000198829 | SUCNR1 | -1.598811276 | 0.00189849 |
| ENSG00000128191 | DGCR8 | -1.598382282 | 2.41E-33 |
| ENSG00000277203 | F8A1 | -1.596933011 | 1.19E-15 |
| ENSG00000157578 | LCA5L | -1.595752349 | 0.009634467 |
| ENSG00000232098 | AC012313.1 | -1.594772548 | 5.03E-08 |
| ENSG00000136059 | VILL | -1.594238978 | 0.0000976 |
| ENSG00000148057 | IDNK | -1.593739514 | 0.00052619 |
| ENSG00000135407 | AVIL | -1.592613234 | 0.00245188 |
| ENSG00000162396 | PARS2 | -1.591622207 | 1.94E-17 |
| ENSG00000167720 | SRR | -1.591394446 | 0.00000389 |
| ENSG00000139437 | TCHP | -1.591175824 | 3.25E-22 |
| ENSG00000177096 | FAM109B | -1.590452515 | 3.86E-12 |
| ENSG00000135773 | CAPN9 | -1.589176682 | 0.000464878 |
| ENSG00000261572 | AC097639.1 | -1.587939935 | 0.000105793 |
| ENSG00000083817 | ZNF416 | -1.587897688 | 3.66E-13 |
| ENSG00000189377 | CXCL17 | -1.58636102 | 0.001012316 |
| ENSG00000163946 | FAM208A | -1.585984214 | 4.11E-30 |
| ENSG00000164294 | GPX8 | -1.585469942 | 2.04E-09 |
| ENSG00000149571 | KIRREL3 | -1.583535709 | 0.018014819 |
| ENSG00000165923 | AGBL2 | -1.583464227 | 0.032999522 |
| ENSG00000105523 | FAM83E | -1.583256079 | 1.63E-10 |
| ENSG00000120915 | EPHX2 | -1.582585286 | 0.00000103 |
| ENSG00000185267 | CDNF | -1.58227174 | 0.004810461 |
| ENSG00000229180 | AC006001.3 | -1.58210937 | 0.000473172 |
| ENSG00000010610 | CD4 | -1.582046796 | 0.02605903 |
| ENSG00000131848 | ZSCAN5A | -1.580326054 | 0.000000524 |
| ENSG00000213793 | ZNF888 | -1.580174717 | 0.0000122 |
| ENSG00000160703 | NLRX1 | -1.579428041 | 5.72E-25 |
| ENSG00000198794 | SCAMP5 | -1.578363275 | 0.003431948 |
| ENSG00000189144 | ZNF573 | -1.577785033 | 0.007597072 |
| ENSG00000135093 | USP30 | -1.577553615 | 3.58E-22 |
| ENSG00000112167 | SAYSD1 | -1.577282323 | 1.88E-20 |
| ENSG00000137124 | ALDH1B1 | -1.576649218 | 2.76E-21 |
| ENSG00000216895 | AC009403.1 | -1.576598049 | 0.0000167 |
| ENSG00000180316 | PNPLA1 | -1.574641071 | 0.015457809 |
| ENSG00000262049 | AC139530.1 | -1.573620175 | 0.00005 |
| ENSG00000203772 | SPRN | -1.572109188 | 0.000000101 |
| ENSG00000278993 | AC002350.1 | -1.571772095 | 0.010447069 |
| ENSG00000164684 | ZNF704 | -1.571699828 | 6.21E-12 |
| ENSG00000118762 | PKD2 | -1.571454482 | 1.48E-10 |
| ENSG00000106351 | AGFG2 | -1.568619658 | 1.3E-29 |
| ENSG00000100023 | PPIL2 | -1.568197966 | 2.34E-25 |
| ENSG00000069712 |  | -1.567690477 | 3.72E-09 |
| ENSG00000101417 | PXMP4 | -1.565956919 | 5.84E-16 |
| ENSG00000183323 | CCDC125 | -1.56361974 | 3.52E-11 |
| ENSG00000178409 | BEND3 | -1.563462677 | 9.39E-13 |
| ENSG00000246898 | LINC00920 | -1.563149093 | 3.46E-09 |
| ENSG00000263002 | ZNF234 | -1.562738831 | 7.18E-13 |
| ENSG00000161798 | AQP5 | -1.560574589 | 1.98E-15 |
| ENSG00000197013 | ZNF429 | -1.560358154 | 0.000646051 |
| ENSG00000178814 | OPLAH | -1.558604258 | 0.000000332 |
| ENSG00000170915 | PAQR8 | -1.557649035 | 1.61E-18 |
| ENSG00000242407 | AC091179.1 | -1.557167251 | 0.010357241 |
| ENSG00000138459 | SLC35A5 | -1.556301728 | 1.16E-27 |
| ENSG00000164081 | TEX264 | -1.554130736 | 6.54E-19 |
| ENSG00000150764 | DIXDC1 | -1.552664902 | 1.03E-10 |
| ENSG00000224870 | AL391244.1 | -1.552165971 | 7.94E-25 |
| ENSG00000180035 | ZNF48 | -1.552081887 | 9.53E-16 |
| ENSG00000213958 | KRT18P29 | -1.551919783 | 0.02541839 |
| ENSG00000152683 | SLC30A6 | -1.551812131 | 3.28E-49 |
| ENSG00000151338 | MIPOL1 | -1.551269353 | 0.00000211 |
| ENSG00000279314 | AC002525.1 | -1.550879407 | 0.001184031 |
| ENSG00000158234 | FAIM | -1.550835712 | 0.00000267 |
| ENSG00000238045 | AC009133.1 | -1.550471588 | 2.91E-09 |
| ENSG00000215790 | SLC35E2 | -1.549172459 | 0.00000187 |
| ENSG00000229719 | MIR194-2HG | -1.549084167 | 0.011192796 |
| ENSG00000253882 | AC099548.2 | -1.54798666 | 0.005005947 |
| ENSG00000151117 | TMEM86A | -1.54780227 | 0.00000246 |
| ENSG00000156853 | ZNF689 | -1.547746419 | 3.91E-18 |
| ENSG00000175782 | SLC35E3 | -1.547634676 | 2.92E-14 |
| ENSG00000118596 | SLC16A7 | -1.547415856 | 5.19E-18 |
| ENSG00000179922 | ZNF784 | -1.545945274 | 1.86E-17 |
| ENSG00000181830 | SLC35C1 | -1.545562049 | 6.89E-52 |
| ENSG00000047457 | CP | -1.545193812 | 0.021449224 |
| ENSG00000137275 | RIPK1 | -1.544628788 | 8.57E-34 |
| ENSG00000236809 | SNX25P1 | -1.544394012 | 0.017657614 |
| ENSG00000145103 | ILDR1 | -1.543899713 | 1.02E-14 |
| ENSG00000173013 | CCDC96 | -1.543829696 | 0.003846429 |
| ENSG00000104427 | ZC2HC1A | -1.537104129 | 0.000698896 |
| ENSG00000170473 | PYM1 | -1.5350329 | 6.45E-35 |
| ENSG00000214960 | ISPD | -1.532985948 | 0.001649872 |
| ENSG00000132326 | PER2 | -1.532489159 | 2.79E-11 |
| ENSG00000151876 | FBXO4 | -1.531620735 | 1.29E-12 |
| ENSG00000237973 | MTCO1P12 | -1.530732257 | 0.044871985 |
| ENSG00000167637 | ZNF283 | -1.530459211 | 1.34E-16 |
| ENSG00000258839 | MC1R | -1.529932516 | 0.0000148 |
| ENSG00000230555 | AL450326.1 | -1.529352663 | 0.007692831 |
| ENSG00000114520 | SNX4 | -1.529216101 | 2.25E-37 |
| ENSG00000238227 | TMEM250 | -1.528993959 | 4.06E-39 |
| ENSG00000114656 | KIAA1257 | -1.527533294 | 0.007068178 |
| ENSG00000167536 | DHRS13 | -1.526942666 | 1.53E-10 |
| ENSG00000166938 | DIS3L | -1.526203217 | 5.64E-32 |
| ENSG00000125124 | BBS2 | -1.525775722 | 1.52E-36 |
| ENSG00000253636 | AC022893.1 | -1.524153835 | 0.046369922 |
| ENSG00000250899 | AC125807.2 | -1.523911248 | 8.54E-22 |
| ENSG00000276791 | AC092117.1 | -1.523681255 | 0.027183857 |
| ENSG00000203880 | PCMTD2 | -1.523036689 | 2.18E-11 |
| ENSG00000214106 | PAXIP1-AS2 | -1.522519742 | 0.00000542 |
| ENSG00000276910 |  | -1.522368972 | 0.004820588 |
| ENSG00000177842 | ZNF620 | -1.522258695 | 6.71E-11 |
| ENSG00000281614 |  | -1.522136448 | 0.01158899 |
| ENSG00000149243 | KLHL35 | -1.521781022 | 6.68E-14 |
| ENSG00000214013 | GANC | -1.521674428 | 1.6E-14 |
| ENSG00000141655 | TNFRSF11A | -1.521557534 | 5.17E-16 |
| ENSG00000226137 | BAIAP2-AS1 | -1.521535437 | 2.91E-17 |
| ENSG00000133111 | RFXAP | -1.521389054 | 6.51E-12 |
| ENSG00000138185 | ENTPD1 | -1.521375024 | 1.86E-09 |
| ENSG00000149634 | SPATA25 | -1.520739988 | 0.011659995 |
| ENSG00000143409 | MINDY1 | -1.519961466 | 1.6E-17 |
| ENSG00000189366 | ALG1L | -1.519227702 | 3.04E-10 |
| ENSG00000279117 | AP001972.5 | -1.519124485 | 5.18E-11 |
| ENSG00000022976 | ZNF839 | -1.518085 | 0.000199069 |
| ENSG00000135324 | MRAP2 | -1.517997952 | 1.38E-15 |
| ENSG00000120738 | EGR1 | -1.517547198 | 0.0000421 |
| ENSG00000118997 | DNAH7 | -1.517457302 | 0.003591089 |
| ENSG00000162194 | LBHD1 | -1.517296091 | 0.000105869 |
| ENSG00000261338 | AC021016.2 | -1.516845306 | 0.010719937 |
| ENSG00000167112 | TRUB2 | -1.5167108 | 4.31E-34 |
| ENSG00000253106 | AC090198.1 | -1.516576297 | 0.001482614 |
| ENSG00000235217 | TSPY26P | -1.516430252 | 0.036541005 |
| ENSG00000155254 | MARVELD1 | -1.515894702 | 1.96E-17 |
| ENSG00000010704 | HFE | -1.515872096 | 3.02E-26 |
| ENSG00000134982 | APC | -1.51445179 | 7.09E-21 |
| ENSG00000160469 | BRSK1 | -1.514171084 | 0.03713537 |
| ENSG00000108733 | PEX12 | -1.513314849 | 6.22E-16 |
| ENSG00000100350 | FOXRED2 | -1.512964632 | 4.23E-23 |
| ENSG00000149292 | TTC12 | -1.512322315 | 3.73E-08 |
| ENSG00000260781 | ARHGAP23P1 | -1.511910068 | 0.0000324 |
| ENSG00000274627 |  | -1.511657231 | 0.01467744 |
| ENSG00000101695 | RNF125 | -1.511527524 | 5.23E-13 |
| ENSG00000278864 | AC055811.4 | -1.510917913 | 0.025987242 |
| ENSG00000273145 | BX537318.1 | -1.510460736 | 0.011581094 |
| ENSG00000183578 | TNFAIP8L3 | -1.508815915 | 0.0000133 |
| ENSG00000164989 | CCDC171 | -1.506083398 | 0.000312514 |
| ENSG00000180346 | TIGD2 | -1.503728944 | 1.35E-23 |
| ENSG00000169562 | GJB1 | -1.503491959 | 7.22E-43 |
| ENSG00000224511 | LINC00365 | -1.502592185 | 0.0000641 |
| ENSG00000124613 | ZNF391 | -1.502560244 | 0.036686305 |
| ENSG00000151062 | CACNA2D4 | -1.502051528 | 0.018277805 |
| ENSG00000234420 | ZNF37BP | -1.501167084 | 2.11E-10 |
| ENSG00000123096 | SSPN | -1.500890671 | 0.000173918 |
| ENSG00000198246 | SLC29A3 | -1.499891814 | 1.82E-20 |
| ENSG00000214300 | SPDYE3 | -1.499879277 | 3.34E-10 |
| ENSG00000175147 | TMEM51-AS1 | -1.499569171 | 0.018875086 |
| ENSG00000070985 | TRPM5 | -1.498625522 | 0.000852197 |
| ENSG00000130544 | ZNF557 | -1.497637747 | 3.32E-11 |
| ENSG00000007062 | PROM1 | -1.496584179 | 4.13E-31 |
| ENSG00000236499 | LINC00896 | -1.495535183 | 0.007520358 |
| ENSG00000067221 | STOML1 | -1.494610317 | 2.1E-12 |
| ENSG00000175787 | ZNF169 | -1.492756067 | 2.04E-10 |
| ENSG00000197180 | AC244090.1 | -1.489986273 | 0.00000576 |
| ENSG00000263528 | IKBKE | -1.489544833 | 0.000000163 |
| ENSG00000003989 | SLC7A2 | -1.488800168 | 4.65E-09 |
| ENSG00000232973 | CYP1B1-AS1 | -1.487383139 | 0.00000684 |
| ENSG00000234899 | SOX9-AS1 | -1.487300346 | 0.006229511 |
| ENSG00000178971 | CTC1 | -1.487190711 | 7.74E-12 |
| ENSG00000175170 | FAM182B | -1.486948915 | 0.038728451 |
| ENSG00000138375 | SMARCAL1 | -1.485106005 | 2.32E-16 |
| ENSG00000104290 | FZD3 | -1.483555687 | 0.000468333 |
| ENSG00000149639 | SOGA1 | -1.481931435 | 7.46E-08 |
| ENSG00000183840 | GPR39 | -1.481607956 | 3.46E-15 |
| ENSG00000140543 | DET1 | -1.479837222 | 7.55E-24 |
| ENSG00000067533 | RRP15 | -1.479798447 | 1.75E-09 |
| ENSG00000163312 | HELQ | -1.479069538 | 2.25E-13 |
| ENSG00000163704 | PRRT3 | -1.478141592 | 0.026159698 |
| ENSG00000174796 | THAP6 | -1.477808205 | 5.15E-23 |
| ENSG00000197362 | ZNF786 | -1.477700153 | 9.4E-20 |
| ENSG00000164976 | MYORG | -1.476849453 | 3.29E-32 |
| ENSG00000242193 | CRYZL2P | -1.475028487 | 1.37E-09 |
| ENSG00000275835 | TUBGCP5 | -1.474533485 | 0.00131583 |
| ENSG00000282685 |  | -1.474501369 | 5.36E-14 |
| ENSG00000262172 | AC116025.2 | -1.474431803 | 0.039985333 |
| ENSG00000178665 | ZNF713 | -1.474328392 | 0.008343013 |
| ENSG00000182749 | PAQR7 | -1.472839256 | 4.1E-12 |
| ENSG00000163617 | CCDC191 | -1.472490312 | 0.00000362 |
| ENSG00000127419 | TMEM175 | -1.472397883 | 9.91E-11 |
| ENSG00000214900 | LINC01588 | -1.472175823 | 0.00145188 |
| ENSG00000280351 | AC127496.7 | -1.471931481 | 0.000427951 |
| ENSG00000159445 | THEM4 | -1.471221499 | 3.07E-17 |
| ENSG00000116704 | SLC35D1 | -1.470224962 | 8.63E-12 |
| ENSG00000274180 | NATD1 | -1.469745047 | 0.000000039 |
| ENSG00000104450 | SPAG1 | -1.46954004 | 5.8E-14 |
| ENSG00000006468 | ETV1 | -1.469016674 | 0.000000104 |
| ENSG00000163214 | DHX57 | -1.468909148 | 2.17E-13 |
| ENSG00000152784 | PRDM8 | -1.468351416 | 0.000000634 |
| ENSG00000184939 | ZFP90 | -1.46808851 | 1.08E-16 |
| ENSG00000198643 | FAM3D | -1.467312937 | 9.43E-17 |
| ENSG00000108984 | MAP2K6 | -1.465382589 | 0.00000122 |
| ENSG00000149474 | KAT14 | -1.465210979 | 2.54E-12 |
| ENSG00000110881 | ASIC1 | -1.465076893 | 2.05E-13 |
| ENSG00000165548 | TMEM63C | -1.463001424 | 0.0000756 |
| ENSG00000233922 | LINC01694 | -1.460974673 | 0.005702673 |
| ENSG00000260630 | SNAI3-AS1 | -1.460239321 | 0.003646886 |
| ENSG00000145632 | PLK2 | -1.458851129 | 1.12E-21 |
| ENSG00000103351 | CLUAP1 | -1.458097892 | 3.88E-11 |
| ENSG00000152348 | ATG10 | -1.45757622 | 4.44E-08 |
| ENSG00000250802 | ZBED3-AS1 | -1.456380171 | 0.031176127 |
| ENSG00000174173 | TRMT10C | -1.456088087 | 1.05E-27 |
| ENSG00000177971 | IMP3 | -1.454986384 | 5.49E-20 |
| ENSG00000229809 | ZNF688 | -1.454566546 | 0.000000187 |
| ENSG00000149054 | ZNF215 | -1.454517275 | 3.74E-15 |
| ENSG00000039139 | DNAH5 | -1.452796851 | 0.00004 |
| ENSG00000247626 | MARS2 | -1.45199508 | 5.23E-15 |
| ENSG00000170854 | RIOX2 | -1.451722984 | 2.61E-15 |
| ENSG00000185621 | LMLN | -1.45036989 | 0.00000325 |
| ENSG00000282221 | AC119427.1 | -1.449962392 | 0.000026 |
| ENSG00000136935 | GOLGA1 | -1.448870131 | 1.44E-23 |
| ENSG00000168282 | MGAT2 | -1.448793853 | 0.0000124 |
| ENSG00000112146 | FBXO9 | -1.448146047 | 2.13E-17 |
| ENSG00000271079 | CTAGE15 | -1.448145381 | 0.024898914 |
| ENSG00000164011 | ZNF691 | -1.447717263 | 8.28E-11 |
| ENSG00000165025 | SYK | -1.447222182 | 3.73E-13 |
| ENSG00000143633 | C1orf131 | -1.446806353 | 2.72E-19 |
| ENSG00000273230 | AC102953.2 | -1.446217205 | 0.000000241 |
| ENSG00000254670 | AC084859.1 | -1.44598298 | 0.000496384 |
| ENSG00000198185 | ZNF334 | -1.445026929 | 1.72E-11 |
| ENSG00000229980 | TOB1-AS1 | -1.444489852 | 0.00052754 |
| ENSG00000105948 | TTC26 | -1.443545325 | 1.97E-09 |
| ENSG00000184922 | FMNL1 | -1.443184039 | 0.020495032 |
| ENSG00000168778 | TCTN2 | -1.441448257 | 3E-14 |
| ENSG00000224739 | AC016735.1 | -1.441424116 | 0.016326986 |
| ENSG00000103248 | MTHFSD | -1.441152452 | 1.17E-15 |
| ENSG00000241399 | CD302 | -1.440278496 | 0.00000316 |
| ENSG00000223392 | CLDN10-AS1 | -1.440265707 | 0.016534768 |
| ENSG00000197763 | TXNRD3 | -1.440190531 | 0.000000653 |
| ENSG00000198515 | CNGA1 | -1.439866976 | 3.13E-09 |
| ENSG00000178401 | DNAJC22 | -1.439629566 | 1.9E-14 |
| ENSG00000144579 | CTDSP1 | -1.438169438 | 5.76E-30 |
| ENSG00000176715 | ACSF3 | -1.438143033 | 1.92E-21 |
| ENSG00000165029 | ABCA1 | -1.438132743 | 6.89E-10 |
| ENSG00000121966 | CXCR4 | -1.4364798 | 0.000453758 |
| ENSG00000162009 | SSTR5 | -1.435945106 | 0.00000774 |
| ENSG00000117877 | CD3EAP | -1.435900735 | 1.73E-14 |
| ENSG00000122507 | BBS9 | -1.435093325 | 3.88E-11 |
| ENSG00000237094 | AL732372.2 | -1.434983296 | 0.019087052 |
| ENSG00000162105 | SHANK2 | -1.433787141 | 0.000000699 |
| ENSG00000170464 | DNAJC18 | -1.433763174 | 0.00000472 |
| ENSG00000129038 | LOXL1 | -1.4313557 | 0.00000162 |
| ENSG00000270012 | AC232271.1 | -1.430269471 | 0.019856882 |
| ENSG00000067601 | PMS2P4 | -1.430202747 | 0.0000298 |
| ENSG00000204950 | LRRC10B | -1.429693024 | 6.81E-08 |
| ENSG00000128928 | IVD | -1.429573948 | 5.57E-26 |
| ENSG00000177432 | NAP1L5 | -1.428223744 | 0.0000278 |
| ENSG00000136040 | PLXNC1 | -1.428001747 | 0.045843535 |
| ENSG00000280789 | PAGR1 | -1.42746861 | 1.63E-09 |
| ENSG00000169372 | CRADD | -1.427437965 | 0.0000508 |
| ENSG00000262823 | AC127521.1 | -1.427126246 | 0.0000896 |
| ENSG00000111731 | C2CD5 | -1.426733163 | 3.99E-41 |
| ENSG00000107815 | TWNK | -1.425632354 | 8.68E-12 |
| ENSG00000167895 | TMC8 | -1.424131721 | 1.26E-08 |
| ENSG00000181513 | ACBD4 | -1.423963203 | 5.27E-15 |
| ENSG00000115365 | LANCL1 | -1.423614292 | 1.08E-25 |
| ENSG00000251169 | LINC01843 | -1.423539385 | 0.00000106 |
| ENSG00000182621 | PLCB1 | -1.421582151 | 0.0000002 |
| ENSG00000159714 | ZDHHC1 | -1.421534191 | 6.13E-10 |
| ENSG00000248092 | NNT-AS1 | -1.421074393 | 2.79E-16 |
| ENSG00000167740 | CYB5D2 | -1.421047775 | 0.000022 |
| ENSG00000083093 | PALB2 | -1.420817137 | 1.13E-13 |
| ENSG00000259264 |  | -1.419652195 | 0.0000705 |
| ENSG00000248429 | FAM198B-AS1 | -1.418793913 | 0.032182487 |
| ENSG00000184154 | LRTOMT | -1.418752466 | 1.42E-10 |
| ENSG00000157782 | CABP1 | -1.418691499 | 0.00000111 |
| ENSG00000130653 | PNPLA7 | -1.417265491 | 0.004799894 |
| ENSG00000122481 | RWDD3 | -1.416598543 | 0.001878182 |
| ENSG00000248663 | LINC00992 | -1.416443064 | 0.00000121 |
| ENSG00000139133 | ALG10 | -1.416311054 | 0.000000753 |
| ENSG00000044459 | CNTLN | -1.415422756 | 0.005302855 |
| ENSG00000198959 | TGM2 | -1.415163682 | 1.22E-10 |
| ENSG00000109576 | AADAT | -1.414303008 | 0.001901361 |
| ENSG00000131781 | FMO5 | -1.41415859 | 0.004149651 |
| ENSG00000120068 | HOXB8 | -1.413429143 | 1.02E-14 |
| ENSG00000149346 | SLX4IP | -1.412584826 | 0.0000021 |
| ENSG00000181904 | C5orf24 | -1.412484766 | 8.95E-27 |
| ENSG00000171617 | ENC1 | -1.411703423 | 1.27E-23 |
| ENSG00000158006 | PAFAH2 | -1.411673212 | 4.37E-26 |
| ENSG00000198042 | MAK16 | -1.411088247 | 5.25E-13 |
| ENSG00000239407 | Z68871.1 | -1.4094284 | 0.000815104 |
| ENSG00000184675 | AMER1 | -1.408577308 | 0.000000604 |
| ENSG00000216937 | CCDC7 | -1.408388721 | 0.002352842 |
| ENSG00000248008 | NRAV | -1.408232208 | 1.29E-10 |
| ENSG00000260565 | ERVK13-1 | -1.40747871 | 0.00000108 |
| ENSG00000177045 | SIX5 | -1.405048365 | 3.51E-16 |
| ENSG00000076770 | MBNL3 | -1.404879722 | 5.16E-15 |
| ENSG00000153982 | GDPD1 | -1.40368389 | 0.0000519 |
| ENSG00000167800 | TBX10 | -1.40269575 | 0.001175304 |
| ENSG00000257122 | RRN3P3 | -1.399926529 | 0.0000556 |
| ENSG00000197037 | ZSCAN25 | -1.399600046 | 4.56E-18 |
| ENSG00000254726 | MEX3A | -1.398641504 | 1.42E-12 |
| ENSG00000163155 | LYSMD1 | -1.398195813 | 0.005380114 |
| ENSG00000005469 | CROT | -1.397884461 | 1.91E-14 |
| ENSG00000272419 | AC241585.2 | -1.397814622 | 0.00000163 |
| ENSG00000152270 | PDE3B | -1.396038881 | 6.29E-35 |
| ENSG00000198680 | TUSC1 | -1.394950139 | 6.59E-15 |
| ENSG00000174516 | PELI3 | -1.39462163 | 1.39E-16 |
| ENSG00000198824 | CHAMP1 | -1.394569697 | 2.91E-22 |
| ENSG00000174606 | ANGEL2 | -1.393794504 | 4.98E-11 |
| ENSG00000176387 | HSD11B2 | -1.393707051 | 0.000011 |
| ENSG00000111554 | MDM1 | -1.392597389 | 6.98E-11 |
| ENSG00000197496 | SLC2A10 | -1.392215097 | 1.72E-11 |
| ENSG00000119636 | BBOF1 | -1.39170021 | 0.02582676 |
| ENSG00000172482 | AGXT | -1.39166362 | 0.006743339 |
| ENSG00000210144 | MT-TY | -1.391562307 | 0.000000194 |
| ENSG00000089820 | ARHGAP4 | -1.39102951 | 0.011982359 |
| ENSG00000143971 | ETAA1 | -1.388618357 | 3.04E-10 |
| ENSG00000106069 | CHN2 | -1.388536417 | 5.36E-08 |
| ENSG00000165338 | HECTD2 | -1.387573983 | 0.012781695 |
| ENSG00000047621 | C12orf4 | -1.387328326 | 6.26E-18 |
| ENSG00000133710 | SPINK5 | -1.38673354 | 0.006884008 |
| ENSG00000163026 | WDCP | -1.386670424 | 5.77E-16 |
| ENSG00000019144 | PHLDB1 | -1.386356189 | 1.13E-17 |
| ENSG00000223396 | RPS10P7 | -1.38575301 | 0.000550528 |
| ENSG00000276672 | AL161891.1 | -1.385270867 | 0.018180928 |
| ENSG00000137760 | ALKBH8 | -1.385058486 | 1.12E-08 |
| ENSG00000162383 | SLC1A7 | -1.384153384 | 0.000148693 |
| ENSG00000169951 | ZNF764 | -1.383682858 | 1.14E-12 |
| ENSG00000089195 | TRMT6 | -1.382930604 | 4.01E-32 |
| ENSG00000165417 | GTF2A1 | -1.382881495 | 6.07E-22 |
| ENSG00000146350 | TBC1D32 | -1.382427531 | 0.00000656 |
| ENSG00000173914 | RBM4B | -1.381936343 | 1.89E-23 |
| ENSG00000187676 | B3GLCT | -1.381324044 | 2.59E-18 |
| ENSG00000111605 | CPSF6 | -1.381018208 | 8.15E-42 |
| ENSG00000149043 | SYT8 | -1.380484021 | 0.00000788 |
| ENSG00000179546 | HTR1D | -1.379964286 | 0.003043661 |
| ENSG00000122512 | PMS2 | -1.379830276 | 3.22E-18 |
| ENSG00000198727 | MT-CYB | -1.377938021 | 0.009583047 |
| ENSG00000037474 | NSUN2 | -1.377654648 | 2.18E-25 |
| ENSG00000234602 | MCIDAS | -1.37740148 | 0.002888174 |
| ENSG00000174007 | CEP19 | -1.377292063 | 0.001528032 |
| ENSG00000122687 | MRM2 | -1.37472655 | 1.16E-35 |
| ENSG00000171160 | MORN4 | -1.373941955 | 0.0000366 |
| ENSG00000104691 | UBXN8 | -1.37257673 | 0.000200015 |
| ENSG00000196152 | ZNF79 | -1.370898652 | 1.33E-08 |
| ENSG00000116984 | MTR | -1.370475697 | 5.68E-21 |
| ENSG00000177058 | SLC38A9 | -1.370393021 | 4.13E-14 |
| ENSG00000261971 | MMP25-AS1 | -1.369883497 | 0.031167647 |
| ENSG00000142686 | C1orf216 | -1.368808743 | 8.46E-12 |
| ENSG00000175066 | GK5 | -1.36813625 | 1.19E-21 |
| ENSG00000196597 | ZNF782 | -1.367599656 | 0.0000373 |
| ENSG00000162650 | ATXN7L2 | -1.366904784 | 1.06E-11 |
| ENSG00000111981 | ULBP1 | -1.366596151 | 0.0000173 |
| ENSG00000198417 | MT1F | -1.366423962 | 0.027680516 |
| ENSG00000111817 | DSE | -1.364474849 | 0.040336266 |
| ENSG00000143578 | CREB3L4 | -1.364176598 | 7.26E-20 |
| ENSG00000196267 | ZNF836 | -1.363602637 | 0.000000233 |
| ENSG00000119684 | MLH3 | -1.36184621 | 2.62E-11 |
| ENSG00000258474 | LINC02313 | -1.361813287 | 0.016557546 |
| ENSG00000259768 | AC004943.2 | -1.361723805 | 5.51E-09 |
| ENSG00000164252 | AGGF1 | -1.361317265 | 2.9E-31 |
| ENSG00000048342 | CC2D2A | -1.359734223 | 0.001493727 |
| ENSG00000183340 | JRKL | -1.359549606 | 1.81E-23 |
| ENSG00000160993 | ALKBH4 | -1.359440628 | 5.56E-15 |
| ENSG00000196268 | ZNF493 | -1.359423374 | 0.00264521 |
| ENSG00000002726 | AOC1 | -1.356067105 | 6.44E-15 |
| ENSG00000205809 | KLRC2 | -1.353891742 | 0.015736855 |
| ENSG00000121310 | ECHDC2 | -1.353367106 | 1.93E-08 |
| ENSG00000270504 | AL391422.3 | -1.352228915 | 0.0000515 |
| ENSG00000170365 | SMAD1 | -1.352171644 | 7.74E-12 |
| ENSG00000130021 | PUDP | -1.350465899 | 1.43E-12 |
| ENSG00000242861 | AL591895.1 | -1.349690631 | 0.000123189 |
| ENSG00000179348 | GATA2 | -1.348747191 | 1.48E-23 |
| ENSG00000182796 | TMEM198B | -1.348412839 | 0.000615247 |
| ENSG00000235554 | AC005822.1 | -1.347668451 | 0.018794422 |
| ENSG00000138231 | DBR1 | -1.347579404 | 6.57E-13 |
| ENSG00000130701 | RBBP8NL | -1.347236534 | 1.32E-11 |
| ENSG00000158315 | RHBDL2 | -1.34624574 | 7.79E-09 |
| ENSG00000204147 | ASAH2B | -1.346132882 | 1.08E-10 |
| ENSG00000049883 | PTCD2 | -1.34588391 | 2.24E-10 |
| ENSG00000164402 | 44447 | -1.345559786 | 1.54E-28 |
| ENSG00000241945 | PWP2 | -1.343303876 | 0.0000408 |
| ENSG00000213949 | ITGA1 | -1.342531371 | 7.67E-14 |
| ENSG00000170468 | RIOX1 | -1.34207181 | 9.48E-14 |
| ENSG00000172339 | ALG14 | -1.342071155 | 1.9E-12 |
| ENSG00000143365 | RORC | -1.341949201 | 0.00000984 |
| ENSG00000120942 | UBIAD1 | -1.341888653 | 1E-25 |
| ENSG00000198919 | DZIP3 | -1.340977757 | 7.32E-10 |
| ENSG00000147475 | ERLIN2 | -1.340728424 | 4.49E-16 |
| ENSG00000149313 | AASDHPPT | -1.339629261 | 4.07E-16 |
| ENSG00000173638 | SLC19A1 | -1.339093069 | 3.05E-24 |
| ENSG00000246526 | LINC002481 | -1.337849227 | 0.01075961 |
| ENSG00000100211 | CBY1 | -1.336299411 | 3.73E-13 |
| ENSG00000151690 | MFSD6 | -1.336268706 | 1.55E-16 |
| ENSG00000113070 | HBEGF | -1.33453761 | 0.011801947 |
| ENSG00000180921 | FAM83H | -1.333805575 | 0.031092691 |
| ENSG00000116205 | TCEANC2 | -1.333435588 | 3.95E-18 |
| ENSG00000062725 | APPBP2 | -1.333326376 | 2.77E-28 |
| ENSG00000185220 | PGBD2 | -1.33332423 | 4.4E-11 |
| ENSG00000154059 | IMPACT | -1.3332565 | 1.63E-11 |
| ENSG00000189362 | NEMP2 | -1.333168987 | 2.89E-09 |
| ENSG00000198786 | MT-ND5 | -1.332738684 | 0.02191767 |
| ENSG00000177311 | ZBTB38 | -1.332330564 | 3.46E-13 |
| ENSG00000255062 | AP001318.2 | -1.331742574 | 0.006962469 |
| ENSG00000167588 | GPD1 | -1.330784712 | 0.000000355 |
| ENSG00000204740 | MALRD1 | -1.329817246 | 0.008878592 |
| ENSG00000157368 | IL34 | -1.329137091 | 0.002106789 |
| ENSG00000187535 | IFT140 | -1.328905927 | 7.99E-11 |
| ENSG00000162600 | OMA1 | -1.328545363 | 1.43E-10 |
| ENSG00000139318 | DUSP6 | -1.328389414 | 1.69E-10 |
| ENSG00000159674 | SPON2 | -1.327826508 | 0.017258529 |
| ENSG00000104129 | DNAJC17 | -1.327165041 | 8.29E-15 |
| ENSG00000176855 | KRT18P28 | -1.326582802 | 0.005346984 |
| ENSG00000241764 | AC002467.1 | -1.32656009 | 0.006224653 |
| ENSG00000076351 | SLC46A1 | -1.326454192 | 0.000000738 |
| ENSG00000277534 | AC007996.1 | -1.325553301 | 0.00000114 |
| ENSG00000196872 | KIAA1211L | -1.32456762 | 1.85E-08 |
| ENSG00000271781 | AC026740.1 | -1.32377723 | 0.012888682 |
| ENSG00000164038 | SLC9B2 | -1.323097943 | 0.000124735 |
| ENSG00000263753 | LINC00667 | -1.32287983 | 7.77E-14 |
| ENSG00000172878 | METAP1D | -1.321801075 | 0.0000239 |
| ENSG00000125630 | POLR1B | -1.320570626 | 3.17E-17 |
| ENSG00000243896 | OR2A7 | -1.319565842 | 0.03810454 |
| ENSG00000134986 | NREP | -1.317825492 | 0.0000589 |
| ENSG00000179743 | AL450998.2 | -1.317562022 | 0.000000236 |
| ENSG00000261801 | LOXL1-AS1 | -1.317362883 | 0.000183959 |
| ENSG00000271254 | AC240274.1 | -1.316861642 | 0.002751869 |
| ENSG00000080839 | RBL1 | -1.316160174 | 1.33E-16 |
| ENSG00000007968 | E2F2 | -1.315357039 | 0.00000114 |
| ENSG00000221994 | ZNF630 | -1.315250685 | 0.000609782 |
| ENSG00000130810 | PPAN | -1.314168842 | 0.000218963 |
| ENSG00000093217 | XYLB | -1.313004355 | 0.000496964 |
| ENSG00000179403 | VWA1 | -1.312243353 | 3.08E-36 |
| ENSG00000172264 | MACROD2 | -1.310884341 | 0.00000247 |
| ENSG00000102870 | ZNF629 | -1.309806123 | 7.22E-15 |
| ENSG00000164631 | ZNF12 | -1.30951006 | 1.16E-15 |
| ENSG00000275342 | PRAG1 | -1.308830938 | 2.62E-09 |
| ENSG00000164124 | TMEM144 | -1.307894193 | 6.56E-13 |
| ENSG00000118160 | SLC8A2 | -1.30733237 | 0.011643876 |
| ENSG00000180488 | MIGA1 | -1.307223707 | 3.07E-18 |
| ENSG00000117480 | FAAH | -1.306254797 | 3.09E-17 |
| ENSG00000151148 | UBE3B | -1.306044031 | 1.67E-22 |
| ENSG00000129691 | ASH2L | -1.305428497 | 1.43E-19 |
| ENSG00000090989 | EXOC1 | -1.305186196 | 1.08E-25 |
| ENSG00000177663 | IL17RA | -1.304294345 | 3.37E-21 |
| ENSG00000034693 | PEX3 | -1.303580663 | 1.22E-13 |
| ENSG00000275464 | FP565260.1 | -1.302722422 | 3.35E-11 |
| ENSG00000176533 | GNG7 | -1.301276965 | 0.019869889 |
| ENSG00000090447 | TFAP4 | -1.299844453 | 2.24E-14 |
| ENSG00000180423 | HARBI1 | -1.299276736 | 0.000000049 |
| ENSG00000122085 | MTERF4 | -1.298331427 | 1.29E-21 |
| ENSG00000134508 | CABLES1 | -1.297473305 | 3.99E-14 |
| ENSG00000238058 | AL355574.1 | -1.297254243 | 0.044447391 |
| ENSG00000095261 | PSMD5 | -1.297122765 | 1.63E-36 |
| ENSG00000179913 | B3GNT3 | -1.296908018 | 3.91E-26 |
| ENSG00000114491 | UMPS | -1.296818048 | 8.01E-30 |
| ENSG00000125375 | ATP5S | -1.296214297 | 4.19E-09 |
| ENSG00000130347 | RTN4IP1 | -1.296187201 | 2.41E-09 |
| ENSG00000171914 | TLN2 | -1.295191748 | 0.000710798 |
| ENSG00000188488 | SERPINA5 | -1.29366791 | 2.87E-09 |
| ENSG00000231806 | PCAT7 | -1.29158616 | 0.001024335 |
| ENSG00000155085 | AK9 | -1.291148314 | 0.000000409 |
| ENSG00000162069 | BICDL2 | -1.290197865 | 2.65E-10 |
| ENSG00000162391 | FAM151A | -1.290007521 | 0.030217631 |
| ENSG00000273151 | AC073957.3 | -1.287810064 | 0.000312483 |
| ENSG00000120697 | ALG5 | -1.287098276 | 6.09E-20 |
| ENSG00000109339 | MAPK10 | -1.286592565 | 0.045091774 |
| ENSG00000273780 |  | -1.285894617 | 0.000000373 |
| ENSG00000165269 | AQP7 | -1.283940116 | 0.034172429 |
| ENSG00000266053 | NDUFV2-AS1 | -1.283891347 | 0.021467333 |
| ENSG00000268573 | AC011815.1 | -1.283863438 | 0.00000449 |
| ENSG00000152147 | GEMIN6 | -1.283847818 | 1.26E-13 |
| ENSG00000185499 | MUC1 | -1.283276276 | 0.032552061 |
| ENSG00000134318 | ROCK2 | -1.283226433 | 2.36E-12 |
| ENSG00000085982 | USP40 | -1.283160633 | 0.00000191 |
| ENSG00000105429 | MEGF8 | -1.28308236 | 6.9E-09 |
| ENSG00000267080 | ASB16-AS1 | -1.281095359 | 0.0000277 |
| ENSG00000206530 | CFAP44 | -1.280911693 | 0.00000585 |
| ENSG00000273247 | AC097376.2 | -1.280729216 | 0.001041912 |
| ENSG00000225526 | MKRN2OS | -1.278832084 | 0.011762655 |
| ENSG00000101076 | HNF4A | -1.277219729 | 1.83E-15 |
| ENSG00000168802 | CHTF8 | -1.27693567 | 5.56E-31 |
| ENSG00000178460 | MCMDC2 | -1.275765238 | 0.006583507 |
| ENSG00000123600 | METTL8 | -1.275589227 | 4.41E-18 |
| ENSG00000166436 | TRIM66 | -1.275495667 | 0.000120872 |
| ENSG00000131944 | FAAP24 | -1.275249819 | 2.05E-11 |
| ENSG00000162390 | ACOT11 | -1.274987371 | 2.44E-10 |
| ENSG00000177853 | ZNF518A | -1.274946464 | 4.02E-24 |
| ENSG00000037757 | MRI1 | -1.273500844 | 0.0000187 |
| ENSG00000231826 | LINC01819 | -1.273282302 | 0.006368447 |
| ENSG00000258297 | AP001157.1 | -1.273162262 | 0.00000143 |
| ENSG00000171552 | BCL2L1 | -1.272362767 | 9.41E-13 |
| ENSG00000165795 | NDRG2 | -1.27133009 | 1.99E-09 |
| ENSG00000106012 | IQCE | -1.270977386 | 3.71E-23 |
| ENSG00000150456 | EEF1AKMT1 | -1.270425146 | 3E-11 |
| ENSG00000160221 | C21orf33 | -1.270359075 | 0.002977682 |
| ENSG00000088367 | EPB41L1 | -1.270108919 | 2.79E-14 |
| ENSG00000123444 | KBTBD4 | -1.26958557 | 9.53E-11 |
| ENSG00000139505 | MTMR6 | -1.269163926 | 4.4E-22 |
| ENSG00000273045 | C2orf15 | -1.268499239 | 0.000000504 |
| ENSG00000128268 | MGAT3 | -1.267637614 | 2.25E-08 |
| ENSG00000245648 | AC022075.1 | -1.266685496 | 0.000799679 |
| ENSG00000203326 | ZNF525 | -1.266202042 | 0.0000016 |
| ENSG00000277938 | AL035252.3 | -1.266077059 | 0.000276037 |
| ENSG00000132664 | POLR3F | -1.2656542 | 2.42E-16 |
| ENSG00000264885 | AC026271.3 | -1.264726427 | 0.020211701 |
| ENSG00000084090 | STARD7 | -1.26261123 | 2.66E-31 |
| ENSG00000154309 | DISP1 | -1.262192426 | 0.0000978 |
| ENSG00000134253 | TRIM45 | -1.262111424 | 0.000295954 |
| ENSG00000006534 | ALDH3B1 | -1.261651497 | 1.84E-15 |
| ENSG00000173137 | ADCK5 | -1.26162437 | 3.39E-09 |
| ENSG00000198853 | RUSC2 | -1.259185877 | 0.016993641 |
| ENSG00000204789 | ZNF204P | -1.259182443 | 0.011850485 |
| ENSG00000236397 | DDX11L2 | -1.258431726 | 0.014171411 |
| ENSG00000166352 | C11orf74 | -1.256513378 | 0.0000045 |
| ENSG00000176209 | SMIM19 | -1.255825587 | 0.00000019 |
| ENSG00000100479 | POLE2 | -1.255634913 | 5.13E-08 |
| ENSG00000185379 | RAD51D | -1.255380071 | 1.87E-24 |
| ENSG00000142627 | EPHA2 | -1.254856295 | 2.47E-15 |
| ENSG00000137601 | NEK1 | -1.253836578 | 7.54E-08 |
| ENSG00000113621 | TXNDC15 | -1.252207492 | 1.39E-12 |
| ENSG00000110195 | FOLR1 | -1.251928484 | 0.005124628 |
| ENSG00000196417 | ZNF765 | -1.251704125 | 1.07E-26 |
| ENSG00000188343 | FAM92A | -1.251618344 | 0.000123613 |
| ENSG00000226383 | LINC01876 | -1.251541018 | 0.048150134 |
| ENSG00000164933 | SLC25A32 | -1.250077531 | 1.52E-18 |
| ENSG00000269313 | MAGIX | -1.249797659 | 0.00000143 |
| ENSG00000172667 | ZMAT3 | -1.249516644 | 1.38E-14 |
| ENSG00000083123 | BCKDHB | -1.249286318 | 6.7E-13 |
| ENSG00000178096 | BOLA1 | -1.249072806 | 0.0000029 |
| ENSG00000138641 | HERC3 | -1.248810498 | 0.000000172 |
| ENSG00000188549 | CCDC9B | -1.248681631 | 5.21E-12 |
| ENSG00000160606 | TLCD1 | -1.24864254 | 1.37E-15 |
| ENSG00000135999 | EPC2 | -1.248201056 | 1.66E-11 |
| ENSG00000074590 | NUAK1 | -1.24818594 | 0.0000241 |
| ENSG00000167105 | TMEM92 | -1.247348557 | 2.83E-11 |
| ENSG00000186862 | PDZD7 | -1.247289329 | 0.000000016 |
| ENSG00000269386 | RAB11B-AS1 | -1.246995348 | 0.000254873 |
| ENSG00000136936 | XPA | -1.245094232 | 2.79E-09 |
| ENSG00000186564 | FOXD2 | -1.244962087 | 0.000000428 |
| ENSG00000272763 | AC103702.2 | -1.24478041 | 3.23E-27 |
| ENSG00000250299 | MRPS31P4 | -1.244734973 | 0.00933535 |
| ENSG00000106003 | LFNG | -1.244453277 | 4.11E-24 |
| ENSG00000169129 | AFAP1L2 | -1.244298214 | 3.79E-22 |
| ENSG00000058453 | CROCC | -1.24383609 | 0.00000461 |
| ENSG00000100650 | SRSF5 | -1.243800832 | 1.2E-34 |
| ENSG00000132394 | EEFSEC | -1.243551168 | 1.41E-16 |
| ENSG00000076003 | MCM6 | -1.243049022 | 1.21E-11 |
| ENSG00000135392 | DNAJC14 | -1.242515838 | 4.66E-19 |
| ENSG00000136213 | CHST12 | -1.242279885 | 2.87E-24 |
| ENSG00000145916 | RMND5B | -1.241936478 | 9.73E-30 |
| ENSG00000132669 | RIN2 | -1.241749108 | 3.2E-25 |
| ENSG00000164323 | CFAP97 | -1.241123542 | 5.93E-15 |
| ENSG00000226453 | LINC02542 | -1.239143647 | 0.02988734 |
| ENSG00000164306 | PRIMPOL | -1.238187321 | 1.1E-09 |
| ENSG00000000457 | SCYL3 | -1.238103319 | 1.96E-18 |
| ENSG00000132773 | TOE1 | -1.236945829 | 9.22E-17 |
| ENSG00000175164 | ABO | -1.235725395 | 0.000000773 |
| ENSG00000275832 | ARHGAP23 | -1.235603128 | 2.44E-09 |
| ENSG00000132436 | FIGNL1 | -1.235584671 | 1.54E-11 |
| ENSG00000075213 | SEMA3A | -1.235527689 | 0.000000011 |
| ENSG00000130193 | THEM6 | -1.23532846 | 1.85E-24 |
| ENSG00000215883 | CYB5RL | -1.234904679 | 0.0000297 |
| ENSG00000187720 | THSD4 | -1.234872161 | 1.8E-09 |
| ENSG00000181031 | RPH3AL | -1.234490051 | 0.000165768 |
| ENSG00000168569 | TMEM223 | -1.233950791 | 5.11E-17 |
| ENSG00000162408 | NOL9 | -1.233640624 | 1.67E-14 |
| ENSG00000140691 | ARMC5 | -1.233610132 | 7.54E-08 |
| ENSG00000115282 | TTC31 | -1.233396767 | 1.2E-09 |
| ENSG00000165801 | ARHGEF40 | -1.233218927 | 1.94E-13 |
| ENSG00000130052 | STARD8 | -1.232652032 | 0.00215167 |
| ENSG00000167100 | SAMD14 | -1.232586127 | 0.001929289 |
| ENSG00000280145 | CU638689.4 | -1.232558161 | 0.03684604 |
| ENSG00000150750 | C11orf53 | -1.231295817 | 0.00000129 |
| ENSG00000179387 | ELMOD2 | -1.23044003 | 3E-16 |
| ENSG00000255310 | AF131215.5 | -1.230364952 | 0.007713311 |
| ENSG00000114812 | VIPR1 | -1.230117746 | 1.49E-15 |
| ENSG00000100105 | PATZ1 | -1.228692705 | 6.95E-24 |
| ENSG00000178567 | EPM2AIP1 | -1.22815257 | 0.00000222 |
| ENSG00000171262 | FAM98B | -1.22681523 | 2.41E-20 |
| ENSG00000235173 | HGH1 | -1.226736554 | 2.38E-17 |
| ENSG00000196678 | ERI2 | -1.226723199 | 3.53E-13 |
| ENSG00000054282 | SDCCAG8 | -1.226507792 | 0.005048453 |
| ENSG00000107560 | RAB11FIP2 | -1.226405108 | 0.000116028 |
| ENSG00000116833 | NR5A2 | -1.225413237 | 0.000000164 |
| ENSG00000100665 | SERPINA4 | -1.22493433 | 0.00000873 |
| ENSG00000164332 | UBLCP1 | -1.224838117 | 2.51E-13 |
| ENSG00000151498 | ACAD8 | -1.224564321 | 3.07E-15 |
| ENSG00000132825 | PPP1R3D | -1.222505545 | 2.51E-13 |
| ENSG00000141519 | CCDC40 | -1.221089646 | 0.000200105 |
| ENSG00000159214 | CCDC24 | -1.220984694 | 0.0000283 |
| ENSG00000011523 | CEP68 | -1.22084123 | 5.85E-11 |
| ENSG00000135966 | TGFBRAP1 | -1.22019286 | 1.47E-21 |
| ENSG00000213742 | ZNF337-AS1 | -1.220142465 | 0.0005089 |
| ENSG00000100557 | CCDC198 | -1.220037066 | 0.000000697 |
| ENSG00000196187 | TMEM63A | -1.219074006 | 4.67E-21 |
| ENSG00000153214 | TMEM87B | -1.218991412 | 3.27E-24 |
| ENSG00000112425 | EPM2A | -1.218483847 | 0.002856663 |
| ENSG00000181638 | ZFP41 | -1.216887701 | 0.0000402 |
| ENSG00000152952 | PLOD2 | -1.216250245 | 1.72E-15 |
| ENSG00000132600 | PRMT7 | -1.215191378 | 1.94E-17 |
| ENSG00000157350 | ST3GAL2 | -1.214718326 | 4.07E-17 |
| ENSG00000152433 | ZNF547 | -1.214351642 | 0.012002056 |
| ENSG00000150540 | HNMT | -1.214206153 | 3.45E-20 |
| ENSG00000143416 | SELENBP1 | -1.213215074 | 0.000000136 |
| ENSG00000188559 | RALGAPA2 | -1.213165098 | 2.99E-10 |
| ENSG00000166415 | WDR72 | -1.213006812 | 1.55E-10 |
| ENSG00000176974 | SHMT1 | -1.212360143 | 1.39E-23 |
| ENSG00000128965 | CHAC1 | -1.212031348 | 0.001575116 |
| ENSG00000170264 | FAM161A | -1.212009655 | 0.00000706 |
| ENSG00000092969 | TGFB2 | -1.211800742 | 0.00000999 |
| ENSG00000164403 | SHROOM1 | -1.209849885 | 0.000000217 |
| ENSG00000141013 | GAS8 | -1.209504772 | 6.35E-12 |
| ENSG00000168010 | ATG16L2 | -1.208917744 | 0.000000398 |
| ENSG00000164209 | SLC25A46 | -1.207753 | 8.18E-22 |
| ENSG00000069702 | TGFBR3 | -1.20628157 | 0.00000326 |
| ENSG00000167964 | RAB26 | -1.206181746 | 9.24E-08 |
| ENSG00000267213 | AC007773.1 | -1.205182488 | 0.026470261 |
| ENSG00000119927 | GPAM | -1.204019099 | 5.96E-18 |
| ENSG00000276234 | TADA2A | -1.203681072 | 0.000304332 |
| ENSG00000164815 | ORC5 | -1.203546431 | 4.86E-17 |
| ENSG00000169598 | DFFB | -1.203498781 | 4.9E-09 |
| ENSG00000275074 | NUDT18 | -1.202799934 | 0.000405299 |
| ENSG00000179409 | GEMIN4 | -1.202750321 | 4.35E-27 |
| ENSG00000180340 | FZD2 | -1.201265806 | 0.000560153 |
| ENSG00000261150 | EPPK1 | -1.201255885 | 0.001960652 |
| ENSG00000157895 | C12orf43 | -1.200475408 | 3.44E-13 |
| ENSG00000263465 | SRSF8 | -1.200289938 | 1.28E-12 |
| ENSG00000151150 | ANK3 | -1.198669151 | 4.43E-13 |
| ENSG00000114805 | PLCH1 | -1.198650496 | 2.1E-10 |
| ENSG00000160401 | CFAP157 | -1.198198356 | 0.023345843 |
| ENSG00000244649 | LINC02086 | -1.19810531 | 0.00000482 |
| ENSG00000134014 | ELP3 | -1.197901324 | 3.88E-16 |
| ENSG00000145284 | SCD5 | -1.197464867 | 0.033439797 |
| ENSG00000196371 | FUT4 | -1.196828888 | 3.66E-24 |
| ENSG00000110092 | CCND1 | -1.195711949 | 1.7E-10 |
| ENSG00000132434 | LANCL2 | -1.195634123 | 1.33E-09 |
| ENSG00000164077 | MON1A | -1.195581621 | 0.0000031 |
| ENSG00000180257 | ZNF816 | -1.195488381 | 3.45E-15 |
| ENSG00000133067 | LGR6 | -1.195438595 | 0.005718726 |
| ENSG00000104133 | SPG11 | -1.195144623 | 1.33E-11 |
| ENSG00000246174 | KCTD21-AS1 | -1.194949855 | 0.024349509 |
| ENSG00000130590 | SAMD10 | -1.194635561 | 6.69E-22 |
| ENSG00000281500 |  | -1.193584763 | 0.000063 |
| ENSG00000058056 | USP13 | -1.192593076 | 8.29E-12 |
| ENSG00000181409 | AATK | -1.191245237 | 6.74E-16 |
| ENSG00000138002 | IFT172 | -1.190874196 | 4.31E-09 |
| ENSG00000113119 | TMCO6 | -1.190214187 | 3.25E-14 |
| ENSG00000095539 | SEMA4G | -1.188441766 | 1.99E-12 |
| ENSG00000179981 | TSHZ1 | -1.18721383 | 1.02E-11 |
| ENSG00000198924 | DCLRE1A | -1.186764695 | 6.43E-23 |
| ENSG00000117697 | NSL1 | -1.186759619 | 1.09E-23 |
| ENSG00000087008 | ACOX3 | -1.186491939 | 1.38E-14 |
| ENSG00000137218 | FRS3 | -1.185990364 | 0.00000289 |
| ENSG00000039319 | ZFYVE16 | -1.185916616 | 5.25E-11 |
| ENSG00000136044 | APPL2 | -1.185837185 | 7.58E-21 |
| ENSG00000260265 | LINC02562 | -1.184126973 | 0.0000182 |
| ENSG00000181284 | TMEM102 | -1.183942639 | 7.45E-10 |
| ENSG00000104361 | NIPAL2 | -1.182672251 | 3.93E-08 |
| ENSG00000151575 | TEX9 | -1.18249789 | 0.000565703 |
| ENSG00000180626 | ZNF594 | -1.181184538 | 0.000344832 |
| ENSG00000174827 | PDZK1 | -1.180150914 | 0.0008617 |
| ENSG00000158423 | RIBC1 | -1.179354821 | 0.004864145 |
| ENSG00000180176 | TH | -1.178054734 | 0.015064021 |
| ENSG00000277072 | STAG3L2 | -1.17756384 | 6.47E-09 |
| ENSG00000166813 | KIF7 | -1.177340257 | 0.0000575 |
| ENSG00000161653 | NAGS | -1.176623515 | 0.000794522 |
| ENSG00000148459 | PDSS1 | -1.174413438 | 9.21E-10 |
| ENSG00000159247 | TUBBP5 | -1.174171324 | 0.000560153 |
| ENSG00000082516 | GEMIN5 | -1.173804235 | 1.65E-16 |
| ENSG00000124279 | FASTKD3 | -1.173655342 | 4.84E-16 |
| ENSG00000205593 | DENND6B | -1.173513332 | 0.0000312 |
| ENSG00000136381 | IREB2 | -1.172150328 | 1.17E-26 |
| ENSG00000189195 | BTBD8 | -1.171823625 | 0.012340676 |
| ENSG00000109586 | GALNT7 | -1.170618911 | 1.19E-12 |
| ENSG00000273850 |  | -1.170220921 | 0.0000158 |
| ENSG00000166133 | RPUSD2 | -1.170172059 | 1.68E-14 |
| ENSG00000111670 | GNPTAB | -1.167964508 | 7.08E-14 |
| ENSG00000186815 | TPCN1 | -1.16778109 | 4.57E-13 |
| ENSG00000143353 | LYPLAL1 | -1.167541632 | 1.85E-10 |
| ENSG00000146828 | SLC12A9 | -1.167042642 | 4.35E-16 |
| ENSG00000177570 | SAMD12 | -1.166815594 | 1.05E-13 |
| ENSG00000204323 | SMIM5 | -1.165550675 | 0.005932819 |
| ENSG00000259120 | SMIM6 | -1.165516349 | 2.27E-08 |
| ENSG00000234912 | SNHG20 | -1.16539989 | 0.004341563 |
| ENSG00000133028 | SCO1 | -1.164610515 | 6.15E-18 |
| ENSG00000181873 | IBA57 | -1.163870941 | 4.06E-15 |
| ENSG00000178695 | KCTD12 | -1.163504588 | 2.38E-08 |
| ENSG00000119943 | PYROXD2 | -1.163023599 | 0.000000427 |
| ENSG00000263142 | LRRC37A17P | -1.162950903 | 0.000161185 |
| ENSG00000196074 | SYCP2 | -1.160709057 | 0.01520023 |
| ENSG00000108666 | C17orf75 | -1.160021374 | 7.49E-19 |
| ENSG00000273142 | AC073335.2 | -1.159821507 | 0.021956348 |
| ENSG00000100307 | CBX7 | -1.159597552 | 2.79E-12 |
| ENSG00000245556 | SCAMP1-AS1 | -1.159232769 | 1.97E-09 |
| ENSG00000142875 | PRKACB | -1.158308457 | 0.000000534 |
| ENSG00000141736 | ERBB2 | -1.15814053 | 7.9E-20 |
| ENSG00000263072 | ZNF213-AS1 | -1.158032814 | 0.00000995 |
| ENSG00000080493 | SLC4A4 | -1.157438951 | 3.11E-10 |
| ENSG00000169231 | THBS3 | -1.156719239 | 0.001067827 |
| ENSG00000265763 | ZNF488 | -1.156687903 | 0.00000482 |
| ENSG00000225032 | AL162586.1 | -1.15647783 | 0.004790002 |
| ENSG00000276021 |  | -1.156326109 | 0.00000216 |
| ENSG00000127989 | MTERF1 | -1.154670094 | 2.48E-18 |
| ENSG00000103174 | NAGPA | -1.154502365 | 3.2E-13 |
| ENSG00000006555 | TTC22 | -1.154225752 | 8.29E-15 |
| ENSG00000264575 | LINC00526 | -1.153813444 | 0.00007 |
| ENSG00000175611 | LINC00476 | -1.153665215 | 0.000302593 |
| ENSG00000104812 | GYS1 | -1.153248354 | 5.99E-19 |
| ENSG00000115827 | DCAF17 | -1.152744619 | 2.29E-09 |
| ENSG00000119004 | CYP20A1 | -1.150883232 | 5.69E-10 |
| ENSG00000101188 | NTSR1 | -1.149676877 | 0.001976507 |
| ENSG00000185813 | PCYT2 | -1.149173792 | 2.4E-21 |
| ENSG00000143553 | SNAPIN | -1.148793849 | 1.39E-17 |
| ENSG00000101276 | SLC52A3 | -1.148491904 | 9.38E-10 |
| ENSG00000146409 | SLC18B1 | -1.148226772 | 6.17E-20 |
| ENSG00000228638 | FCF1P2 | -1.147446171 | 0.003825866 |
| ENSG00000244005 | NFS1 | -1.147236547 | 5E-19 |
| ENSG00000101191 | DIDO1 | -1.146825816 | 4.87E-16 |
| ENSG00000148824 | MTG1 | -1.146800206 | 0.000394195 |
| ENSG00000180530 | NRIP1 | -1.146588634 | 8.96E-14 |
| ENSG00000117174 | ZNHIT6 | -1.146329417 | 1.39E-08 |
| ENSG00000116852 | KIF21B | -1.144935457 | 0.000000324 |
| ENSG00000130772 | MED18 | -1.1443362 | 8.84E-19 |
| ENSG00000151320 | AKAP6 | -1.143819375 | 0.032352376 |
| ENSG00000276043 | UHRF1 | -1.143711993 | 3.67E-12 |
| ENSG00000223478 | AL441992.1 | -1.143449278 | 0.006990431 |
| ENSG00000213516 | RBMXL1 | -1.143304367 | 2.01E-21 |
| ENSG00000272667 | AC012306.2 | -1.14235804 | 0.002395628 |
| ENSG00000163832 | ELP6 | -1.141736534 | 1.07E-13 |
| ENSG00000138061 | CYP1B1 | -1.141472661 | 1.75E-11 |
| ENSG00000143224 | PPOX | -1.141080511 | 8.27E-11 |
| ENSG00000137135 | ARHGEF39 | -1.140484314 | 5.26E-09 |
| ENSG00000089280 | FUS | -1.14028073 | 7.21E-23 |
| ENSG00000185722 | ANKFY1 | -1.138866684 | 4.1E-12 |
| ENSG00000184992 | BRI3BP | -1.138397289 | 6.57E-18 |
| ENSG00000196453 | ZNF777 | -1.138039448 | 1.47E-09 |
| ENSG00000260628 | AC142381.3 | -1.138024118 | 0.000198502 |
| ENSG00000153246 | PLA2R1 | -1.137956536 | 6.21E-12 |
| ENSG00000205978 | NYNRIN | -1.137744143 | 1.25E-09 |
| ENSG00000167528 | ZNF641 | -1.137498556 | 1.18E-12 |
| ENSG00000162591 | MEGF6 | -1.135025815 | 0.000000503 |
| ENSG00000153531 | ADPRHL1 | -1.133730378 | 0.000365856 |
| ENSG00000197165 | SULT1A2 | -1.133466636 | 0.002236152 |
| ENSG00000188747 | NOXA1 | -1.132772104 | 0.000364521 |
| ENSG00000245937 | LINC01184 | -1.132675099 | 1.51E-14 |
| ENSG00000112874 | NUDT12 | -1.132117063 | 1.57E-14 |
| ENSG00000137968 | SLC44A5 | -1.131702561 | 0.00000134 |
| ENSG00000139679 | LPAR6 | -1.131672224 | 0.0000593 |
| ENSG00000259891 | AC107375.1 | -1.131265389 | 0.004331911 |
| ENSG00000272482 | AC254633.1 | -1.130861931 | 0.039073377 |
| ENSG00000111801 | BTN3A3 | -1.13019225 | 0.000127158 |
| ENSG00000063176 | SPHK2 | -1.130118786 | 3.81E-09 |
| ENSG00000168237 | GLYCTK | -1.130025059 | 0.00000685 |
| ENSG00000169047 | IRS1 | -1.130019983 | 2E-15 |
| ENSG00000163629 | PTPN13 | -1.129369424 | 1.13E-08 |
| ENSG00000197385 | ZNF860 | -1.12807921 | 0.0000507 |
| ENSG00000175893 | ZDHHC21 | -1.127277285 | 4.27E-10 |
| ENSG00000145715 | RASA1 | -1.127244539 | 8.3E-22 |
| ENSG00000242622 | AC092910.3 | -1.126870256 | 0.00445916 |
| ENSG00000073711 | PPP2R3A | -1.125880001 | 0.000524284 |
| ENSG00000250312 | ZNF718 | -1.12520254 | 0.023312393 |
| ENSG00000178028 | DMAP1 | -1.124912699 | 4.52E-09 |
| ENSG00000198301 | SDAD1 | -1.12457039 | 2.8E-13 |
| ENSG00000111348 | ARHGDIB | -1.123667897 | 6.12E-11 |
| ENSG00000119541 | VPS4B | -1.123269228 | 1.04E-22 |
| ENSG00000138036 | DYNC2LI1 | -1.123009549 | 1.29E-11 |
| ENSG00000068001 | HYAL2 | -1.122950424 | 4.08E-15 |
| ENSG00000256235 | SMIM3 | -1.122830508 | 5.23E-16 |
| ENSG00000250571 | GLI4 | -1.122677611 | 0.0000646 |
| ENSG00000142632 | ARHGEF19 | -1.122659272 | 7.16E-23 |
| ENSG00000160188 | RSPH1 | -1.122227801 | 0.000160649 |
| ENSG00000162688 | AGL | -1.12203267 | 1.38E-11 |
| ENSG00000111271 | ACAD10 | -1.121222834 | 1.2E-10 |
| ENSG00000247271 | ZBED5-AS1 | -1.12064312 | 0.0000626 |
| ENSG00000139613 | SMARCC2 | -1.120185164 | 8.06E-18 |
| ENSG00000196227 | FAM217B | -1.119980133 | 3.25E-09 |
| ENSG00000171806 | METTL18 | -1.118815925 | 0.000000134 |
| ENSG00000125354 | 44445 | -1.118585581 | 0.009167017 |
| ENSG00000168890 | TMEM150A | -1.118391609 | 0.00000182 |
| ENSG00000196639 | HRH1 | -1.118314118 | 6.31E-08 |
| ENSG00000171492 | LRRC8D | -1.117852477 | 5.01E-16 |
| ENSG00000104783 | KCNN4 | -1.117346146 | 0.021254823 |
| ENSG00000135521 | LTV1 | -1.116391426 | 0.00000011 |
| ENSG00000213859 | KCTD11 | -1.115002949 | 2.74E-09 |
| ENSG00000198231 | DDX42 | -1.114920138 | 6.15E-18 |
| ENSG00000105672 | ETV2 | -1.113096755 | 0.000801011 |
| ENSG00000162227 | TAF6L | -1.113009272 | 8.34E-13 |
| ENSG00000280663 |  | -1.111889525 | 0.0000425 |
| ENSG00000137177 | KIF13A | -1.109922938 | 3.24E-13 |
| ENSG00000130713 | EXOSC2 | -1.109895262 | 1.99E-18 |
| ENSG00000120688 | WBP4 | -1.109705147 | 2.85E-15 |
| ENSG00000116560 | SFPQ | -1.109183033 | 1.17E-09 |
| ENSG00000133121 | STARD13 | -1.108526972 | 0.040453578 |
| ENSG00000088836 | SLC4A11 | -1.107896346 | 0.00029889 |
| ENSG00000260996 | BX255925.1 | -1.107781049 | 0.022973584 |
| ENSG00000142675 | CNKSR1 | -1.106668516 | 1.12E-09 |
| ENSG00000158286 | RNF207 | -1.106206472 | 0.0000964 |
| ENSG00000112659 | CUL9 | -1.106195922 | 1.78E-09 |
| ENSG00000142082 | SIRT3 | -1.10595795 | 5.23E-10 |
| ENSG00000174206 | C12orf66 | -1.105817661 | 0.000000118 |
| ENSG00000198353 | HOXC4 | -1.10564002 | 4.78E-12 |
| ENSG00000179029 | TMEM107 | -1.105079657 | 0.000000025 |
| ENSG00000126460 | PRRG2 | -1.104844669 | 5.62E-09 |
| ENSG00000063761 | ADCK1 | -1.104785472 | 0.000308769 |
| ENSG00000185090 | MANEAL | -1.104621844 | 6.61E-13 |
| ENSG00000136463 | TACO1 | -1.104190834 | 4.84E-20 |
| ENSG00000178229 | ZNF543 | -1.103752959 | 0.00000423 |
| ENSG00000267871 | ZNF460-AS1 | -1.103672754 | 0.0159153 |
| ENSG00000105072 | C19orf44 | -1.103280758 | 0.00000511 |
| ENSG00000221838 | AP4M1 | -1.102811142 | 8.29E-19 |
| ENSG00000016391 | CHDH | -1.10263045 | 4.16E-10 |
| ENSG00000255874 | LINC00346 | -1.101540271 | 0.000000685 |
| ENSG00000100578 | KIAA0586 | -1.101424399 | 1.04E-10 |
| ENSG00000260877 | AP005233.2 | -1.101365576 | 0.043518514 |
| ENSG00000142252 | GEMIN7 | -1.101334907 | 6.34E-11 |
| ENSG00000163714 | U2SURP | -1.10105341 | 2.07E-19 |
| ENSG00000163626 | COX18 | -1.100559242 | 1.98E-08 |
| ENSG00000172081 | MOB3A | -1.100227873 | 0.000000471 |
| ENSG00000180834 | MAP6D1 | -1.100132639 | 0.00894078 |
| ENSG00000176834 | VSIG10 | -1.096985322 | 1.09E-20 |
| ENSG00000263006 | ROCK1P1 | -1.09693663 | 0.004490488 |
| ENSG00000163138 | PACRGL | -1.096246238 | 2.13E-13 |
| ENSG00000112759 | SLC29A1 | -1.095929541 | 1.4E-22 |
| ENSG00000132911 | NMUR2 | -1.094640558 | 1.69E-08 |
| ENSG00000177000 | MTHFR | -1.094606075 | 3.6E-10 |
| ENSG00000106100 | NOD1 | -1.094590984 | 0.000000231 |
| ENSG00000101986 | ABCD1 | -1.094305325 | 5.69E-10 |
| ENSG00000173402 | DAG1 | -1.094099355 | 1.31E-23 |
| ENSG00000249673 | NOP14-AS1 | -1.09378251 | 0.0000015 |
| ENSG00000181027 | FKRP | -1.093526756 | 3.66E-09 |
| ENSG00000097046 | CDC7 | -1.093450064 | 4.45E-15 |
| ENSG00000174705 | SH3PXD2B | -1.092709028 | 0.017049808 |
| ENSG00000184436 | THAP7 | -1.091773604 | 1.15E-09 |
| ENSG00000124098 | FAM210B | -1.091278297 | 1.71E-09 |
| ENSG00000138152 | BTBD16 | -1.09127489 | 0.00000346 |
| ENSG00000186204 | CYP4F12 | -1.090953599 | 0.013920044 |
| ENSG00000198917 | SPOUT1 | -1.090621136 | 3.37E-23 |
| ENSG00000120800 | UTP20 | -1.090031002 | 0.00000413 |
| ENSG00000280071 | FP565260.6 | -1.088915266 | 8.28E-08 |
| ENSG00000160447 | PKN3 | -1.088721639 | 4.19E-15 |
| ENSG00000108469 | RECQL5 | -1.088314617 | 1.3E-13 |
| ENSG00000176884 | GRIN1 | -1.087959669 | 0.012620634 |
| ENSG00000156172 | C8orf37 | -1.08794057 | 0.000774762 |
| ENSG00000150455 | TIRAP | -1.087224293 | 4.74E-08 |
| ENSG00000198734 | F5 | -1.087155222 | 0.020778121 |
| ENSG00000023516 | AKAP11 | -1.087124358 | 8.95E-12 |
| ENSG00000118276 | B4GALT6 | -1.08596266 | 8.71E-09 |
| ENSG00000154330 | PGM5 | -1.085289974 | 0.026259396 |
| ENSG00000198894 | CIPC | -1.085267102 | 6.29E-14 |
| ENSG00000104884 | ERCC2 | -1.084255339 | 7.23E-17 |
| ENSG00000267278 | MAP3K14-AS1 | -1.084228259 | 0.001890328 |
| ENSG00000203705 | TATDN3 | -1.083962083 | 2.63E-10 |
| ENSG00000141564 | RPTOR | -1.083446998 | 1.38E-13 |
| ENSG00000092847 | AGO1 | -1.082901194 | 1.2E-13 |
| ENSG00000114248 | LRRC31 | -1.082827842 | 0.000100529 |
| ENSG00000157388 | CACNA1D | -1.082104735 | 0.000842583 |
| ENSG00000215447 |  | -1.080867708 | 0.00073254 |
| ENSG00000126790 | L3HYPDH | -1.080826908 | 0.011850485 |
| ENSG00000179943 | FIZ1 | -1.080221403 | 4.56E-14 |
| ENSG00000274675 |  | -1.079608566 | 0.001576191 |
| ENSG00000119401 | TRIM32 | -1.079298714 | 1.74E-19 |
| ENSG00000078900 | TP73 | -1.079040026 | 4.57E-08 |
| ENSG00000169994 | MYO7B | -1.078968912 | 0.000412245 |
| ENSG00000016402 | IL20RA | -1.078863149 | 1.58E-08 |
| ENSG00000164764 | SBSPON | -1.078741031 | 2.32E-11 |
| ENSG00000245849 | RAD51-AS1 | -1.078531598 | 0.000816679 |
| ENSG00000136827 | TOR1A | -1.078155083 | 5.6E-22 |
| ENSG00000166166 | TRMT61A | -1.077542489 | 2.53E-09 |
| ENSG00000183778 | B3GALT5 | -1.076897858 | 0.00000458 |
| ENSG00000177082 | WDR73 | -1.075927915 | 0.00000222 |
| ENSG00000165516 | KLHDC2 | -1.075721695 | 4.44E-12 |
| ENSG00000198944 | SOWAHA | -1.075079923 | 0.002086228 |
| ENSG00000261799 | AC007406.5 | -1.074985306 | 0.011912568 |
| ENSG00000083097 | DOPEY1 | -1.074902802 | 0.0000341 |
| ENSG00000160404 | TOR2A | -1.074584624 | 2.04E-11 |
| ENSG00000114395 | CYB561D2 | -1.074420333 | 0.0000088 |
| ENSG00000150990 | DHX37 | -1.072736917 | 4.69E-13 |
| ENSG00000127831 | VIL1 | -1.072317499 | 6.44E-12 |
| ENSG00000114126 | TFDP2 | -1.071522961 | 2.52E-09 |
| ENSG00000168887 | C2orf68 | -1.071514405 | 3.81E-18 |
| ENSG00000138172 | CALHM2 | -1.070822385 | 0.000000113 |
| ENSG00000106066 | CPVL | -1.07040703 | 0.024411139 |
| ENSG00000242366 | UGT1A8 | -1.069959255 | 0.000000384 |
| ENSG00000261762 | AC027228.2 | -1.068560371 | 0.00309146 |
| ENSG00000129173 | E2F8 | -1.068468057 | 0.000258386 |
| ENSG00000031003 | FAM13B | -1.068244596 | 1.19E-10 |
| ENSG00000180801 | ARSJ | -1.068155183 | 8.5E-16 |
| ENSG00000112624 | BICRAL | -1.067634475 | 4E-11 |
| ENSG00000232838 | PET117 | -1.067300741 | 0.001415462 |
| ENSG00000159267 | HLCS | -1.06721238 | 3.74E-11 |
| ENSG00000121851 | POLR3GL | -1.066921242 | 1.14E-08 |
| ENSG00000139910 | NOVA1 | -1.066650297 | 0.013496034 |
| ENSG00000167264 | DUS2 | -1.066537552 | 5E-10 |
| ENSG00000263731 | AC145207.5 | -1.066339089 | 0.048703125 |
| ENSG00000081386 | ZNF510 | -1.065340476 | 8.15E-10 |
| ENSG00000137547 | MRPL15 | -1.065094837 | 1.54E-12 |
| ENSG00000119514 | GALNT12 | -1.06500435 | 9.66E-23 |
| ENSG00000128915 | ICE2 | -1.064996687 | 6.51E-22 |
| ENSG00000253161 | LINC01605 | -1.064784314 | 0.047088266 |
| ENSG00000156162 | DPY19L4 | -1.06458651 | 5.47E-18 |
| ENSG00000113108 | APBB3 | -1.06419467 | 0.00000797 |
| ENSG00000101040 | ZMYND8 | -1.064015855 | 1.01E-08 |
| ENSG00000204920 | ZNF155 | -1.063408548 | 0.000484154 |
| ENSG00000106804 | C5 | -1.062285258 | 0.003439426 |
| ENSG00000257594 | GALNT4 | -1.061854541 | 0.027543276 |
| ENSG00000149809 | TM7SF2 | -1.061668837 | 5.89E-08 |
| ENSG00000172037 | LAMB2 | -1.061471562 | 0.0000224 |
| ENSG00000162755 | KLHDC9 | -1.060766831 | 0.009727234 |
| ENSG00000197226 | TBC1D9B | -1.060533284 | 3.83E-18 |
| ENSG00000101346 | POFUT1 | -1.060355104 | 3.19E-27 |
| ENSG00000119125 | GDA | -1.059685439 | 1.25E-10 |
| ENSG00000138399 | FASTKD1 | -1.059409117 | 0.000000462 |
| ENSG00000171428 | NAT1 | -1.059360769 | 0.013209151 |
| ENSG00000136492 | BRIP1 | -1.05899184 | 0.0000012 |
| ENSG00000118894 | EEF2KMT | -1.058684494 | 1.44E-08 |
| ENSG00000154930 | ACSS1 | -1.058562633 | 6.05E-14 |
| ENSG00000266469 | AC005288.1 | -1.05855717 | 6.08E-08 |
| ENSG00000239521 | CASTOR3 | -1.058472278 | 0.000000884 |
| ENSG00000151413 | NUBPL | -1.058412206 | 0.000000133 |
| ENSG00000116906 | GNPAT | -1.058300886 | 5.49E-15 |
| ENSG00000169583 | CLIC3 | -1.057228606 | 0.0000103 |
| ENSG00000185024 | BRF1 | -1.05694084 | 2.39E-18 |
| ENSG00000177854 | TMEM187 | -1.056820785 | 8.34E-11 |
| ENSG00000166272 | WBP1L | -1.05625404 | 1.95E-22 |
| ENSG00000116005 | PCYOX1 | -1.055186537 | 2.67E-17 |
| ENSG00000233834 | AC005083.1 | -1.054725242 | 0.001219887 |
| ENSG00000146733 | PSPH | -1.053983182 | 2.45E-14 |
| ENSG00000136098 | NEK3 | -1.053386873 | 0.00000243 |
| ENSG00000236515 |  | -1.052864259 | 0.027615183 |
| ENSG00000280832 | GSEC | -1.052809707 | 0.0000405 |
| ENSG00000016864 | GLT8D1 | -1.052353992 | 1.48E-15 |
| ENSG00000164056 | SPRY1 | -1.051520441 | 5.99E-09 |
| ENSG00000111057 | KRT18 | -1.051178035 | 3.63E-12 |
| ENSG00000079691 | CARMIL1 | -1.051118472 | 1.48E-13 |
| ENSG00000166529 | ZSCAN21 | -1.05054148 | 0.00000248 |
| ENSG00000147679 | UTP23 | -1.050398635 | 4.39E-25 |
| ENSG00000186130 | ZBTB6 | -1.049984787 | 4.46E-16 |
| ENSG00000110931 | CAMKK2 | -1.048568399 | 3.83E-19 |
| ENSG00000279672 | AP006621.5 | -1.047995439 | 0.008178264 |
| ENSG00000224051 | CPTP | -1.045651093 | 2.31E-15 |
| ENSG00000151576 | QTRT2 | -1.045068742 | 1.56E-09 |
| ENSG00000083812 | ZNF324 | -1.045016865 | 1.85E-13 |
| ENSG00000215424 | MCM3AP-AS1 | -1.04434184 | 0.000231519 |
| ENSG00000240857 | RDH14 | -1.044323033 | 1.02E-08 |
| ENSG00000165832 | TRUB1 | -1.043968827 | 1.86E-17 |
| ENSG00000197296 | FITM2 | -1.043708794 | 9.7E-13 |
| ENSG00000171103 | TRMT61B | -1.043146175 | 1.34E-13 |
| ENSG00000120159 | CAAP1 | -1.041702258 | 9.51E-23 |
| ENSG00000235280 | MCF2L-AS1 | -1.041635 | 0.001071731 |
| ENSG00000101347 | SAMHD1 | -1.041374093 | 0.0000705 |
| ENSG00000053900 | ANAPC4 | -1.041019357 | 1.28E-14 |
| ENSG00000110002 | VWA5A | -1.040943503 | 0.018924924 |
| ENSG00000156398 | SFXN2 | -1.040567771 | 6.7E-09 |
| ENSG00000100324 | TAB1 | -1.039655788 | 1.48E-15 |
| ENSG00000144182 | LIPT1 | -1.039439715 | 0.000717487 |
| ENSG00000108784 | NAGLU | -1.038398854 | 8.95E-14 |
| ENSG00000184508 | HDDC3 | -1.037110161 | 0.0000157 |
| ENSG00000165140 | FBP1 | -1.035385504 | 8.41E-12 |
| ENSG00000157036 | EXOG | -1.034740025 | 0.007077887 |
| ENSG00000135763 | URB2 | -1.034415669 | 7.79E-12 |
| ENSG00000143036 | SLC44A3 | -1.033893765 | 5.55E-13 |
| ENSG00000135540 | NHSL1 | -1.033724697 | 0.00000922 |
| ENSG00000163933 | RFT1 | -1.033521755 | 1.96E-13 |
| ENSG00000232434 | AJM1 | -1.030851888 | 7.1E-10 |
| ENSG00000083750 | RRAGB | -1.029624234 | 0.0000158 |
| ENSG00000154920 | EME1 | -1.02927406 | 0.00000134 |
| ENSG00000160226 | C21orf2 | -1.029144719 | 0.00010848 |
| ENSG00000070778 | PTPN21 | -1.029114738 | 0.000000485 |
| ENSG00000006530 | AGK | -1.028607213 | 3.94E-08 |
| ENSG00000174808 | BTC | -1.028234817 | 0.00001 |
| ENSG00000174292 | TNK1 | -1.027904255 | 1.33E-08 |
| ENSG00000151883 | PARP8 | -1.027860963 | 8.85E-15 |
| ENSG00000153944 | MSI2 | -1.027489838 | 7.24E-12 |
| ENSG00000091732 | ZC3HC1 | -1.027387801 | 1.73E-11 |
| ENSG00000127995 | CASD1 | -1.027252907 | 1.07E-12 |
| ENSG00000100106 | TRIOBP | -1.026860284 | 6.04E-17 |
| ENSG00000104299 | INTS9 | -1.026836189 | 0.00000667 |
| ENSG00000157426 | AASDH | -1.026824586 | 2E-10 |
| ENSG00000204860 | FAM201A | -1.026824358 | 0.000031 |
| ENSG00000158555 | GDPD5 | -1.026810903 | 0.0000431 |
| ENSG00000239605 | STPG4 | -1.02658727 | 0.001008837 |
| ENSG00000185133 | INPP5J | -1.026571322 | 0.030304995 |
| ENSG00000271335 | AL117336.3 | -1.026370098 | 0.015861111 |
| ENSG00000178896 | EXOSC4 | -1.025679154 | 4.07E-08 |
| ENSG00000162377 | COA7 | -1.025119313 | 8.25E-13 |
| ENSG00000125378 | BMP4 | -1.025074807 | 9.48E-13 |
| ENSG00000186417 | GLDN | -1.024648282 | 0.001001837 |
| ENSG00000012174 | MBTPS2 | -1.024292109 | 1.34E-08 |
| ENSG00000123297 | TSFM | -1.023987646 | 5.14E-12 |
| ENSG00000070614 | NDST1 | -1.022823613 | 2.28E-11 |
| ENSG00000176623 | RMDN1 | -1.022607026 | 8.61E-12 |
| ENSG00000175938 | ORAI3 | -1.022138092 | 0.000533035 |
| ENSG00000180573 | HIST1H2AC | -1.021450669 | 0.000154422 |
| ENSG00000160345 | C9orf116 | -1.020850922 | 0.002777899 |
| ENSG00000204876 | AC021218.1 | -1.020081745 | 1.53E-09 |
| ENSG00000164010 | ERMAP | -1.019817384 | 8.09E-08 |
| ENSG00000141076 | UTP4 | -1.019693671 | 3.84E-10 |
| ENSG00000130768 | SMPDL3B | -1.019573548 | 0.0000105 |
| ENSG00000185278 | ZBTB37 | -1.019120446 | 0.001408797 |
| ENSG00000239415 | AP001469.3 | -1.019120373 | 0.011667671 |
| ENSG00000121316 | PLBD1 | -1.018265416 | 0.000343884 |
| ENSG00000185515 | BRCC3 | -1.018172828 | 1.55E-11 |
| ENSG00000131771 | PPP1R1B | -1.017475379 | 4.11E-09 |
| ENSG00000182557 | SPNS3 | -1.017096678 | 0.001949541 |
| ENSG00000183734 | ASCL2 | -1.016117472 | 0.00000168 |
| ENSG00000179041 | RRS1 | -1.016097932 | 0.00000465 |
| ENSG00000182318 | ZSCAN22 | -1.016077567 | 0.000000145 |
| ENSG00000173548 | SNX33 | -1.015880539 | 5.73E-13 |
| ENSG00000144730 | IL17RD | -1.015290658 | 0.037295296 |
| ENSG00000249550 | LINC01234 | -1.014776716 | 0.00000506 |
| ENSG00000161277 | THAP8 | -1.013783583 | 0.000103949 |
| ENSG00000166578 | IQCD | -1.013580999 | 0.001616582 |
| ENSG00000197779 | ZNF81 | -1.013308441 | 0.000304737 |
| ENSG00000114405 | C3orf14 | -1.013016189 | 0.021449224 |
| ENSG00000198624 | CCDC69 | -1.01301514 | 0.002084029 |
| ENSG00000204519 | ZNF551 | -1.011971426 | 7.74E-12 |
| ENSG00000180902 | D2HGDH | -1.011893063 | 2.23E-09 |
| ENSG00000079974 | RABL2B | -1.01096879 | 0.0000756 |
| ENSG00000175395 | ZNF25 | -1.01061419 | 0.030905612 |
| ENSG00000166971 | AKTIP | -1.010389244 | 4.1E-12 |
| ENSG00000127399 | LRRC61 | -1.010328683 | 2.39E-08 |
| ENSG00000185219 | ZNF445 | -1.010123776 | 0.00000112 |
| ENSG00000137642 | SORL1 | -1.010001995 | 0.00000252 |
| ENSG00000106246 | PTCD1 | -1.0098898 | 0.000421609 |
| ENSG00000071242 | RPS6KA2 | -1.009510861 | 0.018682632 |
| ENSG00000073910 | FRY | -1.009237988 | 0.005535296 |
| ENSG00000136100 | VPS36 | -1.008621414 | 7.22E-11 |
| ENSG00000141337 | ARSG | -1.008026415 | 0.022922078 |
| ENSG00000185418 | TARSL2 | -1.007973057 | 2.56E-08 |
| ENSG00000165698 | SPACA9 | -1.007927455 | 0.000343768 |
| ENSG00000178921 | PFAS | -1.007733293 | 1.18E-08 |
| ENSG00000255455 | AP003486.1 | -1.007604685 | 0.005821234 |
| ENSG00000169740 | ZNF32 | -1.006577735 | 0.000000633 |
| ENSG00000134240 | HMGCS2 | -1.005267372 | 0.000000329 |
| ENSG00000178878 | APOLD1 | -1.004851566 | 0.0000413 |
| ENSG00000163472 | TMEM79 | -1.00456909 | 0.0000264 |
| ENSG00000166143 | PPP1R14D | -1.004004059 | 0.031176127 |
| ENSG00000196730 | DAPK1 | -1.003666269 | 2.79E-12 |
| ENSG00000223745 | CCDC18-AS1 | -1.003628503 | 0.002417703 |
| ENSG00000255135 | AP002360.1 | -1.003359239 | 0.0000474 |
| ENSG00000181915 | ADO | -1.003280059 | 1.83E-15 |
| ENSG00000103034 | NDRG4 | -1.002970406 | 0.016742663 |
| ENSG00000027001 | MIPEP | -1.002889491 | 0.00000144 |
| ENSG00000100201 | DDX17 | -1.002466529 | 1.7E-21 |
| ENSG00000107821 | KAZALD1 | -1.002180486 | 0.00000615 |
| ENSG00000197299 | BLM | -1.001953106 | 9.56E-09 |
| ENSG00000148814 | LRRC27 | -1.001913788 | 0.000304222 |
| ENSG00000143374 | TARS2 | -1.001680098 | 5.86E-13 |
| ENSG00000162777 | DENND2D | -1.001581696 | 7.03E-19 |
| ENSG00000124193 | SRSF6 | -1.001432068 | 4.5E-18 |
| ENSG00000109452 | INPP4B | -1.000755485 | 0.00000137 |
| ENSG00000092964 | DPYSL2 | -1.000648987 | 4.64E-09 |
| ENSG00000163161 | ERCC3 | -1.000604801 | 3.98E-15 |
| ENSG00000136161 | RCBTB2 | -1.000150338 | 0.005003188 |
| ENSG00000103550 | KNOP1 | -0.999999372 | 1.35E-12 |
[truncated: 138,997 more chars]
