# Supplementary material for: Targeting oxeiptosis-mediated tumor suppression: a novel approach to treat colorectal cancers by sanguinarine
Source: Cell Death Discov. 2023 Mar 13;9:94. doi: 10.1038/s41420-023-01376-3 (PMC10011521; doi:10.1038/s41420-023-01376-3)
Supplement: Supplementary file 3 — Supplemental Table S2 [file 41420_2023_1376_MOESM3_ESM.docx]

|  |  |  |  |  |
| --- | --- | --- | --- | --- |
| **Supplemental Table S2.** | | | |  |
|  |  |  |  |  |
| **List of differentially expressed cell death associated genes** | | | | |
| **by SNG in the presence or absence of NAC** | | | |  |
|  |  |  |  |  |
| gene | Control | SNG | SNG+NAC |  |
| CASP3 | 0 | 0.99269624 | 0.09461197 |  |
| BCL2L15 | 0 | -2.2385712 | -0.7927503 |  |
| NFKB1 | 0 | 0.52582511 | -0.8835161 |  |
| NFKBID | 0 | 1.13138115 | 0.52830477 |  |
| CASP7 | 0 | 0.98693083 | 0.29797982 |  |
| BAX | 0 | 0.6844366 | 0.20312563 |  |
| TNFRSF10B | 0 | 1.45068741 | -0.001458 |  |
| TNFRSF10D | 0 | 1.02927098 | 0.51679107 |  |
| GADD45A | 0 | 2.30789727 | -0.6118426 |  |
| APAF1 | 0 | 0.41137223 | 0.32311006 |  |
| BAK1 | 0 | 1.33420926 | 0.18929659 |  |
| TRAF3 | 0 | 0.32672273 | -0.291183 |  |
| BIK | 0 | 1.10492128 | 0.57055366 |  |
| AKT2 | 0 | -0.4647146 | -0.1380257 |  |
| ATM | 0 | -0.3984136 | 0.62435611 |  |
| IKBKB | 0 | -0.8775419 | -0.0008076 |  |
| NFKBIA | 0 | 3.35598967 | 0.33186383 |  |
| AIFM2 | 0 | 1.55796523 | 0.45576802 |  |
| CTSL | 0 | 2.69615853 | 0.92701437 |  |
| PARP2 | 0 | 1.2268619 | 0.52496147 |  |
| CYCS | 0 | 1.03919593 | -0.0277184 |  |
| DIABLO | 0 | 0.73704188 | 0.09661987 |  |
| BID | 0 | 0.44696175 | -0.298944 |  |
| PAWR | 0 | 0.82657363 | 0.03824434 |  |
| RAB7A | 0 | 1.07154446 | 0.09569604 |  |
| LAMP2 | 0 | 1.29326964 | 0.73423953 |  |
| LAMP1 | 0 | 0.70101435 | 0.07711692 |  |
| ATG4D | 0 | 0.81480581 | 0.62443277 |  |
| ATG4A | 0 | 0.93004352 | 0.25716665 |  |
| GABARAPL2 | 0 | 0.75628171 | 0.27860249 |  |
| GABARAPL1 | 0 | 3.74294985 | 1.49254013 |  |
| ATG3 | 0 | 0.60928142 | 0.01919753 |  |
| ATG5 | 0 | 0.45047215 | 0.08803309 |  |
| ATG12 | 0 | 0.75851966 | 0.23808662 |  |
| ZFYVE1 | 0 | 1.42738848 | 0.97963955 |  |
| HMGB1 | 0 | 0.46719147 | -0.3637636 |  |
| DAPK2 | 0 | 1.89051088 | 2.15400145 |  |
| ERN1 | 0 | 2.20712275 | 0.31718539 |  |
| UVRAG | 0 | 0.95886371 | 0.34709353 |  |
| AMBRA1 | 0 | 0.57027394 | -0.1105133 |  |
| ATG14 | 0 | 0.65622909 | 0.22759135 |  |
| SQSTM1 | 0 | 3.12385788 | 1.46109624 |  |
| ATG9A | 0 | 0.55975155 | 0.42971717 |  |
| ATG101 | 0 | 1.09157147 | 0.04993368 |  |
| ULK1 | 0 | 1.09022188 | 0.97150789 |  |
| MTOR | 0 | -0.2671303 | 0.1641255 |  |
| RPTOR | 0 | -1.083447 | -0.4303259 |  |
| BNIP3 | 0 | -0.8941217 | -0.0888205 |  |
| HIF1A | 0 | 0.95672032 | 0.04919998 |  |
| TRAF2 | 0 | 0.94687731 | 0.11162625 |  |
| RIPK1 | 0 | -1.5446288 | -0.3637899 |  |
| CASP8 | 0 | -0.6747027 | -0.3235146 |  |
| FADD | 0 | -1.9394311 | -0.3412092 |  |
| CFLAR | 0 | 0.46120489 | 0.12840558 |  |
| SLC25A6 | 0 | 0.60645772 | 0.09766331 |  |
| VDAC1 | 0 | 0.50614418 | 0.00807726 |  |
| MLKL | 0 | -0.8641457 | -0.6630724 |  |
| VPS4B | 0 | -1.1232692 | -0.1962183 |  |
| VPS4A | 0 | -0.5779413 | -0.2810698 |  |
| FAS | 0 | 0.53993618 | 0.15419468 |  |
| IFNGR2 | 0 | 2.01781124 | 0.03412902 |  |
| IFNB1 | 0 | 6.53421343 | 3.45964516 |  |
| JAK1 | 0 | 1.47385635 | 0.41661777 |  |
| STAT1 | 0 | 0.36536205 | 0.2895664 |  |
| STAT3 | 0 | 0.37083647 | 0.13736256 |  |
| IRF9 | 0 | 2.35125156 | 1.13296672 |  |
| EIF2AK2 | 0 | 0.5179201 | 0.34371151 |  |
| HSP90AA1 | 0 | 2.68412347 | 0.22280185 |  |
| HSP90AB1 | 0 | 1.82433384 | 0.77808531 |  |
| TNFAIP3 | 0 | 7.48733488 | 1.64000509 |  |
| SLC3A2 | 0 | 1.20307043 | -0.3754154 |  |
| GCLC | 0 | 1.96103354 | 1.42206654 |  |
| GCLM | 0 | 3.2865039 | 1.63476012 |  |
| GSS | 0 | -0.2786943 | -0.2434294 |  |
| GPX4 | 0 | 1.2237951 | 0.3920953 |  |
| ACSL4 | 0 | 0.76828582 | 0.32417561 |  |
| TFRC | 0 | 0.26699516 | 0.05183737 |  |
| SLC39A8 | 0 | -0.8412633 | -0.6362514 |  |
| SLC39A14 | 0 | 0.53625929 | 0.05448923 |  |
| SLC40A1 | 0 | -0.4118279 | 0.23166402 |  |
| PCBP1 | 0 | 0.39499589 | 0.0146908 |  |
| FTH1 | 0 | 2.93770086 | 2.03935646 |  |
| FTL | 0 | 2.72841243 | 1.92446736 |  |
| HMOX1 | 0 | 7.3795621 | 2.53402373 |  |
| VDAC2 | 0 | 0.60336674 | 0.14794311 |  |
| PGAM5 | 0 | 0.30896692 | -0.2752611 |  |
|  |  |  |  |  |
|  |  |  |  |  |
|  |  |  |  |  |
|  |  |  |  |  |
|  |  |  |  |  |
|  |  |  |  |  |
|  |  |  |  |  |
