# Supplementary material for: Targeting oxeiptosis-mediated tumor suppression: a novel approach to treat colorectal cancers by sanguinarine
Source: Cell Death Discov. 2023 Mar 13;9:94. doi: 10.1038/s41420-023-01376-3 (PMC10011521; doi:10.1038/s41420-023-01376-3)
Supplement: Supplementary file 4 — Supplemental Table S3 [file 41420_2023_1376_MOESM4_ESM.docx]

|  |  |  |  |  |
| --- | --- | --- | --- | --- |
| **Supplemental Table S3.** | | | |  |
|  |  |  |  |  |
| **List of differentially expressed oxidative stress genes** | | | | |
| **by SNG in the presence or absence of NAC** | | | |  |
|  |  |  |  |  |
|  |  |  |  |  |
| gene | control | SNG | SNG+NAC |  |
| NQO1 | 0 | 1.07299581 | 0.76691107 |  |
| SOD2 | 0 | 2.50154556 | -0.0185168 |  |
| GPX4 | 0 | 1.2237951 | 0.3920953 |  |
| PRDX4 | 0 | 0.39802129 | -0.0257503 |  |
| PRDX1 | 0 | 0.90395105 | 0.42115158 |  |
| PRDX6 | 0 | 0.63409915 | -0.0269869 |  |
| PRDX5 | 0 | 0.63279319 | 0.31424254 |  |
| GPX2 | 0 | 0.31549291 | -0.0050749 |  |
| GSR | 0 | 1.35501136 | 0.53266446 |  |
| GSTP1 | 0 | 0.68284818 | 0.06900932 |  |
| ALOXE3 | 0 | 4.46109722 | -0.6982497 |  |
| GPX1 | 0 | 0.6518802 | -0.3217583 |  |
| GPX3 | 0 | 1.00712435 | 0.83409544 |  |
| SOD1 | 0 | 1.36637188 | 0.11896738 |  |
| TXN | 0 | 1.66993836 | 0.58468428 |  |
| TXNIP | 0 | 1.55992669 | 1.4398571 |  |
| TXNRD1 | 0 | 2.13288708 | 0.86397882 |  |
| GCLM | 0 | 3.2865039 | 1.63476012 |  |
| FTH1 | 0 | 2.93770086 | 2.03935646 |  |
| CYBA | 0 | 0.2762082 | -0.2951409 |  |
| CYB5R4 | 0 | 0.33724533 | -0.2396489 |  |
| FOXO1 | 0 | 1.41238715 | -0.5028679 |  |
| FOXO3 | 0 | 1.43290163 | 0.28497898 |  |
| NCF2 | 0 | 3.62268348 | 0.28613256 |  |
| ROMO1 | 0 | 0.49475808 | -0.0221859 |  |
| TUSC2 | 0 | 0.47507307 | 0.09893279 |  |
| SQSTM1 | 0 | 3.12385788 | 1.46109624 |  |
| RNF112 | 0 | 3.95848064 | 3.96586756 |  |
| HMOX1 | 0 | 7.3795621 | 2.53402373 |  |
| GPX8 | 0 | -1.5854699 | 0.11425414 |  |
| CCS | 0 | -0.5162461 | 0.15508063 |  |
| STK25 | 0 | -0.2507366 | -0.0061573 |  |
| VRK2 | 0 | -0.4605706 | -0.2807684 |  |
| EDN1 | 0 | -2.7745286 | -0.8653174 |  |
| EGFR | 0 | -0.5368911 | -0.6630056 |  |
| NOXA1 | 0 | -1.1327721 | 0.15853665 |  |
| MSRB2 | 0 | -0.286341 | -0.1138109 |  |
| NCOA7 | 0 | -0.3242392 | -0.0458143 |  |
| PXDN | 0 | -2.3597472 | 0.21340381 |  |
| KEAP1 | 0 | -0.5223232 | -0.0750365 |  |
| TXNRD3 | 0 | -1.4401905 | -0.3136058 |  |
| TXNRD2 | 0 | -0.2591528 | -0.214228 |  |
| TXNDC9 | 0 | -0.5992021 | -0.3350952 |  |
| GSTK1 | 0 | -0.4118962 | -0.0991142 |  |
| ISG15 | 0 | 4.29982528 | 0.39383761 |  |
| KLF4 | 0 | 3.73752887 | 0.46509055 |  |
| HERC5 | 0 | 6.84846117 | 1.96292922 |  |
| PGAM5 | 0 | 0.30896692 | -0.2752611 |  |
| NOX1 | 0 | -2.9565115 | -3.016104 |  |
|  |  |  |  |  |
|  |  |  |  |  |
|  |  |  |  |  |
|  |  |  |  |  |
|  |  |  |  |  |
