# Supplementary material for: Targeting oxeiptosis-mediated tumor suppression: a novel approach to treat colorectal cancers by sanguinarine
Source: Cell Death Discov. 2023 Mar 13;9:94. doi: 10.1038/s41420-023-01376-3 (PMC10011521; doi:10.1038/s41420-023-01376-3)

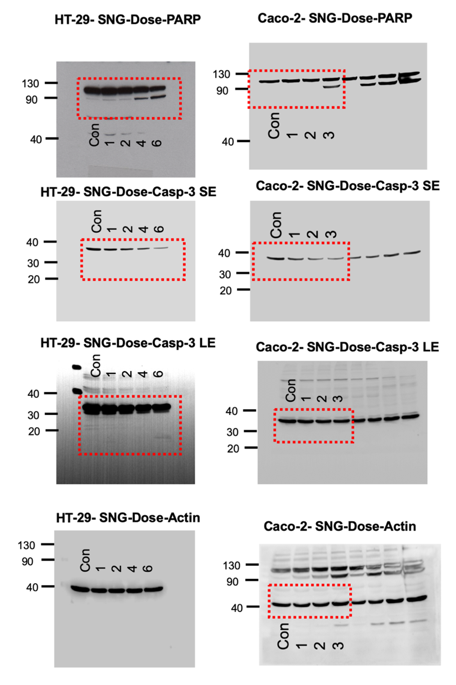
**Fig 2 B**

**
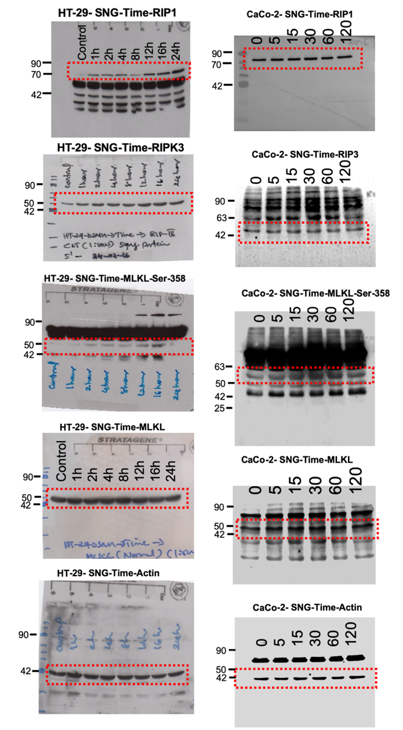
Fig 2 C**

**Fig 2 E**

**
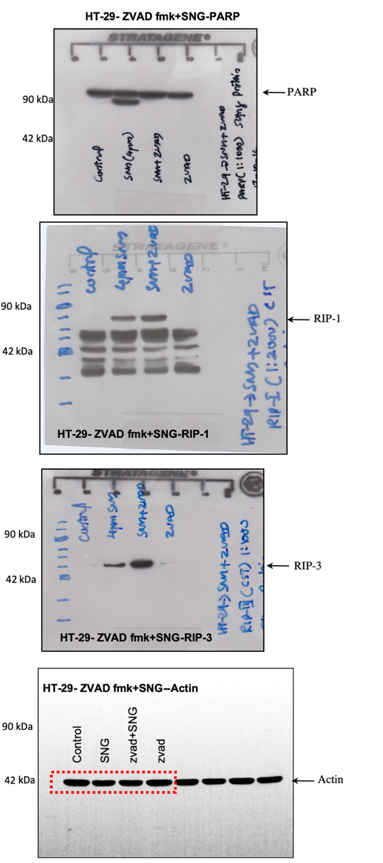
**


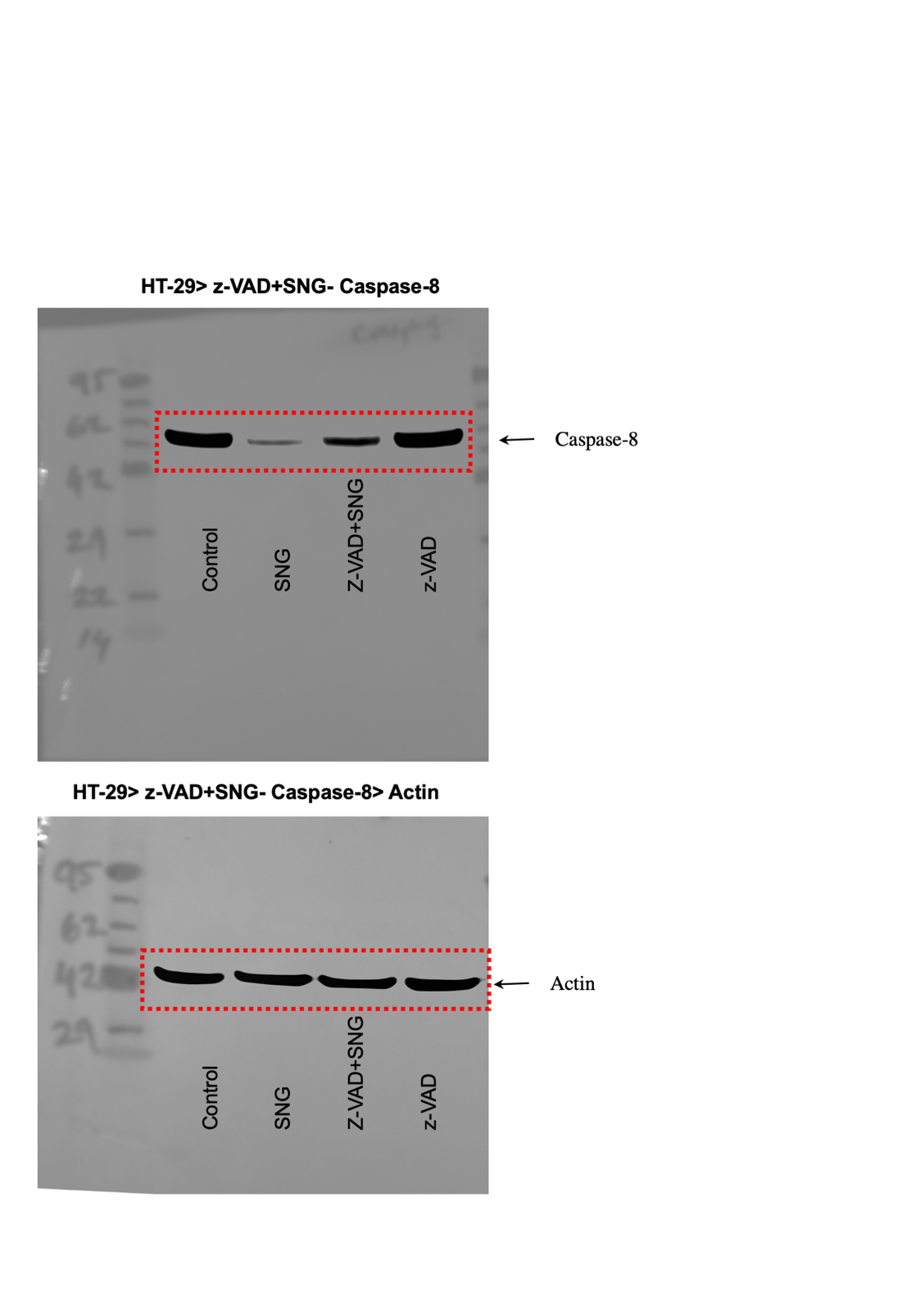


**
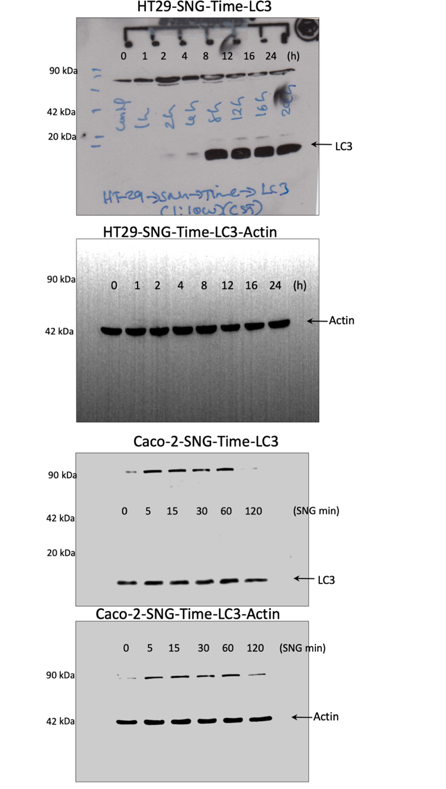
Fig 2 H**

**
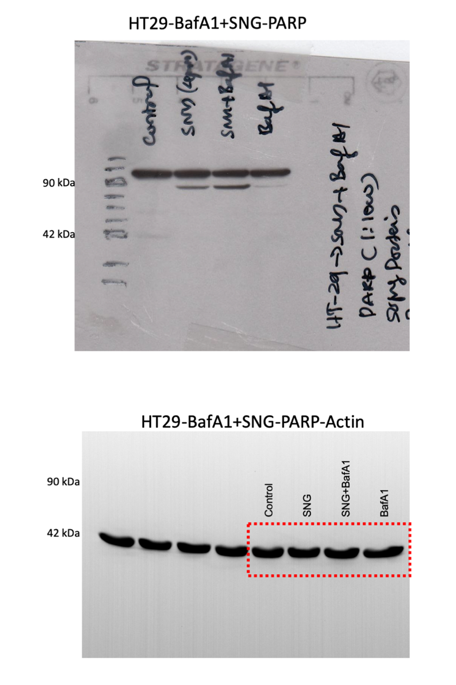
Fig 2 J**

**
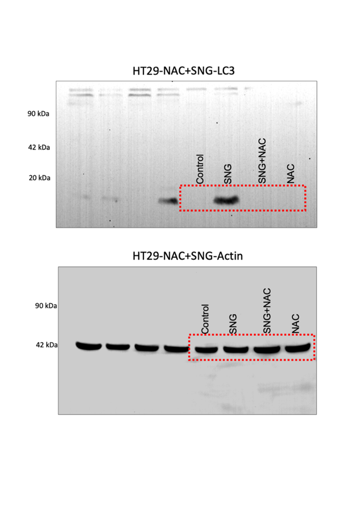

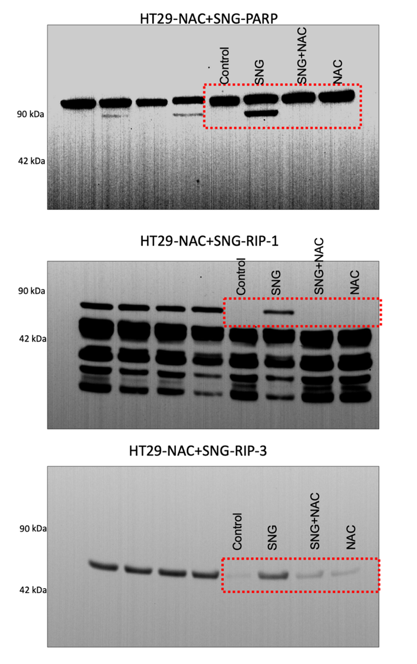
Fig 3 F**

**Fig 5A**


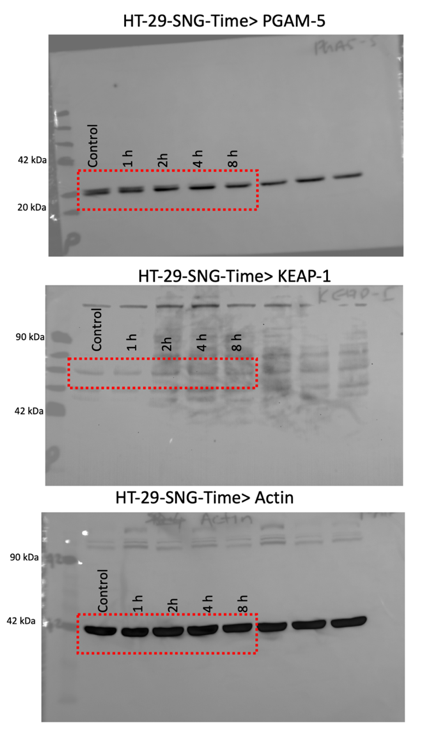


**Fig 5 B**


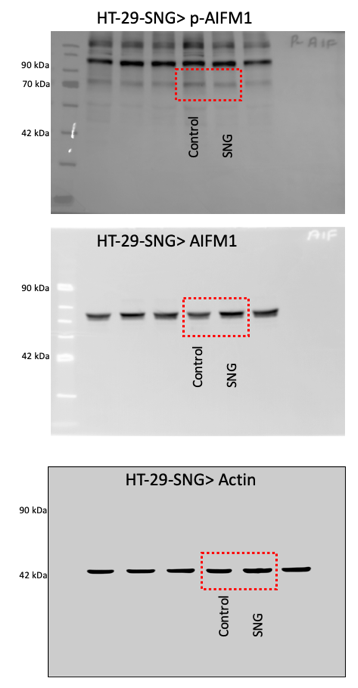

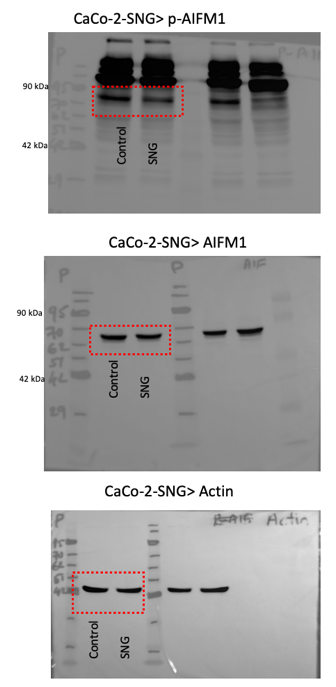


**Fig 5 C**


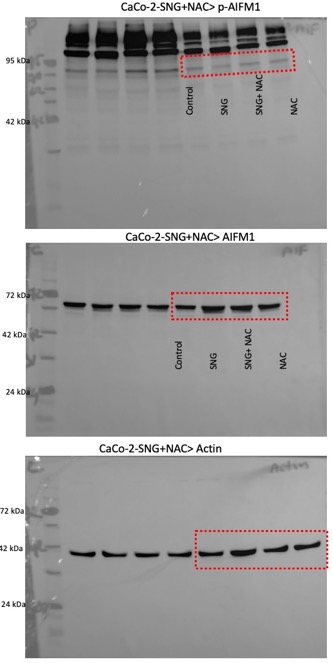

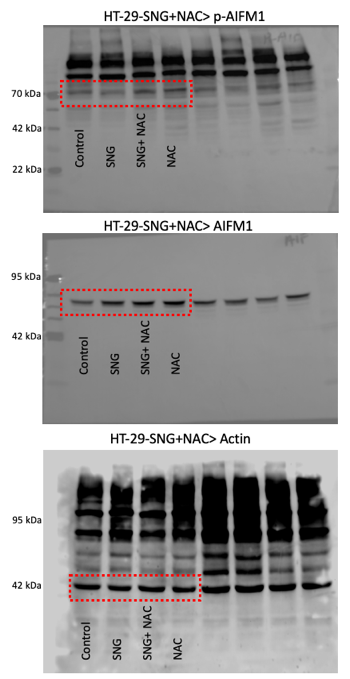


**Fig 5 F**


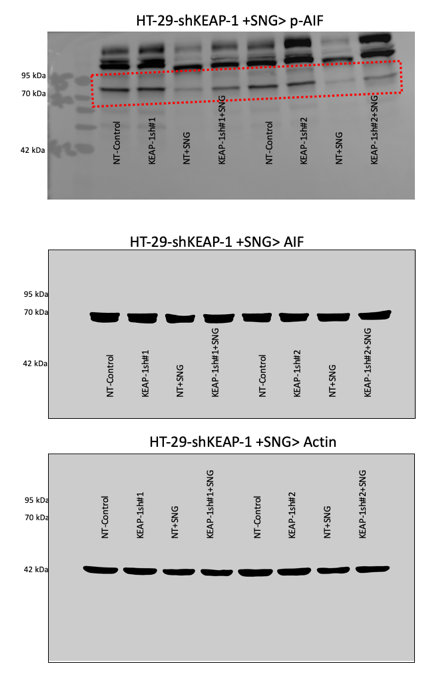


**
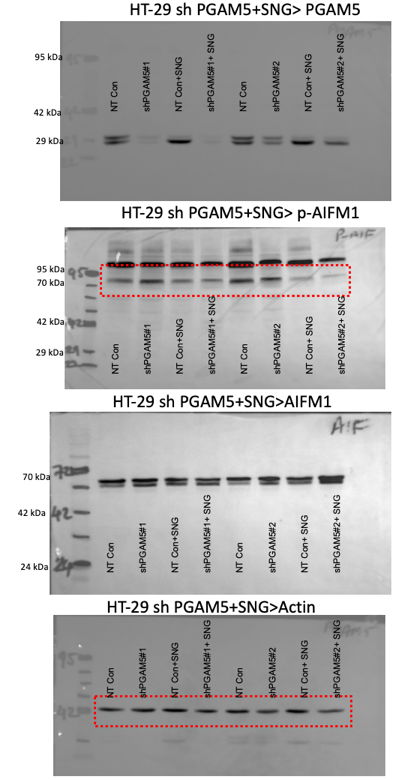
Fig 6 A**

**
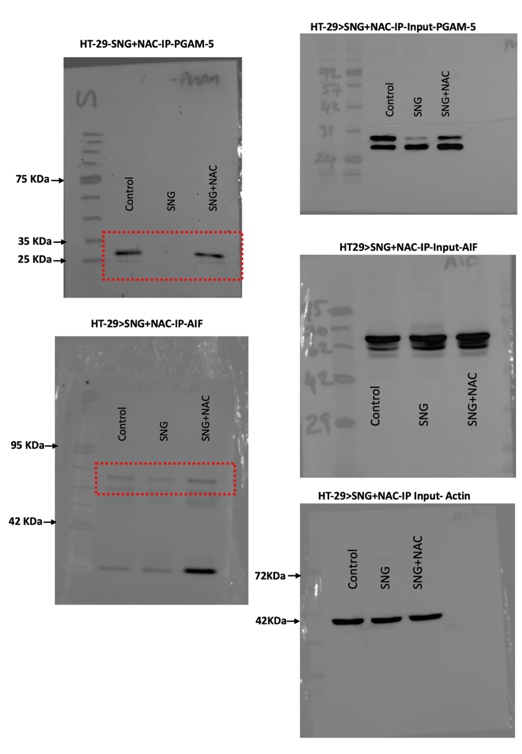
Fig 6 G**

**Fig 6 H**


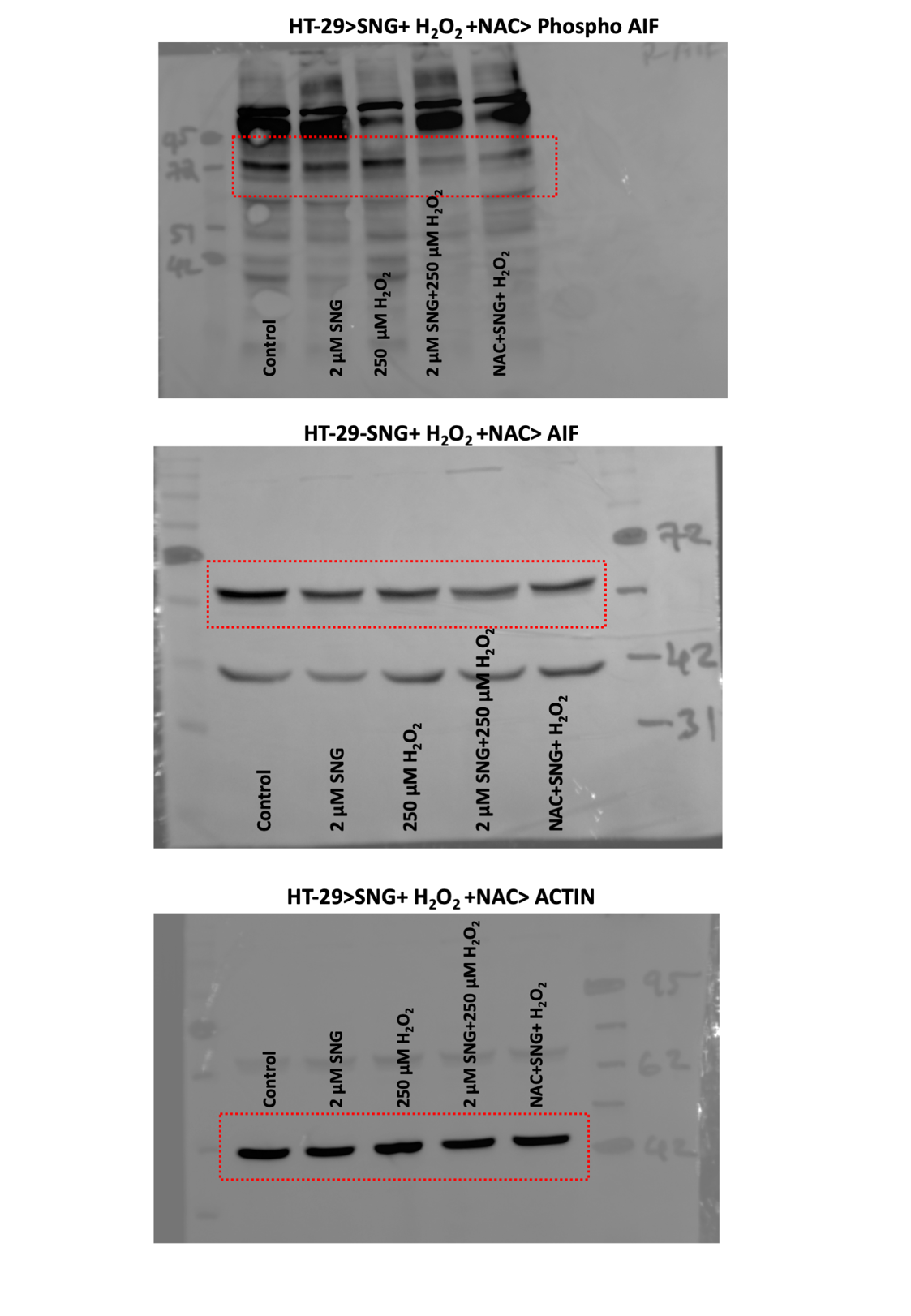


**Fig 7 C**


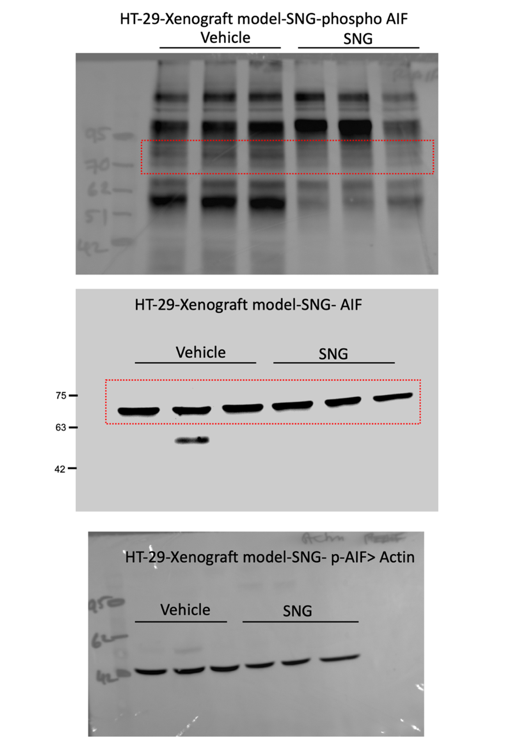


**Supplimentary Fig 4**


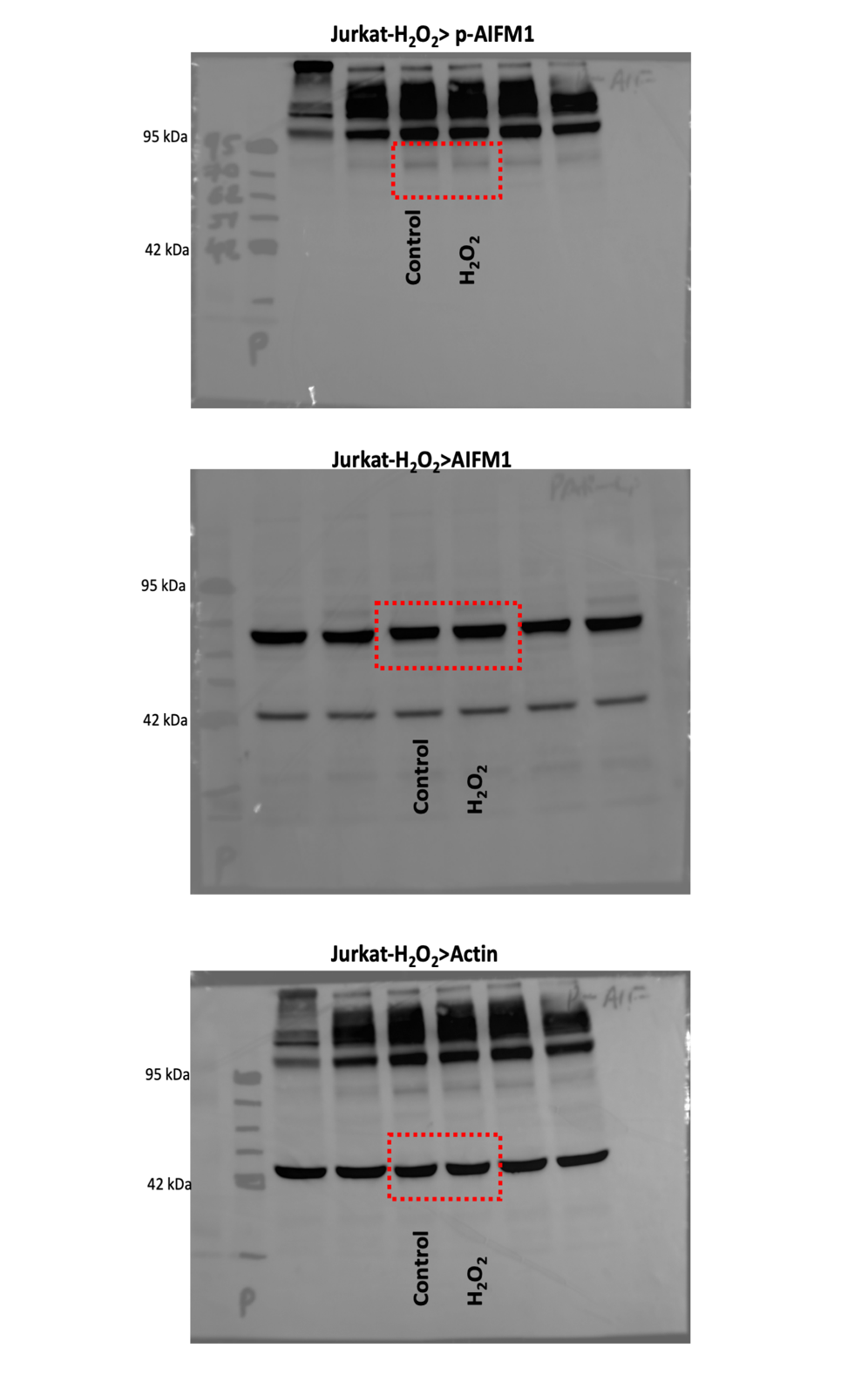

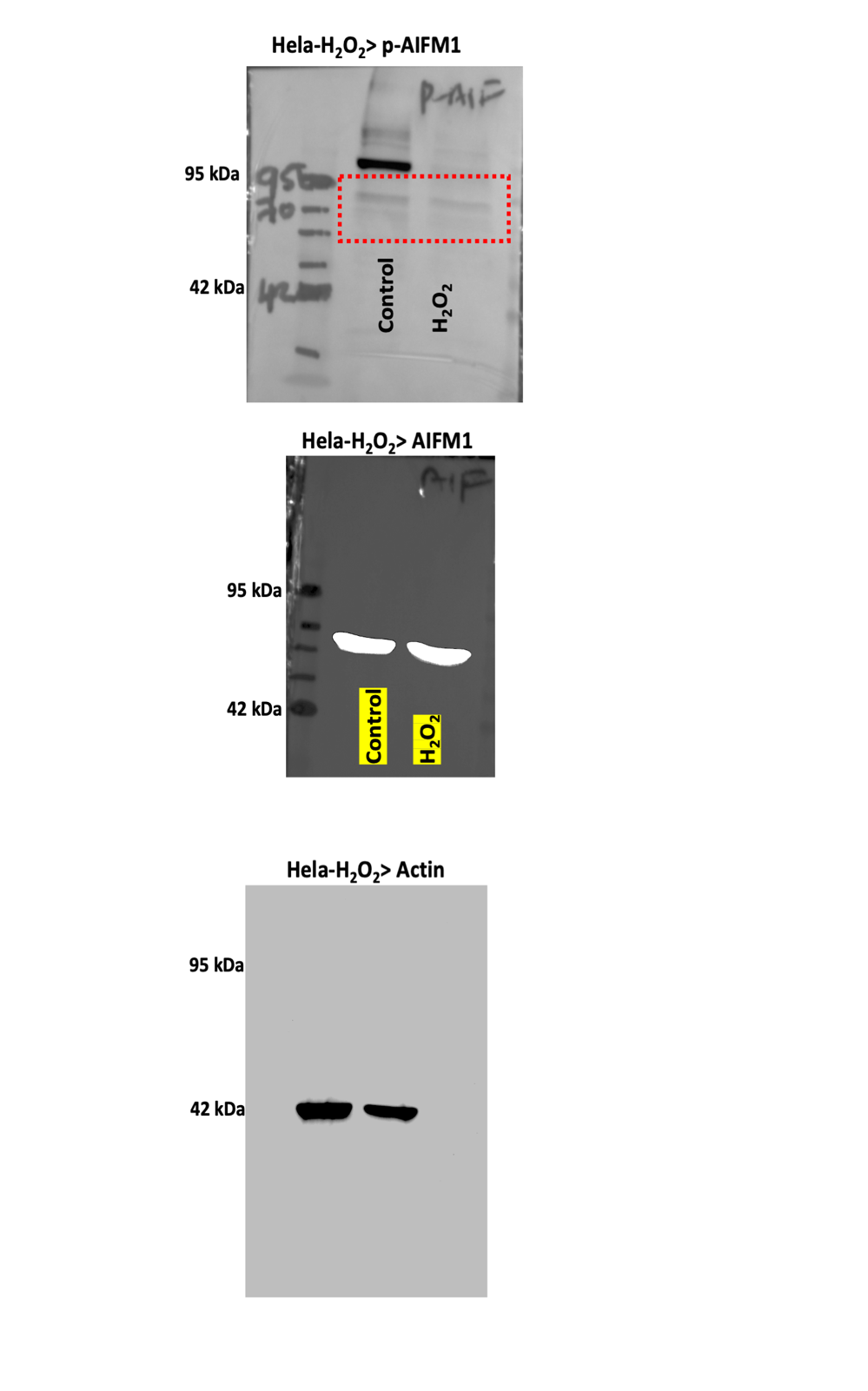


**
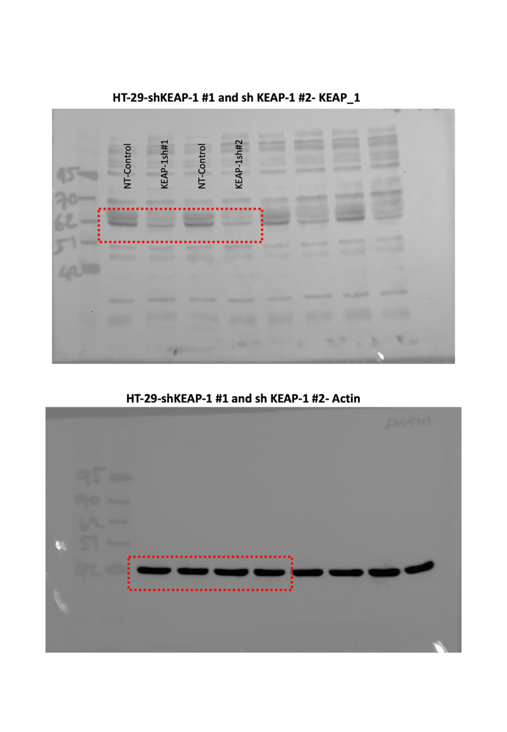
Supplimentary Fig 5**

**Supplimentary Fig 6**


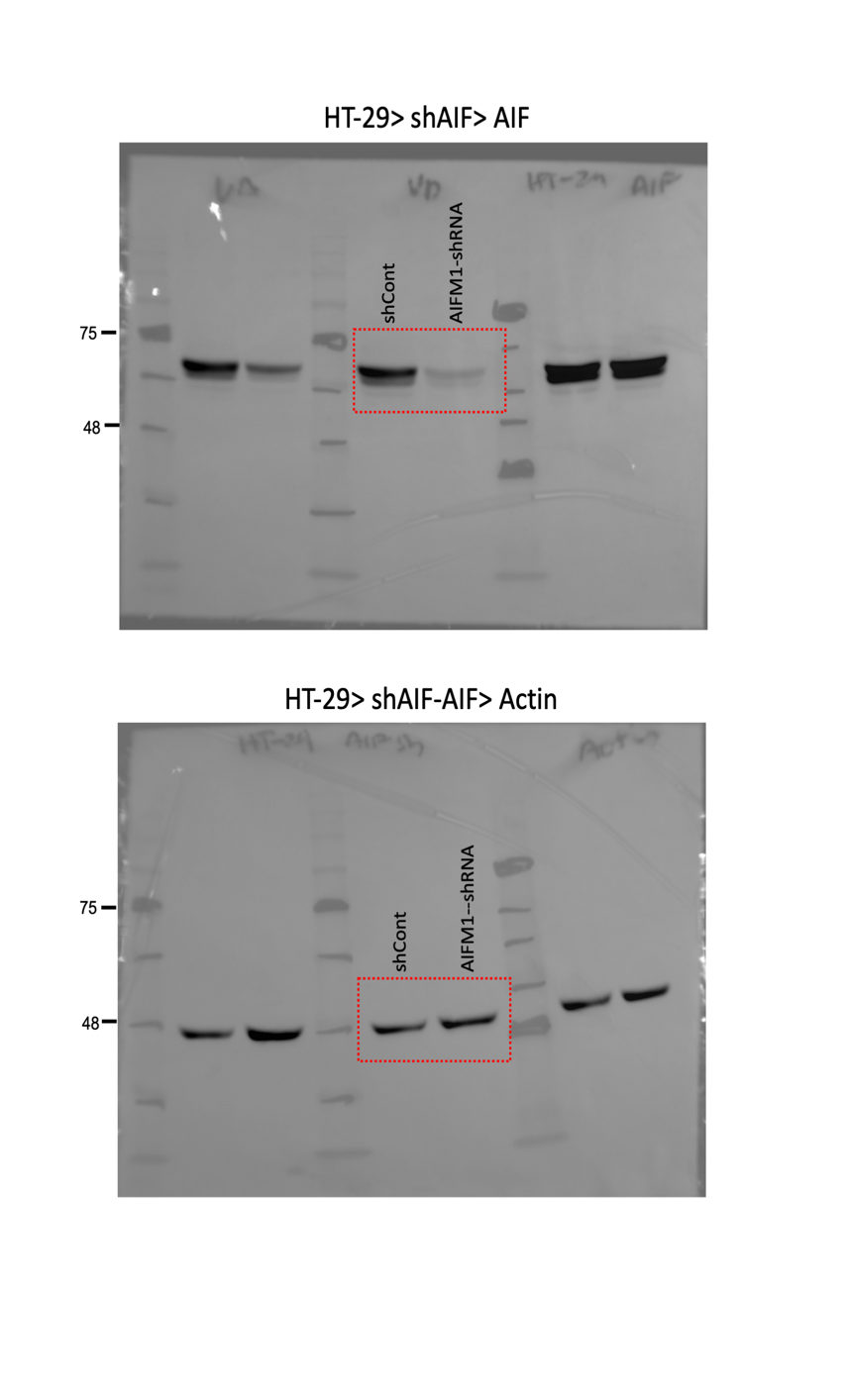

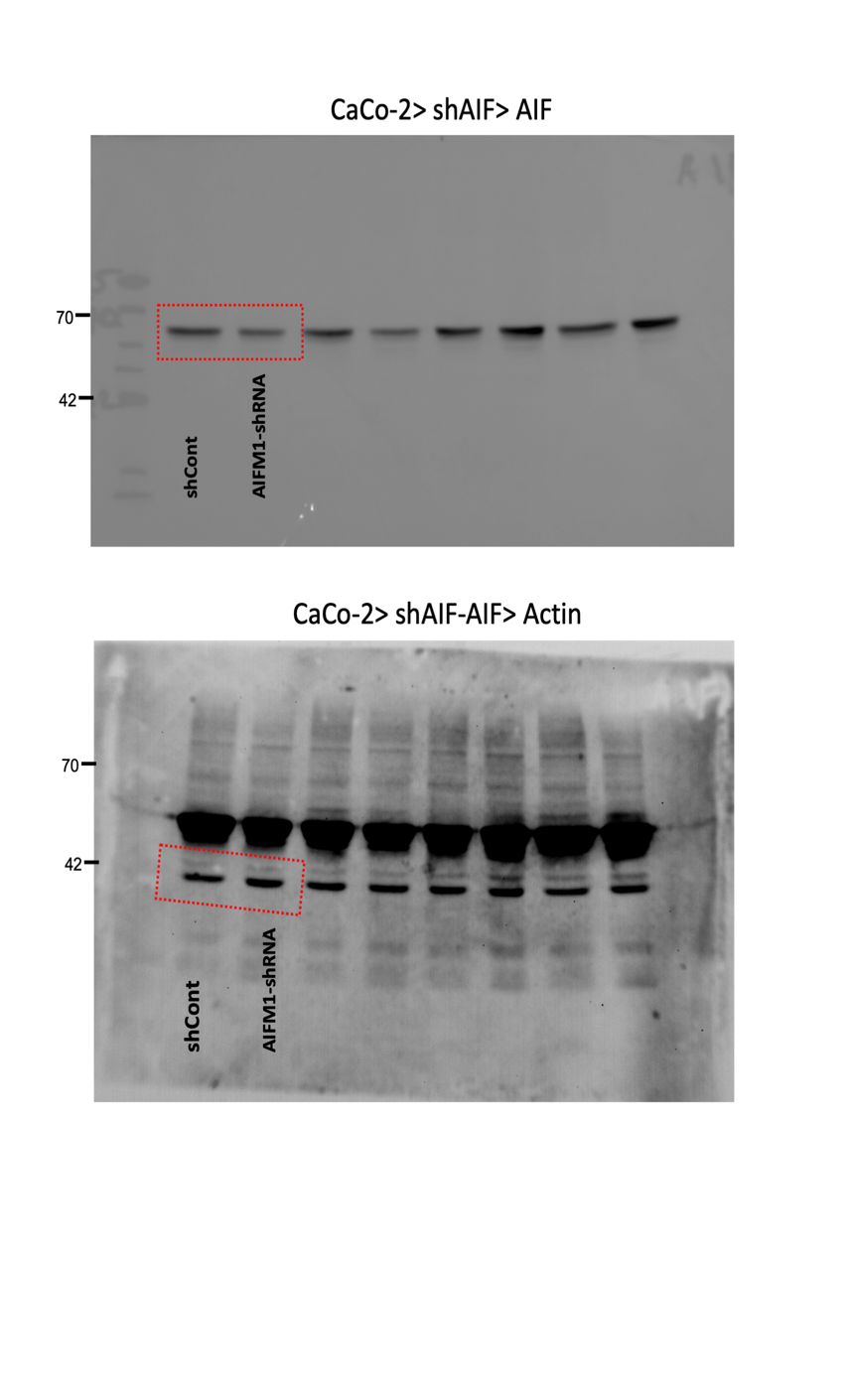

Supplement: Supplementary file 5 — Original Western Blots [file 41420_2023_1376_MOESM5_ESM.docx]
